# Stereodivergent Olefination of Enantioenriched Boronic Esters

**DOI:** 10.1002/anie.201610387

**Published:** 2016-12-13

**Authors:** Roly J. Armstrong, Cristina García‐Ruiz, Eddie L. Myers, Varinder K. Aggarwal

**Affiliations:** ^1^ School of ChemistryUniversity of BristolCantock's CloseBristolBS8 1TSUK

**Keywords:** alkenes, boron, isomers, oxidation, selenium

## Abstract

A stereodivergent coupling reaction between vinyl halides and boronic esters is described. This coupling process proceeds without a transition‐metal catalyst, instead proceeding by electrophilic selenation or iodination of a vinyl boronate complex followed by stereospecific *syn* or *anti* elimination. Chiral, nonracemic boronic esters could be coupled with complete enantiospecificity. The process enables the highly stereoselective synthesis of either the *E* or *Z* alkene from a single isomer of a vinyl coupling partner.

The stereodefined synthesis of multiply substituted alkenes continues to attract attention because of their importance in natural products, pharmaceuticals, and materials.^1^ Of the many methods that exist, the Suzuki–Miyaura cross‐coupling reaction is widely used as it enables the direct union between vinyl halides and boronates.^2^ However, whilst sp^2^‐hybridized and primary organoborons couple efficiently, the corresponding reactions with secondary or tertiary boronic esters do not, thus limiting its broader use.^3^ An attractive feature of the Suzuki–Miyaura reaction is that it is stereospecific,^4^ but if one geometrical isomer of a given coupling partner is much easier to make than the other, as is often the case, this again limits its wider application.^5^ Herein, we address both of these issues and describe a stereospecific method for coupling secondary boronic esters with a single geometrical isomer of a vinyl halide, thus leading to either the *E* or *Z* isomer of the coupled product.

To develop a solution to these problems, we turned our attention to the Zweifel olefination.^6^ In this process, a vinyl metal is combined with a boronic ester, resulting in the formation of a boronate complex. Addition of iodine to the double bond gives an intermediate iodonium ion, which triggers a 1,2‐metallate rearrangement leading to a β‐iodoboronic ester. Upon treatment with methoxide, the β‐iodoboronic ester undergoes *anti* elimination to produce a single isomer of the resulting alkene (Scheme 1 c).^7^ We reasoned that if the *anti* elimination could be diverted to a *syn* elimination instead, then we should be able to access the alternative geometric isomer from the same geometry of the vinyl metal. Such *syn* elimination processes are known for boron, but most are specific for trialkyl boranes, the most notable example being Zweifel's use of cyanogen bromide, involving the intermediacy of a β‐bromo cyanoborane (Scheme 1 a).^8^ In the realm of the more atom economic and readily available boronic esters, *syn* elimination processes have been reported for substrates with β‐positioned N‐oxide (Scheme 1 b),^9^ and carbamate moieties.^10^ However, the lack of suitable electrophiles for introducing such functionality within our envisioned manifold, led us to consider *syn* elimination of a β‐selenoxyboronic ester. We speculated that such an intermediate could be obtained through the electrophilic addition of ArSeCl to a vinyl boronate followed by oxidation. If the selenoxide could attack a boron atom instead of a hydrogen atom, with formation of the strong B−O bond providing the driving force, then the desired *syn* elimination should result. However, a successful process would require a) chemoselective oxidation of a selenide in the presence of an easily oxidizable boronic ester^11^ and b) selective elimination of the boronic ester in the presence of a β‐hydrogen atom.^12^

**Scheme 1:**
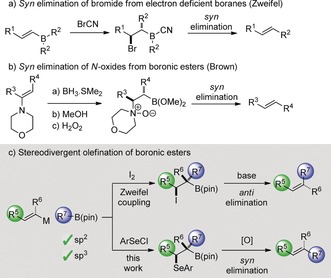
Previous work and strategy for stereodivergent olefination. pin=pinacol.

We commenced our study with *E*‐vinyl bromide **1** (Table 1). Lithium–halogen exchange followed by addition of enantioenriched boronic ester **2** gave the desired vinyl boronate complex. Upon addition of sodium methoxide in methanol followed by iodine we isolated coupled product **3 a** in 80 % yield as a single *Z*‐isomer with complete enantiospecificity (entry 1).^13^ Moreover, we were pleased to find that when the same boronate complex was treated with PhSeCl, smooth conversion into the desired β‐selenoboronic ester was observed. This crude intermediate could be treated with sodium methoxide in methanol, thus triggering an *anti* elimination to afford the product in 80 % yield as a single *Z* isomer without any loss of enantiomeric purity (entry 2). In certain cases (see later) this procedure could serve as a useful alternative to the Zweifel coupling.

**Table 1:** Optimization of reaction conditions for stereodivergent cross‐coupling.^[a]^ 
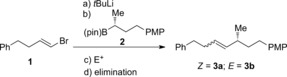

Entry	E^+^	Elimination	Yield [%]^[b]^	*E*/*Z*
1^[c,d]^	I_2_	NaOMe (MeOH)	79 (80)	<2:98
2^[d]^	PhSeCl	NaOMe (MeOH)	78 (80)	<2:98
3	PhSeCl	H_2_O_2_ (H_2_O)	66	55:45
4	PhSeCl	*m*CPBA (THF)	71	5:95
5^[e]^	PhSeCl	*m*CPBA (CH_2_Cl_2_)	71	>98:2
6^[d,e]^	PhSeCl	*m*CPBA (THF)	75 (74)	>98:2

[a] Vinyl bromide (1.05 equiv), *t*BuLi (2.1 equiv), THF, −78 °C; then boronic ester (1.0 equiv), THF, −78 °C; then either I_2_ (1.2 equiv), THF/MeOH (3:1), 0 °C or PhSeCl (1.2 equiv), THF, −78 °C to RT; then elimination. [b] Determined by NMR analysis using 1,3,5‐trimethoxybenzene as an internal standard. Values within parentheses indicate the yield of isolated product. [c] NaOMe added prior to I_2_. [d] With enantioenriched **2** (96:4 e.r.) the product was obtained in 100 % e.s. [e] Reaction mixture filtered through silica gel prior to oxidation. *m*CPBA=*meta*‐chloroperbenzoic acid, PMP=*para*‐methoxyphenyl, THF=tetrahydrofuran.

We next turned our attention to the development of a protocol for *syn* elimination. Upon treatment of a THF solution of the crude β‐selenoboronic ester with aqueous hydrogen peroxide we obtained the coupled product in modest yield, but as a 55:45 *E*/*Z* ratio (entry 3). When *m*CPBA was employed as an oxidant we obtained the undesired *Z* isomer almost exclusively (entry 4). However, these reactions showed that chemoselective oxidation of the selenide did indeed occur in the presence of the boronic ester. Remarkably, we found that filtration of the crude reaction solution of β‐selenoboronic ester through a short plug of silica gel, followed by addition of *m*CPBA in dichloromethane resulted in a complete switch in selectivity and **3 b** was obtained as a single *E* isomer in 71 % yield (entry 5).^14^ Changing the oxidation solvent to THF afforded the product in slightly improved yield, and still with complete stereo‐ and enantiospecificity (entry 6).

Having identified optimal reaction conditions for generating either the *E* or *Z* alkene, we explored scope, initially focusing on variation of the boronic ester (Table 2). With nonbranched secondary boronic esters, both coupling processes proceeded efficiently to provide the corresponding alkenes in excellent yields and levels of selectivity, together with complete enantiospecificity. Notably, boronic ester **6**, bearing an electron‐rich trisubstituted alkene, and ester‐containing boronic ester **4** underwent efficient coupling with no evidence of side reactions. Benzylic, natural‐product‐derived and menthol‐derived boronic esters **8**, **12**, and **14**, coupled smoothly under our optimized reaction conditions for selenation‐oxidation (Method B; >98:2 *E*/*Z*) but with reduced selectivity in the Zweifel procedure (Method A). This issue is addressed later. A vinyl boronic ester could also be employed, thus enabling the stereodivergent synthesis of *Z*,*E* or *E*,*E* dienes **17 a** and **17 b** in 71 % and 79 % yield, respectively. We were also able to extend the process to the coupling of vinyl boronic esters and organolithiums, thus accessing styrenes **18 a** and **18 b** with excellent yields and high stereoselectivity.

**Table 2:** Stereodivergent coupling: boronic ester scope.^[a]^ 
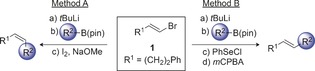

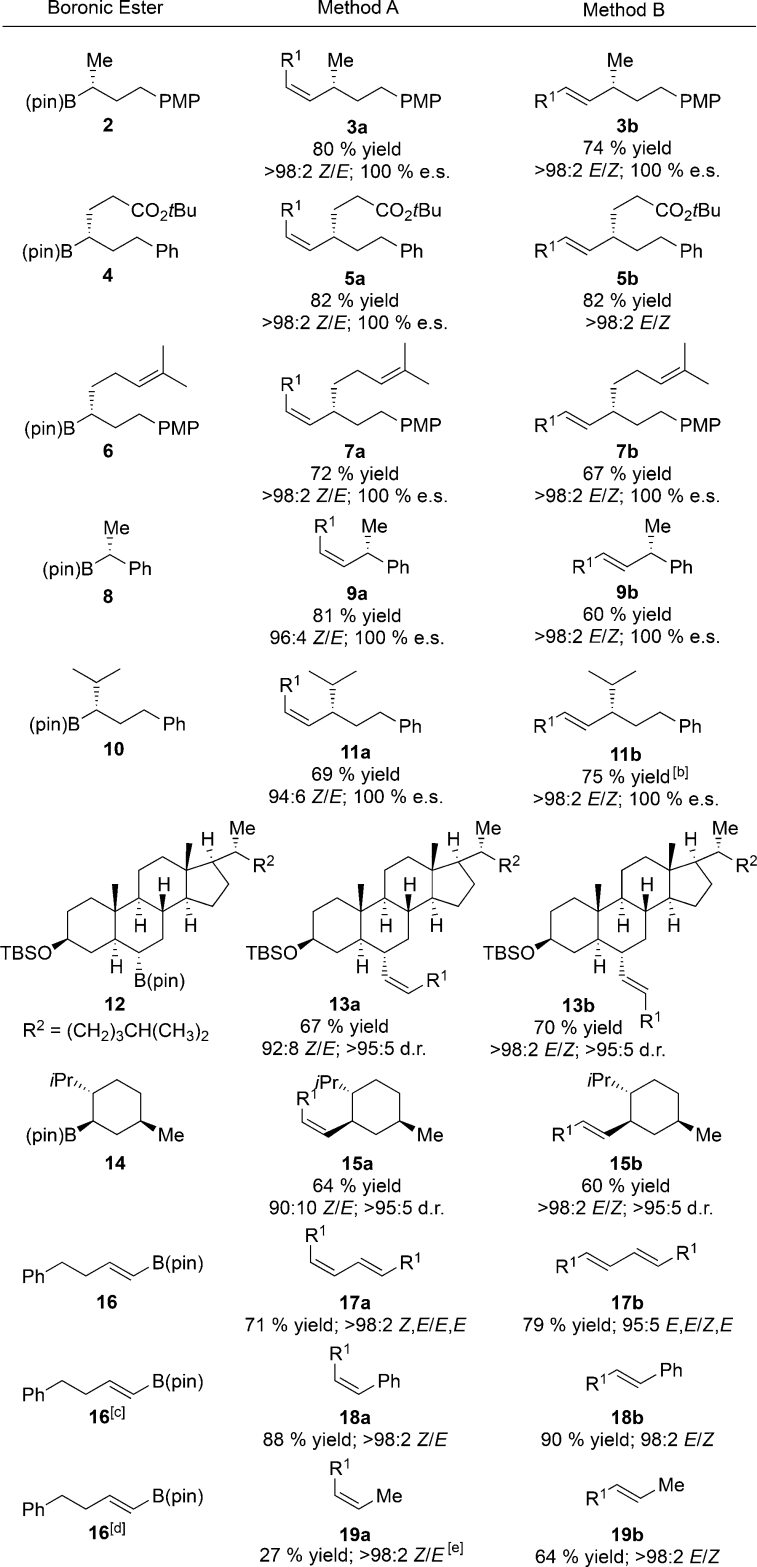

[a] Vinyl bromide (1.05 equiv), *t*BuLi (2.1 equiv), THF, −78 °C; then boronic ester (1.0 equiv), THF, −78 °C; then either NaOMe (3.0 equiv), I_2_ (1.2 equiv), THF/MeOH (3:1), 0 °C or PhSeCl (1.2 equiv), THF, −78 °C to RT; then SiO_2_ filtration; then *m*CPBA (2.0 equiv), THF, −78 °C to −45 °C. [b] PhSeSePh removed by oxidation with H_2_O_2_. [c] Ate complex formed with **16** and PhLi (1.05 equiv). [d] Ate complex formed with **16** and MeLi (1.05 equiv). [e] (*E*)‐(4‐Iodobut‐3‐en‐1‐yl)benzene also isolated in 29 % yield. TBS=*tert*‐butyldimethylsilyl.

Synthesis of methyl‐substituted‐alkenes by Zweifel coupling has previously been reported to be challenging because of the poor migratory aptitude of a methyl group.6g In line with this observation, we found that coupling of **16** with methyl lithium led to **19 a** in moderate yield, an issue which is addressed later.^15^ We were pleased to find that our selenation‐oxidation protocol enabled the synthesis of **19 b** in good yield and excellent stereoselectivity.

We were interested in the trend where bulkier coupling partners resulted in lower *Z*/*E* selectivity in the Zweifel reaction. In this process, the normally favored *anti*‐elimination pathway brings the two substituents into close proximity, and the barrier to elimination will increase as the groups get larger. This scenario may allow the less favored *syn*‐elimination process to compete, thus leading to a mixture of isomers (Scheme 2 A). We rationalized that if we could disfavor the *syn*‐elimination pathway further by using a poorer leaving group, for example a selenide in place of an iodide, high *Z* selectivity should be restored. Therefore, we turned to the reaction conditions we had previously developed for methoxide‐promoted *anti* elimination of a β‐selenoboronic ester (Table 1, entry 2).

**Scheme 2:**
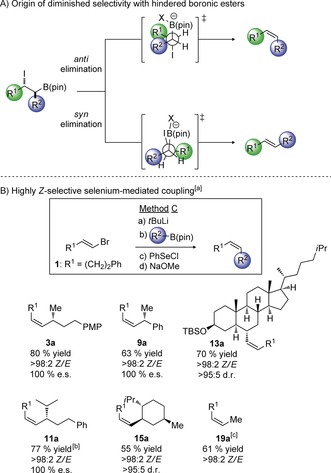
Highly *Z*‐selective olefination. [a] Vinyl bromide (1.05 equiv), *t*BuLi (2.1 equiv), THF, −78 °C; then boronic ester (1.0 equiv), THF, −78 °C; then PhSeCl (1.2 equiv), THF, −78 °C to RT; then NaOMe (5.0–20.0 equiv), THF/MeOH (1:1), 0 °C or RT. [b] PhSeSePh removed by oxidation with H_2_O_2_. [c] Ate complex formed with **16** and MeLi (1.05 equiv).

Gratifyingly, when we carried out the cross coupling of benzylic boronic ester **8** under these conditions, the coupled product **9 a** was obtained in 63 % yield as a single *Z*‐isomer (Scheme 2 B). Moreover, when these conditions were applied to other *Z*‐selective couplings that had previously given lower *Z* selectivity, the products **11 a**, **13 a** and **15 a** were all obtained in good to excellent yields as a single alkene isomer. Additionally, under these conditions, the coupling of **16** with MeLi proceeded smoothly, thus affording isomerically pure **19 a** in 61 % yield.

We next turned our attention to varying the vinyl halide coupling partner, focusing our attention on trisubstituted vinyl bromides, as stereospecific synthesis of trisubstituted alkenes is often more difficult (Table 3).^16^ We were delighted to find that commercially available *E*‐2‐bromobut‐2‐ene (**20**) could be successfully coupled with enantioenriched boronic ester **2** to afford either isomer of the coupled product with excellent yields and stereoselectivity.^17^ The same coupled products could be obtained through stereodivergent coupling of isomeric *Z*‐bromide **22**. For many trisubstituted vinyl halides only one geometrical isomer can be readily accessed. For example, vinyl bromide **23**, prepared stereoselectively by hydrozirconation of the corresponding alkyne,^18^ underwent coupling to afford either **24 a** or **24 b** in excellent yields and with near perfect stereocontrol. Similarly, vinyl bromide **25**, prepared from 2‐butyn‐1‐ol by hydroxyl‐directed hydroalumination,^19^ underwent stereodivergent coupling with **2** to afford the coupled products **26 a** and **26 b** in 61 % and 55 % yield, respectively. Finally, the methodology can be applied in settings relevant to complex molecule synthesis, as illustrated with boronic ester **27**, which is readily prepared in high *Z*‐selectivity by cross‐metathesis. Reaction with an alkyl lithium derived from the Roche ester and subsequent olefination gave either the *E* or *Z* trisubstituted alkene with high selectivity. *Z*‐alkene **28 a** represents the C9−C17 fragment of discodermolide and its ease of synthesis is especially notable. In Novartis's formidable synthesis of discodermolide the synthesis of this trisubstituted alkene was one of the most challenging reactions they encountered.^20^

**Table 3:** Stereodivergent coupling: vinyl partner scope.^[a]^ 
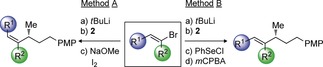

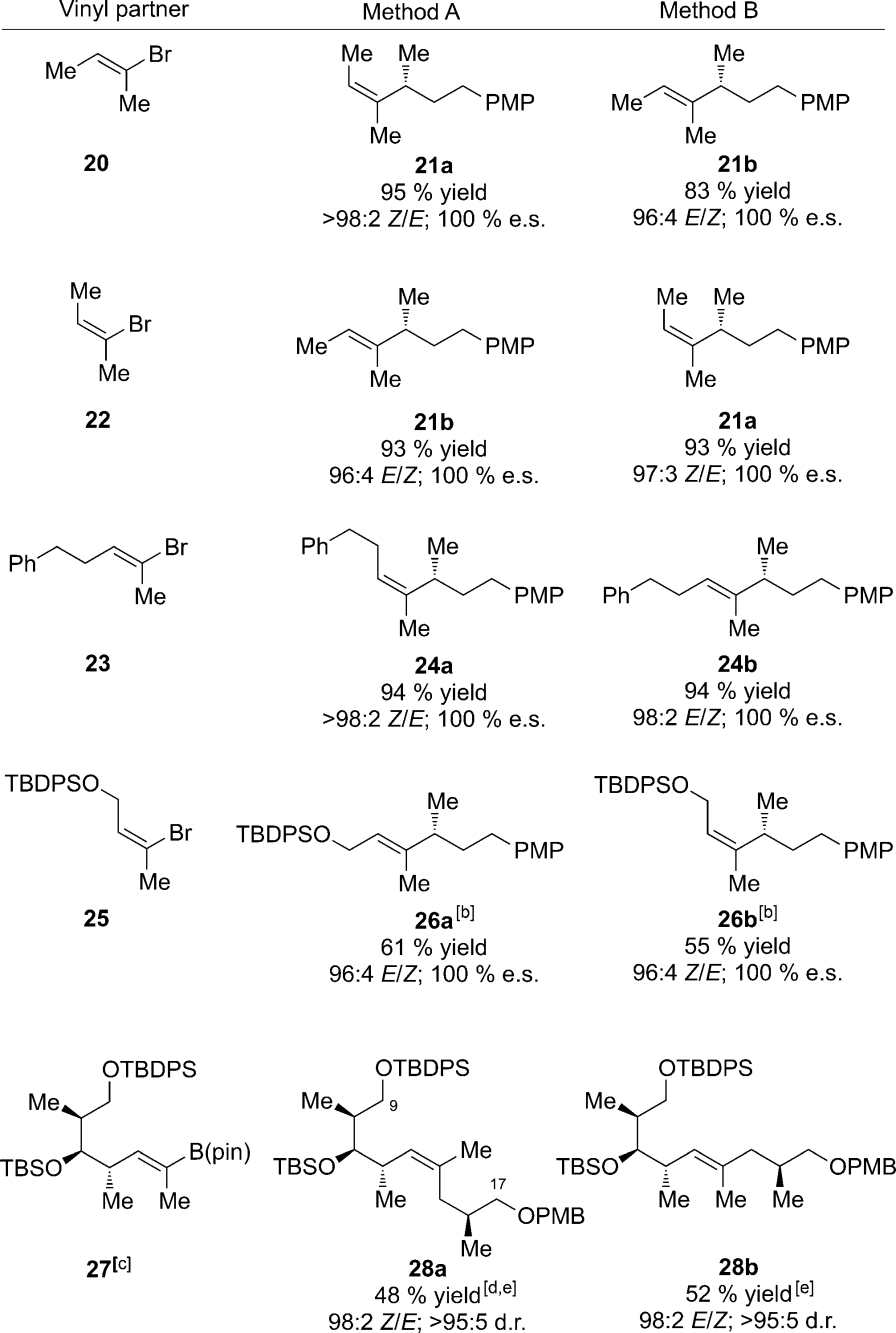

[a] Vinyl bromide (2.0 equiv), *t*BuLi (4.0 equiv), THF, −78 °C; then boronic ester (1.0 equiv), THF, −78 °C; then either NaOMe (3.0 equiv), I_2_ (1.2 equiv), THF/MeOH (3:1), −78 °C to 0 °C *or* PhSeCl (1.2 equiv), 1:1 CF_3_CH_2_OH/THF, −78 °C to RT; then SiO_2_ filtration; then *m*CPBA (2.0 equiv), THF, −78 °C to −45 °C. [b] Vinyl lithium formed in situ by addition of *t*BuLi to a mixture of **2** and **25**. [c] Boronate complex formed between (*S*)‐LiCH_2_CH(CH_3_)CH_2_OPMB and **27**. [d] Using Method C. [e] The intermediate β‐selenoboronate was isolated (yield based on 2 steps). PMB=*para*‐methoxybenzyl, TBDPS=*tert*‐butyldiphenylsilyl.

The putative *syn* elimination of β‐selenoxyboronic esters was investigated by DFT calculations using the B3LYP functional with a cc‐PVDT(H,C)/cc‐PVTZ(B,O)/RECP‐DZ(Se) basis set. Both diastereomers of the β‐selenoxyboronic ester (diastereomeric at the selenium center) which would give (*E*)‐but‐2‐ene (Scheme 3) show global minima involving a strong interaction between the selenoxide oxygen atom and the boron atom. These conformers were primed to undergo elimination with remarkably low barriers (0.4–2.2 kcal mol^−1^), which were more accessible than rotation about the Se‐C‐C‐B dihedral.^21^ Traditional selenoxide eliminations, involving the expulsion of phenylselenenic acid from other conformers, were also calculated for comparison, and showed significantly higher barriers. Interestingly, elimination to give the vinyl boronic ester was calculated to be considerably more facile than elimination to give the allyl boronic ester (5.6–7.0 versus 10.0–11.0 kcal mol^−1^), thus suggesting that hydrogen atoms geminal to trivalent boron substituents are more activated. This observation is in agreement with related eliminations of β‐sulfoxysilanes, which undergo faster eliminations (to give vinyl silanes) relative to nonsilyl derivatives.12a,12b

**Scheme 3:**
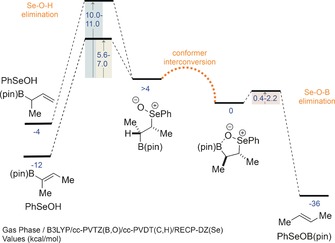
Computational results.

In conclusion, we have developed an efficient method for the stereodivergent coupling of vinyl halides with boronic esters. This reaction proceeds with no requirement for a transition metal and tolerates aliphatic, vinylic, and aromatic coupling partners. Where chiral, nonracemic boronic esters were employed, the coupling took place with complete enantiospecificity, and the process has been successfully applied to the stereodivergent synthesis of trisubstituted alkenes. DFT studies probing the mechanism of this interesting transformation suggest that *syn* elimination of a β‐selenoxyboronic ester is a remarkably facile process. We believe that this approach will find widespread application in the stereoselective synthesis of polysubstituted alkenes.

## Conflict of interest

The authors declare no conflict of interest.

## Supporting information

As a service to our authors and readers, this journal provides supporting information supplied by the authors. Such materials are peer reviewed and may be re‐organized for online delivery, but are not copy‐edited or typeset. Technical support issues arising from supporting information (other than missing files) should be addressed to the authors.

Supplementary
