# Supplementary material for: Stereodivergent Olefination of Enantioenriched Boronic Esters
Source: Angew Chem Int Ed Engl. 2016 Dec 13;56(3):786–90. doi: 10.1002/anie.201610387 (PMC5347846; doi:10.1002/anie.201610387)

Angewandte Chemie
Eine Zeitschrift der Gesellschaft Deutscher Chemiker

Supporting Information

Stereodivergent Olefination of Enantioenriched Boronic Esters

*Roly J. Armstrong, Cristina García-Ruiz, Eddie L. Myers, and Varinder K. Aggarwal**

anie_201610387_sm_miscellaneous_information.pdf

Contents

**1.1**	**General Information**	**S2**
**1.2**	**General Experimental Procedures**	**S3**
2.2.1	*General Procedure A: Z-Selective Coupling*	**S3**
2.2.2	*General Procedure B: E-Selective Coupling*	**S4**
2.2.3	*General Procedure C: Modified Z-Selective Coupling*	**S5**
**1.3**	**Experimental Procedures**	**S6**
1.3.1	*Starting Material Synthesis*	**S6**
1.3.2	*Stereodivergent Coupling*	**S15**
**2.**	**Computational Investigations**	**S60**
**3.1**	**References**	**S91**
3.1.1	References For Supporting Information	**S91**
3.1.2	Ref. [20] from Manuscript With Extended Author List	**S93**
**4.**	**NMR spectra**	**S94**

1.1 General Information

Reactions were carried out in flame-dried glassware under an atmosphere of nitrogen unless stated otherwise. Room temperature refers to 20-25 °C. Temperatures of 0 °C were obtained using an ice/water bath. Temperatures of -78 °C were obtained using a dry ice/acetone bath. Temperatures of -45 °C were obtained using a dry ice/acetonitrile bath. Reflux conditions were obtained using an oil bath equipped with a contact thermometer.

Dichloromethane, diethyl ether and tetrahydrofuran were purified by filtration through activated alumina columns employing the method of Grubbs *et al*.[1] All other solvents were used as supplied without prior purification. *n*-Butyllithium was purchased from Acros Organics as a 1.6 M solution in hexane and the molarity was confirmed by titration against *N*-benzylbenzamide.[2] *tert*-Butyllithium was purchased from Sigma Aldrich as a 1.7 M solution in pentane and the molarity was confirmed by titration against *N*-benzylbenzamide.[1] All other reagents were used directly as supplied by major chemical suppliers, or following purification procedures described by Perrin and Armarego.[3]

Thin layer chromatography was performed on Merck Kieselgel 60 F_{254} 0.25 mm precoated aluminium plates. Product spots were visualized under UV light (λ = 254 nm) and/or by staining with potassium permanganate solution. Flash chromatography was performed using VWR silica gel 60 (40-63 µm particle size) using head pressure by means of a nitrogen line.

NMR spectroscopy was carried out using Joel Lambda 300, Joel ECP 400, Varian 400-MR, VNMRS500a or Bruker Cryo 500 MHz spectrometers in the deuterated solvent stated, using the residiual non-deuterated solvent signal as an internal reference. Chemical shifts are quoted in ppm with signal splittings recorded as singlet (s), doublet (d), triplet (t), quartet (q), quintet (qn), sextet (sex), septet (sept), octet (oct), nonet (non) and multiplet (m). The abbreviation br. denotes broad. Coupling constants, *J*, are measured to the nearest 0.1 Hz and are presented as observed.

Infrared spectra were recorded neat on a PerkinElmer Spectrum One FT-IR spectrometer equipped with an attenuated total reflectance attachment with internal calibration. Absorption maxima (λ_{max}) are quoted in wavenumbers (cm^{-1}).

Mass spectra were recorded by the University of Bristol, School of Chemistry departmental mass spectrometry service using electron impact ionisation (EI), chemical ionisation (CI) or electrospray ionisation (ESI) techniques for low- and high-resolution mass spectra. HRMS EI and CI were performed on a VG Analytical Autospec mass spectrometer at 70 eV. HRMS ESI was performed on either a Bruker Daltonics Apex IV, 7-Tesla FT-ICR or microTOF II. Nanospray was performed on a Synapt G2S mass spectrometer. Samples were submitted in CH_2Cl_2.

Optical rotations were recorded on a Bellingham and Stanley Ltd. ADP220 polarimeter at 589 nm in a cell with a path length of 1 dm (using the sodium D line, 589 nm). Concentrations are reported in g/100 mL. Temperatures are reported in °C.

Chiral HPLC was performed on a Agilent 1100 Series HPLC unit equipped with UV-vis diode-array detector monitored fitted with the appropriate Daicel Chiralpak column (dimensions: 0.46 cm ø x 25 cm) and corresponding guard column (0.4 cm ø x 1 cm). Wavelengths (λ) are reported in nm, retention times (t_R) are reported in minutes and solvent flow rates are reported in mL min^{-1}.

Chiral GC was performed on an Agilent 7890A using the appropriate Chiraldex column. Retention times (t_R) are reported in minutes and gas flow rates are reported in mL min^{-1}.

1.2 General Experimental Procedures

1.2.1 General Procedure A: *Z*-Selective Coupling

A stirred solution of vinyl halide (1.05 eq.) in THF (5 mL/mmol vinyl boronic ester) was cooled to -78 ^{o}C and a solution of *tert*-butyllithium (1.7 M in pentane, 2.1 eq.) was added dropwise. The resulting solution was stirred at -78 ^{o}C for 30 minutes and then a solution of boronic ester (1.0 eq.) in THF

(5 mL/mmol boronic ester) was added dropwise. The resulting solution was stirred at -78 ^{o}C for 30 minutes and then warmed to 0 ^{o}C and stirred for 2 minutes. After this time, a suspension of sodium methoxide (3 M in MeOH, 3.0 eq.) was added in a single portion followed by dropwise addition of a solution of iodine (0.5 M in MeOH, 1.2 eq.). The resulting mixture was stirred at 0 ^{o}C for 30 minutes and then saturated aqueous sodium sulfite was added followed by water and dichloromethane. The organic layer was separated and the aqueous layer was extracted twice with dichloromethane. The combined organic extracts were dried over anhydrous magnesium sulfate, filtered and concentrated under reduced pressure. Purification of the residue *via* column chromatography (see experimental methods section for specific details) afforded the corresponding alkene.

1.2.2 General Procedure B: *E*-Selective Coupling

A stirred solution of vinyl halide (1.05 eq.) in THF (5 mL/mmol vinyl boronic ester) was cooled to -78 ^{o}C and a solution of *tert*-butyllithium (1.7 M in pentane, 2.1 eq.) was added dropwise. The resulting solution was stirred at -78 ^{o}C for 30 minutes and then a solution of boronic ester (1.0 eq.) in THF (5 mL/mmol boronic ester) was added dropwise. The resulting solution was stirred at -78 ^{o}C for 30 minutes and a solution of PhSeCl (1.0 M in THF, 1.2 eq.) was added dropwise. The resulting solution was stirred at -78 ^{o}C for 30 minutes and then warmed to room temperature and stirred for a further 15 minutes and then filtered through a short plug of silica gel washing with diethyl ether. The filtrate was concentrated under reduced pressure and the residue was dissolved in THF (10 mL/mmol boronic ester). The resulting stirred solution was cooled to -78 ^{o}C and a solution of *m*CPBA (0.2 M in THF, 2.0 eq.) was added dropwise. The resulting solution was warmed to -45 ^{o}C (acetonitrile/dry ice bath) and stirred for 30 minutes. After this time dimethylsulfide (20 eq.) was added and the mixture was allowed to warm to room temperature and then filtered through a short plug of silica gel, washing with diethyl ether. The filtrate was concentrated under reduced pressure. Purification of the residue *via* column chromatography (see experimental methods section for specific details) afforded the corresponding alkene.

1.2.3 General Procedure C: Modified *Z*-Selective Coupling

A stirred solution of vinyl halide (1.05 eq.) in THF (5 mL/mmol boronic ester) was cooled to -78 ^{o}C and a solution of *tert*-butyllithium (1.7 M in pentane, 2.1 eq.) was added dropwise. The resulting solution was stirred at -78 ^{o}C for 30 minutes and then a solution of boronic ester (1.0 eq.) in THF (5 mL/mmol boronic ester) was added dropwise. The resulting solution was stirred at -78 ^{o}C for 30 minutes and a solution of PhSeCl (1.0 M in THF, 1.2 eq.) was added dropwise. The resulting solution was stirred at -78 ^{o}C for 30 minutes and then warmed to room temperature and stirred for a further 15 minutes and then cooled to 0 ^{o}C. A solution of sodium methoxide (0.5 M in MeOH, 5.0 eq.) was added dropwise and the resulting solution was stirred at 0 ^{o}C for 2 hours and then saturated aqueous sodium sulfite was added followed by water and dichloromethane. The organic layer was separated and the aqueous layer was extracted twice with dichloromethane. The combined organic extracts were dried over anhydrous magnesium sulfate, filtered and concentrated under reduced pressure. Purification of the residue *via* column chromatography (see experimental methods section for specific details) afforded the corresponding alkene.

1.3 Experimental Procedures

1.3.1 Starting Material Synthesis

(*E*)-(4-Bromobut-3-en-1-yl)benzene, 1

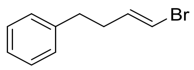

1

4-Phenyl-1-butyne (926 mg, 7.11 mmol) was stirred neat at room temperature and diisobutylaluminum hydride solution (1.0 M in hexane, 7.82 mL, 7.82 mmol) was added dropwise. The resulting solution was heated to 50 °C for 3 hours and then cooled to room temperature and diluted with anhydrous diethyl ether (4 mL). The resulting solution was cooled to -45 °C and *N*-bromosuccinimide (1.52 g, 8.53 mmol) was added portionwise. The resulting suspension was warmed to 0 °C and stirred for 15 min and then warmed carefully to room temperature [caution: exotherm observed] and stirred for 1 h. The resulting black solution was poured into a rapidly stirred ice cold mixture of pentane and 3M aqueous HCl. The organic layer was separated and the aqueous layer was extracted twice with pentane. The combined organic extracts were washed with brine and then dried over anhydrous magnesium sulfate, filtered and concentrated under reduced pressure. Purification of the residue *via* column chromatography eluting with hexane afforded the title compound **1** as a colourless oil (1.11 g, 74 %, >98:2 *E*/*Z*). The spectral data matched that previously reported in the literature.[4] This material could be stored over copper turnings in the dark at -18 °C for several months without isomerization.

^{1}H NMR (400 MHz, $CDCl_3$) δ_H = 7.32 – 7.26 (m, 2H), 7.23 – 7.14 (m, 3H), 6.21 (dt, J = 13.5, 7.2 Hz, 1H), 6.04 (dt, J = 13.6, 1.4 Hz, 1H), 2.74 – 2.68 (m, 2H), 2.39 – 2.32 (m, 2H);

^{13}C NMR (101 MHz, $CDCl_3$) δ_C = 140.9, 137.2, 128.5, 128.5, 126.2, 105.1, 35.1, 34.8;

FTIR (neat) ν/cm^{-1} = 3026, 2928, 1620, 1496, 1454, 1218, 934, 743, 698;

LRMS (EI^+): calculated for $C_{10}H_{11}Br$ = 210, mass found = 210.

(*E*)-(4-Bromopent-3-en-1-yl)benzene, 23

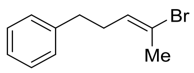

23

By analogy to a literature procedure,[5,6] to a flask protected from light was added Cp_2ZrCl_2 (487 mg, 1.66 mmol) and degassed THF (2 mL). The solution was cooled to 0 °C and diisobutylaluminum hydride solution (1 M in hexane, 1.7 mL, 1.66 mmol) was added dropwise [slow addition is crucial]. After 30 min at 0 °C, pent-3-yn-1-ylbenzene (120 mg, 0.83 mmol) was dissolved in degassed THF (2 mL) and added and then the reaction was heated to 50°C for 1 h. The resulting mixture was cooled to 0°C and *N*-bromosuccinimide (326 mg, 1.83 mmol) was added. After 10 min the reaction was quenched with saturated aqueous $NaHCO_3$ and then filtered through a plug of silica gel. The silica gel was rinsed with ethyl acetate and the layers were separated. The combined organic extracts were washed with brine and then dried over anhydrous magnesium sulfate, filtered and concentrated under reduced pressure. Purification of the residue *via* column chromatography eluting with heptane afforded the title compound **23** as a colourless oil (130 mg, 70 %, >98:2 *E/Z*, 16:1 mixture of regioisomers). A small quantity (c.a. 100 mg) of regioisomerically pure **23** was obtained by repeated column chromatography eluting with pentane. The *E/Z* selectivity was confirmed by nOe (see NMR spectra).

^{1}H NMR (500 MHz, $CDCl_3$) δ_H = 7.34 – 7.29 (m, 2H), 7.26 – 7.18 (m, 3H), 5.91 (tq, J = 7.6, 1.4 Hz, 1H), 2.71 (t, J = 7.7 Hz, 2H), 2.35 (q, J = 7.6 Hz, 2H), 2.15 (d, J = 1.0 Hz, 3H);

^{13}C NMR (121 MHz, $CDCl_3$) δ_C = 141.1, 131.1, 128.4, 128.4, 126.1, 120.1, 35.2, 31.5, 23.1;

FTIR (neat) ν/cm^{-1} = 3027, 2922, 1651, 1496, 1454, 1379, 1092, 1063, 747;

HRMS (EI^+): calculated for $C_{11}H_{13}$ = 145.1017, mass found = 145.1017. [*N.B. mass corresponds to loss of Br*].

(*Z*)-3-Bromobut-2-en-1-ol, 32

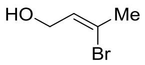

32

By analogy to literature procedure,[7,8] a solution of Red-Al (60 % w/w in toluene, 3.8 mL, 11.4 mmol) was added dropwise to a stirred solution of 2-butyn-1-ol (0.54 mmol, 7.1 mmol) in anhydrous Et_2O (12 mL) at 0 °C. The resulting mixture was warmed to room temperature and stirred for 4 h. After this time, the reaction was cooled to 0 °C and *N*-bromosuccinimide (3.8 g, 21.4 mmol) was added in several portions and then the resulting mixture was warmed to room temperature and stirred overnight. After this time, the solution was cooled to 0 °C and saturated aqueous sodium thiosulfate was added. The organic layer was separated and the aqueous layer was extracted three times with ethyl acetate. The combined organic extracts were dried over anhydrous magnesium sulfate, filtered and concentrated under reduced pressure. Purification *via* column chromatography eluting with 10:90 ethyl acetate/pentane afforded the title compound **32** as a colourless oil (700 mg, 65 %, >98:2 *Z*/*E*).

^{1}H NMR (500 MHz, $CDCl_3$) δ_H = 5.92 – 5.85 (m, 1H), 4.24 – 4.17 (m, 2H), 2.34 – 2.27 (m, 3H), 2.08 (bs, 1H);

^{13}C NMR (121 MHz, $CDCl_3$) δ_C = 128.1, 124.3, 62.4, 28.8;

FTIR (neat) ν/cm^{-1} = 3307, 2918, 1661, 1428, 1103, 1081, 1010;

LRMS (EI$^+$): calculated for C_4H_7BrO = 150, mass found = 150.

(*Z*)-((3-Bromobut-2-en-1-yl)oxy)(tert-butyl)diphenylsilane, 25

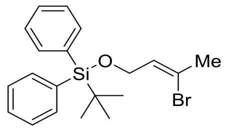

25

Imidazole (135 mg, 1.98 mmol) and *tert*-butyldimethylchlorosilane (0.53 mL, 1.09 mmol) were added sequentially to a stirred solution of **32** (250 mg, 1.65 mmol) in dichloromethane (6 mL) at room temperature. The resulting mixture was stirred at room temperature for 4 hours and then poured into water. The aqueous phase was extracted with CH_2Cl_2 and the combined organic extracts were then washed with brine, dried over anhydrous magnesium sulfate, filtered and then concentrated under reduced pressure. Purification *via* column chromatography eluting with pentane gave the title compound **25** as a colourless oil (500 mg, 78 %, >98:2 *Z*/*E*). The *Z*/*E* selectivity was confirmed by nOe (see NMR spectra).

^{1}H NMR (400 MHz, $CDCl_3$) δ_H = 7.72 – 7.60 (m, 4H), 7.48 – 7.29 (m, 6H), 5.92 – 5.84 (m, 1H), 4.35 – 4.26 (m, 2H), 2.30 – 2.15 (m, 3H), 1.06 (s, 9H);

^{13}C NMR (101 MHz, $CDCl_3$) δ_C = 135.6, 133.7, 129.7, 129.1, 127.8, 121.9, 64.4, 28.8, 26.9, 19.3;

FTIR (neat) ν/cm^{-1} = 3071, 2957, 2930, 2857, 1662, 1462, 1427, 1361, 1105, 1053, 823, 738;

HRMS (ESI$^+$): calculated for $C_{20}H_{25}BrNaOSi$ = 411.0750, mass found = 411.0749.

(*E*)-4,4,5,5-Tetramethyl-2-(4-phenylbut-1-en-1-yl)-1,3,2-dioxaborolane, 16

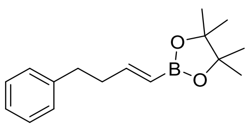

16

4-Phenyl-1-butyne (1.83 g, 14.1 mmol) was stirred neat in a room temperature water bath and freshly distilled catecholborane (1.65 mL, 15.5 mmol) was added dropwise. The resulting solution was heated

to 70 °C for 2 hours and then cooled to room temperature and diluted with THF (20 mL). Pinacol (2.00 g, 16.9 mmol) was added and the resulting solution was stirred at room temperature for 2 hours. After this time, the reaction mixture was diluted with diethyl ether and water. The organic layer was separated and the aqueous layer was extracted twice with diethyl ether. The combined organic extracts were washed with brine and then dried over anhydrous magnesium sulfate, filtered and concentrated under reduced pressure. Purification of the residue *via* column chromatography eluting with 98:2 hexane/ethyl acetate afforded the title compound **16** as a colourless oil (1.53 g, 42 %, >98:2 *E*/*Z*). The spectral data matched that previously reported in the literature.[9]

^{1}H NMR (400 MHz, $CDCl_3$) δ_H = 7.30 – 7.23 (m, 2H), 7.20 – 7.14 (m, 3H), 6.70 (dt, J = 17.9, 6.2 Hz, 1H), 5.50 (dt, J = 17.9, 1.6 Hz, 1H), 2.77 – 2.70 (m, 2H), 2.51 – 2.43 (m, 2H), 1.26 (s, 12H);

^{13}C NMR (101 MHz, $CDCl_3$) δ_C = 153.5, 141.9, 128.4, 128.4, 125.9, 83.2, 37.6, 34.7, 24.9. [*N.B. The carbon attached to boron was not observed due to quadrupolar relaxation*];

^{11}B NMR (96 MHz, $CDCl_3$) δ_B = 29.0;

FTIR (neat) ν/cm^{-1} = 2978, 1637, 1397, 1359, 1319, 1143, 1002, 972, 849;

LRMS (EI$^+$): calculated for $C_{16}H_{23}BO_2$ = 258, mass found = 258.

(*R*)-2-(1-(4-Methoxyphenyl)-7-methyloct-6-en-3-yl)-4,4,5,5-tetramethyl-1,3,2-dioxaborolane, 6

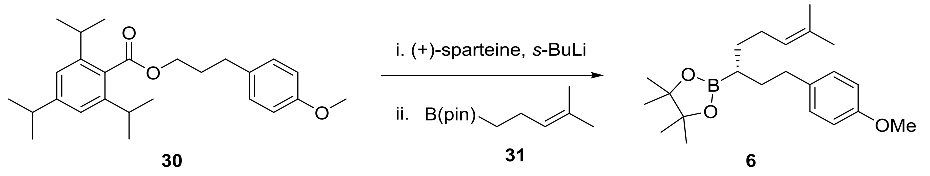

A stirred solution of **30**[10] (3.70 g, 9.32 mmol) and (+)-sparteine (2.17 g, 9.25 mmol) in diethyl ether (40 mL) was cooled to -78 °C. A solution of *sec*-butyllithium (1.3 M in hexanes, 6.59 mL, 8.57 mmol) was added dropwise. The resulting mixture was stirred at -78 °C for 5 hours and then a solution of **31**[11] (1.50 g, 7.13 mmol) in diethyl ether (12 mL) was added dropwise. The resulting solution was stirred at

-78 °C for 1 hours and then allowed to warm to room temperature. The mixture was then heated to reflux for 20 hours and then cooled to room temperature and diluted with diethyl ether. The resulting solution was washed three times with aqueous 3 M HCl followed by brine and then dried over anhydrous magnesium sulfate, filtered and concentrated under reduced pressure. Purification of the residue *via* column chromatography eluting with a gradient from 60:40 toluene/pentane to 100:0 toluene/pentane afforded the title compound **6** as a colourless oil (530 mg, 21 %, 93.0:7.0 e.r.).

^{1}H NMR (400 MHz, $CDCl_3$) δ_H = 7.08 (d, J = 8.7 Hz, 2H), 6.80 (d, J = 8.7 Hz, 2H), 5.11 (tsept, J = 7.1, 1.4 Hz, 1H), 3.77 (s, 3H), 2.60 – 2.46 (m, 2H), 2.05 – 1.88 (m, 2H), 1.76 – 1.55 (m, 8H), 1.54 – 1.32 (m, 2H), 1.25 (s, 12H), 1.11 – 0.99 (m, 1H);

^{13}C NMR (101 MHz, $CDCl_3$) δ_C = 157.7, 135.3, 131.3, 129.3, 125.0, 113.7, 83.0, 55.3, 34.8, 33.8, 31.5, 27.8, 25.8, 24.9, 17.7. [*N.B. The carbon attached to boron was not observed due to quadrupolar relaxation*];

^{11}B NMR (128 MHz, $CDCl_3$) δ_B = 33.9;

FTIR (neat) ν/cm^{-1} = 2977, 2922, 2855, 1612, 1512, 1380, 1314, 1244, 1143, 1039, 823;

HRMS (EI$^+$): calculated for $C_{22}H_{35}BO_3$ = 358.2679, mass found = 358.2669.

$[\alpha]_D^{20}$ = -3 (c = 1.00, $CHCl_3$).

Determination of e.r.

A stirred solution of **6** (10 mg, 0.028 mmol) in THF (0.5 mL) was cooled to 0 °C and 2M aqueous sodium hydroxide (0.5 mL) and 30 % aqueous hydrogen peroxide (0.25 mL) were sequentially added. The resulting rapidly stirred mixture was warmed to room temperature for 2 hours and then directly purified by preparative TLC (10:90 diethyl ether/hexane) to afford an analytical quantity of the chiral alcohol which was analysed by chiral HPLC (93.0:7.0 e.r.).

Chiral HPLC (Chiralpak IB with guard, 95 % IPA, 5 % hexane, 0.7 mL/min, 25 °C, λ = 210.8 nm, 5 μL injection):

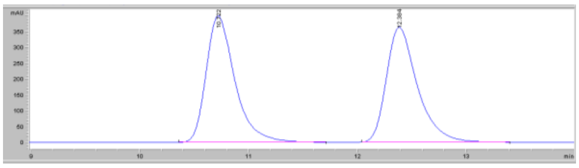

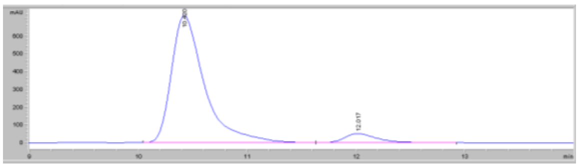

RetTime [min]	Type	Width [min]	Area [mAU*s]	Height [mAU]	Area %
10.474	BP	0.2784	8061.14844	438.69055	50.1742
11.943	VB	0.2954	8005.16406	403.62411	49.8258

RetTime [min]	Type	Width [min]	Area [mAU*s]	Height [mAU]	Area %
10.420	BV	0.3163	1.50642e4	719.45251	93.0454
12.017	VB	0.3258	1125.96497	51.75937	6.9546

(5*R*,6*S*)-5-((*S*)-but-3-en-2-yl)-2,2,3,3,6,10,10-heptamethyl-9,9-diphenyl-4,8-dioxa-3,9-disilaundecane, 34

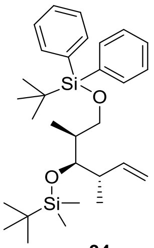

34

A solution of (2S,3R,4S)-1-((tert-butyldiphenylsilyl)oxy)-2,4-dimethylhex-5-en-3-ol, **33**[12] (267 mg, 0.269 mmol) in CH_2Cl_2 (8 mL) was stirred at 0°C and 2,6-lutidine (0.25 mL, 1.06 mmol) and TBSOTf (0.16 mL, 1.4 mmol) were added successively. The reaction mixture was warmed to room temperature and stirred for 4 hours. After this time, saturated aqueous $NaHCO_3$ was added and the organic layer was separated and the aqueous layer was extracted twice with dichloromethane. The combined organic extracts were dried over anhydrous magnesium sulfate, filtered and concentrated under reduced pressure. Purification of the residue *via* column chromatography eluting with pentane afforded the title compound **34** as a colourless oil (275 mg, 76 %).

^{1}H NMR (400 MHz, $CDCl_3$) δ_H = 7.71 – 7.59 (m, 4H), 7.48 – 7.31 (m, 6H), 5.74 (ddd, *J* = 17.7, 10.3, 7.6 Hz, 1H), 4.99 – 4.81 (m, 2H), 3.80 (dd, *J* = 9.9, 5.8 Hz, 1H), 3.54 (t, *J* = 5.0 Hz, 1H), 3.48 – 3.31 (m, 1H),

2.41 – 2.24 (m, 1H), 2.06 – 1.88 (m, 1H), 1.06 (s, 9H), 0.96 (d, J = 4.2 Hz, 3H), 0.95 (d, J = 4.3 Hz, 3H), 0.82 (s, 9H), -0.00 (s, 3H), -0.06 (s, 3H).

^{13}C NMR (101 MHz, $CDCl_3$) δ_C = 142.8, 135.7, 135.7, 134.2, 134.1, 129.5, 129.5, 127.6, 113.6, 77.7, 66.2, 41.6, 40.6, 27.0, 26.1, 19.4, 18.4, 15.9, 14.4, -3.7, -4.0.

FTIR (neat) ν/cm^{-1} = 3071, 2957, 2929, 2886, 2857, 1472, 1462, 1428, 1253, 1111, 1080, 1031, 1005, 911, 835, 773, 738.

HRMS (ESI$^+$): calculated for $C_{30}H_{48}NaO_2Si_2^+$ = 519.3085, mass found = 519.3094;

$[\alpha]_D^{25}$ = -5.77 (c = 0.87, $CHCl_3$).

(5*R*,6*S*)-2,2,3,3,6,10,10-heptamethyl-9,9-diphenyl-5-((*S*,*Z*)-4-(4,4,5,5-tetramethyl-1,3,2-dioxaborolan-2-yl)pent-3-en-2-yl)-4,8-dioxa-3,9-disilaundecane, 27

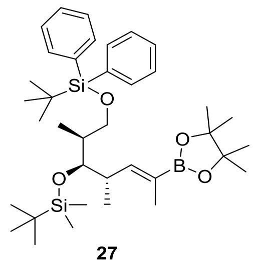

27

A stirred solution alkene **34** (314 mg, 0.63 mmol) and isopropenyl boronic acid pinacol ester (0.6 mL, 3.16 mmol) in degassed CH_2Cl_2 (0.3 mL) was stirred at room temperature. A solution of 2,6-dichlorobenzoquinone (12 mg, 0.06 mmol) in degassed dichloromethane (0.3 mL) was then added. A solution of Hoveyda-Grubbs 2nd generation catalyst (0.2 M in degassed dichloromethane, 0.16 mL, 0.032 mmol) was added. The resulting solution was heated to 40 °C under nitrogen for 1 hour and then the solution was freeze-pump-thawed (liquid nitrogen) to remove solvated ethylene. A further portion of HG-II catalyst solution (0.2 M in degassed dichloromethane, 0.08 mL, 0.016 mmol) was added and the reaction mixture was heated to 40 ^{o}C for 1 hour. The mixture was freeze-pump-thawed (liquid nitrogen) and then a final portion of HG-II catalyst solution (0.2 M in degassed

dichloromethane, 0.08 mL, 0.016 mmol) was added. The reaction mixture was then heated to 40°C for 16 hours and was then diluted with a mixture of hexanes:Et_2O (10:1) and then passed through a short plug of silica gel, washing with diethyl ether. The filtrate was concentrated under reduced pressure (*c.a.* 0.2 mmHg) to remove excess isopropenyl boronic acid pinacol ester. Purification of the residue *via* column chromatography eluting with 1:1 toluene/pentane) afforded the title compound **27** as a colourless oil (310 mg, 77%). The *Z*/*E* selectivity was confirmed by nOe (see NMR spectra).

^{1}H NMR (400 MHz, $CDCl_3$) δ_H = 7.68 – 7.60 (m, 4H), 7.43 – 7.31 (m, 6H), 6.29 – 6.08 (m, 1H), 3.78 (dd, *J* = 9.9, 5.1 Hz, 1H), 3.50 (t, *J* = 5.4 Hz, 1H), 3.41 (t, *J* = 9.2 Hz, 1H), 2.78 – 2.64 (m, 1H), 1.99 – 1.84 (m, 1H), 1.65 (s, 3H), 1.24 (s, 6H), 1.22 (s, 6H), 1.05 (s, 9H), 0.96 (d, *J* = 6.9 Hz, 3H), 0.91 (d, *J* = 6.7 Hz, 3H), 0.81 (s, 9H), -0.02 (s, 3H), -0.11 (s, 3H);

^{13}C NMR (101 MHz, $CDCl_3$) δ_C = 150.6, 135.7, 134.2, 134.2, 129.5, 127.6, 83.1, 77.5, 66.3, 41.4, 36.0, 27.1, 26.2, 25.1, 24.7, 19.4, 18.4, 15.7, 14.2, 14.0, -3.8, -3.9. [*N.B. The carbon attached to boron was not observed due to quadrupolar relaxation*];

^{11}B NMR (96 MHz, $CDCl_3$) δ_B = 28.7;

FTIR (neat) ν/cm^{-1} = 2958, 2930, 2857, 1472, 1428, 1411, 1370, 1339, 1303, 1253, 1143, 1111, 1079, 1024, 964, 860, 774, 738;

HRMS (ESI$^+$): calculated for $C_{37}H_{61}BNaO_4Si_2^+$ = 659.4101, mass found = 659.4108;

$[\alpha]_D^{25}$ = -4.6 (*c* = 0.8, $CHCl_3$).

1.3.2 Stereodivergent Coupling

(*R*,*Z*)-1-Methoxy-4-(3-methyl-7-phenylhept-4-en-1-yl)benzene, 3a

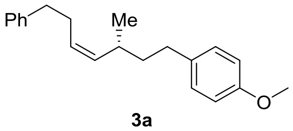

3a

According to **General Procedure A** with **1** (45 mg, 0.21 mmol) and **2**[13] (59 mg, 0.20 mmol, 95.5:4.5 e.r.). The *Z*/*E* ratio of the product was determined to be >98:2 by ^{1}H NMR analysis of the crude reaction mixture. Purification *via* column chromatography eluting with a gradient from hexane to 1:99 diethyl ether/hexane afforded the title compound **3a** as a colourless oil (48 mg, 80 %, >98:2 *Z*/*E*, 95.5:4.5 *e.r.*).

^{1}H NMR (400 MHz, $CDCl_3$) δ_H = 7.30-7.24 (m, 2H), 7.19-7.14 (m, 3H), 7.06 (d, J = 8.7 Hz, 2H), 6.80 (d, J = 8.7 Hz, 2H), 5.39 (dtd, J = 10.9, 7.3, 0.9 Hz, 1H), 5.19 (ddt, J = 11.1, 9.8, 1.5 Hz, 1H), 3.77 (s, 3H), 2.65 (t, J = 7.7 Hz, 2H), 2.55-2.28 (m, 5H), 1.57 (dddd, J = 13.3, 10.1, 6.4, 5.3 Hz, 1H), 1.43 (dddd, J = 13.3, 10.0, 8.5, 5.7 Hz, 1H), 0.89 (d, J = 6.6 Hz, 3H);

^{13}C NMR (101 MHz, $CDCl_3$) δ_C = 157.7, 142.2, 136.8, 135.0, 129.3, 128.6, 128.3, 127.8, 125.9, 113.8, 55.3, 39.6, 36.3, 32.9, 31.5, 29.6, 21.3;

FTIR (neat) ν/cm^{-1} = 2952, 2924, 1612, 1511, 1454, 1244, 1176, 1037, 825, 734, 698;

HRMS (EI^{+}): calculated for $C_{21}H_{26}O$ = 294.1984, mass found = 294.1979;

$[\alpha]_D^{25}$ = +44 (*c* = 1.00, $CHCl_3$);

Chiral HPLC (Chiralpak IB with guard, 0.2 % IPA, 99.8 % hexane, 1.0 mL/min, 25 °C, λ = 273.1 nm, 5 μL injection): *N.B. in enantioenriched spectrum, peak at 11.2 min corresponds to E-isomer (c.a. 0.6 %).*

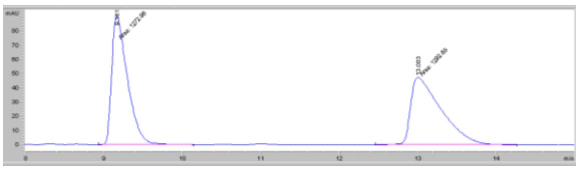

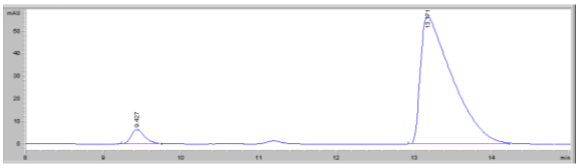

RetTime [min]	Type	Width [min]	Area [mAU*s]	Height [mAU]	Area %
9.161	MM	0.2338	1272.95911	90.74634	49.6125
13.003	MM	0.4527	1292.84583	47.59566	50.3875

RetTime [min]	Type	Width [min]	Area [mAU*s]	Height [mAU]	Area %
9.427	BB	0.1792	76.42831	6.40486	4.5405
13.171	BB	0.4042	1606.82983	56.78058	95.4595

Alternative preparation of **3a**

According to **General Procedure C** with **1** (45 mg, 0.21 mmol) and **2**[13] (59 mg, 0.20 mmol, 95.5:4.5 e.r.). The *Z*/*E* ratio of the product was determined to be >98:2 by ^{1}H NMR analysis of the crude reaction mixture. Purification *via* column chromatography eluting with a gradient from hexane to 1:99 diethyl ether/hexane afforded the title compound **3a** as a colourless oil (48 mg, 80 %, >98:2 *Z*/*E*, 95.5:4.5 *e.r.*). The spectral data for this material was identical to that described above.

Chiral HPLC (Chiralpak IB with guard, 0.2 % IPA, 99.8 % hexane, 1.0 mL/min, 25 °C, λ = 273.1 nm, 5 μL injection): *N.B. in enantioenriched spectrum, peak at 11.2 min corresponds to E-isomer (c.a. 0.6 %).*

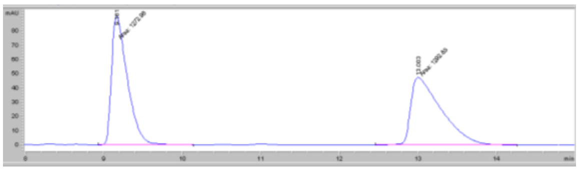

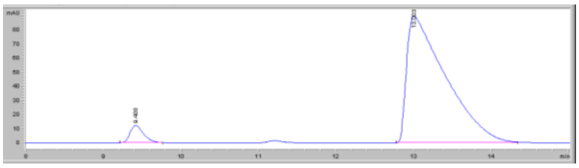

RetTime [min]	Type	Width [min]	Area [mAU*s]	Height [mAU]	Area %
9.161	MM	0.2338	1272.95911	90.74634	49.6125
13.003	MM	0.4527	1292.84583	47.59566	50.3875

RetTime [min]	Type	Width [min]	Area [mAU*s]	Height [mAU]	Area %
9.408	BB	0.1839	150.20271	12.34832	4.5480
13.003	BB	0.4877	3152.39185	90.20178	95.4520

(*R*,*E*)-1-Methoxy-4-(3-methyl-7-phenylhept-4-en-1-yl)benzene, 3b

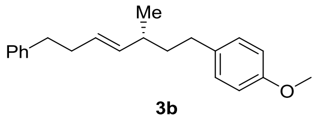

3b

According to **General Procedure B** with **1** (34 mg, 0.16 mmol) and **2**[13] (45 mg, 0.16 mmol, 95.5:4.5 e.r.). The *E*/*Z* ratio of the product was determined to be >98:2 by ^{1}H NMR analysis of the crude reaction mixture. Purification *via* column chromatography eluting with a gradient from hexane to 1:99 diethyl ether/hexane afforded the title compound **3b** as a colourless oil (34 mg, 74 %, >98:2 *E*/*Z*, 95.5:4.5 e.r.).

^{1}H NMR (400 MHz, $CDCl_3$) δ_H = 7.32 – 7.26 (m, 2H), 7.22 – 7.16 (m, 3H), 7.07 (d, J = 8.7 Hz, 2H), 6.83 (d, J = 8.7 Hz, 2H), 5.44 (dtd, J = 15.2, 6.5, 0.8 Hz, 1H), 5.32 (ddt, J = 15.3, 7.7, 1.2 Hz, 1H), 3.79 (s, 3H), 2.70 (t, J = 7.7 Hz, 2H), 2.52 (ddd, J = 13.7, 9.2, 6.5 Hz, 1H), 2.46 (ddd, J = 13.9, 9.3, 7.0 Hz, 1H), 2.35 (q, J = 7.3 Hz, 2H), 2.10 (sept, J = 6.9 Hz, 1H), 1.61 – 1.46 (m, 2H), 0.99 (d, J = 6.7 Hz, 3H);

^{13}C NMR (101 MHz, $CDCl_3$) δ_C = 157.7, 142.2, 136.9, 135.1, 129.4, 128.6, 128.3, 128.2, 125.8, 113.8, 55.3, 39.2, 36.4, 36.3, 34.5, 32.8, 21.1;

FTIR (neat) ν/cm^{-1} = 2924, 1611, 1511, 1454, 1243, 1176, 1037, 969, 822, 745, 698;

HRMS (EI$^+$): calculated for $C_{21}H_{26}O$ = 294.1984, mass found = 294.1978;

$[\alpha]_D^{25}$ = –16 (*c* = 1.00, $CHCl_3$);

Chiral HPLC (Chiralpak IB with guard, 0.2 % IPA, 99.8 % hexane, 1.0 mL/min, 25 °C, λ = 273.1 nm, 5 μL injection):

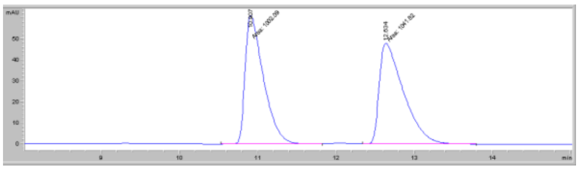

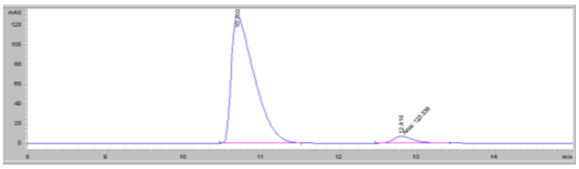

RetTime [min]	Type	Width [min]	Area [mAU*s]	Height [mAU]	Area %
10.907	MM	0.2716	1002.09204	61.49525	49.0329
12.634	MM	0.3608	1041.62317	48.11095	50.9671

RetTime [min]	Type	Width [min]	Area [mAU*s]	Height [mAU]	Area %
10.702	BB	0.3066	2646.86206	129.43910	95.5478
12.810	MM	0.2971	123.33555	6.91868	4.4522

tert-Butyl (_S_,_Z_)-4-phenethyl-8-phenyloct-5-enoate, 5a

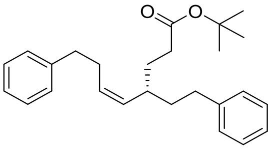

5a

A stirred solution of **1** (60 mg, 0.28 mmol) in THF (1.03 mL) was cooled to -78 °C and a solution of *tert*-butyllithium (1.7 M in pentane, 0.33 mL, 0.57 mmol) was added dropwise. The resulting solution was stirred at -78 °C for 30 minutes to form a 0.2 M solution of the corresponding vinyl lithium. In a separate Schlenk tube, a solution of **4**[13] (30 mg, 0.080 mmol, 95.5:4.5 e.r.) in THF (0.40 mL) was cooled to -78 °C and the vinyl lithium solution described above (0.44 mL, 0.088 mmol) was added in a single portion by syringe. The resulting solution was stirred at -78 °C for 30 minutes and then warmed to 0 °C and stirred for 2 minutes. After this time, a suspension of sodium methoxide (3.0 M in MeOH, 0.08 mL, 0.24 mmol) was added in a single portion followed by dropwise addition of a solution of iodine (0.5 M in MeOH, 0.19 mL, 0.096 mmol). The resulting mixture was stirred at 0 °C for 30 minutes and then saturated aqueous sodium sulfite was added followed by water and dichloromethane. The organic layer was separated and the aqueous layer was extracted twice with dichloromethane. The combined organic extracts were dried over anhydrous magnesium sulfate, filtered and concentrated under reduced pressure. The *Z*/*E* ratio of the product was determined to be >98:2 by ^{1}H NMR analysis of the crude reaction mixture. Purification of the residue *via* column chromatography eluting with a gradient

from pentane to 95:5 pentane/diethyl ether afforded the title compound **5a** as a colourless oil (25 mg, 82 %, >98:2 *Z*/*E*, 95.5:4.5 e.r.).

^{1}H NMR (400 MHz, $CDCl_3$) δ_H = 7.29 – 7.22 (m, 4H), 7.19 – 7.11 (m, 6H), 5.52 (dtd, *J* = 10.9, 7.2, 0.9 Hz, 1H), 5.09 (tt, *J* = 10.6, 1.6 Hz, 1H), 2.66 (td, *J* = 7.9, 2.6 Hz, 2H), 2.58 (ddd, *J* = 13.7, 10.4, 5.2 Hz, 1H), 2.44 (ddd, *J* = 13.8, 10.4, 6.3 Hz, 1H), 2.38 – 2.25 (m, 3H), 2.16 (ddd, *J* = 15.9, 9.2, 5.8 Hz, 1H), 2.06 (ddd, *J* = 15.9, 9.1, 6.9 Hz, 1H), 1.76 – 1.64 (m, 2H), 1.50 – 1.34 (m, 2H), 1.41 (s, 9H);

^{13}C NMR (101 MHz, $CDCl_3$) δ_C = 173.3, 142.7, 142.0, 134.2, 130.3, 128.6, 128.4, 128.4, 128.3, 125.9, 125.7, 80.0, 37.6, 36.7, 36.1, 33.7, 33.4, 30.8, 29.8, 28.2;

FTIR (neat) ν/cm^{-1} = 2976, 2927, 2862, 1127, 1608, 1496, 1454, 1366, 1255, 1145, 737, 698;

HRMS (ESI$^+$): calculated for $C_{26}H_{34}NaO_2^+$ = 401.2451, mass found = 401.2454;

$[\alpha]_D^{25}$ = +13 (*c* = 0.90, $CHCl_3$);

Chiral HPLC (Chiralpak IB with guard, 0.2 % IPA, 99.8 % hexane, 1.0 mL/min, 25 °C, λ = 210.8 nm, 5 µL injection):

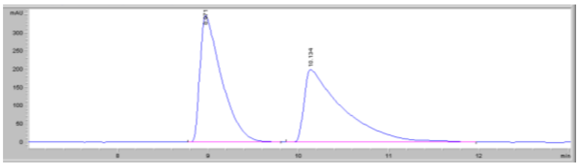

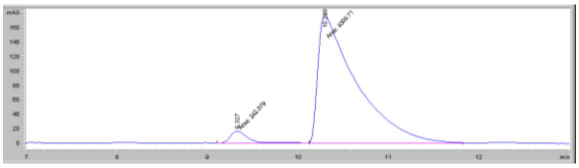

RetTime [min]	Type	Width [min]	Area [mAU*s]	Height [mAU]	Area %
8.971	PB	0.2440	5774.73633	347.22952	49.3790
10.134	BB	0.3980	5919.97510	199.62711	50.6210

RetTime [min]	Type	Width [min]	Area [mAU*s]	Height [mAU]	Area %
9.327	MF	0.2410	242.07895	16.74062	4.3635
10.298	FM	0.4976	5305.70508	177.70953	95.6365

tert-Butyl (_S_,_E_)-4-phenethyl-8-phenyloct-5-enoate, 5b

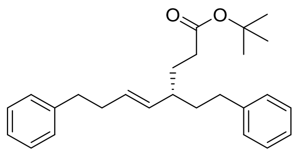

5b

A stirred solution of **1** (60 mg, 0.28 mmol) in THF (1.03 mL) was cooled to -78 °C and a solution of _tert_-butyllithium (1.7 M in pentane, 0.33 mL, 0.57 mmol) was added dropwise. The resulting solution was stirred at -78 °C for 30 minutes to form a 0.2 M solution of the corresponding vinyl lithium. In a separate Schlenk tube, a solution of **4**[13] (30 mg, 0.080 mmol, 95.5:4.5 e.r.) in THF (0.40 mL) was cooled to -78 °C and the vinyl lithium solution described above (0.44 mL, 0.088 mmol) was added in a single portion by syringe. The resulting solution was stirred at -78 °C for 30 minutes and then a solution of PhSeCl (1.0 M in THF, 0.10 mL, 0.10 mmol) was added dropwise. The resulting solution was stirred at -78 °C for 30 minutes and then warmed to room temperature and stirred for a further 15 minutes and then filtered through a short plug of silica gel washing with diethyl ether. The filtrate was concentrated under reduced pressure and the residue was dissolved in THF (0.80 mL). The resulting stirred solution was cooled to -78 °C and a solution of _m_CPBA (0.2 M in THF, 0.80 mL, 0.16 mmol) was added dropwise. The resulting solution was warmed to -45 °C (acetonitrile/dry ice bath) and stirred for 30 minutes. After this time, dimethylsulfide (0.11 mL, 1.6 mmol) was added and the mixture was allowed to warm to room temperature and then filtered through a short plug of silica gel washing with diethyl ether. The filtrate was concentrated under reduced pressure. The _E_/_Z_ ratio of the product was determined to be >98:2 by ^{1}H NMR analysis of the crude reaction mixture. Purification of the residue _via_ column chromatography eluting with a gradient from pentane to 95:5 pentane/diethyl ether afforded the title compound **5b** as a colourless oil (25 mg, 82 %, >98:2 _E_/_Z_).

^{1}H NMR (400 MHz, $CDCl_3$) δ_H = 7.30 – 7.23 (m, 4H), 7.21 – 7.10 (m, 6H), 5.42 (dt, _J_ = 15.3, 6.8 Hz, 1H), 5.11 (ddt, _J_ = 15.2, 9.0, 1.4 Hz, 1H), 2.71 (t, _J_ = 7.6 Hz, 2H), 2.57 (ddd, _J_ = 13.8, 10.1, 5.3 Hz, 1H), 2.45

(ddd, *J* = 13.9, 10.0, 6.7 Hz, 1H), 2.40 – 2.32 (m, 2H), 2.15 (ddd, *J* = 15.3, 9.4, 5.8 Hz, 1H), 2.07 (ddd, *J* = 15.8, 9.2, 6.8 Hz, 1H), 1.97 – 1.86 (m, 1H), 1.72 – 1.59 (m, 2H), 1.55 – 1.38 (m, 2H), 1.42 (s, 9H);

^{13}C NMR (101 MHz, $CDCl_3$) δ_C = 173.4, 142.8, 142.0, 134.3, 131.1, 128.6, 128.5, 128.4, 128.3, 125.8, 125.7, 80.0, 42.2, 37.3, 36.1, 34.4, 33.6, 33.5, 30.6, 28.2;

FTIR (neat) ν/cm^{-1} = 3025, 2976, 2926, 2858, 1727, 1496, 1454, 1366, 1255, 1145, 978, 746, 698;

HRMS (ESI$^+$): calculated for $C_{26}H_{34}NaO_2^+$ = 401.2451, mass found = 401.2453;

$[\alpha]_D^{25}$ = +3 (*c* = 0.71, $CHCl_3$).

(*R*,*Z*)-1-Methoxy-4-(7-methyl-3-(4-phenylbut-1-en-1-yl)oct-6-en-1-yl)benzene, 7a

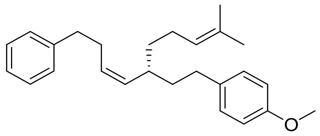

7a

According to **General Procedure A** with **1** (38 mg, 0.18 mmol) and **6** (62 mg, 0.17 mmol, 93.0:7.0 e.r.). The *Z*/*E* ratio of the product was determined to be >98:2 by ^{1}H NMR analysis of the crude reaction mixture. Purification *via* column chromatography eluting with 1:199 diethyl ether/hexane afforded the title compound **7a** as a colourless oil (45 mg, 72 %, >98:2 *Z*/*E*, 93.1:6.9 e.r.).

^{1}H NMR (400 MHz, $CDCl_3$) δ_H = 7.31-7.24 (m, 2H), 7.21-7.14 (m, 3H), 7.05 (d, J = 8.6 Hz, 2H), 6.80 (d, J = 8.6 Hz, 2H), 5.49 (dtd, J = 11.0, 7.3, 0.6 Hz, 1H), 5.13 (ddt, J = 11.4, 10.2, 1.6 Hz, 1H), 5.10-5.04 (m, 1H), 3.77 (s, 3H), 2.65 (t, J = 7.9 Hz, 2H), 2.52 (ddd, J = 13.7, 10.5, 5.3 Hz, 1H), 2.43-2.26 (m, 4H), 2.01-1.78 (m, 2H), 1.67 (d, J = 1.4 Hz, 3H), 1.66-1.59 (m, 1H), 1.57 (d, J = 1.4 Hz, 3H), 1.45-1.34 (m, 2H), 1.23-1.12 (m, 1H);

^{13}C NMR (101 MHz, $CDCl_3$) δ_C = 157.7, 142.2, 135.2, 135.1, 131.4, 129.3, 129.3, 128.5, 128.4, 125.9, 124.9, 113.8, 55.3, 38.0, 36.9, 36.3, 36.0, 32.9, 29.9, 25.9, 25.8, 17.8;

FTIR (neat) ν/cm^{-1} = 2915, 2853, 1612, 1511, 1454, 1244, 1176, 1039, 820, 733, 698;

HRMS (EI$^+$): calculated for $C_{26}H_{34}O$ = 362.2610, mass found = 362.2608;

$[\alpha]_D^{25}$ = +9 (*c* = 1.00, $CHCl_3$);

Chiral HPLC (Chiralpak IB with guard, 0.5 % IPA, 99.5 % hexane, 1.0 mL/min, 25 °C, λ = 210.8 nm, 5 µL injection):

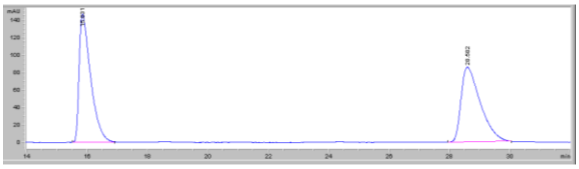

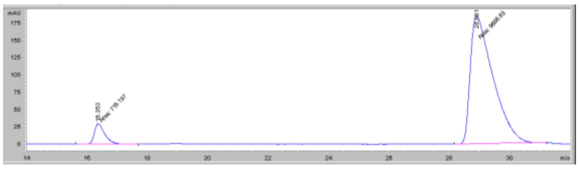

RetTime [min]	Type	Width [min]	Area [mAU*s]	Height [mAU]	Area %
15.831	BB	0.3849	3884.27539	146.69380	50.4859
28.582	BB	0.6327	3809.50854	85.68136	49.5141

RetTime [min]	Type	Width [min]	Area [mAU*s]	Height [mAU]	Area %
16.353	MM	0.4035	716.15735	29.57770	6.9042
28.881	MM	0.8718	9656.63477	184.60527	93.0958

(*R*,*E*)-1-Methoxy-4-(7-methyl-3-(4-phenylbut-1-en-1-yl)oct-6-en-1-yl)benzene, 7b

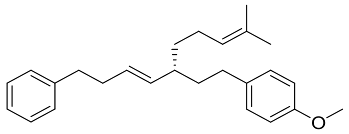

7b

According to **General Procedure B** with **1** (38 mg, 0.18 mmol) and **6** (62 mg, 0.17 mmol, 93.3:6.7 e.r.). The *E*/*Z* ratio of the product was determined to be >98:2 by ^{1}H NMR analysis of the crude reaction mixture. Purification *via* column chromatography eluting with a gradient from hexane to 1:199 diethyl ether/hexane afforded the title compound **7b** as a colourless oil (42 mg, 67 %, >98:2 *E*/*Z*, 92.9:7.1 e.r.).

^{1}H NMR (400 MHz, $CDCl_3$) δ_H = 7.30-7.24 (m, 2H), 7.22-7.14 (m, 3H), 7.04 (d, J = 8.7 Hz, 2H), 6.81 (d, J = 8.6 Hz, 2H), 5.41 (dtd, J = 15.3, 6.6, 0.7 Hz, 1H), 5.17 (ddt, J = 15.2, 8.9, 1.4 Hz, 1H), 5.10-5.03 (m, 1H), 3.78 (s, 3H), 2.70 (t, J = 7.7 Hz, 2H), 2.51 (ddd, J = 13.8, 10.2, 5.3 Hz, 1H), 2.44-2.32 (m, 3H), 2.00-1.80 (m, 3H), 1.67 (d, J = 1.1 Hz, 3H), 1.65-1.55 (m, 4H), 1.49-1.31 (m, 2H), 1.28-1.17 (m, 1H);

^{13}C NMR (101 MHz, $CDCl_3$) δ_C = 157.6, 142.2, 135.3, 135.2, 131.2, 130.1, 129.3, 128.6, 128.3, 125.8, 124.9, 113.7, 55.3, 42.2, 37.6, 36.3, 35.7, 34.5, 32.7, 25.8, 25.8, 17.8;

FTIR (neat) ν/cm^{-1} = 2914, 1612, 1512, 1454, 1300, 1245, 1176, 1039, 970, 821, 745, 698;

HRMS (EI^+): calculated for $C_{26}H_{34}O$ = 362.2610, mass found = 362.2601;

$[\alpha]_D^{25}$ = –2 (*c* = 1.00, $CHCl_3$);

Chiral HPLC (Chiralpak IB with guard, 0.5 % IPA, 99.5 % hexane, 1.0 mL/min, 25 °C, λ = 210.8 nm, 5 μL injection):

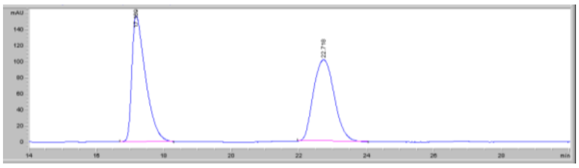

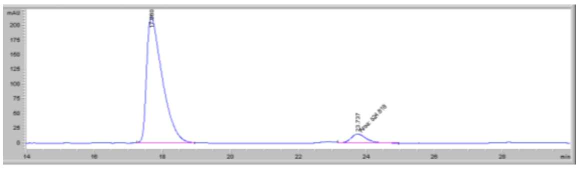

RetTime [min]	Type	Width [min]	Area [mAU*s]	Height [mAU]	Area %
17.169	BB	0.4150	4437.01318	157.35081	50.3843
22.718	BB	0.6997	4369.33105	101.60836	49.6157

RetTime [min]	Type	Width [min]	Area [mAU*s]	Height [mAU]	Area %
17.668	BB	0.4667	6871.77148	216.26610	92.9071
23.737	FM	0.5533	524.61591	15.80225	7.0929

(*S*,*Z*)-Hex-3-ene-1,5-diyldibenzene, 9a

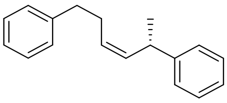

9a

According to **General Procedure A** with **1** (45 mg, 0.21 mmol) and **8**[14] (47 mg, 0.20 mmol, 98.0:2.0 e.r.). The *Z*/*E* ratio of the product was determined to be 96:4 by ^1H NMR analysis of the crude reaction mixture. Purification *via* column chromatography eluting with hexane afforded the title compound **9a** as a colourless oil (39 mg, 81 %, 96:4 *Z*/*E*, 98.0:2.0 e.r.).

^1H NMR (400 MHz, $CDCl_3$) δ_H = 7.31-7.25 (m, 4H), 7.22-7.14 (m, 6H), 5.53 (ddt, J = 10.8, 9.3, 1.4 Hz, 1H), 5.42 (dtd, J = 10.7, 7.2, 0.9 Hz, 1H), 3.71 (dq, J = 9.5, 7.0 Hz, 1H), 2.66 (t, J = 7.7 Hz, 2H), 2.53-2.35 (m, 2H), 1.26 (d, J = 7.0 Hz, 3H);

^{13}C NMR (101 MHz, $CDCl_3$) δ_C = 146.6, 142.0, 135.7, 128.6, 128.5, 128.4, 127.7, 126.9, 125.9, 125.9, 37.3, 36.0, 29.5, 22.2;

FTIR (neat) ν/cm^{-1} = 2962, 2925, 1602, 1494, 1452, 1027, 731;

HRMS (EI^+): calculated for $C_{18}H_{20}$ = 236.1565, mass found = 236.1570;

$[\alpha]_D^{25}$ = +185 (*c* = 1.00, $CHCl_3$);

Chiral HPLC (Chiralpak IB with guard, 100 % hexane, 0.5 mL/min, 25 °C, λ = 210.8 nm, 5 μL injection): *N.B. peaks at 15.2 and 16.3 min correspond to E-isomer (c.a. 4 %).*

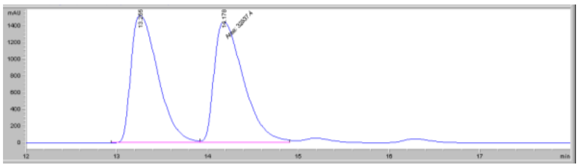

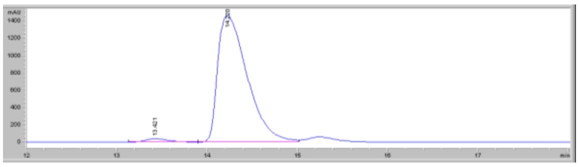

RetTime [min]	Type	Width [min]	Area [mAU*s]	Height [mAU]	Area %
13.255	BV	0.3223	3.15093e4	1517.05420	49.1974
14.178	MF	0.3759	3.25374e4	1442.52527	50.8026

RetTime [min]	Type	Width [min]	Area [mAU*s]	Height [mAU]	Area %
13.421	BV	0.2625	667.46747	39.23516	1.9735
14.220	VV	0.3503	3.31546e4	1463.75903	98.0265

Alternative preparation of **9a**

According to **General Procedure C** with **1** (45 mg, 0.21 mmol), **8**[14] (47 mg, 0.20 mmol, 98.0:2.0 e.r.). The *Z/E* ratio of the product was determined to be >98:2 by ^1H NMR analysis of the crude reaction mixture. Purification *via* column chromatography eluting with hexane afforded the title compound **9a** as a colourless oil (30 mg, 63 %, >98:2 *Z/E*, 98.0:2.0 e.r.). The spectral data for this material was identical to that described above.

Chiral HPLC (Chiralpak IB with guard, 100 % hexane, 0.5 mL/min, 25 °C, λ = 210.8 nm, 5 μL injection):

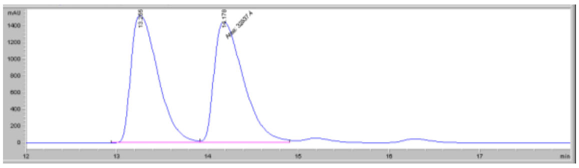

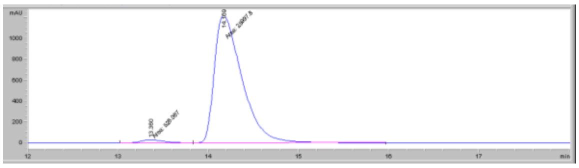

RetTime [min]	Type	Width [min]	Area [mAU*s]	Height [mAU]	Area %
13.255	BV	0.3223	3.15093e4	1517.05420	49.1974
14.178	MF	0.3759	3.25374e4	1442.52527	50.8026

RetTime [min]	Type	Width [min]	Area [mAU*s]	Height [mAU]	Area %
13.360	MF	0.2945	526.06732	29.77309	1.9834
14.169	FM	0.3558	2.59978e4	1217.78723	98.0166

(*S*,*E*)-Hex-3-ene-1,5-diyldibenzene, 9b

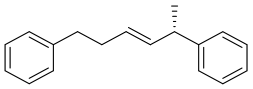

9b

According to **General Procedure B** with **1** (45 mg, 0.21 mmol), **8**[14] (47 mg, 0.20 mmol, 98.0:2.0 e.r.). The *E*/*Z* ratio of the product was determined to be >98:2 by ^{1}H NMR analysis of the crude reaction mixture. Purification *via* column chromatography eluting with hexane afforded the title compound **9b** as a colourless oil (29 mg, 60 %, >98:2 *E*/*Z*, 97.8:2.2 e.r.). The spectral data matched that previously reported in the literature.[15]

^{1}H NMR (400 MHz, $CDCl_3$) δ_H = 7.32-7.25 (m, 4H), 7.22-7.15 (m, 6H), 5.62 (ddt, *J* = 15.3, 6.5, 1.2 Hz, 1H), 5.50 (dtd, *J* = 15.3, 6.5, 1.2 Hz, 1H), 3.42 (qn, *J* = 6.9 Hz, 1H), 2.70 (t, *J* = 7.7 Hz, 2H), 2.39-2.32 (m, 2H), 1.33 (d, *J* = 7.1 Hz, 3H);

^{13}C NMR (101 MHz, $CDCl_3$) δ_C = 146.4, 142.1, 135.8, 128.6, 128.4, 128.3, 128.3, 127.3, 126.0, 125.8, 42.3, 36.1, 34.5, 21.5;

FTIR (neat) ν/cm^{-1} = 3026, 2964, 2927, 1494, 1452, 966, 744, 696;

LRMS (EI^+): calculated for $C_{18}H_{20}$ = 236, mass found = 236;

$[\alpha]_D^{25}$ = –1 (*c* = 1.00, $CHCl_3$);

Chiral HPLC (Chiralpak IB with guard, 100 % hexane, 0.5 mL/min, 25 °C, λ = 210.8 nm, 5 μL injection):

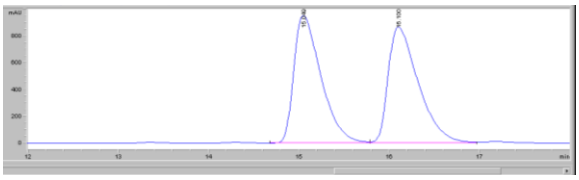

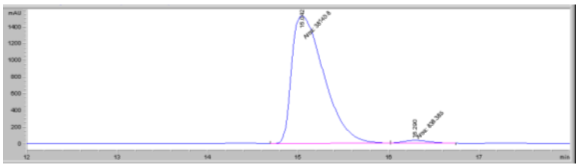

RetTime [min]	Type	Width [min]	Area [mAU*s]	Height [mAU]	Area %
15.049	VV	0.3190	2.00293e4	953.60999	49.7534
16.100	VV	0.3538	2.02279e4	868.33289	50.2466

RetTime [min]	Type	Width [min]	Area [mAU*s]	Height [mAU]	Area %
15.042	MF	0.4147	3.81408e4	1532.87146	97.8491
16.290	FM	0.3634	838.38507	38.44569	2.1509

(*S*,*Z*)-(5-Isopropylhept-3-ene-1,7-diyl)dibenzene, 11a

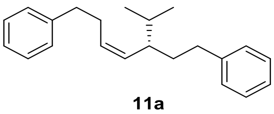

11a

According to **General Procedure A** with **1** (45 mg, 0.21 mmol) and **10**[16] (59 mg, 0.20 mmol, 98.5:1.5 e.r.). The *Z*/*E* ratio of the product was determined to be 94:6 by ^{1}H NMR analysis of the crude reaction mixture. Purification *via* column chromatography eluting with hexane afforded the title compound **11a** as a colourless oil (41 mg, 69 %, 94:6 *Z*/*E*, 98.6:1.4 *e.r.*).

^{1}H NMR (400 MHz, $CDCl_3$) δ_H = 7.31-7.24 (m, 4H), 7.21-7.13 (m, 6H), 5.55 (dt, J = 11.0, 7.2 Hz, 1H), 5.21 (tt, J = 10.8, 1.6 Hz, 1H), 2.71-2.63 (m, 2H), 2.58 (ddd, J = 13.6, 10.8, 4.9 Hz, 1H), 2.42-2.29 (m, 3H), 2.17 (tdd, J = 10.0, 5.7, 3.8 Hz, 1H), 1.75 (dddd, J = 13.2, 10.9, 6.2, 3.7 Hz, 1H), 1.63-1.49 (m, 1H), 1.44 (dtd, J = 13.2, 10.5, 5.0 Hz, 1H), 0.86 (d, J = 6.7 Hz, 3H), 0.82 (d, J = 6.8 Hz, 3H);

^{13}C NMR (101 MHz, $CDCl_3$) δ_C = 143.2, 142.2, 132.9, 130.1, 128.6, 128.4, 128.4, 128.3, 125.9, 125.6, 43.3, 36.2, 34.9, 34.1, 32.4, 30.0, 20.7, 19.1;

FTIR (neat) ν/cm^{-1} = 2954, 1604, 1496, 1453, 746, 732, 697;

HRMS (EI^{+}): calculated for $C_{22}H_{28}$ = 292.2191, mass found = 292.2196;

$[\alpha]_D^{25}$ = +40 (*c* = 1.00, $CHCl_3$);

Chiral HPLC (Chiralpak IB with guard, 100 % hexane, 1.0 mL/min, 25 °C, λ = 210.8 nm, 5 μL injection):

N.B. peak at 8.6 min corresponds to E-isomer (c.a. 6 %).

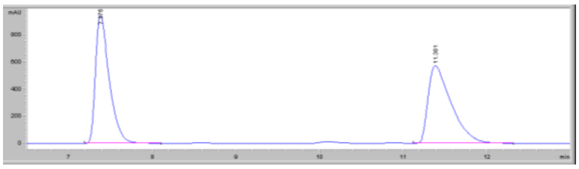

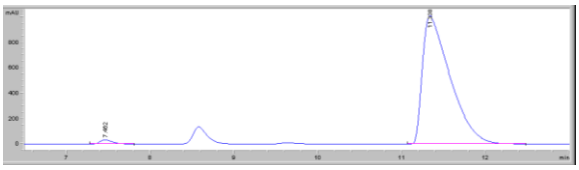

RetTime [min]	Type	Width [min]	Area [mAU*s]	Height [mAU]	Area %
7.375	BB	0.1730	1.07419e4	942.06219	49.5411
11.381	VB	0.2901	1.09409e4	569.70374	50.4589

RetTime [min]	Type	Width [min]	Area [mAU*s]	Height [mAU]	Area %
7.462	BB	0.1515	336.34323	33.40057	1.4362
11.338	VB	0.3425	2.30833e4	1010.54010	98.5638

Alternative preparation of **11a**

According to **General Procedure C** with **1** (45 mg, 0.21 mmol) and **10**[14] (59 mg, 0.20 mmol, 98.5:1.5 e.r.). The *Z*/*E* ratio of the product was determined to be >98:2 by ^{1}H NMR analysis of the crude reaction mixture. Purification *via* column chromatography eluting with hexane afforded an inseparable mixture of the title compound **11a** with diphenyldiselenide. According to a modified literature procedure,[17] the residue was dissolved in dichloromethane (0.6 mL) and hydrogen peroxide (30 % w/w in water, 50 μL, 0.50 mmol) and pyridine (20 μL, 0.25 mmol) were added sequentially. The resulting biphasic mixture was stirred rapidly at room temperature for 2 hours and then directly purified by preparative TLC (hexane) to afford the title compound **11a** as a colourless oil (46 mg, 77 %, >98:2 *Z*/*E*, 98.6:1.4 e.r.). The spectral data for this material was identical to that described above.

Chiral HPLC (Chiralpak IB with guard, 100 % hexane, 1.0 mL/min, 25 °C, λ = 210.8 nm, 5 μL injection):

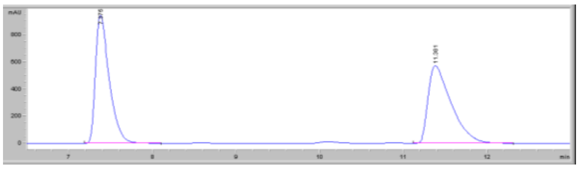

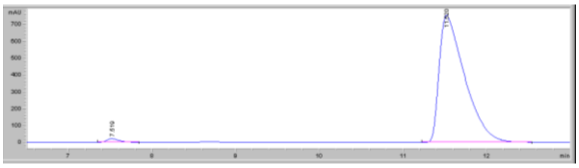

RetTime [min]	Type	Width [min]	Area [mAU*s]	Height [mAU]	Area %
7.375	BB	0.1730	1.07419e4	942.06219	49.5411
11.381	VB	0.2901	1.09409e4	569.70374	50.4589

RetTime [min]	Type	Width [min]	Area [mAU*s]	Height [mAU]	Area %
7.519	BB	0.1523	221.59053	21.84584	1.3715
11.520	BB	0.3145	1.59353e4	754.06317	98.6285

(*S*,*E*)-(5-Isopropylhept-3-ene-1,7-diyl)dibenzene, 11b

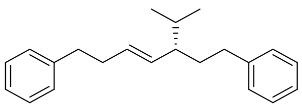

11b

According to **General Procedure B** with **1** (45 mg, 0.21 mmol) and **10**[16] (59 mg, 0.20 mmol, 98.5:1.5 e.r.). The *E*/*Z* ratio of the product was determined to be >98:2 by ^{1}H NMR analysis of the crude reaction mixture. Purification *via* column chromatography eluting with hexane afforded an inseparable mixture of the title compound **11b** with diphenyldiselenide. According to a modified literature procedure,[17] the residue was dissolved in dichloromethane (0.6 mL) and hydrogen peroxide (30 % w/w in water, 50 μL, 0.50 mmol) and pyridine (20 μL, 0.25 mmol) were added sequentially. The resulting biphasic mixture was stirred rapidly at room temperature for 1 hour and then directly purified by preparative TLC (hexane) to afford the title compound **11b** as a colourless oil (45 mg, 75 %, >98:2 *E*/*Z*, 98.5:1.5 e.r.).

^{1}H NMR (400 MHz, $CDCl_3$) δ_H = 7.32-7.25 (m, 4H), 7.24-7.13 (m, 6H), 5.42 (dt, J = 15.3, 6.7 Hz, 1H), 5.23 (ddt, J = 15.3, 9.2, 1.4 Hz, 1H), 2.73 (t, J = 7.6 Hz, 2H), 2.64-2.54 (m, 1H), 2.45-2.36 (m, 3H), 1.81-1.65 (m, 2H), 1.61-1.43 (m, 2H), 0.84 (d, J = 6.8 Hz, 3H), 0.80 (d, J = 6.8 Hz, 3H);

^{13}C NMR (101 MHz, $CDCl_3$) δ_C = 143.3, 142.1, 132.9, 131.0, 128.6, 128.5, 128.3, 128.3, 125.8, 125.6, 49.0, 36.4, 34.6, 34.5, 34.1, 32.1, 20.8, 19.0;

FTIR (neat) ν/cm^{-1} = 2955, 2925, 1496, 1453, 972, 744, 696;

HRMS (EI^{+}): calculated for $C_{22}H_{28}$ = 292.2191, mass found = 292.2189;

$[\alpha]_D^{25}$ = –6 (*c* = 1.00, $CHCl_3$).

Chiral HPLC (Chiralpak IB with guard, 100 % hexane, 1.0 mL/min, 25 °C, λ = 210.8 nm, 5 μL injection):

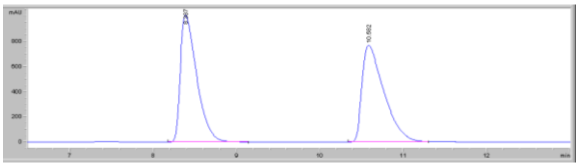

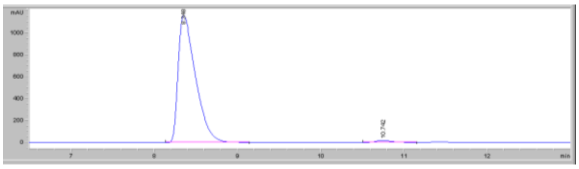

RetTime [min]	Type	Width [min]	Area [mAU*s]	Height [mAU]	Area %
8.387	PB	0.2052	1.37293e4	1005.85498	49.4980
10.582	PV	0.2715	1.40078e4	765.48322	50.5020

RetTime [min]	Type	Width [min]	Area [mAU*s]	Height [mAU]	Area %
8.348	BB	0.2183	1.67724e4	1162.57275	98.5097
10.742	BB	0.2140	253.73335	17.82857	1.4903

***tert*-Butyl(((3*S*,5*R*,6*R*,8*S*,9*S*,10*R*,13*R*,14*S*,17*R*)-10,13-dimethyl-17-((*R*)-6-methylheptan-2-yl)-6-((*Z*)-4-phenylbut-1-en-1-yl)hexadecahydro-1*H*-cyclopenta[*a*]phenanthren-3-yl)oxy)dimethylsilane, 13a**

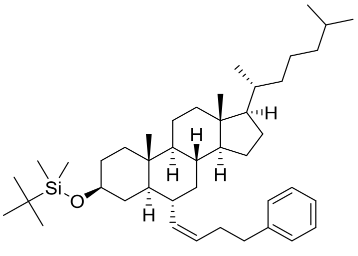

13a

According to **General Procedure A** with **1** (30 mg, 0.14 mmol) and **12**[16] (85 mg, 0.14 mmol). The *Z*/*E* ratio of the product was determined to be 92:8 by ^{1}H NMR analysis of the crude reaction mixture. Purification *via* column chromatography eluting with a gradient from hexane to 1:99 diethyl ether/hexane afforded the title compound **13a** as a colourless oil (57 mg, 67 %, 92:8 *Z*/*E*, >95:5 d.r.).

^{1}H NMR (400 MHz, $CDCl_3$) δ_H = 7.30-7.24 (m, 2H), 7.21-7.14 (m, 3H), 5.37 (dt, J = 10.9, 7.3 Hz, 1H), 4.99 (ddt, J = 11.0, 9.7, 1.6 Hz, 1H), 3.45 (tt, J = 10.8, 4.5 Hz, 1H), 2.72-2.55 (m, 2H), 2.39-2.23 (m, 2H), 2.23-2.11 (m, 1H), 1.96 (dt, J = 12.6, 3.4 Hz, 1H), 1.86-1.61 (m, 4H), 1.58-0.54 (m, 48H), 0.03 (s, 6H);

^{13}C NMR (101 MHz, $CDCl_3$) δ_C = 142.2, 135.5, 128.5, 128.3, 128.0, 125.9, 72.6, 56.5, 56.4, 54.3, 49.0, 42.7, 40.2, 39.6, 39.3, 37.4, 36.3, 36.2, 35.9, 35.9, 35.5, 35.3, 34.7, 32.0, 29.6, 28.4, 28.1, 26.1, 24.3, 23.9, 22.9, 22.7, 21.3, 18.8, 18.4, 13.3, 12.2, -4.4, -4.4;

FTIR (neat) ν/cm^{-1} = 2930, 2853, 1467, 1379, 1251, 1097, 1082, 872, 835, 773, 733, 697;

HRMS (EI^+): calculated for $C_{43}H_{71}OSi$ = 631.5274, mass found = 631.5289;

$[\alpha]_D^{25}$ = +26 (*c* = 1.00, $CHCl_3$).

Alternative preparation of **13a**

According to a modification of **General Procedure C**, a stirred solution of **1** (30 mg, 0.14 mmol) in THF (0.73 mL) was cooled to -78 ^oC and a solution of *tert*-butyllithium (1.7 M in pentane, 0.17 mL, 0.28 mmol) was added dropwise. The resulting solution was stirred at -78 ^oC for 30 minutes and then a solution of **12** (85 mg, 0.14 mmol) in THF (0.66 mL) was added dropwise. The resulting solution was stirred at -78 ^oC for 30 minutes and a solution of PhSeCl (1.0 M in THF, 0.16 mL, 0.16 mmol) was added dropwise. The resulting solution was stirred at -78 ^oC for 30 minutes and then warmed to room temperature and stirred for a further 15 minutes and then cooled to 0 ^oC. A suspension of sodium methoxide (2.0 M in MeOH, 1.35 mL, 2.70 mmol) was added dropwise and the resulting mixture was stirred at 0 ^oC for 1 hour and then at room temperature for 20 hours and then saturated aqueous sodium sulfite was added followed by water and dichloromethane. The organic layer was separated and the aqueous layer was extracted twice with dichloromethane. The combined organic extracts were dried over anhydrous magnesium sulfate, filtered and concentrated under reduced pressure. The *Z*/*E* ratio of the product was determined to be >98:2 *Z*/*E* by ^1H NMR analysis of the crude reaction mixture. Purification *via* column chromatography eluting with a gradient from hexane to 1:99 diethyl ether/hexane followed by preparative TLC (1:49 diethyl ether/hexane) afforded the title compound **13a** as a colourless oil (60 mg, 70 %, >98:2 *Z*/*E*, >95:5 d.r.). The spectral data for this material was identical to that described above.

***tert*-Butyl(((3*S*,5*R*,6*R*,8*S*,9*S*,10*R*,13*R*,14*S*,17*R*)-10,13-dimethyl-17-((*R*)-6-methylheptan-2-yl)-6-((*E*)-4-phenylbut-1-en-1-yl)hexadecahydro-1*H*-cyclopenta[*a*]phenanthren-3-yl)oxy)dimethylsilane, 13b**

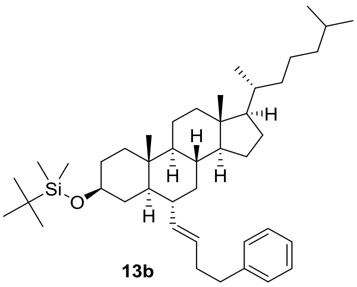

13b

According to **General Procedure B** with **1** (30 mg, 0.14 mmol) and **12** (85 mg, 0.14 mmol, 93.3:6.7 e.r.). The E/Z ratio of the product was determined to be >98:2 by ^{1}H NMR analysis of the crude reaction mixture. Purification *via* column chromatography eluting with a gradient from hexane to 1:99 diethyl ether/hexane afforded the title compound **13b** as a colourless oil (60 mg, 70 %, >98:2 *E*/*Z*, >95:5 d.r.).

^{1}H NMR (400 MHz, $CDCl_3$) δ_H = 7.30-7.26 (m, 2H), 7.20-7.14 (m, 3H), 5.37 (dt, J = 15.2, 6.7 Hz, 1H), 5.09 (ddt, J = 15.3, 9.0, 1.4 Hz, 1H), 3.47 (tt, J = 10.9, 4.6 Hz, 1H), 2.72-2.56 (m, 2H), 2.36-2.19 (m, 2H), 1.96 (dt, J = 12.6, 3.4 Hz, 1H), 1.89-1.61 (m, 5H), 1.60-0.55 (m, 48H), 0.03 (s, 6H).;

^{13}C NMR (101 MHz, $CDCl_3$) δ_C = 142.3, 135.9, 129.1, 128.5, 128.3, 125.8, 72.7, 56.5, 56.4, 54.3, 49.0, 42.7, 41.5, 40.2, 39.9, 39.6, 37.4, 36.6, 36.3, 35.9, 35.5, 35.5, 34.7, 34.6, 31.9, 28.4, 28.1, 26.1, 24.3, 23.9, 22.9, 22.7, 21.3, 18.8, 18.4, 13.3, 12.2, -4.4, -4.4;

FTIR (neat) ν/cm^{-1} = 2930, 2853, 1467, 1380, 1251, 1097, 1083, 968, 872, 835, 773, 698;

HRMS (EI$^+$): calculated for $C_{43}H_{71}OSi$ = 631.5274, mass found = 631.5269;

$[\alpha]_D^{25}$ = +20 (c = 1.00, $CHCl_3$).

((*Z*)-4-((2*R*,5*S*)-2-Isopropyl-5-methylcyclohexyl)but-3-en-1-yl)benzene, 15a

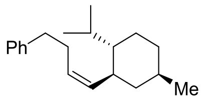

15a

According to **General Procedure A** with **1** (45 mg, 0.21 mmol) and **14**[18] (54 mg, 0.20 mmol, >95:5 d.r.). Purification *via* column chromatography eluting with hexane afforded the title compound **15a** as a colourless oil (35 mg, 64 %, 90:10 *Z/E,* >95:5 d.r.).

^{1}H NMR (400 MHz, $CDCl_3$) δ_H = 7.33 – 7.24 (m, 2H), 7.20 (d, *J* = 7.5 Hz, 3H), 5.35 (dt, *J* = 10.9, 7.2 Hz, 1H), 5.17 – 5.06 (m, 1H), 2.75 – 2.58 (m, 2H), 2.43 – 2.32 (m, 2H), 2.17 (qd, *J* = 10.3, 3.4 Hz, 1H), 1.81 (septd, *J* = 6.8, 2.8 Hz, 1H), 1.74-1.67 (m, 1H), 1.64 – 1.56 (m, 1H), 1.40 – 1.25 (m, 2H), 1.06 – 0.88 (m, 3H), 0.86 (d, *J* = 7.0 Hz, 3H), 0.83 (d, *J* = 6.4 Hz, 3H), 0.81 – 0.72 (m, 1H), 0.69 (d, *J* = 6.9 Hz, 3H);

^{13}C NMR (101 MHz, $CDCl_3$) δ_C = 142.2, 136.0, 128.4, 128.2, 127.3, 125.7, 47.5, 42.7, 39.2, 36.3, 35.2, 32.4, 29.7, 28.2, 24.1, 22.6, 21.6, 15.9;

FTIR (neat) ν/cm^{-1} = 3026, 2951, 2915, 2868, 2842, 1604, 1496, 1453, 1384, 1366, 1349, 1184, 1165, 734, 697;

HRMS (EI$^+$): calculated for $C_{20}H_{30}$ = 270.2348, mass found = 270.2346;

$[\alpha]_D^{25}$ = –33 (c = 0.80, $CHCl_3$).

Alternative preparation of **15a**

According to a modification of **General Procedure C**, a stirred solution of **1** (36 mg, 0.17 mmol) in THF (0.85 mL) was cooled to -78 °C and a solution of *tert*-butyllithium (1.7 M in pentane, 0.20 mL, 0.34 mmol) was added dropwise. The resulting solution was stirred at -78 °C for 30 minutes and then a solution of **12** (43 mg, 0.16 mmol) in THF (0.85 mL) was added dropwise. The resulting solution was

stirred at -78 °C for 30 minutes and a solution of PhSeCl (1.0 M in THF, 0.19 mL, 0.19 mmol) was added dropwise. The resulting solution was stirred at -78 °C for 30 minutes and then warmed to room temperature and stirred for a further 15 minutes and then cooled to 0 °C. A suspension of sodium methoxide (2.0 M in MeOH, 1.60 mL, 3.2 mmol) was added dropwise and the resulting mixture was stirred at 0 °C for 1 hour and then at room temperature for 20 hours and then saturated aqueous sodium sulfite was added followed by water and dichloromethane. The organic layer was separated and the aqueous layer was extracted twice with dichloromethane. The combined organic extracts were dried over anhydrous magnesium sulfate, filtered and concentrated under reduced pressure. Purification *via* column chromatography eluting with hexane afforded the title compound **15a** as a colourless oil (23 mg, 53 %, >98:2 *Z*/*E*, >95:5 d.r.). The spectral data for this material was identical to that described above.

((*E*)-4-((2*R*,5*S*)-2-isopropyl-5-methylcyclohexyl)but-3-en-1-yl)benzene, 15b

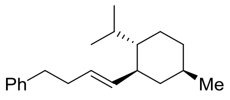

15b

According to **General Procedure B** with **1** (23 mg, 0.11 mmol) and **14**[18] (27 mg, 0.10 mmol, >95:5 d.r.). Purification *via* column chromatography eluting with hexane afforded the title compound **15b** as a colourless oil (16 mg, 60 %, >98:2 *E*/*Z*, >95:5 d.r.).

^{1}H NMR (400 MHz, CDCl$_3$) δ_H = 7.31 – 7.22 (m, 2H), 7.21 – 7.12 (m, 3H), 5.37 (dt, *J* = 15.4, 6.7 Hz, 1H), 5.15 (dd, *J* = 15.3, 9.1 Hz, 1H), 2.73-2.61 (m, 2H), 2.36 – 2.23 (m, 2H), 1.88 – 1.64 (m, 3H), 1.63 – 1.48 (m, 2H), 1.43 – 1.24 (m, 2H), 1.03 – 0.85 (m, 3H), 0.85 (d, *J* = 6.5 Hz, 3H), 0.83 (d, *J* = 7.0 Hz, 3H), 0.66 (d, *J* = 6.9 Hz, 3H);

^{13}C NMR (101 MHz, CDCl$_3$) δ_C = 142.1, 136.2, 128.5, 128.1, 128.1, 125.6, 47.1, 44.6, 43.5, 36.2, 35.2, 34.4, 32.5, 27.8, 24.1, 22.6, 21.5, 15.3;

FTIR (neat) ν/cm^{-1} = 3027, 2951, 2914, 2868, 2843, 1604, 1496, 1454, 1384, 1367, 968, 744, 697;

HRMS (EI$^+$): calculated for $C_{20}H_{30}$ = 270.2348, mass found = 270.2349;

$[\alpha]_D^{25}$ = –36 (*c* = 0.80, $CHCl_3$).

(3*Z*,5*E*)-1,8-Diphenylocta-3,5-diene, 17a

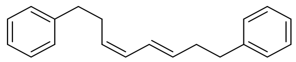

17a

According to **General Procedure A** with **1** (45 mg, 0.21 mmol) and **16** (52 mg, 0.20 mmol). The *Z*,*E*/*E*,*E* ratio of the product was determined to be >98:2 by ^{1}H NMR analysis of the crude reaction mixture. Purification *via* column chromatography eluting with a gradient from pentane to 1:99 diethyl ether/pentane afforded the title compound **17a** as a colourless oil (38 mg, 71 %, >98:2 *Z*,*E*/*E*,*E*).

^{1}H NMR (400 MHz, $CDCl_3$) δ_H = 7.35 – 7.26 (m, 4H), 7.25 – 7.15 (m, 6H), 6.31 (ddq, J = 15.1, 11.0, 1.4 Hz, 1H), 5.98 (tq, J = 11.0, 1.4 Hz, 1H), 5.71 (dt, J = 15.0, 6.9 Hz, 1H), 5.38 (dt, J = 10.9, 7.5 Hz, 1H), 2.75 – 2.66 (m, 4H), 2.53 – 2.38 (m, 4H);

^{13}C NMR (101 MHz, $CDCl_3$) δ_C = 142.0, 141.9, 133.9, 129.2, 129.2, 128.6, 128.5, 128.4, 128.4, 126.1, 125.9, 125.9, 36.0, 35.9, 34.8, 29.7;

FTIR (neat) ν/cm^{-1} = 3025, 2924, 1603, 1496, 1453, 983, 948, 747, 698;

HRMS (EI$^+$): calculated for $C_{20}H_{22}$ = 262.1722, mass found = 262.1720.

(3*E*,5*E*)-1,8-Diphenylocta-3,5-diene, 17b

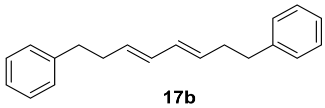

17b

A stirred solution of **1** (45 mg, 0.21 mmol) in THF (1.0 mL) was cooled to -78 °C and a solution of *tert*-butyllithium (1.7 M in pentane, 0.25 mL, 0.43 mmol) was added dropwise. The resulting solution was stirred at -78 °C for 30 minutes and then a solution of **16** (52 mg, 0.20 mmol) in THF (1.0 mL) was added dropwise. The resulting solution was stirred at -78 °C for 30 minutes and then a solution of PhSeCl (1.0 M in THF, 0.24 mL, 0.24 mmol) was added dropwise. The resulting solution was stirred at -78 °C for 30 minutes and then warmed to room temperature and stirred for a further 15 minutes and then filtered through a short plug of silica gel, washing with diethyl ether. The filtrate was concentrated under reduced pressure and the residue was dissolved in THF (2.0 mL). The resulting stirred solution was cooled to -78 °C and a solution of *m*CPBA (0.2 M in THF, 2.0 mL, 0.40 mmol) was added dropwise [n.b. in order to obtain high *E*-selectivity with benzylic or allylic substrates it is critical to add the *m*CPBA solution slowly, i.e. over 5 min]. The resulting solution was warmed to -45 °C (acetonitrile/dry ice bath) and stirred for 30 minutes. After this time, dimethylsulfide (0.3 mL, 4.0 mmol) was added and the mixture was allowed to warm to room temperature. Saturated aqueous sodium sulfite was added followed by water and diethyl ether. The organic layer was separated and the aqueous layer was extracted twice with diethyl ether. The combined organic extracts were washed with saturated aqueous potassium carbonate and brine and then dried over anhydrous magnesium sulfate, filtered and concentrated under reduced pressure [*N.B.* basic aqueous workup was necessary because our standard procedure of filtration through silica gel led to isomerization of the product by residual chlorobenzoic acid]. Purification *via* column chromatography eluting with a gradient from pentane to 1:199 diethyl ether/pentane afforded the title compound **17b** as a colourless oil (42 mg, 79 %, 95:5 *E*,*E*/*Z*,*E*).

^{1}H NMR (400 MHz, $CDCl_3$) δ_H = 7.35 – 7.27 (m, 4H), 7.24 – 7.18 (m, 6H), 6.12 – 6.02 (m, 2H), 5.72 – 5.58 (m, 2H), 2.72 (t, J = 7.4 Hz, 4H), 2.41 (q, J = 7.4 Hz, 4H);

^{13}C NMR (101 MHz, $CDCl_3$) δ_C = 141.9, 131.7, 130.8, 128.4, 128.3, 125.8, 35.9, 34.5;

FTIR (neat) ν/cm^{-1} = 3025, 2924, 1603, 1496, 1454, 988, 744, 698;

HRMS (EI$^+$): calculated for $C_{20}H_{22}$ = 262.1722, mass found = 262.1733.

(*Z*)-But-1-ene-1,4-diyldibenzene, 18a

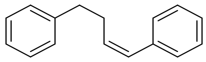

18a

A stirred solution of **16** (52 mg, 0.20 mmol) in THF (2.0 mL) was cooled to -78 °C and phenyl lithium (1.9 M in dibutyl ether, 0.11 mL, 0.21 mmol) was added dropwise. The resulting solution was stirred at -78 °C for 30 minutes and then warmed to 0 °C and stirred for 2 minutes. After this time, a suspension of sodium methoxide (3.0 M in MeOH, 0.20 mL, 0.60 mmol) was added in a single portion followed by dropwise addition of a solution of iodine (0.5 M in MeOH, 0.49 mL, 0.24 mmol). The resulting mixture was stirred at 0 °C for 30 minutes and then saturated aqueous sodium sulfite was added followed by water and dichloromethane. The organic layer was separated and the aqueous layer was extracted twice with dichloromethane. The combined organic extracts were dried over anhydrous magnesium sulfate, filtered and concentrated under reduced pressure. The *Z*/*E* ratio of the product was determined to be >98:2 by ^{1}H NMR analysis of the crude reaction mixture. Purification of the residue *via* column chromatography eluting with pentane afforded the title compound **18a** as a colourless oil (37 mg, 88 %, >98:2 *Z*/*E*). The spectral data matched that previously reported in the literature.[19]

^{1}H NMR (400 MHz, $CDCl_3$) δ_H = 7.37 – 7.20 (m, 10H), 6.48 (dt, J = 11.7, 1.9 Hz, 1H), 5.74 (dt, J = 11.6, 7.0 Hz, 1H), 2.83 – 2.77 (m, 2H), 2.74 – 2.66 (m, 2H);

^{13}C NMR (101 MHz, $CDCl_3$) δ_C = 141.8, 137.7, 131.9, 129.6, 128.8, 128.6, 128.5, 128.3, 126.7, 126.0, 36.2, 30.5;

FTIR (neat) ν/cm^{-1} = 3025, 2923, 1601, 1494, 1453, 1076, 766, 748, 697;

LRMS (EI^+): calculated for $C_{16}H_{16}^+$ = 208, mass found = 208.

(*E*)-But-1-ene-1,4-diyldibenzene, 18b

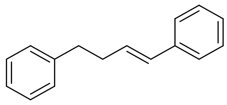

18b

A stirred solution of **16** (52 mg, 0.20 mmol) in THF (2.0 mL) was cooled to -78 °C and phenyl lithium (1.9 M in dibutyl ether, 0.11 mL, 0.21 mmol) was added dropwise. The resulting solution was stirred at -78 °C for 30 minutes and then a solution of PhSeCl (1.0 M in THF, 0.24 mL, 0.24 mmol) was added dropwise. The resulting solution was stirred at -78 °C for 30 minutes and then warmed to room temperature and stirred for a further 15 minutes and then filtered through a short plug of silica gel washing with diethyl ether. The filtrate was concentrated under reduced pressure and the residue was dissolved in THF (2.0 mL). The resulting stirred solution was cooled to -78 °C and a solution of *m*CPBA (0.2 M in THF, 2.0 mL, 0.40 mmol) was added dropwise [N.B. in order to obtain high *E*-selectivity with benzylic or allylic substrates it is critical to add the *m*CPBA solution slowly, i.e. over 5 min]. The resulting solution was warmed to -45 °C (acetonitrile/dry ice bath) and stirred for 30 minutes. After this time dimethylsulfide (0.3 mL, 4.0 mmol) was added and the mixture was allowed to warm to room temperature and then filtered through a short plug of silica gel, washing with diethyl ether. The filtrate was concentrated under reduced pressure. The *E*/*Z* ratio of the product was determined to be 97:3 by ^1H NMR analysis of the crude reaction mixture. Purification *via* column chromatography eluting with pentane afforded the title compound **18b** as a colourless oil (38 mg, 90 %, 98:2 *E*/*Z*). The spectral data matched that previously reported in the literature.[20]

^{1}H NMR (400 MHz, $CDCl_3$) δ_H = 7.38 – 7.28 (m, 6H), 7.27 – 7.19 (m, 4H), 6.44 (d, *J* = 15.8 Hz, 1H), 6.28 (dt, *J* = 15.8, 6.7 Hz, 1H), 2.82 (t, *J* = 7.0 Hz, 2H), 2.56 (q, *J* = 7.0 Hz, 2H);

^{13}C NMR (101 MHz, $CDCl_3$) δ_C = 141.9, 137.8, 130.5, 130.1, 128.6, 128.6, 128.5, 127.0, 126.1, 126.0, 36.0, 35.0;

FTIR (neat) ν/cm^{-1} = 3025, 2924, 1601, 1496, 1453, 963, 738, 692;

LRMS (EI^+): calculated for $C_{16}H_{16}^+$ = 208, mass found = 208.

(*Z*)-Pent-3-en-1-ylbenzene, 19a

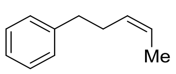

19a

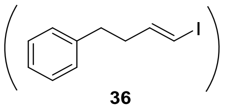

36

A stirred solution of **16** (52 mg, 0.20 mmol) in THF (2.1 mL) was cooled to -78 °C and a solution of methyl lithium (1.6 M in diethyl ether, 0.13 mL, 0.21 mmol) was added dropwise. The resulting solution was stirred at -78 °C for 30 minutes and then warmed to 0 °C and stirred for 5 minutes. After this time, a suspension of sodium methoxide (3.0 M in MeOH, 0.20 mL, 0.60 mmol) was added in a single portion, followed by dropwise addition of a solution of iodine (0.5 M in MeOH, 0.49 mL, 0.24 mmol). The resulting mixture was stirred at 0 °C for 30 minutes and then saturated aqueous sodium sulfite was added followed by water and dichloromethane. The organic layer was separated and the aqueous layer was extracted twice with dichloromethane. The combined organic extracts were dried over anhydrous magnesium sulfate, filtered and concentrated under reduced pressure. Purification of the residue *via* column chromatography eluting with pentane afforded the title compound **19a** as a colourless oil (8 mg, 27 %, >98:2 *Z*/*E*) along with (*E*)-(4-iodobut-3-en-1-yl)benzene **36** as a colourless oil (15 mg, 29 %).

(*Z*)-Pent-3-en-1-ylbenzene, **19a**

The spectral data matched that reported in the literature.[21]

^{1}H NMR (400 MHz, $CDCl_3$) δ_H = 7.30-7.25 (m, 2H), 7.22-7.15 (m, 3H), 5.52-5.38 (m, 2H), 2.66 (t, *J* = 7.8 Hz, 2H), 2.40-2.33 (m, 2H), 1.58-1.54 (m, 3H);

^{13}C NMR (101 MHz, $CDCl_3$) δ_C = 142.3, 129.7, 128.5, 128.3, 125.8, 124.6, 35.9, 28.9, 12.8;

FTIR (neat) ν/cm^{-1} = 3020, 2926, 1604, 1496, 1453, 748, 697;

LRMS (EI$^+$): calculated for $C_{11}H_{14}^+$ = 146, mass found = 146.

<u>(*E*)-(4-Iodobut-3-en-1-yl)benzene, **36**</u>

The spectral data matched that reported in the literature.[22]

^{1}H NMR (400 MHz, $CDCl_3$) δ_H = 7.32 – 7.26 (m, 2H), 7.22 – 7.13 (m, 3H), 6.55 (dt, J = 14.2, 7.1 Hz, 1H), 6.02 (dt, J = 14.4, 1.4 Hz, 1H), 2.71 (t, J = 7.7 Hz, 2H), 2.40 – 2.33 (m, 2H);

^{13}C NMR (101 MHz, $CDCl_3$) δ_C = 145.6, 140.9, 128.5, 128.5, 126.2, 75.4, 37.8, 34.9;

FTIR (neat) ν/cm^{-1} = 3025, 2922, 1603, 1496, 1453, 1205, 940, 752;

LRMS (EI$^+$): calculated for $C_{10}H_{11}$ = 131.09, mass found = 131.09 [*N.B. mass corresponds to loss of I*]

Alternative preparation of **19a**

A stirred solution of **16** (49 mg, 0.19 mmol) in THF (1.9 mL) was cooled to -78 °C and a solution of methyl lithium (1.6 M in diethyl ether, 0.13 mL, 0.21 mmol) was added dropwise. The resulting solution was stirred at -78 °C for 30 minutes and then a solution of PhSeCl (1.0 M in THF, 0.23 mL, 0.23 mmol) was added dropwise. The resulting solution was stirred at -78 °C for 30 minutes and then warmed to room temperature and stirred for a further 15 minutes and then cooled to 0 °C. A solution of sodium methoxide (0.5 M in MeOH, 1.9 mL, 0.95 mmol) was added dropwise and the resulting solution was stirred at 0 °C for 2 hours and then saturated aqueous sodium sulfite was added, followed by water and dichloromethane. The organic layer was separated and the aqueous layer was extracted twice with dichloromethane. The combined organic extracts were dried over anhydrous magnesium

sulfate, filtered and concentrated under reduced pressure. The *Z*/*E* ratio of the product was determined to be >98:2 by ^{1}H NMR analysis of the crude reaction mixture. Purification of the residue *via* column chromatography eluting with pentane afforded the title compound **19a** as a colourless oil (17 mg, 61 %, >98:2 *Z*/*E*). The spectral data for this material was identical to that described above.

(*E*)-Pent-3-en-1-ylbenzene, 19b

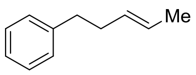

19b

A stirred solution of **16** (52 mg, 0.20 mmol) in THF (2.0 mL) was cooled to -78 °C and a solution of methyl lithium (1.6 M in diethyl ether, 0.13 mL, 0.21 mmol) was added dropwise. The resulting solution was stirred at -78 °C for 30 minutes and then a solution of PhSeCl (1.0 M in THF, 0.25 mL, 0.25 mmol) was added dropwise. The resulting solution was stirred at -78 °C for 30 minutes and then warmed to room temperature and stirred for a further 15 minutes and then filtered through a short plug of silica gel washing with diethyl ether. The filtrate was concentrated under reduced pressure and the residue was dissolved in THF (2.0 mL). The resulting stirred solution was cooled to -78 °C and a solution of *m*CPBA (0.2 M in THF, 2.0 mL, 0.41 mmol) was added dropwise. The resulting solution was warmed to -45 °C (acetonitrile/dry ice bath) and stirred for 30 minutes. After this time dimethylsulfide (0.30 mL, 4.0 mmol) was added and the mixture was allowed to warm to room temperature and then filtered through a short plug of silica gel washing with diethyl ether. The filtrate was concentrated under reduced pressure. The *E*/*Z* ratio of the product was determined to be >98:2 by ^{1}H NMR analysis of the crude reaction mixture. Purification of the residue *via* column chromatography eluting with pentane afforded the title compound **19b** as a colourless oil (19 mg, 64 %, >98:2 *E*/*Z*). The spectral data matched that previously reported in the literature.[23,24]

^{1}H NMR (400 MHz, $CDCl_3$) δ_H = 7.30-7.23 (m, 2H), 7.20-7.14 (m, 3H), 5.53-5.40 (m, 2H), 2.66 (t, *J* = 7.9 Hz, 2H), 2.33-2.25 (m, 2H), 1.66-1.62 (m, 3H);

^{13}C NMR (101 MHz, $CDCl_3$) δ_C = 142.3, 130.7, 128.5, 128.3, 125.8, 125.5, 36.2, 34.5, 18.0;

FTIR (neat) ν/cm^{-1} = 2924, 1604, 1496, 1453, 965, 742, 697;

LRMS (EI^+): calculated for $C_{11}H_{14}^+$ = 146, mass found = 146.

(*Z*)-(5,5-dimethylhex-3-en-1-yl)benzene, 34a

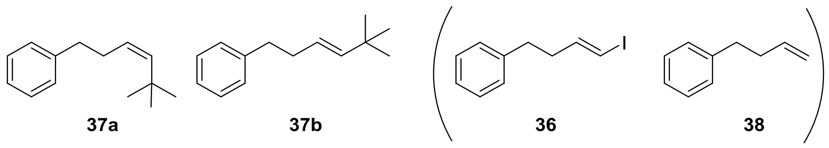

A stirred solution of **1** (45 mg, 0.21 mmol) in THF (1.0 mL) was cooled to -78 °C and a solution of *tert*-butyllithium (1.7 M in pentane, 0.25 mL, 0.43 mmol) was added dropwise. The resulting solution was stirred at -78 °C for 30 minutes and then a solution of *tert*-butylboronic acid pinacol ester (37 mg, 0.20 mmol) in THF (1.0 mL) was added dropwise. The resulting solution was stirred at -78 °C for 30 minutes and then warmed to 0 °C and stirred for 1 hour. After this time, a suspension of sodium methoxide (3.0 M in MeOH, 0.20 mL, 0.60 mmol) was added in a single portion followed by dropwise addition of a solution of iodine (0.5 M in MeOH, 2.0 mL, 1.0 mmol). The resulting mixture was stirred at 0 °C for 30 minutes and then saturated aqueous sodium sulfite was added followed by water and dichloromethane. The organic layer was separated and the aqueous layer was extracted twice with dichloromethane. The combined organic extracts were dried over anhydrous magnesium sulfate, filtered and concentrated under reduced pressure. Crude ^{1}H NMR of the residue *vs* 1,3,5-trimethoxybenzene (0.067 mmol) as an internal standard indicated that the title compound **37a** was present in 38 % NMR yield (6:1 *Z*/*E*) along with vinyl iodide **36** (13 % NMR yield) and alkene **38** (13 % NMR yield). The ^{1}H NMR signals for **37a**,[25] **37b**,[26] and **38**[27] matched those previously reported in the literature. The spectral data for **36** was identical to that described above. Diagnostic ^{1}H NMR signals for **37a**, **37b** and **38** are given below.

Diagnostic data for **37a** (33 % NMR yield): ^{1}H NMR (400 MHz, $CDCl_3$). δ_H = 5.34 (dt, *J* = 11.9, 1.5 Hz, 1H), 5.20 (dt, *J* = 11.9, 7.2 Hz, 1H), 2.54 – 2.44 (m, 2H), 1.08 (s, 9H).

Diagnostic data for **37b** (5 % NMR yield): ^{1}H NMR (400 MHz, $CDCl_3$). δ_H = 5.44 (dt, *J* = 15.5, 0.8 Hz, 1H), 5.34 (dt, *J* = 15.6, 6.4 Hz, 1H), 2.68 – 2.62 (m, 2H), 2.32 – 2.23 (m, 2H), 0.97 (s, 9H).

Diagnostic data for alkene **38** (13 % NMR yield): ^{1}H NMR (400 MHz, $CDCl_3$). δ_H = 5.86 (ddt, *J* = 16.9, 10.2, 6.5 Hz, 1H), 5.04 (ddt, *J* = 17.1, 2.0, 1.6 Hz, 1H), 4.97 (ddt, *J* = 10.2, 2.0, 1.2 Hz, 1H).

(*Z*)-Pent-3-en-1-ylbenzene, 37b

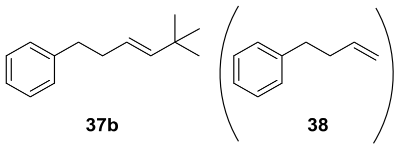

37b **38**

A stirred solution of **1** (45 mg, 0.21 mmol) in THF (1.0 mL) was cooled to -78 °C and a solution of *tert*-butyllithium (1.7 M in pentane, 0.25 mL, 0.43 mmol) was added dropwise. The resulting solution was stirred at -78 °C for 30 minutes and then a solution of *tert*-butylboronic acid pinacol ester (37 mg, 0.20 mmol) in THF (1.0 mL) was added dropwise. The resulting solution was stirred at -78 °C for 30 minutes and then warmed to 0 °C and stirred for 1 hour. The resulting solution was then cooled to -78 °C and a solution of PhSeCl (1.0 M in THF, 0.24 mL, 0.24 mmol) was added dropwise. The resulting solution was stirred at -78 °C for 30 minutes and then warmed to room temperature and stirred for a further 15 minutes and then filtered through a short plug of silica gel, washing with diethyl ether. The filtrate was concentrated under reduced pressure and the residue was dissolved in THF (2.0 mL). The resulting stirred solution was cooled to -78 °C and a solution of *m*CPBA (0.2 M in THF, 2.0 mL, 0.40 mmol) was added dropwise. The resulting solution was warmed to -45 °C (acetonitrile/dry ice bath) and stirred for 30 minutes. After this time dimethylsulfide (0.30 mL, 4.0 mmol) was added and the mixture was allowed to warm to room temperature and then filtered through a short plug of silica gel washing with diethyl ether. The filtrate was concentrated under reduced pressure. Crude ^{1}H NMR of the residue *vs* 1,3,5-trimethoxybenzene (0.067 mmol) as an internal standard indicated that the

title compound **37b** was present in 11 % NMR yield (>95:5 *E*/*Z*) along with alkene **38** (1 % NMR yield) and several other unidentified impurities. The spectral data for **37b** and **38** was identical to that described above.

(*R*,*Z*)-1-(3,4-Dimethylhex-4-en-1-yl)-4-methoxybenzene, 21a

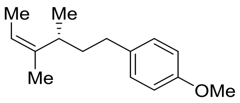

21a

A stirred solution of (*E*)-2-bromobut-2-ene **20** (41 μL, 0.41 mmol) in THF (1.0 mL) was cooled to -78 °C and a solution of *tert*-butyllithium (1.7 M in pentane, 0.48 mL, 0.81 mmol) was added dropwise. The resulting solution was stirred at -78 °C for 30 minutes and then a solution of **2**[13] (59 mg, 0.20 mmol) in THF (1.0 mL) was added dropwise. The resulting solution was stirred at -78 °C for 30 minutes and then a suspension of sodium methoxide (3.0 M in MeOH, 0.20 mL, 0.60 mmol) was added in a single portion followed by dropwise addition of a solution of iodine (0.5 M in MeOH, 0.49 mL, 0.24 mmol). The resulting mixture was warmed to 0 °C and stirred for 30 minutes and then saturated aqueous sodium sulfite was added followed by water and dichloromethane. The organic layer was separated and the aqueous layer was extracted twice with dichloromethane. The combined organic extracts were dried over anhydrous magnesium sulfate, filtered and concentrated under reduced pressure. The *E*- and *Z*-isomers of the product possessed no diagnostic ^{1}H NMR signals, so the *Z*/*E* ratio was determined by GC following purification. Purification of the residue *via* column chromatography eluting with 99:1 pentane/diethyl ether, taking care not to remove any of the minor isomer afforded the title compound **21a** as a colourless oil (42 mg, 95 %, >98:2 *Z*/*E* by GC, 95.3:4.7 e.r.). The *E*/*Z* selectivity was confirmed by nOe (see NMR spectra).

^{1}H NMR (400 MHz, $CDCl_3$) δ_H = 7.08 (d, J = 8.8 Hz, 2H), 6.81 (d, J = 8.6 Hz, 2H), 5.24 (qq, J = 6.8, 1.3 Hz, 1H), 3.78 (s, 3H), 2.74 – 2.63 (m, 1H), 2.52 – 2.38 (m, 2H), 1.63 – 1.56 (m, 5H), 1.53 (dq, J = 6.8, 1.5 Hz, 3H), 0.97 (d, J = 6.9 Hz, 3H);

^{13}C NMR (101 MHz, $CDCl_3$) δ_C = 157.7, 139.5, 135.1, 129.3, 119.3, 113.7, 55.3, 37.0, 33.2, 33.1, 19.1, 18.1, 12.9;

FTIR (neat) ν/cm^{-1} = 2958, 2930, 1612, 1511, 1455, 1300, 1244, 1176, 1039, 820, 808;

HRMS (ESI^+): calculated for $C_{15}H_{22}NaO^+$ = 241.1563, mass found = 241.1566;

$[\alpha]_D^{25}$ = +40 (*c* = 1.00, $CHCl_3$);

Chiral GC: Chiraldex *β*-DM, injector T = 250 °C, detector T = 300 °C. Oven conditions: T = 70 °C for 5 min then ramp (10 °C/min) until 130 °C, then ramp (0.2 °C/min) until 136.5 °C, then ramp (20 °C/min) until 180 °C, hold for 5 min. He carrier gas at 1.0 mL/min. *N.B. in enantioenriched spectrum, peak at 34.6 min corresponds to both enantiomers of the E-isomer (99.5:0.5 Z/E).*

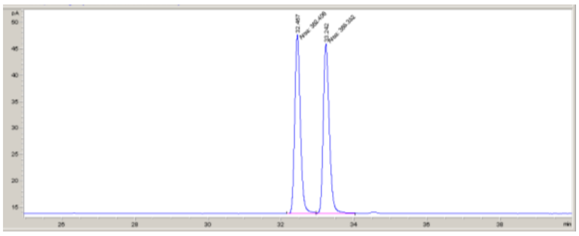

RetTime [min]	Type	Width [min]	Area [pA*s]	Height [pA]	Area %
32.457	MF	0.1788	362.40585	33.77582	49.79761
33.242	FM	0.1904	365.35162	31.98371	50.20239

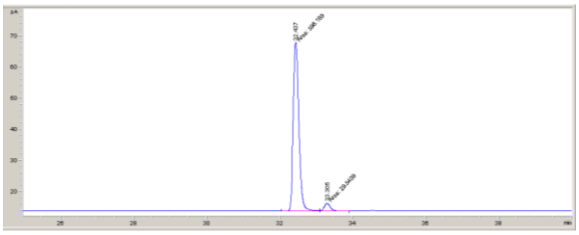

RetTime [min]	Type	Width [min]	Area [pA*s]	Height [pA]	Area %
32.437	MF	0.1846	598.16901	54.01247	95.29387
33.305	FM	0.1929	29.54086	2.55252	4.70613

Alternative preparation of **21a**

A stirred solution of (*Z*)-2-bromobut-2-ene **22** (41 μL, 0.41 mmol) in THF (0.5 mL) was cooled to -78 °C and a solution of *tert*-butyllithium (1.7 M in pentane, 0.48 mL, 0.81 mmol) was added dropwise. The resulting solution was stirred at -78 °C for 30 minutes and then a solution of **2**[13] (59 mg, 0.20 mmol) in THF (0.5 mL) was added dropwise. The resulting solution was stirred at -78 °C for 30 minutes and then 2,2,2-trifluoroethanol (1.0 mL) was added dropwise. Freshly ground phenylselenyl chloride (47 mg, 0.24 mmol) was added in a single portion and the resulting mixture was stirred rapidly at -78 °C and then warmed to room temperature and stirred for 15 minutes and then concentrated under reduced pressure. The residue was filtered through a short plug of silica gel washing with diethyl ether. The

filtrate was concentrated under reduced pressure and the residue was dissolved in THF (2.0 mL). The resulting stirred solution was cooled to -78 °C and a solution of *m*CPBA (0.2 M in THF, 2.0 mL, 0.40 mmol) in THF (2.0 mL) was added dropwise. The resulting solution was warmed to -45 °C (acetonitrile/dry ice bath) and stirred for 30 minutes. After this time dimethylsulfide (0.30 mL, 4.1 mmol) was added and the mixture was allowed to warm to room temperature and then filtered through a short plug of silica gel, washing with diethyl ether. The filtrate was concentrated under reduced pressure. The *E*- and *Z*- isomers of the product possessed no diagnostic ^{1}H NMR signals, so the *Z*/*E* ratio was determined by GC following purification. Purification of the residue *via* column chromatography eluting with 99:1 pentane/diethyl ether, taking care not to remove any of the minor isomer afforded the title compound **21a** as a colourless oil (41 mg, 93 %, 97:3 *Z*/*E* by GC, 95.6:4.4 e.r.). The spectral data for this material was identical to that described above.

Chiral GC: Chiraldex *β*-DM, injector T = 250 °C, detector T = 300 °C. Oven conditions: T = 70 °C for 5 min then ramp (10 °C/min) until 130 °C, then ramp (0.2 °C/min) until 136.5 °C, then ramp (20 °C/min) until 180 °C, hold for 5 min. He carrier gas at 1.0 mL/min. *N.B. in enantioenriched spectrum, peak at 34.6 min corresponds to both enantiomers of the E-isomer (97.5:2.5 Z/E).*

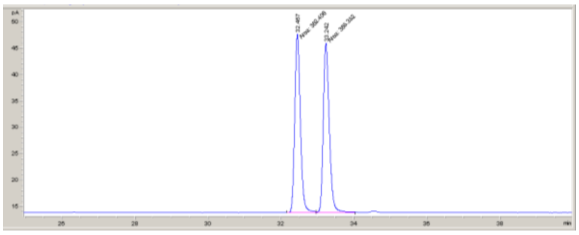

RetTime [min]	Type	Width [min]	Area [pA*s]	Height [pA]	Area %
32.457	MF	0.1788	362.40585	33.77582	49.79761
33.242	FM	0.1904	365.35162	31.98371	50.20239

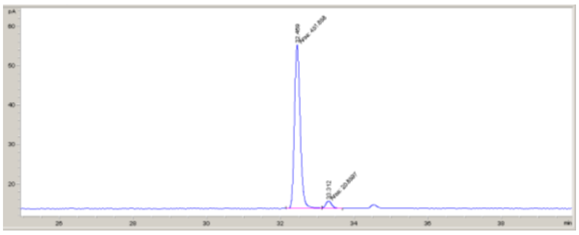

RetTime [min]	Type	Width [min]	Area [pA*s]	Height [pA]	Area %
32.459	MF	0.1814	451.65814	41.48993	95.58542
33.312	FM	0.1889	20.85968	1.84079	4.41458

(*R*,*E*)-1-(3,4-Dimethylhex-4-en-1-yl)-4-methoxybenzene, 21b

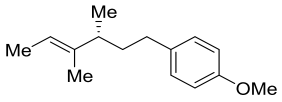

21b

A stirred solution of (*E*)-2-bromobut-2-ene **20** (41 μL, 0.41 mmol) in THF (0.5 mL) was cooled to -78 °C and a solution of *tert*-butyllithium (1.7 M in pentane, 0.48 mL, 0.81 mmol) was added dropwise. The resulting solution was stirred at -78 °C for 30 minutes and then a solution of **2**[13] (59 mg, 0.20 mmol) in THF (0.5 mL) was added dropwise. The resulting solution was stirred at -78 °C for 30 minutes and then 2,2,2-trifluoroethanol (1.0 mL) was added dropwise. Freshly ground phenylselenyl chloride (47 mg, 0.24 mmol) was added in a single portion and the resulting mixture was stirred rapidly at -78 °C and then warmed to room temperature and stirred for 15 minutes and then concentrated under reduced pressure. The residue was filtered through a short plug of silica gel, washing with diethyl ether. The filtrate was concentrated under reduced pressure and the residue was dissolved in THF (2.0 mL). The resulting stirred solution was cooled to -78 °C and a solution of *m*CPBA (0.2 M, 2.0 mL, 0.40 mmol) in THF (2.0 mL) was added dropwise. The resulting solution was warmed to -45 °C (acetonitrile/dry ice bath) and stirred for 30 minutes. After this time dimethylsulfide (0.30 mL, 4.1 mmol) was added and the mixture was allowed to warm to room temperature and then filtered through a short plug of silica gel, washing with diethyl ether. The filtrate was concentrated under reduced pressure. The *E*- and *Z*-isomers of the product possessed no diagnostic ^{1}H NMR signals, so the *E*/*Z* ratio was determined by GC following purification. Purification of the residue *via* column chromatography eluting with 99:1 pentane/diethyl ether, taking care not to remove any of the minor isomer afforded the title compound **21b** as a colourless oil (37 mg, 83 %, 96:4 *E*/*Z* by GC, 95.4:4.6 e.r.).

^{1}H NMR (400 MHz, $CDCl_3$) δ_H = 7.07 (d, J = 8.6 Hz, 2H), 6.81 (d, J = 8.6 Hz, 2H), 5.24 (q, J = 6.5 Hz, 1H), 3.78 (s, 3H), 2.43 (t, J = 8.0 Hz, 2H), 2.12 (sex, J = 7.0 Hz, 1H), 1.69 – 1.42 (m, 8H), 0.99 (d, J = 6.9 Hz, 3H);

^{13}C NMR (101 MHz, $CDCl_3$) δ_C = 157.6, 139.6, 135.2, 129.3, 118.2, 113.7, 55.3, 42.5, 37.1, 33.2, 19.9, 13.3, 11.9;

FTIR (neat) ν/cm^{-1} = 2956, 2924, 1612, 1511, 1457, 1244, 1176, 1039, 821;

HRMS (ESI$^+$): calculated for $C_{15}H_{22}NaO^+$ = 241.1563, mass found = 241.1562;

$[\alpha]_D^{25}$ = +9 (*c* = 1.00, $CHCl_3$);

Chiral HPLC (2 x Chiralpak IA (no guard), 100 % hexane, 0.5 mL/min, 25 °C, λ = 210.8 nm, 5 μL injection): *N.B. in enantioenriched spectrum, peak at 23.2 min corresponds to major enantiomer of the Z-isomer.*

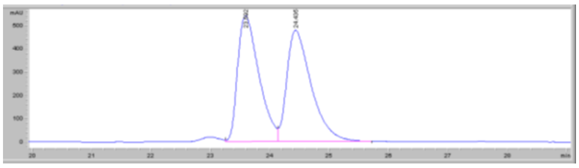

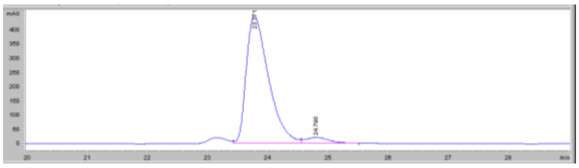

RetTime [min]	Type	Width [min]	Area [mAU*s]	Height [mAU]	Area %
23.592	VV	0.3822	1.34740e4	541.63336	49.5715
24.435	VB	0.4363	1.37069e4	478.33783	50.4285

RetTime [min]	Type	Width [min]	Area [mAU*s]	Height [mAU]	Area %
23.771	VV	0.3935	1.15785e4	447.98227	95.4272
24.790	VB	0.3786	554.82745	20.70750	4.5728

Alternative preparation of **21b**

A stirred solution of (*Z*)-2-bromobut-2-ene **22** (41 μL, 0.41 mmol) in THF (1.0 mL) was cooled to -78 °C and a solution of *tert*-butyllithium (1.7 M in pentane, 0.48 mL, 0.81 mmol) was added dropwise. The resulting solution was stirred at -78 °C for 30 minutes and then a solution of **2**[13] (59 mg, 0.20 mmol) in THF (1.0 mL) was added dropwise. The resulting solution was stirred at -78 °C for 30 minutes and then a suspension of sodium methoxide (3 M in MeOH, 0.20 mL, 0.60 mmol) was added in a single portion followed by dropwise addition of a solution of iodine (0.5 M in MeOH, 0.49 mL, 0.24 mmol). The resulting mixture was warmed to 0 °C and stirred for 30 minutes and then saturated aqueous sodium sulfite was added followed by water and dichloromethane. The organic layer was separated and the aqueous layer was extracted twice with dichloromethane. The combined organic extracts were dried

over anhydrous magnesium sulfate, filtered and concentrated under reduced pressure. The *E*- and *Z*-isomers of the product possessed no diagnostic ^{1}H NMR signals, so the *E/Z* ratio was determined by GC following purification. Purification of the residue *via* column chromatography eluting with 99:1 pentane/diethyl ether, taking care not to remove any of the minor isomer afforded the title compound **21b** as a colourless oil (41 mg, 93 %, 96:4 *E/Z* by GC, 95.5:4.5 e.r.). The *E/Z* selectivity was confirmed by nOe (see NMR spectra). The spectral data for this material was identical to that described above.

Chiral HPLC (2 x Chiralpak IA (no guard), 100 % hexane, 0.5 mL/min, 25 °C, λ = 210.8 nm, 5 μL injection): *N.B. in enantioenriched spectrum, peak at 23.3 min corresponds to major enantiomer of the Z-isomer.*

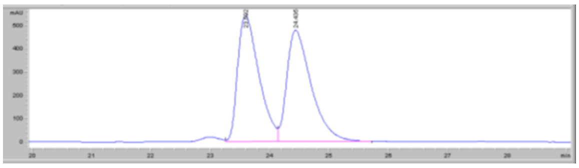

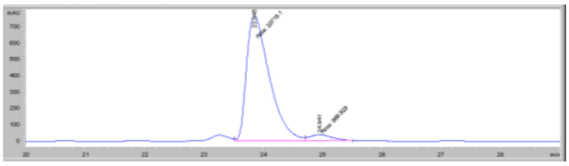

RetTime [min]	Type	Width [min]	Area [mAU*s]	Height [mAU]	Area %
23.592	VV	0.3822	1.34740e4	541.63336	49.5715
24.435	VB	0.4363	1.37069e4	478.33783	50.4285

RetTime [min]	Type	Width [min]	Area [mAU*s]	Height [mAU]	Area %
23.845	FM	0.4482	2.07161e4	770.39923	95.5274
24.941	FM	0.4394	969.92877	36.79175	4.4726

(*R*,*Z*)-1-(3,4-Dimethyl-7-phenylhept-4-en-1-yl)-4-methoxybenzene, 24a

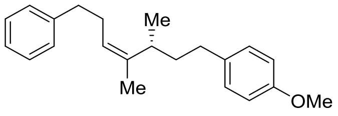

24a

A stirred solution of **23** (30 mg, 0.13 mmol) in THF (0.50 mL) was cooled to -78 °C and a solution of *tert*-butyllithium (1.7 M in pentane, 0.16 mL, 0.27 mmol) was added dropwise. The resulting solution was stirred at -78 °C for 30 minutes and then a solution of **2**[13] (19 mg, 0.065 mmol) in THF (0.5 mL) was added dropwise. The resulting solution was stirred at -78 °C for 30 minutes and then a suspension of sodium methoxide (3.0 M in MeOH, 0.07 mL, 0.20 mmol) was added in a single portion followed by dropwise addition of a solution of iodine (0.5 M in MeOH, 0.16 mL, 0.08 mmol). The resulting mixture was warmed to 0 °C and stirred for 30 minutes and then saturated aqueous sodium sulfite was added followed by water and dichloromethane. The organic layer was separated and the aqueous layer was

extracted twice with dichloromethane. The combined organic extracts were dried over anhydrous magnesium sulfate, filtered and concentrated under reduced pressure. The *Z*/*E* ratio of the product was determined to be >98:2 by ^{1}H NMR analysis of the crude reaction mixture. Purification of the residue *via* column chromatography eluting with a gradient from pentane to 99:1 pentane/diethyl ether afforded the title compound **24a** as a colourless oil (19 mg, 94 %, >98:2 *Z*/*E*, 95.6:4.4 e.r.). The *E*/*Z* selectivity was confirmed by nOe (see NMR spectra).

^{1}H NMR (400 MHz, $CDCl_3$) δ_H = 7.29 – 7.24 (m, 2H), 7.20 – 7.13 (m, 3H), 7.06 (d, *J* = 8.7 Hz, 2H), 6.80 (d, *J* = 8.7 Hz, 2H), 5.20 (tq, *J* = 7.2, 1.5 Hz, 1H), 3.77 (s, 3H), 2.68 – 2.56 (m, 3H), 2.48 – 2.33 (m, 2H), 2.31 – 2.22 (m, 2H), 1.62 – 1.52 (m, 5H), 0.92 (d, *J* = 6.9 Hz, 3H);

^{13}C NMR (101 MHz, $CDCl_3$) δ_C = 157.7, 142.4, 139.5, 135.0, 129.2, 128.5, 128.3, 125.8, 124.8, 113.8, 55.3, 36.9, 36.5, 33.6, 33.2, 29.4, 19.2, 18.1;

FTIR (neat) ν/cm^{-1} = 2957, 2930, 1611, 1511, 1454, 1245, 1177, 1038, 699;

HRMS (EI^+): calculated for $C_{22}H_{28}O$ = 308.2140, mass found = 308.2147;

$[\alpha]_D^{25}$ = +47 (*c* = 0.85, $CHCl_3$);

Chiral HPLC (Chiralpak IB with guard), 0.1 % IPA, 99.9 % hexane, 0.5 mL/min, 5 °C, λ = 210.8 nm, 5 µL injection): *N.B. in enantioenriched spectrum, peak at 9.6 min corresponds to major enantiomer of the E-isomer (c.a. 0.8 %).*

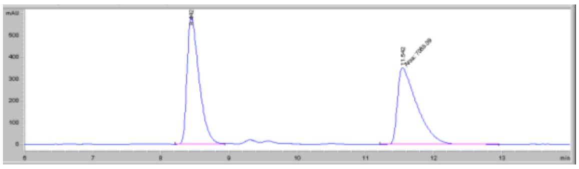

RetTime [min]	Type	Width [min]	Area [mAU*s]	Height [mAU]	Area %
8.442	VV	0.1848	7210.26563	589.27594	50.4378
11.542	MM	0.3354	7085.08984	352.04602	49.5622

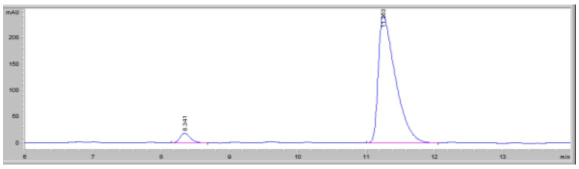

RetTime [min]	Type	Width [min]	Area [mAU*s]	Height [mAU]	Area %
8.341	BB	0.1646	201.92857	18.60212	4.5531
11.253	BB	0.2572	4233.00195	242.98067	95.4469

(*R*,*E*)-1-(3,4-Dimethyl-7-phenylhept-4-en-1-yl)-4-methoxybenzene, 24b

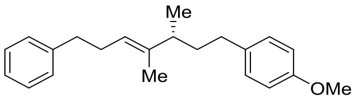

24b

A stirred solution of **23** (30 mg, 0.13 mmol) in THF (0.50 mL) was cooled to -78 °C and a solution of *tert*-butyllithium (1.7 M in pentane, 0.16 mL, 0.27 mmol) was added dropwise. The resulting solution was stirred at -78 °C for 30 minutes and then a solution of **2**[13] (19 mg, 0.067 mmol) in THF (0.5 mL) was added dropwise. The resulting solution was stirred at -78 °C for 30 minutes and then 2,2,2-trifluoroethanol (1.0 mL) was added dropwise. Freshly ground phenylselenyl chloride (15 mg, 0.080 mmol) was added in a single portion and the resulting mixture was stirred rapidly at -78 °C and then warmed to room temperature and stirred for 15 minutes and then concentrated under reduced pressure. The residue was filtered through a short plug of silica gel, washing with diethyl ether. The filtrate was concentrated under reduced pressure and the residue was dissolved in THF (0.67 mL). The resulting stirred solution was cooled to -78 °C and a solution of *m*CPBA (0.2 M in THF, 0.67 mL, 0.13 mmol) was added dropwise. The resulting solution was warmed to -45 °C (acetonitrile/dry ice bath) and stirred for 30 minutes. After this time dimethylsulfide (0.10 mL, 1.3 mmol) was added and the mixture was allowed to warm to room temperature and then filtered through a short plug of silica gel washing with diethyl ether. The filtrate was concentrated under reduced pressure. The *E*/*Z* ratio of the product was determined to be 98:2 by ^{1}H NMR analysis of the crude reaction mixture. Purification of the residue *via* column chromatography eluting with a gradient from pentane to 99:1 pentane/diethyl ether afforded the title compound **24b** as a colourless oil (19 mg, 94 %, 98:2 *E*/*Z*, 95.3:4.7 e.r.). The *E*/*Z* selectivity was confirmed by nOe (see NMR spectra).

^{1}H NMR (400 MHz, $CDCl_3$) δ_H = 7.30 – 7.24 (m, 2H), 7.22 – 7.14 (m, 3H), 7.05 (d, J = 8.7 Hz, 2H), 6.81 (d, J = 8.6 Hz, 2H), 5.21 (t, J = 7.2 Hz, 1H), 3.78 (s, 3H), 2.67 (t, J = 7.7 Hz, 2H), 2.46 – 2.25 (m, 4H), 2.16 – 2.04 (m, 1H), 1.67 – 1.42 (m, 5H), 0.98 (d, J = 6.9 Hz, 3H);

^{13}C NMR (101 MHz, $CDCl_3$) δ_C = 157.6, 142.4, 139.6, 135.2, 129.3, 128.6, 128.3, 125.7, 123.6, 113.7, 55.3, 42.5, 37.1, 36.2, 33.1, 29.8, 20.0, 12.2;

FTIR (neat) ν/cm^{-1} = 2926, 1611, 1511, 1454, 1244, 1176, 1038, 822, 747, 698;

HRMS (EI$^+$): calculated for $C_{22}H_{28}O$ = 308.2140, mass found = 308.2150;

$[\alpha]_D^{25}$ = +1 (*c* = 0.85, $CHCl_3$);

Chiral HPLC (Chiralpak IB with guard), 0.1 % IPA, 99.9 % hexane, 0.5 mL/min, 5 °C, λ = 210.8 nm, 5 µL injection): *N.B. in enantioenriched spectrum, peak at 12.0 min corresponds to major enantiomer of the Z-isomer (c.a. 2 %).*

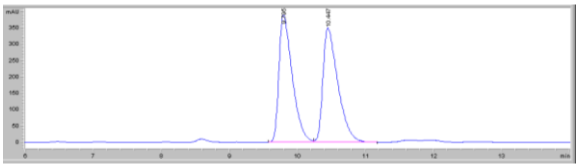

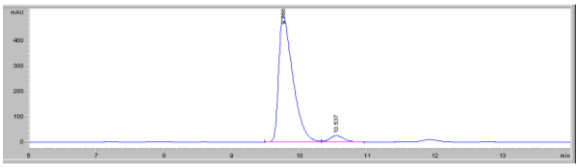

RetTime [min]	Type	Width [min]	Area [mAU*s]	Height [mAU]	Area %
9.795	BV	0.2051	5318.28271	390.06207	49.8374
10.447	VB	0.2309	5352.98584	349.13498	50.1626

RetTime [min]	Type	Width [min]	Area [mAU*s]	Height [mAU]	Area %
9.756	BV	0.2146	7214.98389	499.30853	95.2827
10.537	VB	0.2181	357.20233	25.09091	4.7173

(*R*,*E*)-*tert*-Butyl((6-(4-methoxyphenyl)-3,4-dimethylhex-2-en-1-yl)oxy)diphenylsilane, 26a

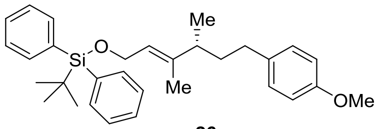

26a

A stirred solution of **25** (50 mg, 0.13 mmol) and **2** (19 mg, 0.06 mmol) in THF (1.0 mL) was cooled to -78 °C and a solution of *tert*-butyllithium (1.7 M in pentane, 0.15 mL, 0.26 mmol) was added dropwise over 5 minutes. The resulting solution was stirred at -78 °C for 90 minutes and was then allowed to warm to 0 °C. A suspension of sodium methoxide (3.0 M in MeOH, 0.06 mL, 0.18 mmol) was added in a single portion followed by dropwise addition of a solution of iodine (0.5 M in MeOH, 0.15 mL, 0.08 mmol). The resulting mixture was stirred at 0 °C for 30 minutes and then saturated aqueous sodium sulfite was added followed by water and dichloromethane. The organic layer was

separated and the aqueous layer was extracted twice with dichloromethane. The combined organic extracts were dried over anhydrous magnesium sulfate, filtered and concentrated under reduced pressure. Purification of the residue *via* column chromatography eluting with a gradient from pentane to 1:9 toluene/pentane afforded the corresponding alkene as a colourless oil (19 mg, 61 %, 96:4 *E/Z*, 95.3:4.7 e.r.). The *E/Z* selectivity was confirmed by nOe (see NMR spectra).

^{1}H NMR (400 MHz, $CDCl_3$) δ_H = 7.76 – 7.64 (m, 4H), 7.47 – 7.30 (m, 6H), 7.11 – 7.04 (m, 2H), 6.85 – 6.77 (m, 2H), 5.42 (t, *J* = 6.2 Hz, 1H), 4.31 – 4.21 (m, 2H), 3.79 (s, 3H), 2.49-2.39 (m, 2H), 2.15 – 2.07 (m, 1H), 1.70 – 1.55 (m, 1H), 1.57 – 1.44 (m, 1H), 1.37 (s, 3H), 1.05 (s, 9H), 0.98 (d, *J* = 6.9 Hz, 3H).

^{13}C NMR (101 MHz, $CDCl_3$) δ_C = 157.6, 140.4, 135.6, 134.9, 134.1, 129.5, 129.2, 127.6, 124.2, 113.6, 61.1, 55.3, 42.0, 36.8, 32.9, 26.8, 19.6, 19.2, 12.5;

FTIR (neat) ν/cm^{-1} = 2929, 2856, 1611, 1511, 1462, 1428, 1245, 1176, 1110, 1039, 822, 739;

HRMS (ESI$^+$): calculated for $C_{31}H_{40}NaO_2Si^+$ = 495.2690, mass found = 495.2670;

$[\alpha]_D^{25}$ = -3 (*c* = 0.60, $CHCl_3$);

Chiral HPLC (Chiralpak IB with guard), 0.2 % IPA, 99.8 % hexane, 1.0 mL/min, 25 °C, λ = 210.8 nm, 5 µL injection): *N.B. in enantioenriched spectrum, peak at 7.9 min corresponds to major enantiomer of the Z-isomer (c.a. 4 %).*

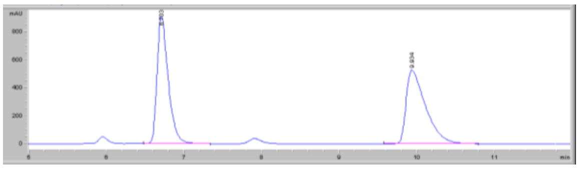

RetTime [min]	Type	Width [min]	Area [mAU*s]	Height [mAU]	Area %
6.703	VB	0.1527	9110.77734	910.75439	49.8555
9.934	PB	0.2593	9163.57813	525.94330	50.1445

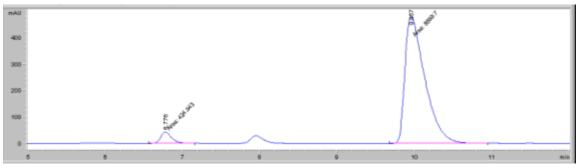

RetTime [min]	Type	Width [min]	Area [mAU*s]	Height [mAU]	Area %
6.776	MM	0.1620	424.54321	43.67380	4.6734
9.957	MF	0.2981	8659.69531	484.14630	95.3266

(*R*,*Z*)-*tert*-Butyl((6-(4-methoxyphenyl)-3,4-dimethylhex-2-en-1-yl)oxy)diphenylsilane, 26b

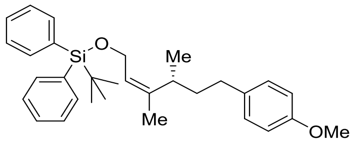

26b

A stirred solution of **25** (50 mg, 0.13 mmol) and **2** (19 mg, 0.06 mmol) in THF (1.0 mL) was cooled to -78 °C and a solution of *tert*-butyllithium (1.7 M in pentane, 0.15 mL, 0.26 mmol) was added dropwise over 5 minutes. The resulting solution was stirred at -78 °C for 90 minutes and then 2,2,2-trifluoroethanol (1.0 mL) was added dropwise. Freshly ground phenylselenyl chloride (15 mg, 0.07 mmol) was added in a single portion and the resulting mixture was stirred rapidly at -78 °C and then warmed to room temperature and stirred for 15 minutes and then concentrated under reduced pressure. The residue was filtered through a short plug of silica gel washing with diethyl ether. The filtrate was concentrated under reduced pressure and the residue was dissolved in THF (0.6 mL). The resulting stirred solution was cooled to -78 °C and a solution of *m*CPBA (0.2 M in THF, 0.6 mL, 0.13 mmol) was added dropwise. The resulting solution was warmed to -45 °C (acetonitrile/dry ice bath) and stirred for 30 minutes. After this time dimethylsulfide (0.10 mL, 1.3 mmol) was added and the mixture was allowed to warm to room temperature and then filtered through a short plug of silica gel washing with diethyl ether. The filtrate was concentrated under reduced pressure. Purification of the residue *via* column chromatography eluting with a gradient from pentane to 1:9 toluene/pentane afforded the title compound **26b** as a colourless oil (17 mg, 55 %, 96:4 *Z*/*E*, 95.3:4.7 e.r.). The *Z*/*E* selectivity was confirmed by nOe (see NMR spectra).

^{1}H NMR (400 MHz, $CDCl_3$) δ_H = 7.73 – 7.61 (m, 4H), 7.44 – 7.32 (m, 6H), 7.05 – 6.96 (m, 2H), 6.79 – 6.68 (m, 2H), 5.42 (td, *J* = 6.2, 1.5 Hz, 1H), 4.18 (d, *J* = 6.2 Hz, 2H), 3.75 (s, 3H), 2.47 – 2.22 (m, 3H), 1.63 (d, *J* = 1.4 Hz, 3H), 1.58 – 1.45 (m, 2H), 1.04 (s, 9H), 0.90 (d, *J* = 6.8 Hz, 3H).

^{13}C NMR (101 MHz, $CDCl_3$) δ_C = 157.6, 140.8, 135.6, 134.7, 134.0, 129.5, 129.1, 127.6, 125.4, 113.6, 60.3, 55.2, 36.7, 34.0, 33.0, 26.8, 19.2, 19.2, 17.8.

FTIR (neat) ν/cm^{-1} = 2930, 2856, 1612, 1511, 1462, 1428, 1244, 1176, 1111, 1040, 823, 740, 702;

HRMS (ESI$^+$): calculated for $C_{31}H_{40}NaO_2Si^+$ = 495.2690, mass found = 495.2703;

$[\alpha]_D^{25}$ = + 32 (*c* = 0.60, $CHCl_3$);

Chiral HPLC (Chiralpak IB with guard, 0.2 % IPA, 99.8 % hexane, 1.0 mL/min, 25 °C, λ = 210.8 nm, 5 μL injection): *n.b. in enantioenriched spectrum, peak at 10.0 min corresponds to major enantiomer of the E-isomer (c.a. 4 %).*

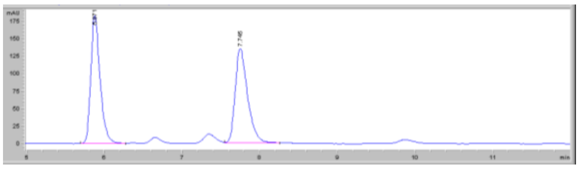

RetTime [min]	Type	Width [min]	Area [mAU*s]	Height [mAU]	Area %
5.871	BB	0.1253	1521.01892	182.33713	50.1650
7.745	VB	0.1686	1511.01074	134.99991	49.8350

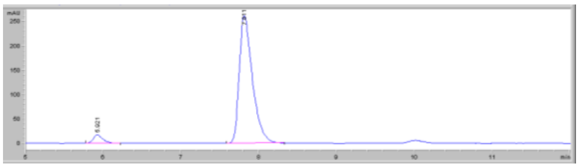

RetTime [min]	Type	Width [min]	Area [mAU*s]	Height [mAU]	Area %
5.921	BB	0.1301	151.11208	17.26782	4.6785
7.811	VB	0.1771	3078.79517	262.01620	95.3215

(5S,6S)-5-((2R,6S)-7-((4-methoxybenzyl)oxy)-4,6-dimethyl-3-(phenylselanyl)-4-(4,4,5,5-tetramethyl-1,3,2-dioxaborolan-2-yl)heptan-2-yl)-2,2,3,3,6,10,10-heptamethyl-9,9-diphenyl-4,8-dioxa-3,9-disilaundecane, 39

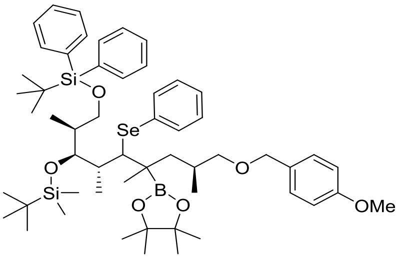

39

A stirred solution of (*R*)-1-((3-bromo-2-methylpropoxy)methyl)-4-methoxybenzene[28] (35.4 mg, 0.13 mmol) in diethyl ether (0.5 mL) was cooled to -78 °C and a solution of *tert*-butyllithium (1.7 M in pentane, 0.15 mL, 0.26 mmol) was added dropwise. The resulting solution was stirred at -78 °C for 15 minutes and then a solution of boronic ester **27** (64 mg, 0.10 mmol) in THF (0.5 mL) was added

dropwise. The resulting solution was stirred at -78 °C for 30 minutes and then 2,2,2-trifluoroethanol (1.0 mL) was added dropwise. Freshly ground phenylselenyl chloride (23 mg, 0.12 mmol) was added in a single portion and the resulting mixture was stirred rapidly at -78 °C and then warmed to room temperature and stirred for 15 minutes and then concentrated under reduced pressure. The residue was filtered through a short plug of silica gel washing with diethyl ether. The filtrate was concentrated under reduced pressure. Purification of the residue *via* column chromatography eluting with 94:6 pentane/diethyl ether afforded the title compound **39** as an off white gummy foam (51 mg, 52 %, 73:27 d.r.).

^{1}H NMR (400 MHz, $CDCl_3$) δ_H = 7.72-7.62 (m, 6H_{maj} + 4H_{min}), 7.57-7.54 (m, 2H_{min}), 7.45-7.16 (11H_{maj} + 11H_{min}), 6.91-6.85 (m, 2H_{maj} + 2H_{min}), 4.51-4.36 (m, 2H_{maj} + 2H_{min}), 4.04-3.99 (m, 1H_{min}), 3.86-3.78 (m, 4H_{maj} + 4H_{min}), 3.53-3.38 (m, 3H_{maj} + 1H_{min}), 3.35 (dd ,J = 10.0, 8.8 Hz, 1H_{maj}), 3.12 (t, J = 8.7 Hz, 1H_{min}), 3.06-2.99 (m, 1H_{maj} + 2H_{min}), 2.37-2.28 (m, 1H_{maj}), 2.25-2.16 (m, 1H_{min}), 2.01-1.90 (m, 2H_{min}), 1.90-1.82 (m, 1H_{maj}), 1.77-1.65 (m, 2H_{maj}), 1.44-1.00 (28H_{maj} + 29H_{min}), 1.03 (d, J = 6.4 Hz, 3H_{maj}), 0.98 (d, J = 6.9 Hz, 3H_{min}), 0.91 (d, J = 7.1 Hz, 3H_{min}), 0.75 (s, 9H_{min}), 0.72 (s, 9H_{maj}), 0.56 (d, J = 6.9 Hz, 3H_{maj}), -0.01 (s, 3H_{maj}), -0.20 (s, 3H_{min}), -0.31 (s, 3H_{min}), -0.32 (s, 3H_{maj});

^{13}C NMR (101 MHz, $CDCl_3$) δ_C = 159.0 (maj), 159.0 (min), 135.7 (min), 135.7 (min), 135.6 (maj), 135.6 (maj), 134.1 (min), 134.1 (maj), 134.1 (min), 134.0 (maj), 133.1 (maj), 133.0 (min), 132.6 (maj + min), 131.0 (min), 130.9 (maj), 129.4 (maj), 129.4 (min), 129.2 (maj), 129.1 (min), 128.9 (maj), 128.8 (min), 127.6 (min), 127.5 (maj), 126.4 (maj), 126.3 (min), 113.7 (maj + min), 83.8 (maj), 83.2 (min), 77.8 (maj), 77.2 (min), 76.9 (min), 76.5 (maj), 72.6 (maj), 72.6 (min), 65.9 (min), 65.8 (min), 65.3 (maj), 61.5 (maj), 55.3 (maj + min), 43.2 (maj + min), 41.4 (min), 39.0 (maj), 37.9 (maj), 32.2 (maj), 30.6 (min), 30.3 (min), 27.0 (maj), 27.0 (min), 26.2 (maj), 26.2 (min), 25.5 (maj), 25.1 (min), 25.0 (maj), 24.9 (min), 20.7 (maj), 20.5 (maj), 20.4 (min), 19.3 (maj), 19.3 (min), 18.5 (min), 18.4 (maj), 17.0 (maj), 16.7 (maj + min), 15.3 (min), 14.7 (min), -3.2 (maj), -3.5 (min), -4.0 (maj), -4.6 (min). [*N.B. The carbon attached to boron was not observed due to quadrupolar relaxation*];

^{11}B NMR (96 MHz, $CDCl_3$) δ_B = 31.1;

FTIR (neat) ν/cm^{-1} = 2957, 2930, 2856, 1612, 1579, 1513, 1472, 1462, 1388, 1371, 1360, 1313, 1248, 1139, 1110, 1037, 836;

LRMS (Nanospray$^+$): calculated for $C_{55}H_{83}BO_6SeSi_2Na^+$ = 1010, mass found = 1010.

(5*R*,6*S*)-5-((2*S*,6*S*,*Z*)-7-((4-methoxybenzyl)oxy)-4,6-dimethylhept-3-en-2-yl)-2,2,3,3,6,10,10-heptamethyl-9,9-diphenyl-4,8-dioxa-3,9-disilaundecane, 28a

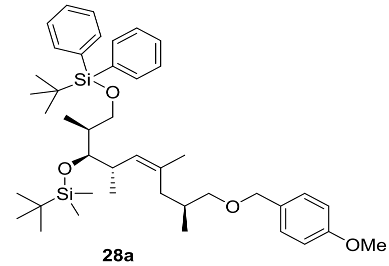

28a

A stirred solution of **39** (26.0 mg, 0.027 mmol, 73:27 d.r.) in THF (0.27 mL) was cooled to 0 °C and a suspension of sodium methoxide (3.0 M in MeOH, 0.18 mL, 0.53 mmol) was added dropwise and the resulting solution was stirred at 0 °C for 1 hour and then warmed to room temperature and stirred for 2 hours. After this time, saturated aqueous ammonium chloride was added followed by water and dichloromethane. The organic layer was separated and the aqueous layer was extracted twice with dichloromethane. The combined organic extracts were dried over anhydrous magnesium sulfate, filtered and concentrated under reduced pressure. The *Z*/*E* ratio of the product was determined to be 98:2 by ^{1}H NMR analysis of the crude reaction mixture. Purification of the residue *via* column chromatography eluting with 3:97 diethyl ether/pentane afforded the title compound **28a** as a colourless oil (17.0 mg, 92 % yield, 98:2 *Z*/*E*, >95:5 d.r.). The *Z*/*E* selectivity was confirmed by nOe (see NMR spectra).

^{1}H NMR (400 MHz, $CDCl_3$) δ_H = 7.66-7.62 (m, 4H), 7.44 – 7.29 (m, 6H), 7.24 (d, *J* = 8.3 Hz, 2H), 6.86 (d, *J* = 8.5 Hz, 2H), 5.04 (d, *J* = 9.8 Hz, 1H), 4.41 (s, 2H), 3.79 (s, 3H), 3.72 (dd, J = 9.8, 5.8 Hz, 1H), 3.44 (t, *J*

= 5.3 Hz, 1H), 3.37 (dd, J = 9.4, 8.7 Hz, 1H), 3.23 (dd, J = 8.9, 4.9 Hz, 1H), 3.17 (dd, J = 8.7, 5.8 Hz, 1H), 2.57 – 2.41 (m, 1H), 2.02 – 1.85 (m, 4H), 1.56 (d, J = 1.3 Hz, 3H), 1.05 (s, 9H), 0.91 (d, J = 6.9 Hz, 3H), 0.87 (d, J = 6.7 Hz, 3H), 0.85 – 0.79 (m, 12H), -0.01 (s, 3H), -0.08 (s, 3H);

^{13}C NMR (101 MHz, $CDCl_3$) δ_C = 159.1, 135.7, 134.2, 134.2, 131.6, 131.5, 130.9, 129.5, 129.2, 127.6, 113.8, 77.9, 76.2, 72.7, 66.7, 55.3, 41.6, 36.1, 35.3, 31.8, 27.0, 26.2, 23.5, 19.4, 18.4, 17.2, 16.8, 13.6, -3.9, -3.9;

FTIR (neat) ν/cm^{-1} = 2957, 2930, 5856, 1613, 1513, 1462, 1248, 1080, 1110, 1038, 835, 824, 773, 702;

HRMS (ESI$^+$): calculated for $C_{43}H_{66}NaO_4Si_2^+$ = 725.4392, mass found = 725.4387;

$[\alpha]_D^{25}$ = +8 (c = 0.50, $CHCl_3$).

<u>Alternative prepatation of **28a** with (*R*)-1-((3-iodo-2-methylpropoxy)methyl)-4-methoxybenzene:</u>

A stirred solution of (*R*)-1-((3-iodo-2-methylpropoxy)methyl)-4-methoxybenzene[29] (42 mg, 0.13 mmol) in diethyl ether (0.50 mL) was cooled to -100 °C (ethanol/N_2 bath) and a solution of *tert*-butyllithium (1.7 M in pentane, 0.15 mL, 0.26 mmol) was added dropwise. The resulting solution was stirred at -100 °C for 15 minutes and then a solution of **27** (64 mg, 0.10 mmol) in THF (0.5 mL) was added dropwise. The resulting solution was warmed to -78 °C and stirred for 30 minutes. After this time, a suspension of sodium methoxide (3 M in MeOH, 0.10 mL, 0.30 mmol) was added in a single portion followed by dropwise addition of a solution of iodine (0.5 M in MeOH, 0.24 mL, 0.12 mmol). The resulting mixture was warmed to 0 °C and stirred for 30 minutes and then saturated aqueous sodium sulfite was added followed by water and dichloromethane. The organic layer was separated and the aqueous layer was extracted twice with dichloromethane. The combined organic extracts were dried over anhydrous magnesium sulfate, filtered and concentrated under reduced pressure. Analysis of the crude residue by 1H NMR, employing 1,1,2,2-tetrachloroethane (0.05 mmol) as an internal standard indicated that **28a** was formed in 52 % NMR yield (~95:5 *Z*/*E*, >95:5 d.r.). The product could not be separated from side products. The spectral data for **28a** matched that described above.

Alternative prepatation of **28a** with (*R*)-1-((3-bromo-2-methylpropoxy)methyl)-4-methoxybenzene:

A stirred solution of (*R*)-1-((3-bromo-2-methylpropoxy)methyl)-4-methoxybenzene[28] (35.4 mg, 0.13 mmol) in diethyl ether (0.50 mL) was cooled to -78 °C and a solution of *tert*-butyllithium (1.7 M in pentane, 0.15 mL, 0.26 mmol) was added dropwise. The resulting solution was stirred at -78 °C for 15 minutes and then a solution of **27** (64 mg, 0.10 mmol) in THF (0.5 mL) was added dropwise and the resulting solution was stirred at -78 °C for 30 minutes. After this time, a suspension of sodium methoxide (3 M in MeOH, 0.10 mL, 0.30 mmol) was added in a single portion followed by dropwise addition of a solution of iodine (0.5 M in MeOH, 0.24 mL, 0.12 mmol). The resulting mixture was warmed to 0 °C and stirred for 30 minutes and then saturated aqueous sodium sulfite was added followed by water and dichloromethane. The organic layer was separated and the aqueous layer was extracted twice with dichloromethane. The combined organic extracts were dried over anhydrous magnesium sulfate, filtered and concentrated under reduced pressure. Analysis of the crude residue by 1H NMR, employing 1,1,2,2-tetrachloroethane (0.05 mmol) as an internal standard indicated that **28a** was formed in 51 % NMR yield (~95:5 *Z*/*E*, >95:5 d.r.). The product could not be separated from side products. The spectral data for **28a** matched that described above.

(5*R*,6*S*)-5-((2*S*,6*S*,*E*)-7-((4-methoxybenzyl)oxy)-4,6-dimethylhept-3-en-2-yl)-2,2,3,3,6,10,10-heptamethyl-9,9-diphenyl-4,8-dioxa-3,9-disilaundecane, 28b

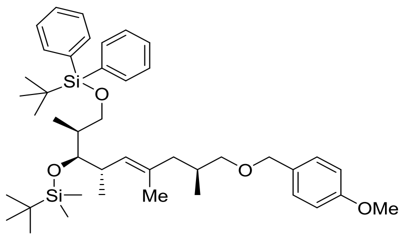

28b

A stirred solution of **39** (21.7 mg, 0.022 mmol, 73:27 d.r.) in THF (0.22 mL) was cooled to -78 °C and a solution of *m*CPBA (0.2 M in THF, 0.22 mL, 0.044 mmol) was added dropwise. The resulting solution was warmed to -45 °C (acetonitrile/dry ice bath) and stirred for 30 minutes. After this time

dimethylsulfide (32 µL, 0.44 mmol) was added and the mixture was allowed to warm to room temperature and then filtered through a short plug of silica gel, washing with diethyl ether. The filtrate was concentrated under reduced pressure. The *E*/*Z* ratio of the product was determined to be 98:2 by ^{1}H NMR analysis of the crude reaction mixture. Purification of the residue *via* column chromatography eluting with 3:97 diethyl ether/pentane afforded the title compound **28b** as a colourless oil (15.4 mg, quant, 98:2 *E*/*Z*, >95:5 d.r.). The *E*/*Z* selectivity was confirmed by nOe (see NMR spectra).

^{1}H NMR (400 MHz, $CDCl_3$) δ_H = 7.67 – 7.61 (m, 4H), 7.42 – 7.31 (m, 6H), 7.25 (d, *J* = 8.6 Hz, 2H), 6.87 (d, *J* = 8.6 Hz, 2H), 4.93 (d, *J* = 9.8 Hz, 1H), 4.40 (s, 2H), 3.79 (s, 3H), 3.73 (dd, *J* = 9.9, 5.8 Hz, 1H), 3.46 (dd, *J* = 6.8, 3.7 Hz, 1H), 3.40 (dd, *J* = 9.9, 8.3 Hz, 1H), 3.25 (dd, *J* = 9.0, 5.4 Hz, 1H), 3.13 (dd, *J* = 9.1, 6.9 Hz, 1H), 2.47 (dqn, *J* = 9.6, 6.7 Hz, 1H), 2.08 – 1.83 (m, 3H), 1.64 (dd, *J* = 13.1, 8.0 Hz, 1H), 1.51 (d, *J* = 1.4 Hz, 3H), 1.04 (s, 9H), 0.94 (d, *J* = 6.9 Hz, 3H), 0.85 (d, *J* = 6.6 Hz, 3H), 0.84 – 0.79 (m, 11H), -0.00 (s, 3H), -0.04 (s, 3H);

^{13}C NMR (101 MHz, $CDCl_3$) δ_C = 159.1, 135.7, 134.2, 134.1, 131.8, 131.0, 130.7, 129.5, 129.5, 129.2, 127.6, 113.8, 78.3, 75.4, 72.7, 66.1, 55.3, 44.4, 41.3, 36.3, 31.6, 27.0, 26.2, 19.3, 18.4, 17.8, 17.4, 16.0, 14.2, -3.8, -3.9;

FTIR (neat) ν/cm^{-1} = 2956, 2930, 2856, 1613, 1513, 1472, 1462, 1248, 1083, 1110, 1038, 835, 701;

HRMS (ESI$^+$): calculated for $C_{43}H_{66}NaO_4Si_2^+$ = 725.4392, mass found = 725.4374;

$[\alpha]_D^{25}$ = -10 (*c* = 0.5, $CHCl_3$).

2. Computational Investigations

Introduction: The two diastereomers of the β-selenoxy pinacol boronic ester that would give *trans*-2-butene upon *syn* elimination (**1** and **2**) were investigated computationally using density functional theory (DFT) to explore both the conformational landscape characterised by the Se–C–C–B dihedral angle and the three possible types of selenoxide elimination reactions, namely, Se–O–B elimination (*SE1*; to give **3** and **4**) and Se–O–H elimination to give either the allylic boronic ester (*SE2*; **5** and **6**) or the vinylic boronic ester (*SE3*, **7** and **6**). All DFT calculations were carried out for the systems in the gas phase using GAUSSIAN 09.[30]

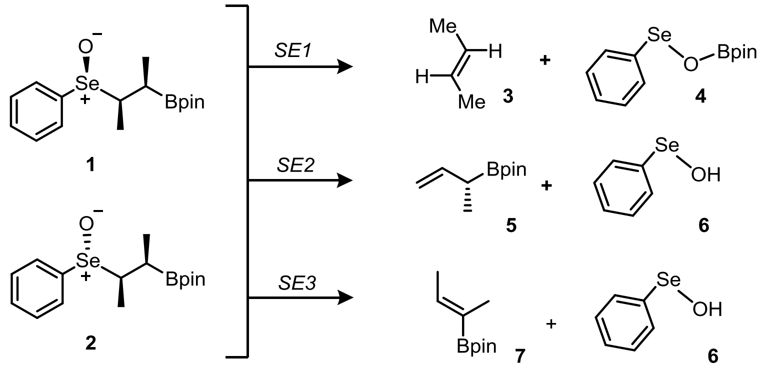

Scheme 1: Selenoxide elimination reactions investigated computationally.

Method: At the B3LYP[31]/6-31G* level of theory, a relaxed potential energy surface scan of the dihedral angle defined by the Se–C–C–B bond was carried out in steps of 18° for an optimized structure of **1** and **2**. The resulting structures representing minima and maxima on the energy surface were then optimised to a conformer or a transition state for conformer interconversion, respectively. Vibrational frequency calculations were carried out to confirm these stationary points. Transition-state-like structures for selenoxide elimination reactions were identified through a relaxed potential energy surface scan of the appropriate conformer through either elongating the C–B bond in increments of 0.1 Å (for *SE1* reactions) or reducing the pertinent (Se)O–H(C) distance in increments of 0.1 Å (for *SE2* and *SE3* reactions). The transition-state like structures were optimised and vibrational frequency calculations carried out. Connection of the resulting optimised transition-state structures to starting

material and products were confirmed through the Intrinsic Reaction Coordinate (IRC) method.[32] All conformer minima identified for **1** and **2**, products **3**–**7**, and transition-state structures were reoptimized using the B3LYP functional together with the following basis sets (as used by Bayse and co-workers):[33] selenium was represented by a relativistic effective core potential double-ζ basis set augmented with even-tempered s, p, and d diffuse functions (obtained from the Basis Set Exchange website[34] under CRENBL ECP);[35] boron and oxygen were represented by Dunning's split-valence triple-ζ plus polarization function basis set;[36] carbon and hydrogen were represented by Dunning's double-ζ plus polarization basis sets.[37] Reported energy values have been corrected for zero-point energy.

__Results__: (for diastereomer **1**)

A dihedral scan of diastereomer **1** gave four minima (**1A**, **1C**, **1E**, and **1G**) and three transition state structures for conformer interconversion (**1B**, **1D**, and **1F**) (Figure 1). Structures **1A** and **1G** have a similar Se–C–C–B dihedral angle but in structure **1G**, the oxygen atom that is attached to selenium atom is pointing away from the boron atom.

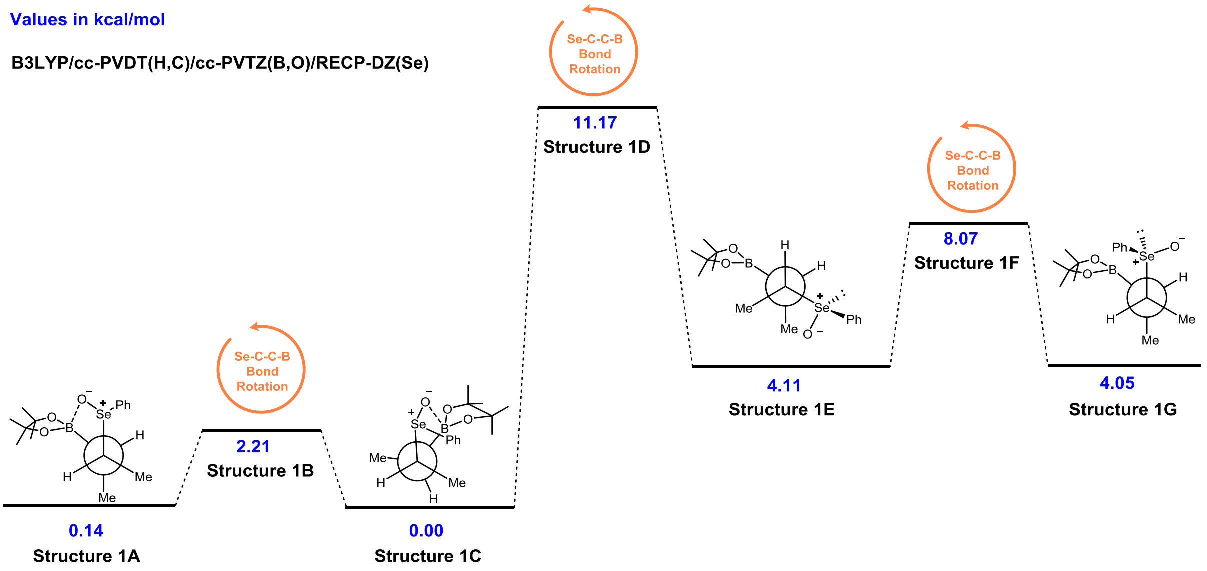

Figure 1: Conformational analysis of diastereomer **1**; all energy values (kcal mol^{-1}) given are ZPE corrected potential energies relative to that of structure **1C**.

Transition state structures **1A_TS_SE1** and **1C_TS_SE1** for selenoxide elimination *SE1* (to give **3** and **4**) were found from conformers **1A** and **1C**, showing barriers of 4.80 and 2.19 kcal mol^{-1}, respectively (Figure 2). IRC analysis confirmed the connection between these transition state structures and the conformers.

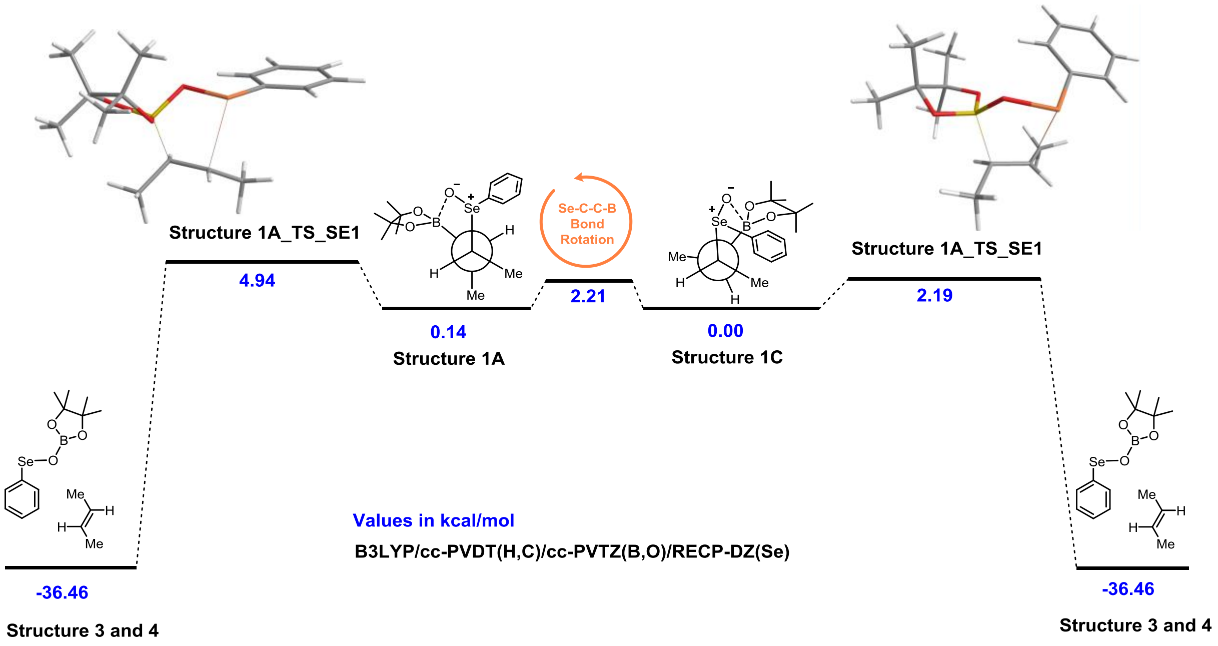

Figure 2: Fragmentation of structures **1A** and **1C** through selenoxide elimination *SE1*.

A transition state structure (**1E_TS_SE2**) for selenoxide elimination *SE2* (to give **5** and **6**) was found from structure **1E**; the transformation showing a barrier of 11.02 kcal mol^{-1}.

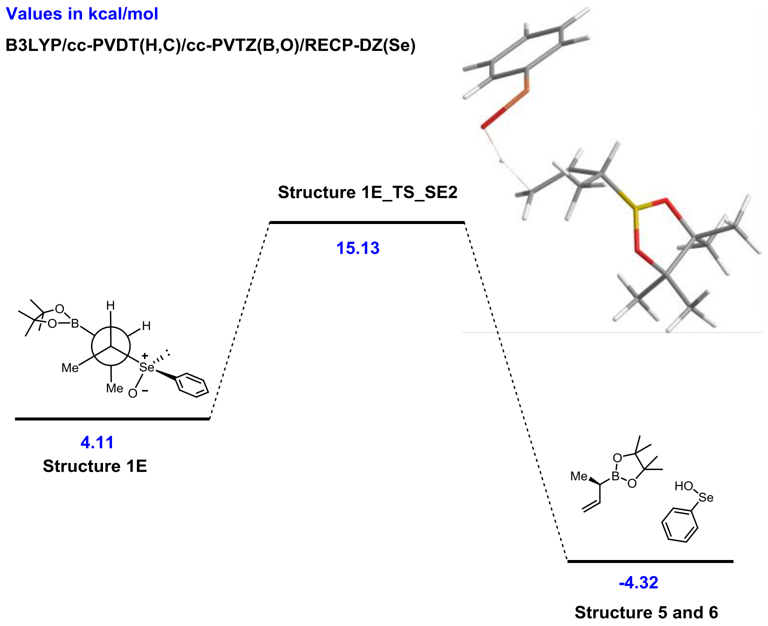

Figure 3: Fragmentation of structure **1E** through selenoxide elimination *SE2*.

A transition state structure (**1H_TS_SE3**) for selenoxide elimination *SE3* (to give **6** and **7**) was found from structure **1G**; however IRC analysis connected the transition state structure to structure **1H**, the transformation showing a barrier of 5.6 kcal mol^{-1}.

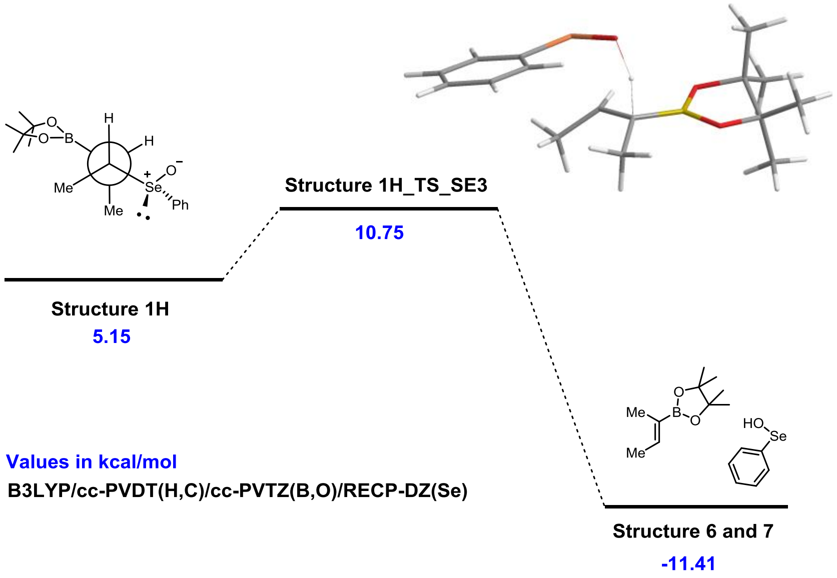

Figure 4: Fragmentation of structure **1H** through selenoxide elimination *SE3*.

<u>*Results*</u>: (for diastereomer **2**)

A dihedral scan of diastereomer **2** gave three minima (**2A**, **2B**, and **2D**) and two conformational transition state structures (**2C** and **2E**). A transition state could not be found linking **2A** and **2B** (Figure 5).

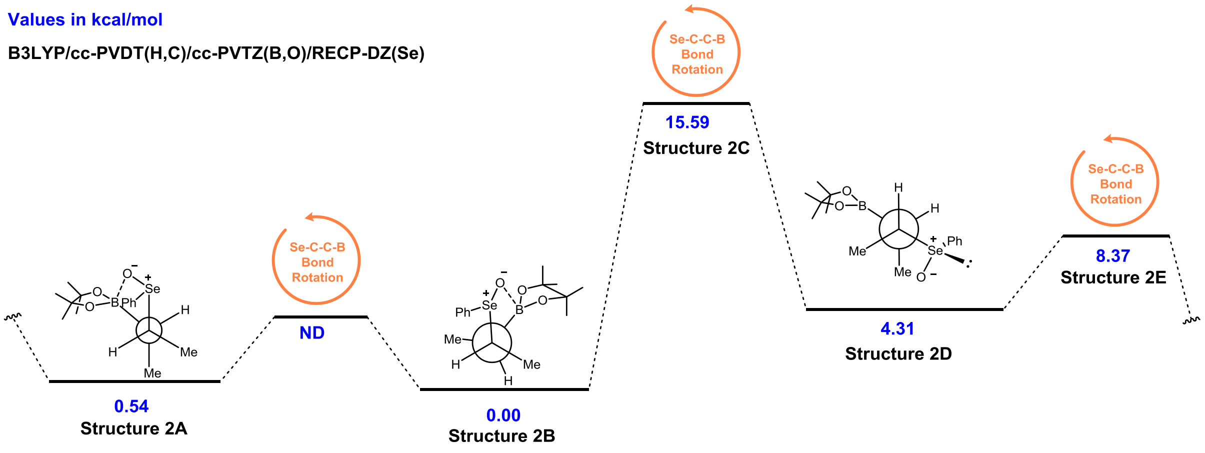

Figure 5: Conformational analysis of diastereomer **2**; a transition state could not be found for conversion of conformer **2A** into conformer **2B** through rotation about the Se–C–C–B dihedral. All energy values (kcal mol^{-1}) are relative to that of structure **2B**.

A transition state structure (**2A_TS_SE1**) for selenoxide elimination *SE1* (to give **3** and **4**) was found from structure **2B** (Figure 6), showing a very low barrier (0.35 kcal mol^{-1}). The same transition state structure was found from structure **2A**; however IRC analysis connected the transition state structure to structure **2B**.

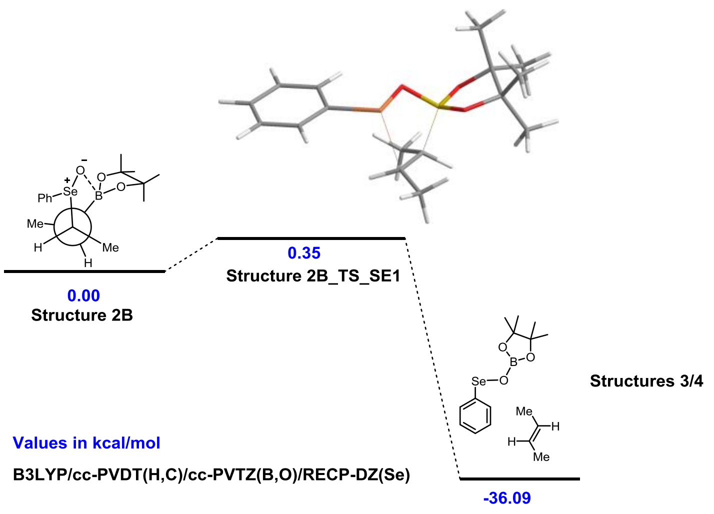

Figure 6: Fragmentation of structure **2B** through selenoxide elimination *SE1*.

A transition state structure (**2F_TS_SE2**) for selenoxide elimination *SE2* (to give **5** and **6**) was found from structure **2D**; however IRC analysis connected the transition state to new conformer **2F** (Figure 7), the transformation showing a barrier of 10 kcal mol^{-1}.

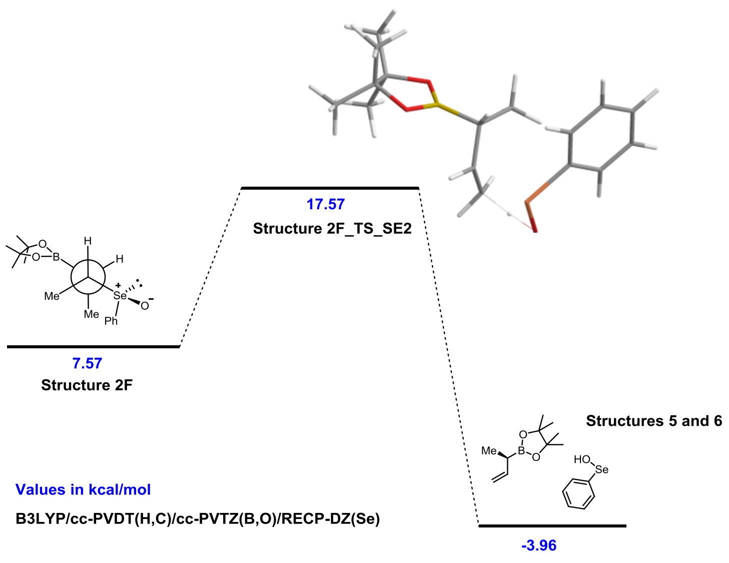

Figure 7: Fragmentation of structure **2F** through selenoxide elimination *SE2*.

A transition state structure (**2D_TS_SE3**) for selenoxide elimination *SE3* (to give **6** and **7**) was found from structure 2D (Figure 8); IRC analysis confirmed their connection, the transformation showing a barrier of 6.98 kcal mol^{-1}.

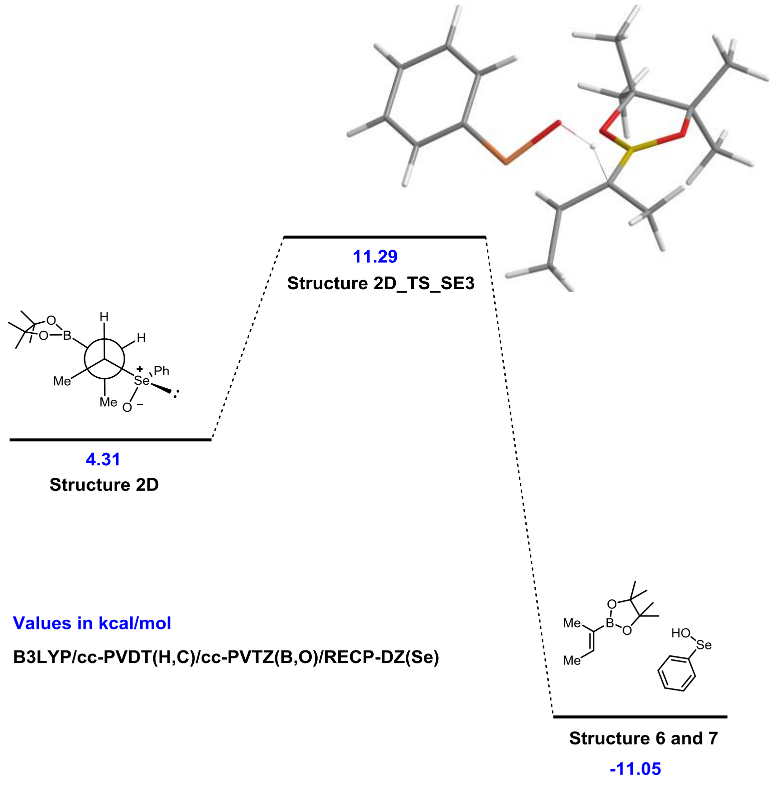

Figure 8: Fragmentation of structure **2D** through selenoxide elimination *SE3*.

Structure 1A

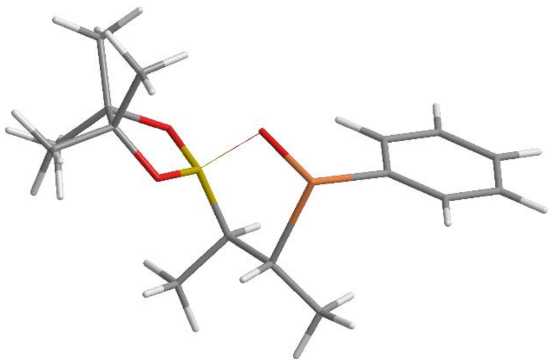

C	-0.68304800	-1.91334300	0.01954200
C	0.23087100	-1.30320400	1.06195600
B	1.15286700	-0.15160900	0.34413100
O	2.04989200	-0.68896400	-0.64207400
C	3.16861500	0.21169000	-0.75348900
C	3.23425200	0.85049100	0.69702400
O	1.87281200	0.76016600	1.16097600
C	3.66567700	2.31890600	0.73109000
C	4.10946900	0.04260800	1.66870400
C	4.40288100	-0.59975500	-1.15315000
C	2.86807900	1.24492100	-1.85375800
C	-2.76615300	0.41010000	-0.26611200
C	-2.64145600	1.30588500	0.79733300
C	-3.80118600	1.79692700	1.40424400
C	-5.06142200	1.39801700	0.94616500
C	-5.17104400	0.50971000	-0.12843100
C	-4.01838800	0.01540900	-0.74624200
C	-1.92905100	-2.65933500	0.46050100
C	1.09342200	-2.38726600	1.73158300
H	-0.10217000	-2.50474800	-0.70859000
H	-0.39562700	-0.82171500	1.83573700
H	3.67049900	2.67695600	1.77258300
H	4.68215900	2.44724300	0.32361300
H	2.97513200	2.95483200	0.16168200
H	5.18226900	0.13847100	1.43729700
H	3.94248100	0.41929700	2.68963100
H	3.84355800	-1.02386200	1.65335300
H	4.26132000	-1.01207200	-2.16470800
H	5.30717400	0.03048500	-1.16893200
H	4.57428500	-1.44162700	-0.46925800
H	3.72713800	1.91013700	-2.03558600
H	2.64984900	0.70740000	-2.79023800
H	1.99100500	1.85640600	-1.60299800
H	-1.64738000	1.61416600	1.12707000
H	-3.71755400	2.49787200	2.23761100
H	-5.96336200	1.78719700	1.42362600
H	-6.15441700	0.20541400	-0.49320800
H	-4.10490400	-0.66712800	-1.59629800
H	-2.55518000	-2.98867500	-0.38583000
H	-1.61522300	-3.56737200	1.00304000
H	-2.54733600	-2.05777100	1.14372700
H	1.73462000	-1.92591300	2.49825700
H	0.49232500	-3.16870000	2.22859600
H	1.75241100	-2.87424200	0.99522400
Se	-1.16078900	-0.25052000	-1.15709000
O	0.01608400	0.75794000	-0.41850000

Zero-point energy: 0.384666 Hartree
Electronic and zero point energy: -1009.251654 Hartree

Structure 1B

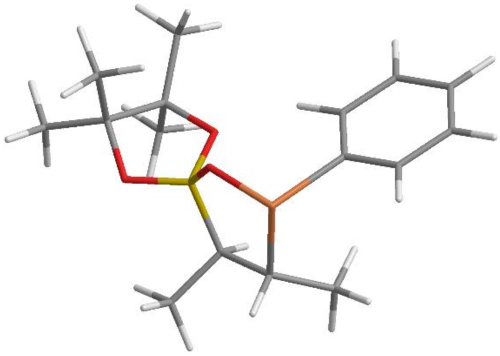

C	-0.90542900	-2.15168500	0.71219500
C	0.52161500	-1.72531300	1.01765600
B	1.14677600	-0.54058700	0.01625100
O	2.45364800	-0.81071700	-0.48426000
C	3.17873600	0.42626000	-0.58794800
C	2.52719700	1.28753400	0.56348900
O	1.17856100	0.78837100	0.59883000
C	2.49413500	2.79448800	0.30047800
C	3.16342700	1.02114300	1.93821200
C	4.66895800	0.13083900	-0.40758500
C	2.94166500	1.02008500	-1.98693800
C	-2.46556900	0.39971100	-0.32079400
C	-1.85764900	1.51784000	0.25701900
C	-2.67373100	2.56262400	0.70320200
C	-4.06455600	2.48823400	0.57251500
C	-4.65654000	1.36570600	-0.01447500
C	-3.85464700	0.31658300	-0.47272900
C	-1.95008100	-1.98201700	1.80208600
C	1.44154400	-2.95952200	1.06952400
H	-0.94829400	-3.17382600	0.29933700
H	0.50123800	-1.25221700	2.01457700
H	2.01724000	3.31078400	1.14916600
H	3.51249400	3.20202700	0.18922700
H	1.92195900	3.03321500	-0.60613300
H	4.17521000	1.44919500	2.01915200
H	2.53268000	1.47951600	2.71576800
H	3.22528700	-0.05781700	2.14427500
H	5.02881400	-0.48014200	-1.25009200
H	5.25994400	1.06132500	-0.38724800
H	4.86265600	-0.42826200	0.51766700
H	3.54403100	1.92706400	-2.15591500
H	3.22912200	0.27025400	-2.73978000
H	1.88132300	1.26202700	-2.14450700
H	-0.76929200	1.56121400	0.36192500
H	-2.21367400	3.44285600	1.15775000
H	-4.69074900	3.31157400	0.92354800
H	-5.74135400	1.30865200	-0.12685000
H	-4.31564300	-0.55226400	-0.95150500
H	-2.96583400	-2.25563400	1.47152400
H	-1.68409800	-2.64795900	2.64082400
H	-1.97375300	-0.95085900	2.18574000
H	2.45980200	-2.66834400	1.36417300
H	1.07917300	-3.72029600	1.78426400
H	1.52647900	-3.43425400	0.07783000
Se	-1.42693800	-1.09728400	-1.01657200
O	0.15189100	-0.48544300	-1.24556600

Zero-point energy: 0.384631 Hartree
Electronic and zero point energy: -1009.248351 Hartree
One imaginary frequency at: -37.21 cm^{-1}

Structure 1C

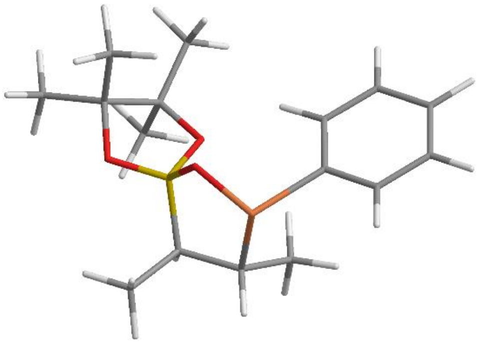

C	-0.71034600	-1.95186400	1.14203200
C	0.78275500	-1.93914700	0.89016000
B	1.20862700	-0.62660500	-0.02258700
O	2.50210800	-0.70941400	-0.61250000
C	3.08016800	0.60634200	-0.64893500
C	2.40499300	1.29769300	0.59926300
O	1.11406800	0.66098300	0.64427800
C	2.20946200	2.80964900	0.47221800
C	3.13480200	0.98483900	1.91636600
C	4.60140600	0.46933000	-0.56508500
C	2.69845400	1.27378900	-1.98093200
C	-2.48864000	0.26970900	-0.31360500
C	-1.90114300	1.50736800	-0.03925700
C	-2.73444700	2.59906000	0.22229500
C	-4.12567400	2.45149600	0.21042300
C	-4.69868800	1.20725100	-0.07006800
C	-3.87843800	0.10875200	-0.34346100
C	-1.22486500	-1.08683700	2.27823200
C	1.27654000	-3.25699900	0.27612800
H	-1.15574000	-2.96029900	1.17218800
H	1.26477300	-1.78374700	1.87184600
H	1.71699300	3.19943100	1.37760700
H	3.17639500	3.32842900	0.36578400
H	1.58254500	3.06572400	-0.39283900
H	4.09996200	1.51033100	1.99180900
H	2.50221000	1.30623900	2.75852000
H	3.31972000	-0.09450500	2.02102400
H	4.97774000	-0.02868800	-1.47220700
H	5.08747700	1.45635200	-0.49410800
H	4.90611500	-0.13583600	0.29925000
H	3.18566800	2.25425600	-2.10518200
H	3.02218900	0.62104300	-2.80586300
H	1.61026100	1.40328900	-2.06622600
H	-0.81247800	1.59717300	-0.01317200
H	-2.28975800	3.57263500	0.44015300
H	-4.76744700	3.31133800	0.41523900
H	-5.78445800	1.09124400	-0.08837100
H	-4.32626500	-0.85943400	-0.58553400
H	-2.32311900	-1.01385400	2.29693300
H	-0.90630100	-1.55273200	3.22707400
H	-0.79377500	-0.07663000	2.23722200
H	2.36293300	-3.22063400	0.11255200
H	1.04784500	-4.12853300	0.91517700
H	0.81927600	-3.44149500	-0.71237600
Se	-1.41031900	-1.29072700	-0.75162200
O	0.11218900	-0.63981300	-1.20341200

Zero-point energy: 0.384506 Hartree
Electronic and zero point energy: -1009.251876 Hartree

Structure 1D

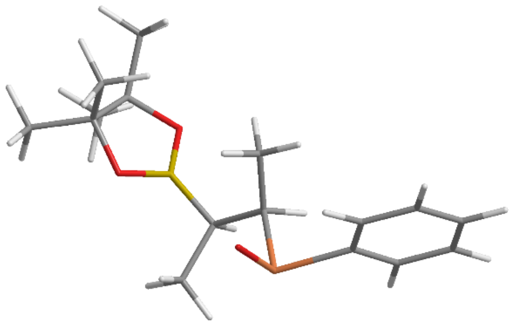

C	0.55288500	0.03220300	-0.65942400
C	-0.70847300	-0.71046500	-1.22760700
B	-2.07055300	-0.18773600	-0.61155200
O	-2.77904100	-0.86295000	0.34365300
C	-3.89788900	-0.02251100	0.76636300
C	-4.03591700	0.98639500	-0.44529800
O	-2.69488800	0.96037800	-1.02858000
C	-4.36819600	2.42446700	-0.05633500
C	-4.99436200	0.50267800	-1.53983900
C	-5.10991500	-0.91983800	1.00360700
C	-3.47490100	0.64590800	2.07933200
C	3.46217800	0.04688500	-0.03364800
C	3.94575300	0.54097600	1.17733700
C	5.02643300	1.42890700	1.17078600
C	5.61440300	1.80988000	-0.03997100
C	5.12789100	1.30040200	-1.24909000
C	4.04966300	0.40980800	-1.24846900
C	0.28341300	1.03623300	0.45419000
C	-0.66678900	-2.25370600	-1.16004700
H	1.09374900	0.52278900	-1.48532500
H	-0.75008900	-0.43588900	-2.29695800
H	-4.41306400	3.05094700	-0.96000200
H	-5.34899700	2.47710500	0.44265300
H	-3.61197700	2.85243700	0.61441300
H	-6.04302800	0.54313100	-1.20833400
H	-4.88558100	1.15185900	-2.42138200
H	-4.76649900	-0.52831200	-1.84885600
H	-4.90520500	-1.60029400	1.84369100
H	-5.99659400	-0.31877000	1.26104800
H	-5.34526500	-1.53219500	0.12338400
H	-4.28974700	1.25096000	2.50509800
H	-3.20723600	-0.13524400	2.80599500
H	-2.59552700	1.29113400	1.94111300
H	3.46594300	0.21351200	2.10403200
H	5.41219100	1.82312400	2.11409700
H	6.45970200	2.50197800	-0.04286500
H	5.59133500	1.59278800	-2.19430200
H	3.67900800	0.00294400	-2.19425100
H	1.19187800	1.59492300	0.72348000
H	-0.47630900	1.76418300	0.12797900
H	-0.07352400	0.52120600	1.36024200
H	-1.58868500	-2.68050300	-1.58500700
H	0.17757800	-2.67496000	-1.72872500
H	-0.59935900	-2.61277500	-0.12061600
Se	1.98940300	-1.26313300	0.04052200
O	1.68659200	-1.38495100	1.66594000

Zero-point energy: 0.385257 Hartree
Electronic and zero point energy: -1009.234082 Hartree
One imaginary frequency at: -38.67 cm^{-1}

Structure 1E

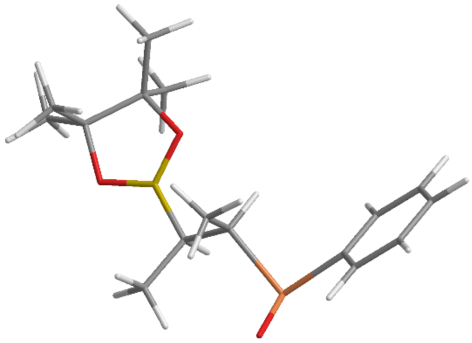

C	0.45091400	-0.48055300	0.15605600
C	-0.74997800	-1.23110000	-0.45411200
B	-2.09012700	-0.43163700	-0.20608600
O	-3.31421900	-1.02689700	-0.06460500
C	-4.28428100	0.00795500	0.29203400
C	-3.56134000	1.32694700	-0.19591400
O	-2.15177900	0.93778200	-0.16018100
C	-3.75069600	2.54280500	0.70672100
C	-3.87635800	1.69422000	-1.65059300
C	-5.60414600	-0.30521600	-0.40731100
C	-4.46199000	-0.06585600	1.81282200
C	3.29159200	0.36536600	-0.21265500
C	4.24639600	0.20001400	0.78969900
C	5.08482500	1.27053200	1.11731100
C	4.96501800	2.48864200	0.43998200
C	4.00927600	2.64040900	-0.57081000
C	3.16968500	1.57292800	-0.90473000
C	0.56885800	-0.55054100	1.67125700
C	-0.85321300	-2.71321000	-0.04478400
H	0.44146700	0.56534500	-0.19119100
H	-0.62261700	-1.19495000	-1.55744000
H	-3.19228300	3.39815600	0.29737600
H	-4.81381600	2.82643500	0.76183300
H	-3.38464500	2.35723200	1.72483500
H	-4.90844100	2.05947600	-1.76240000
H	-3.19145100	2.49245300	-1.97326200
H	-3.73652900	0.83495700	-2.32331900
H	-6.01307500	-1.24975500	-0.01820000
H	-6.34412700	0.48883100	-0.21848400
H	-5.47664900	-0.41476500	-1.49211100
H	-5.22711000	0.64177400	2.16636900
H	-4.77967300	-1.08310000	2.08546300
H	-3.52023800	0.14691800	2.34024900
H	4.31457600	-0.76952000	1.29132900
H	5.83551100	1.15285700	1.90261800
H	5.62247400	3.32284300	0.69582200
H	3.91981100	3.58992900	-1.10386000
H	2.43056100	1.69008200	-1.70297800
H	1.31596300	0.16556400	2.04477900
H	-0.39905500	-0.30514500	2.14124000
H	0.87604200	-1.55610900	1.99803500
H	-1.67780600	-3.20274600	-0.58398700
H	0.07565400	-3.26636100	-0.25432100
H	-1.06125700	-2.82225300	1.03091600
Se	2.17700500	-1.19733300	-0.67028200
O	2.72622500	-2.35847100	0.38114800

Zero-point energy: 0.384951 Hartree
Electronic and zero point energy: -1009.245324 Hartree

Structure 1F

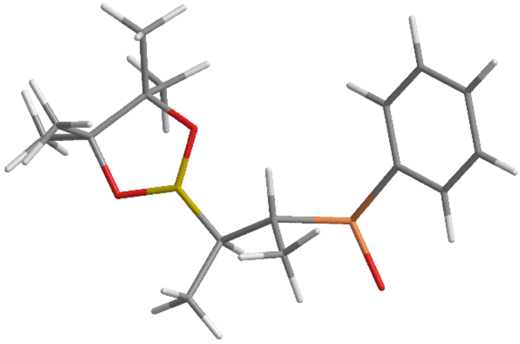

C	0.51593300	-0.98982800	0.61276200
C	-0.80237000	-1.66336700	0.11084200
B	-1.99286500	-0.63167400	0.07935500
O	-3.31078200	-0.98320700	0.19317600
C	-4.10415800	0.24237900	0.27086800
C	-3.12778200	1.31310800	-0.36128700
O	-1.81498400	0.71673000	-0.11226600
C	-3.16585900	2.69152700	0.29226500
C	-3.26781900	1.44382400	-1.88213600
C	-5.40824900	0.02382800	-0.49118500
C	-4.39406800	0.48163000	1.75706400
C	2.98341900	0.51337300	-0.20189800
C	4.26263600	0.13587900	0.20443000
C	5.13455800	1.10777400	0.70595700
C	4.72271800	2.44186500	0.79273900
C	3.43870700	2.81060000	0.37576500
C	2.56292300	1.84413700	-0.12908500
C	1.23474000	-1.65707700	1.77648800
C	-1.17892300	-2.98514600	0.80962900
H	0.34128400	0.07523300	0.83096600
H	-0.66776100	-1.92650800	-0.96053100
H	-2.44128400	3.35641200	-0.20183500
H	-4.16492100	3.14429700	0.18984600
H	-2.91015000	2.64584000	1.35886000
H	-4.20895700	1.94141400	-2.16079900
H	-2.43218900	2.04646700	-2.26802400
H	-3.23378800	0.46161000	-2.37649600
H	-6.00436300	-0.75206600	0.01222500
H	-6.00607600	0.94888900	-0.51590800
H	-5.22897500	-0.30554000	-1.52287200
H	-5.04577800	1.35555500	1.90768200
H	-4.90266600	-0.40318400	2.16754200
H	-3.46719600	0.63510400	2.32962600
H	4.55029300	-0.91563900	0.11613700
H	6.13912500	0.82329800	1.02845900
H	5.40604600	3.19995800	1.18265000
H	3.11919500	3.85347700	0.44039600
H	1.56141500	2.13295600	-0.46148500
H	2.06890000	-1.03004000	2.12604900
H	0.54915800	-1.80684900	2.62555300
H	1.65106500	-2.63228700	1.48162200
H	-2.08258000	-3.40794400	0.34571000
H	-0.37600600	-3.73241100	0.72949700
H	-1.40474800	-2.84053800	1.87756800
Se	1.81672000	-0.88453700	-0.95683400
O	2.74431100	-2.25376100	-0.83168200

Zero-point energy: 0.385085 Hartree
Electronic and zero point energy: -1009.239015 Hartree
One imaginary frequency at: -43.97 cm^{-1}

Structure 1G

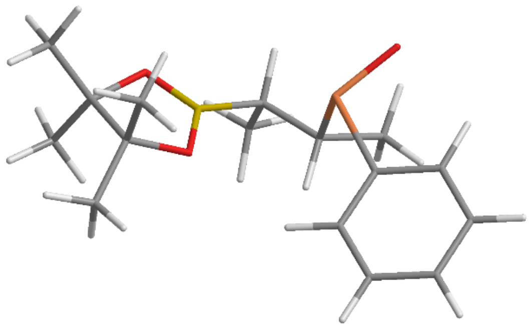

C	-0.63291000	1.50206600	0.75239200
C	0.82639400	1.94792500	0.53927600
B	1.80232700	0.74576500	0.23615800
O	3.07272000	0.91781800	-0.24121600
C	3.77436800	-0.36061200	-0.13951300
C	2.57968500	-1.39478600	-0.06736400
O	1.49158600	-0.57148000	0.46917800
C	2.80040500	-2.57461600	0.87490800
C	2.11917900	-1.89325700	-1.44127600
C	4.68650400	-0.50814000	-1.35407200
C	4.60918100	-0.30103400	1.14487300
C	-2.64252000	-0.52276400	-0.14008300
C	-3.97627400	-0.20706600	-0.39438800
C	-4.98351800	-1.01264100	0.14757300
C	-4.64885300	-2.12248300	0.93020700
C	-3.30586500	-2.43269900	1.17351600
C	-2.29330900	-1.63292300	0.63412800
C	-1.63052500	2.59509200	1.10118300
C	1.38152500	2.72949800	1.75784500
H	-0.67229600	0.67775200	1.48384700
H	0.86098000	2.64001400	-0.32424200
H	1.91216600	-3.22446700	0.87087900
H	3.66152500	-3.17851300	0.54736400
H	2.97547300	-2.24699700	1.90787500
H	2.86057200	-2.56960200	-1.89267500
H	1.17553100	-2.44703900	-1.32599200
H	1.93826000	-1.05973100	-2.13546300
H	5.46628700	0.26762100	-1.32558900
H	5.18440600	-1.49087300	-1.35233400
H	4.13409400	-0.39597900	-2.29590200
H	5.22175100	-1.20625600	1.27292100
H	5.28322000	0.56666000	1.09147500
H	3.97360800	-0.18141600	2.03490000
H	-4.19817600	0.66574300	-1.01511000
H	-6.03266300	-0.77354700	-0.04279800
H	-5.43687100	-2.75159700	1.35083600
H	-3.04566400	-3.30094800	1.78378600
H	-1.24281300	-1.87263800	0.82130500
H	-2.63882500	2.17949900	1.24812700
H	-1.33999900	3.10237500	2.03489700
H	-1.69496300	3.34111700	0.29410400
H	2.43915700	2.99050800	1.59898500
H	0.83019100	3.66594100	1.92966600
H	1.31783800	2.12823300	2.68114900
Se	-1.26811700	0.61401000	-0.98154000
O	-2.16546200	1.77861400	-1.74867000

Zero-point energy: 0.384967 Hartree
Electronic and zero point energy: -1009.245426 Hartree

Structure 1A_TS_SE1

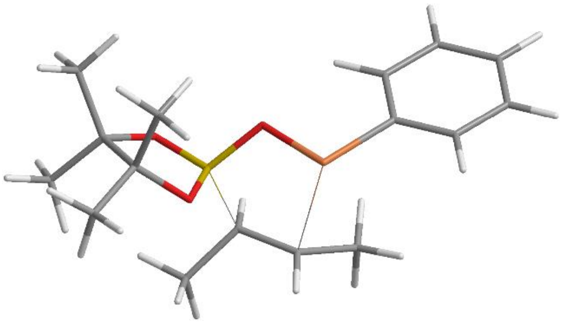

C	0.50984400	2.26654900	-0.34425500
C	-0.55799900	2.02386800	0.56219600
B	-1.16230600	0.29677000	0.37669700
O	-1.83995400	0.19682400	-0.88636600
C	-2.92997600	-0.73531300	-0.70339300
C	-3.27717500	-0.57743700	0.84404300
O	-2.05951600	-0.04495500	1.41182000
C	-3.59383800	-1.89496500	1.56032500
C	-4.41450000	0.41694700	1.11954200
C	-4.05997300	-0.34524600	-1.65748700
C	-2.42491300	-2.14209900	-1.06861100
C	2.79537100	-0.47714600	-0.14693700
C	2.91432300	-0.77258000	1.21553600
C	4.18577800	-0.95758000	1.76709700
C	5.32930500	-0.85328000	0.96911000
C	5.20148900	-0.56440500	-0.39347400
C	3.93615100	-0.37902300	-0.95577600
C	1.87911100	2.67793900	0.10126700
C	-1.80320400	2.89654200	0.33772900
H	0.23262800	2.55824500	-1.36520700
H	-0.22298800	2.02845800	1.61029700
H	-3.80567500	-1.69069000	2.62127700
H	-4.48020200	-2.38497900	1.12511200
H	-2.74758800	-2.59260200	1.51700000
H	-5.39173100	0.00669900	0.81989200
H	-4.44684000	0.62585700	2.19988900
H	-4.26607400	1.36888200	0.59364000
H	-3.72000600	-0.46549200	-2.69792700
H	-4.94260800	-0.98918700	-1.51340400
H	-4.36481200	0.70086900	-1.52270400
H	-3.22562300	-2.89518000	-1.00305900
H	-2.05594900	-2.12594600	-2.10589000
H	-1.59545800	-2.45355800	-0.41878500
H	2.01559700	-0.85845800	1.82749900
H	4.28007300	-1.18906400	2.83071500
H	6.31904900	-1.00064800	1.40658100
H	6.08953300	-0.48561500	-1.02476900
H	3.84530500	-0.16123000	-2.02423600
H	2.64671200	2.57161100	-0.67914600
H	1.82744400	3.75136000	0.37435900
H	2.20380700	2.13475100	1.00200900
H	-2.59329900	2.60754000	1.04249200
H	-1.57462600	3.96326200	0.50362600
H	-2.19404000	2.77783700	-0.68348200
Se	1.07018600	-0.23439100	-0.98118100
O	0.08119500	-0.50361000	0.44947500

Zero-point energy: 0.383353 Hartree
Electronic and zero point energy: -1009.244001 Hartree
One imaginary frequency at: -222.92 cm^{-1}

Structure 1C_TS_SE1

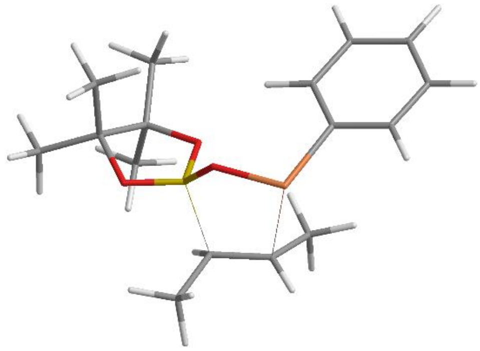

C	-0.42934100	-2.08020800	1.36365400
C	0.92789900	-1.98443400	0.93924200
B	1.16115900	-0.55777900	-0.16852800
O	2.46576000	-0.62089900	-0.72311700
C	3.05402100	0.69319400	-0.66355700
C	2.33335200	1.32398600	0.59134300
O	1.04147600	0.68013000	0.55625800
C	2.12552400	2.83783200	0.52287200
C	3.02274600	0.96662600	1.91844200
C	4.56990800	0.54464500	-0.53178200
C	2.71793700	1.42255200	-1.97425500
C	-2.51131000	0.25817800	-0.34648500
C	-1.94020400	1.50184000	-0.05997100
C	-2.77663300	2.56999400	0.28012100
C	-4.16314600	2.40155000	0.34115600
C	-4.72465600	1.15324700	0.05373200
C	-3.90210100	0.08082500	-0.29890400
C	-0.99903600	-1.18755300	2.42570600
C	1.53070300	-3.23845800	0.30957800
H	-0.95430200	-3.03104800	1.21880700
H	1.54666100	-1.53478500	1.72927700
H	1.60369000	3.18395200	1.42920400
H	3.09028000	3.36797200	0.46638000
H	1.52048000	3.12468800	-0.34762900
H	3.96870700	1.51521600	2.04809000
H	2.35513000	1.23448000	2.75212600
H	3.24357300	-0.10872700	1.98638600
H	4.97678000	0.08756700	-1.44695200
H	5.05489100	1.52556700	-0.39861600
H	4.84355700	-0.10001000	0.31423100
H	3.20154400	2.41062200	-2.03230900
H	3.07586500	0.81257800	-2.81724700
H	1.63243000	1.55081400	-2.09291100
H	-0.85624900	1.61904800	-0.08329200
H	-2.33429400	3.54385600	0.50315400
H	-4.80736200	3.24229600	0.60772100
H	-5.80743000	1.01365200	0.09191700
H	-4.35105900	-0.88640900	-0.54499400
H	-2.09674600	-1.12899800	2.38563100
H	-0.72906300	-1.60943300	3.41306800
H	-0.57581600	-0.17435900	2.36499700
H	2.53948900	-3.03461500	-0.07334400
H	1.59037400	-4.07403000	1.02862500
H	0.92446400	-3.58097000	-0.54651900
Se	-1.48449700	-1.30483000	-0.85865600
O	0.11745600	-0.73407000	-1.22616700

Zero-point energy: 0.382663 Hartree
Electronic and zero point energy: -1009.248391 Hartree
One imaginary frequency at: -237.81 cm^{-1}

Structure 3

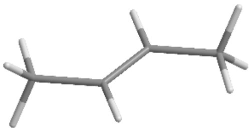

C	-0.53997000	0.39512800	-0.00004500
C	0.53995100	-0.39514900	-0.00005800
C	-1.96420700	-0.07990700	0.00003100
C	1.96420000	0.07993100	0.00002500
H	-0.39193000	1.48390900	-0.00004300
H	0.39208200	-1.48395800	-0.00002800
H	-2.51178800	0.29278400	0.88433500
H	-2.51182500	0.29267700	-0.88429500
H	-2.02635200	-1.17972300	0.00010900
H	2.51159400	-0.29224700	0.88467700
H	2.51201900	-0.29320200	-0.88394700
H	2.02636000	1.17974000	-0.00052200

Zero-point energy: 0.107040 Hartree
Electronic and zero point energy: -157.124101 Hartree

Structure 4

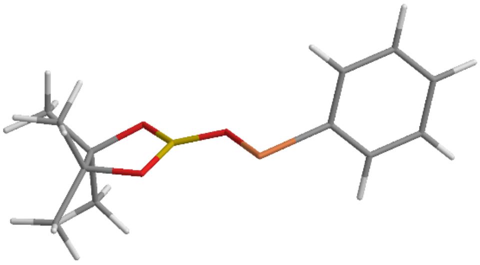

B	-1.13972400	0.34837900	0.06081600
O	-1.82344200	-0.80913600	-0.24834700
C	-3.22571200	-0.58244800	0.08512500
C	-3.32277500	0.99714300	0.04006400
O	-1.96402900	1.40203800	0.37613000
C	-4.27690300	1.61480500	1.05851600
C	-3.61799400	1.54360200	-1.36208500
C	-4.09200600	-1.31490400	-0.93532600
C	-3.44928800	-1.16283100	1.48639700
C	2.91498500	-0.19983400	-0.05054400
C	3.52578300	-0.17861000	1.21267400
C	4.77953400	0.41644100	1.37375400
C	5.42711000	0.99115300	0.27541700
C	4.82188100	0.97284700	-0.98523600
C	3.56810300	0.37867200	-1.14960100
H	-4.26084500	2.71091200	0.96158900
H	-5.31000100	1.27294500	0.88552800
H	-3.99092500	1.36404000	2.08832200
H	-4.65523300	1.33995400	-1.66907000
H	-3.46542000	2.63302600	-1.35759700
H	-2.94039300	1.11040600	-2.11308800
H	-3.94139700	-2.40083100	-0.83791200
H	-5.15980000	-1.10355100	-0.76494700
H	-3.83761400	-1.03014100	-1.96456700
H	-4.50421300	-1.09158500	1.79211600
H	-3.16135200	-2.22470200	1.48267600
H	-2.83300000	-0.64680900	2.23776500
H	3.01433600	-0.62768900	2.06591600
H	5.25141700	0.43182500	2.35886200
H	6.40805300	1.45526900	0.40249600
H	5.32676100	1.42300100	-1.84302600
H	3.08924300	0.36229600	-2.13025000
Se	1.20579100	-1.06014800	-0.28496800
O	0.20694300	0.46566700	0.05910100

Zero-point energy: 0.275044 Hartree
Electronic and zero point energy: -852.185867 Hartree

Structure 1E_TS_SE2

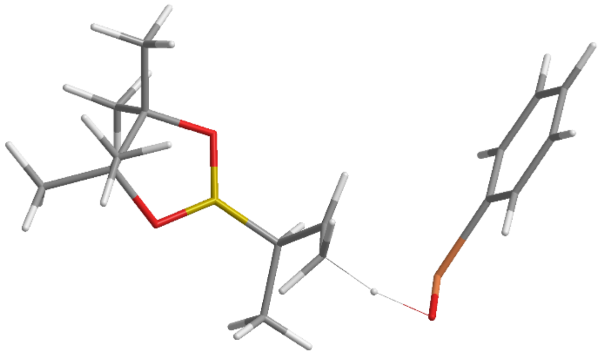

C	0.20224300	-0.56870100	0.31067500
C	-0.77543100	-1.04831900	-0.74273200
B	-2.15355100	-0.33800200	-0.40106400
O	-3.22758400	-0.98020100	0.14917400
C	-4.20476800	0.03683100	0.54265000
C	-3.78461900	1.26957200	-0.35526900
O	-2.37046900	0.99737400	-0.61356400
C	-3.89865400	2.63289500	0.32093300
C	-4.47988700	1.29681400	-1.72121300
C	-5.60409000	-0.51133700	0.27865500
C	-4.00326600	0.27612700	2.04301700
C	3.41311200	0.37596200	-0.17318000
C	4.02962700	0.56065100	1.06710400
C	4.66961400	1.77227700	1.34366200
C	4.69825000	2.79164600	0.38611600
C	4.08404700	2.59750000	-0.85548600
C	3.44062100	1.38926600	-1.13875300
C	0.35286500	-1.18083300	1.59337000
C	-0.90880400	-2.57398300	-0.85967800
H	0.47643900	0.48726700	0.22142400
H	-0.44828100	-0.63408500	-1.71292100
H	-3.56127500	3.41839200	-0.37196900
H	-4.94421600	2.84702900	0.59420300
H	-3.28047300	2.69315400	1.22600100
H	-5.54517500	1.55719900	-1.62885300
H	-3.99315000	2.05386100	-2.35384400
H	-4.40160400	0.32629200	-2.23340700
H	-5.79015900	-1.37841100	0.93009300
H	-6.36934600	0.24986900	0.49933200
H	-5.72580000	-0.83898700	-0.76196800
H	-4.73867200	0.99155600	2.44107300
H	-4.12650800	-0.67827000	2.57598200
H	-2.99356100	0.65544200	2.25907700
H	4.00388200	-0.25042900	1.79823700
H	5.15303800	1.91875700	2.31278700
H	5.20126500	3.73615800	0.60533000
H	4.10461000	3.38917800	-1.60830500
H	2.96373900	1.24428400	-2.11297000
H	0.53268600	-0.49196200	2.42911400
H	-0.34677100	-1.99064900	1.83575200
H	1.47278200	-1.77420500	1.48971100
H	-1.60157500	-2.84110900	-1.67270700
H	0.06368900	-3.04634200	-1.07399000
H	-1.30692400	-3.02491200	0.06130300
Se	2.53197600	-1.31316400	-0.57569900
O	2.66014900	-2.13963700	0.91331200

Zero-point energy: 0.380242 Hartree
Electronic and zero point energy: -1009.227766 Hartree
One imaginary frequency at: -802.63 cm^{-1}

Structure 5

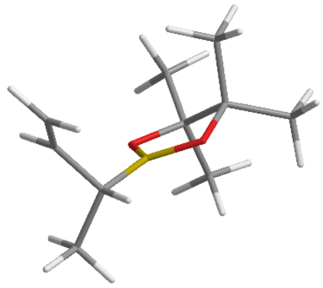

C	-2.93535500	-0.38318700	0.59371100
C	-2.21400900	0.31946700	-0.53424200
B	-0.65963200	0.16655100	-0.30372000
O	0.14704800	-0.63235500	-1.06967400
C	1.45110300	-0.71540400	-0.41368300
C	1.44722000	0.57312100	0.50321300
O	0.01829400	0.80755400	0.70115500
C	2.00328500	1.82067100	-0.19316400
C	2.10248300	0.39826700	1.87059800
C	1.45787900	-2.02824300	0.37789100
C	2.53297400	-0.74311200	-1.48980100
C	-3.63944700	-1.51555500	0.48761800
C	-2.63981000	1.80197900	-0.62733500
H	-2.84611700	0.09656400	1.57740500
H	-2.46902400	-0.19308900	-1.47731300
H	1.78154200	2.70286400	0.42565300
H	3.09386200	1.75848100	-0.32793600
H	1.53838000	1.97296200	-1.17859200
H	3.17089000	0.15121600	1.76376400
H	2.02646000	1.33721400	2.43957500
H	1.61695400	-0.39203200	2.45773400
H	1.26021200	-2.86128800	-0.31289400
H	2.42973700	-2.20459300	0.86344500
H	0.67367500	-2.03701400	1.14926900
H	3.53674900	-0.73859100	-1.03550900
H	2.43520300	-1.66159700	-2.08796300
H	2.45324400	0.11333500	-2.17193600
H	-4.12507000	-1.96820600	1.35596300
H	-3.75541600	-2.02942900	-0.47208700
H	-2.12676200	2.31491300	-1.45732500
H	-3.72567900	1.89191600	-0.79281500
H	-2.39134800	2.34400100	0.29947400

Zero-point energy: 0.278268 Hartree
Electronic and zero point energy: -567.704714 Hartree

Structure 6

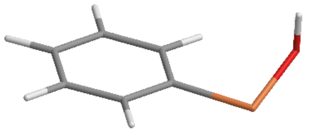

C	-0.33187500	-0.11905300	-0.02977900
C	-0.88769900	1.15702100	-0.18085200
C	-2.27521100	1.31579100	-0.13767100
C	-3.11174400	0.20750100	0.03086900
C	-2.55218400	-1.06581100	0.17195800
C	-1.16434500	-1.23106100	0.15630500
H	-0.23391800	2.01891600	-0.32597800
H	-2.70515600	2.31434300	-0.24829200
H	-4.19596700	0.33617400	0.05628500
H	-3.19667900	-1.93668200	0.31343000
H	-0.73549700	-2.22678900	0.29959000
H	2.16987700	1.30129800	1.32333700
Se	1.57983400	-0.39863900	-0.12667200
O	2.14016600	1.27001600	0.35293600

Zero-point energy: 0.103986 Hartree
Electronic and zero point energy: -441.554051 Hartree

Structure 1H

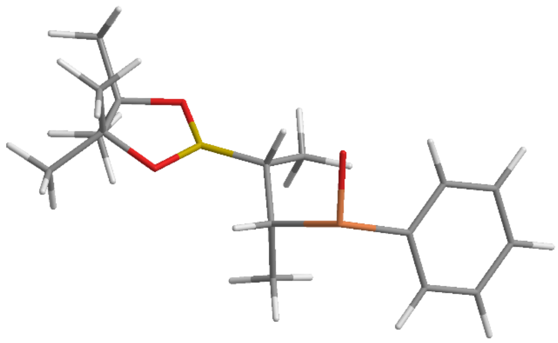

C	-0.34483800	-0.58228400	0.77504200
C	0.32772900	0.37027800	-0.21803900
B	1.89630800	0.31365400	-0.10024000
O	2.72204900	1.28373100	-0.60619300
C	4.09121200	0.97313000	-0.20334300
C	3.99978200	-0.56926200	0.13142400
O	2.58673200	-0.72590400	0.47245300
C	4.83862800	-1.02641200	1.32154900
C	4.26075300	-1.47307400	-1.07895200
C	5.02736700	1.33202500	-1.35427100
C	4.39809300	1.84685600	1.01862600
C	-3.35291900	-0.12270200	-0.02481300
C	-3.67490400	0.52288600	-1.21905600
C	-4.70638000	1.46626300	-1.23047600
C	-5.40965100	1.75233800	-0.05491700
C	-5.09009100	1.08799700	1.13370100
C	-4.06154700	0.13971700	1.15060400
C	-0.69431900	-0.04088800	2.15393100
C	-0.21552700	1.81220100	-0.22599600
H	0.28973700	-1.47613200	0.89227000
H	0.12526900	-0.07093800	-1.22180000
H	4.68829300	-2.10381300	1.48805000
H	5.91076100	-0.85913200	1.13088400
H	4.56118400	-0.50005200	2.24422400
H	5.32185400	-1.46307800	-1.37095800
H	3.98332500	-2.50559300	-0.81986100
H	3.65640000	-1.16946200	-1.94647400
H	5.00324600	2.41893000	-1.52483100
H	6.06522900	1.04792700	-1.11746600
H	4.73288100	0.83771000	-2.28907500
H	5.43917900	1.72492800	1.35428000
H	4.24318000	2.90226400	0.74960400
H	3.73056400	1.61090300	1.86077600
H	-3.11187300	0.26549500	-2.12056200
H	-4.96510800	1.97763100	-2.16084600
H	-6.21678600	2.48849300	-0.06648400
H	-5.64603600	1.30263800	2.04943700
H	-3.82789400	-0.39238100	2.07675900
H	-1.15735400	-0.81518100	2.78893100
H	0.22649100	0.28680300	2.66720900
H	-1.37692200	0.82056300	2.10932900
H	0.26170800	2.38823700	-1.03269700
H	-1.30258400	1.84348700	-0.38839100
H	0.00050400	2.34221600	0.71635300
Se	-1.96199900	-1.52127700	-0.09792600
O	-1.52992200	-1.51804500	-1.70226500

Zero-point energy: 0.385166 Hartree
Electronic and zero point energy: -1009.243667 Hartree

Structure 1H_TS_SE3

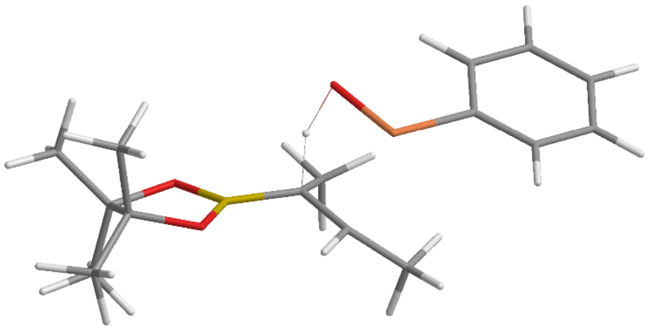

C	-0.31849500	0.04453500	1.37776500
C	0.37398600	0.82521100	0.37417600
B	1.88682100	0.50188100	0.21560000
O	2.77143800	1.32498700	-0.43651500
C	4.10795300	0.75829600	-0.28803300
C	3.79458000	-0.75379000	0.05066500
O	2.47065300	-0.66270900	0.65931200
C	4.74504000	-1.40360000	1.05262800
C	3.64856700	-1.63869900	-1.19299400
C	4.87547100	0.98494100	-1.58792200
C	4.78268000	1.51426600	0.86292700
C	-3.32214300	-0.33064800	-0.22075100
C	-3.49763200	0.61597600	-1.23348400
C	-4.69021800	1.34203600	-1.29630400
C	-5.70333000	1.11958500	-0.35706500
C	-5.52382800	0.16471400	0.64861700
C	-4.33260800	-0.56447100	0.71863300
C	-1.28288400	0.64016100	2.36646500
C	-0.06221300	2.28006300	0.16142900
H	0.23110500	-0.82493200	1.75454200
H	0.00582100	0.20868600	-0.67001800
H	4.43144200	-2.44179700	1.24004900
H	5.77357400	-1.42622500	0.65813900
H	4.75030100	-0.87365500	2.01417100
H	4.61714500	-1.80675300	-1.68817300
H	3.24199800	-2.61486900	-0.88939700
H	2.95187200	-1.19809400	-1.92119800
H	5.02357200	2.06391600	-1.74653200
H	5.86794600	0.50819900	-1.54498700
H	4.33236800	0.58802900	-2.45534800
H	5.82683300	1.19521000	1.00276900
H	4.77817300	2.59014400	0.63283600
H	4.24322300	1.36849900	1.81068100
H	-2.69814400	0.76709100	-1.96225100
H	-4.83076000	2.08286700	-2.08718400
H	-6.63486500	1.68742400	-0.41108500
H	-6.31306900	-0.01565900	1.38230700
H	-4.20110400	-1.31409000	1.50469600
H	-1.81934500	-0.13527500	2.93513000
H	-0.71940300	1.25130100	3.09821500
H	-2.02516500	1.30185600	1.89696100
H	0.47423700	2.71778300	-0.69290300
H	-1.14000600	2.36382900	-0.04746900
H	0.15472500	2.91121600	1.04103900
Se	-1.67974800	-1.37698500	-0.14776900
O	-0.76892900	-0.69203500	-1.41396700

Zero-point energy: 0.380250 Hartree
Electronic and zero point energy: -1009.234742 Hartree
One imaginary frequency at: -600.86 cm^{-1}

Structure 7

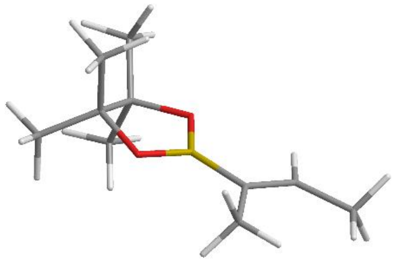

C	-2.90134100	-0.75669300	0.11734000
C	-2.12215200	0.33668600	-0.04181600
B	-0.58139500	0.14573100	-0.01258000
O	0.30025100	1.18123200	-0.21693300
C	1.64289600	0.69665700	0.08205000
C	1.47299400	-0.86777600	-0.06698300
O	0.05516500	-1.05216000	0.21611800
C	2.27858100	-1.70483100	0.92340600
C	1.70824600	-1.37128000	-1.49667000
C	2.62119800	1.33995900	-0.89747200
C	1.96208400	1.14072300	1.51494600
C	-4.39909600	-0.84744800	0.13557700
C	-2.64840100	1.74100800	-0.24596000
H	-2.38473300	-1.71448600	0.25367700
H	2.07677400	-2.77343400	0.75414300
H	3.35943000	-1.53786600	0.78941100
H	2.01387900	-1.47282400	1.96326300
H	2.77265000	-1.32764400	-1.77372000
H	1.37600200	-2.41792200	-1.56452600
H	1.13228400	-0.78572100	-2.22864000
H	2.65265600	2.42705700	-0.72862500
H	3.63894900	0.94385000	-0.75150300
H	2.32503000	1.16968700	-1.94079800
H	2.98884500	0.87063800	1.80544200
H	1.86123700	2.23441300	1.57953300
H	1.26486000	0.69298100	2.23865500
H	-4.75483000	-1.53136300	-0.65602500
H	-4.74806900	-1.28033600	1.09044700
H	-4.90031900	0.12062700	-0.00151900
H	-2.27382000	2.16369300	-1.19384400
H	-3.74575300	1.80055700	-0.25954300
H	-2.28148800	2.41483000	0.54708600

Zero-point energy: 0.277955 Hartree
Electronic and zero point energy: -567.716018 Hartree

Structure 2A

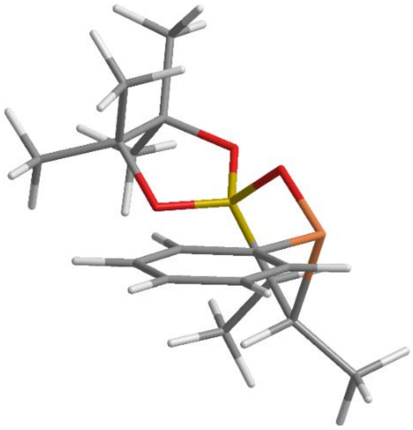

C	-0.86169400	1.98292500	0.74948100
C	0.65003300	1.99885100	0.60489900
Se	-1.45153300	1.01947500	-0.98223700
B	1.20382600	0.63238900	-0.12419500
O	1.23508500	-0.53985200	0.71668900
C	2.29243600	-1.39956000	0.24074800
C	3.31065400	-0.36284100	-0.38875800
O	2.46392200	0.73049900	-0.77977000
C	4.05833100	-0.86857600	-1.62572200
C	4.32848800	0.17223600	0.63223400
C	2.83751100	-2.19566400	1.42829300
C	1.71506200	-2.37518300	-0.79979300
C	-2.46855900	-0.45627600	-0.21532800
C	-3.82939900	-0.51311600	-0.53562500
C	-4.61460200	-1.54033400	-0.00247500
C	-4.03123400	-2.50413100	0.82455100
C	-2.66469500	-2.44406700	1.12212200
C	-1.86896600	-1.41851300	0.60423600
O	0.11122200	0.40598000	-1.32396400
C	-1.59777400	3.31119500	0.85045600
C	1.31757900	2.23691700	1.97037200
H	-1.18284500	1.29193100	1.54579400
H	0.91698000	2.83744400	-0.06661600
H	4.73221600	-0.07957600	-1.99453000
H	4.67050800	-1.75501200	-1.39064200
H	3.36579600	-1.12275500	-2.43881300
H	5.08006900	-0.58588400	0.90459800
H	4.85247600	1.03257900	0.18832000
H	3.83145300	0.51479300	1.55088900
H	2.06195600	-2.88252800	1.80327500
H	3.70909300	-2.80275700	1.13358300
H	3.13236300	-1.53867800	2.25717500
H	2.47411400	-3.09302600	-1.14845600
H	0.89615500	-2.95005500	-0.33885500
H	1.30520700	-1.84152500	-1.66795200
H	-4.27938100	0.23175300	-1.19774100
H	-5.67860100	-1.59073300	-0.24331300
H	-4.64349000	-3.31040500	1.23477100
H	-2.21096100	-3.20294500	1.76342700
H	-0.79875500	-1.35904900	0.82472600
H	-2.69251600	3.18791000	0.90114300
H	-1.28363300	3.82524200	1.77430600
H	-1.35538100	3.97534300	0.00498000
H	2.40770400	2.31541600	1.84126400
H	0.97713400	3.16672100	2.45904600
H	1.12448200	1.39551100	2.65486700

Zero-point energy: 0.383731 Hartree
Electronic and zero point energy: -1009.251586 Hartree

Structure 2B

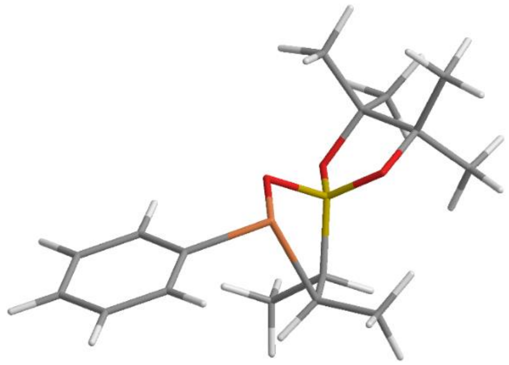

C	-0.66105300	1.70454200	0.93893900
C	0.34971900	0.80963300	1.60728500
Se	-1.09294100	0.60236700	-0.85811400
B	1.15180700	-0.06983400	0.44729100
O	1.98673400	-1.11570500	0.91509300
C	3.13085300	-1.20394800	0.04286900
C	3.28385700	0.28609000	-0.46421800
O	1.92380400	0.75783600	-0.46128100
C	3.85562200	0.42996900	-1.87566000
C	4.09174600	1.16190400	0.50847500
C	4.31443500	-1.73277900	0.85434300
C	2.80559900	-2.18994500	-1.09131900
C	-2.81993500	-0.17568600	-0.40062300
C	-3.97366300	0.58381900	-0.61880300
C	-5.22181200	0.02847900	-0.32161100
C	-5.30684500	-1.27668800	0.17315400
C	-4.14475800	-2.02936500	0.37192200
C	-2.89061100	-1.48274300	0.08412000
O	-0.05506400	-0.69330800	-0.39967000
C	-0.20255500	3.09022500	0.51854700
C	-0.24933600	-0.06736700	2.71407800
H	-1.65509500	1.71926000	1.41293800
H	1.12957700	1.46734500	2.03012200
H	3.91475600	1.49641600	-2.14566400
H	4.87265300	0.00893200	-1.93900200
H	3.22203600	-0.07072600	-2.61983100
H	5.16472500	0.91279200	0.49884200
H	3.98253300	2.21660700	0.21118600
H	3.72132300	1.05761900	1.53909200
H	4.11441600	-2.76972200	1.16633300
H	5.23998000	-1.73160500	0.25540900
H	4.48172900	-1.13697300	1.76156600
H	3.67535500	-2.36659900	-1.74411400
H	2.50853500	-3.15187600	-0.64602300
H	1.96720200	-1.83048000	-1.70395700
H	-3.91163400	1.60045700	-1.01786400
H	-6.12792400	0.61656300	-0.48241100
H	-6.28328200	-1.70992200	0.40038100
H	-4.21188700	-3.05070200	0.75310800
H	-1.97159300	-2.05291800	0.23053300
H	-0.95076100	3.61593800	-0.09778300
H	-0.03890000	3.69634500	1.42634900
H	0.74877900	3.03762900	-0.03115000
H	0.53152400	-0.70009300	3.16163700
H	-0.71135900	0.53117700	3.51963000
H	-1.02311300	-0.74418100	2.31795100

Zero-point energy: 0.384245 Hartree
Electronic and zero point energy: -1009.252452 Hartree

Structure 2C

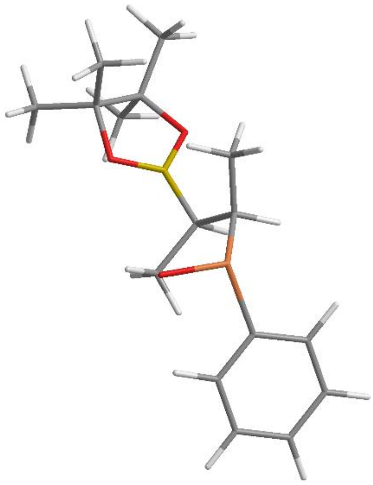

C	-0.45236000	-0.43874500	-1.00668300
C	0.26402600	0.70888800	-0.21881800
Se	-2.03678900	-1.43775600	-0.17041900
B	1.82897000	0.49877500	-0.09829600
O	2.71911300	0.94203500	-1.04535700
C	4.06619000	0.84612700	-0.48528900
C	3.87154200	-0.19724300	0.68939600
O	2.44484100	-0.07860000	0.97821900
C	4.64645000	0.11357300	1.96786000
C	4.11992600	-1.65058200	0.26928200
C	5.02192400	0.40701000	-1.59159900
C	4.43527000	2.25292100	-0.00006000
C	-3.35988800	0.02375100	-0.08670500
C	-3.82885800	0.64763200	-1.24552800
C	-4.83268800	1.61645000	-1.14476800
C	-5.36869900	1.94122200	0.10586100
C	-4.90364500	1.29711900	1.25748400
C	-3.89606900	0.33248400	1.16366800
O	-1.71457300	-1.77657100	1.41825200
C	0.47348300	-1.56210400	-1.48740400
C	-0.30694700	1.05742600	1.17446000
H	-0.96697700	-0.01277600	-1.88627800
H	0.14369500	1.60598100	-0.85209300
H	4.42244300	-0.64961700	2.72813800
H	5.73218500	0.10011600	1.78110400
H	4.37394900	1.09224400	2.38366500
H	5.18995000	-1.84482000	0.09994300
H	3.77016400	-2.31708100	1.07151200
H	3.57063300	-1.90813800	-0.64764900
H	5.06603200	1.18250400	-2.37120400
H	6.04003200	0.26564700	-1.19514100
H	4.69875500	-0.52938400	-2.06475900
H	5.46306200	2.29073600	0.39163500
H	4.36170100	2.95267200	-0.84583300
H	3.74965100	2.60055600	0.78686800
H	-3.42425200	0.38723300	-2.22801500
H	-5.19862300	2.11489700	-2.04549300
H	-6.15596200	2.69486000	0.18116600
H	-5.32739400	1.54689900	2.23318100
H	-3.50896700	-0.18781000	2.04407000
H	-0.06400900	-2.31058100	-2.09206000
H	1.27678600	-1.14392200	-2.11505000
H	0.94014600	-2.08215500	-0.63540000
H	0.28869100	1.86652500	1.62792000
H	-1.34444500	1.41315700	1.11039000
H	-0.28426600	0.19161800	1.84925500

Zero-point energy: 0.385162 Hartree
Electronic and zero point energy: -1009.227606 Hartree
One imaginary frequency at: -42.50 cm^{-1}

Structure 2D

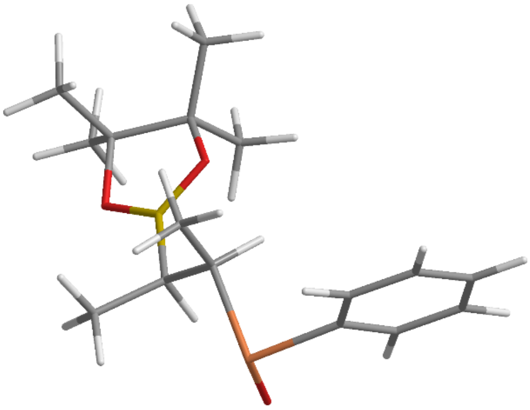

C	0.48473700	-0.63919400	0.55519000
C	-0.68948400	-1.31057400	-0.16509400
Se	2.22116200	-1.41754800	-0.22721000
B	-1.99872100	-0.43992200	-0.07173400
O	-2.00450300	0.91054000	0.17054000
C	-3.34891200	1.41201100	-0.11072700
C	-4.22594800	0.10241000	0.01512300
O	-3.25433400	-0.94961500	-0.27564100
C	-5.37274100	-0.00860600	-0.98595300
C	-4.73940400	-0.15594200	1.43644600
C	-3.67545800	2.51174900	0.89572600
C	-3.31252600	1.98819700	-1.53085900
C	3.27888600	0.23427800	-0.02448600
C	3.95716400	0.52242700	1.16228700
C	4.71851400	1.69336100	1.24853700
C	4.80451400	2.55360600	0.14919000
C	4.13510800	2.24393100	-1.04026500
C	3.37089800	1.07714900	-1.13211000
O	1.88395400	-1.45088200	-1.85282400
C	0.51875200	-0.75493600	2.07396000
C	-0.91981400	-2.78736800	0.20651400
H	0.53987800	0.41740300	0.25499300
H	-0.42184700	-1.27895900	-1.24380600
H	-5.90428700	-0.96022200	-0.83470000
H	-6.09563300	0.81078300	-0.84485800
H	-5.01331600	0.01529700	-2.02283900
H	-5.52871000	0.55716900	1.71893800
H	-5.15844700	-1.17198000	1.48456800
H	-3.92829900	-0.08902500	2.17680100
H	-2.99733900	3.36505600	0.74392100
H	-4.70830200	2.87065200	0.76076500
H	-3.55748300	2.16695600	1.93133300
H	-4.27495800	2.44447000	-1.80794500
H	-2.53473600	2.76456200	-1.58091000
H	-3.06607300	1.21499200	-2.27356300
H	3.90860800	-0.16046900	2.01523600
H	5.25092400	1.92862300	2.17315000
H	5.40395100	3.46444500	0.21719300
H	4.21323500	2.91203800	-1.90145000
H	2.84899200	0.79373400	-2.05057600
H	1.36474500	-0.19506900	2.50365400
H	-0.40241300	-0.32389900	2.50264700
H	0.58944700	-1.79992100	2.41634000
H	-1.71871000	-3.21691400	-0.41534100
H	-0.01524600	-3.39742700	0.04492700
H	-1.22654500	-2.91140100	1.25785600

Zero-point energy: 0.384869 Hartree
Electronic and zero point energy: -1009.245577 Hartree

Structure 2E

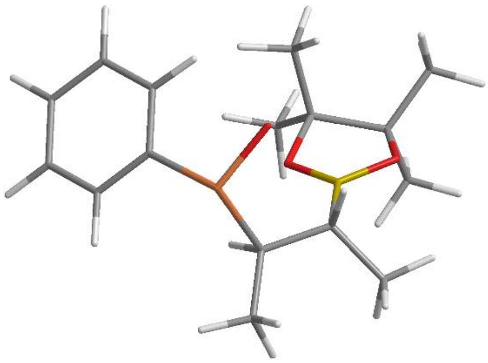

C	0.72891600	-1.74949800	0.77618100
C	-0.60864200	-2.04002600	0.06016600
Se	2.13780800	-1.23049200	-0.65796300
B	-1.58076700	-0.80247800	0.05982000
O	-1.27278400	0.43396900	0.56356000
C	-2.33845400	1.35701200	0.17379100
C	-3.54204900	0.37292800	-0.11392200
O	-2.85252800	-0.86856700	-0.44973300
C	-4.43272800	0.76428500	-1.29027900
C	-4.40032300	0.07891900	1.12238500
C	-2.56322400	2.34312000	1.31675500
C	-1.84528100	2.09637600	-1.07474100
C	2.45846600	0.62435100	-0.08111700
C	3.13654700	0.90149200	1.10840600
C	3.39910400	2.23121700	1.45305100
C	2.99762200	3.26647700	0.60199900
C	2.33256900	2.97436100	-0.59370400
C	2.06000400	1.64696200	-0.93990600
O	1.27715000	-1.05275300	-2.06663000
C	1.36236500	-2.88600500	1.57653800
C	-1.33964300	-3.31340400	0.54496000
H	0.66121000	-0.84687700	1.40068300
H	-0.38504500	-2.18633200	-1.01819100
H	-5.22137400	0.00831400	-1.42333100
H	-4.91993900	1.73541700	-1.10707900
H	-3.86470200	0.82706100	-2.22738000
H	-5.00818200	0.94972500	1.41172200
H	-5.07965900	-0.75584200	0.89412800
H	-3.78053400	-0.21476600	1.98273400
H	-1.66128000	2.95814300	1.45520300
H	-3.40414300	3.01815000	1.09047000
H	-2.77150600	1.83065600	2.26515900
H	-2.57377100	2.84966300	-1.41149800
H	-0.90220200	2.60820200	-0.83442500
H	-1.64862000	1.39958100	-1.90270200
H	3.46541000	0.09384900	1.76910600
H	3.92363200	2.45780300	2.38426600
H	3.21107700	4.30400700	0.86938400
H	2.02724200	3.78391900	-1.26131400
H	1.54430900	1.38208500	-1.86667900
H	2.32167500	-2.57378600	2.02224700
H	0.70466100	-3.19007800	2.40671200
H	1.55644900	-3.77963200	0.96107800
H	-2.26614500	-3.44831900	-0.03224800
H	-0.73201100	-4.22177000	0.41373300
H	-1.62436700	-3.25266200	1.60892100

Zero-point energy: 0.384949 Hartree
Electronic and zero point energy: -1009.239106 Hartree
One imaginary frequency at: -37.55 cm^{-1}

Structure 2B_TS_SE1

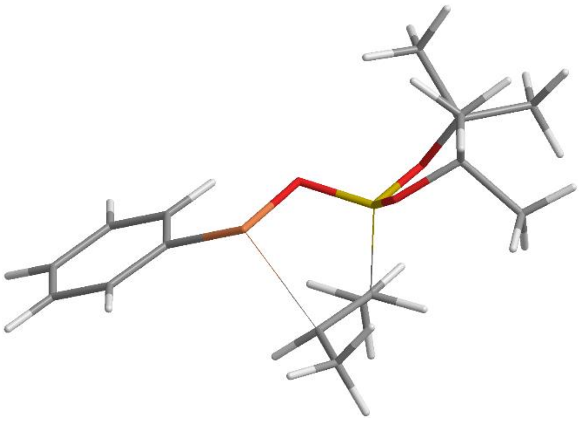

C	-0.53674200	1.77038700	1.08716300
C	0.40706800	0.84473300	1.67185200
Se	-1.10349000	0.51732300	-0.94223900
B	1.11637800	-0.13588300	0.39374000
O	1.98866000	-1.12861800	0.90463100
C	3.14475400	-1.19191100	0.04396100
C	3.23674400	0.28742300	-0.50705400
O	1.85338200	0.69306900	-0.53354800
C	3.81084900	0.41403900	-1.91891700
C	3.99949200	1.22767100	0.44110300
C	4.34391000	-1.64627900	0.87625900
C	2.86339200	-2.22223600	-1.06160900
C	-2.83478500	-0.18752800	-0.42696700
C	-3.97332200	0.56973900	-0.72845000
C	-5.23661200	0.07824100	-0.38755800
C	-5.35930400	-1.16404600	0.24237300
C	-4.21554700	-1.91577500	0.53059300
C	-2.94684200	-1.43353400	0.19637300
O	-0.09680400	-0.74074900	-0.27785800
C	-0.08957800	3.11644200	0.58462600
C	-0.12889000	-0.04052400	2.79950300
H	-1.57507200	1.72741900	1.44014400
H	1.32476100	1.38370700	1.95596600
H	3.82775000	1.47352200	-2.21998600
H	4.84463300	0.03374300	-1.96340100
H	3.20340800	-0.13395500	-2.65122500
H	5.08261800	1.02827600	0.43455600
H	3.84222700	2.26778500	0.11534400
H	3.64073100	1.13431500	1.47673300
H	4.18491000	-2.68043100	1.21910500
H	5.27141900	-1.62550800	0.28087100
H	4.48280100	-1.01678900	1.76540800
H	3.74008400	-2.38009600	-1.70965400
H	2.60585800	-3.18261700	-0.58971200
H	2.01149200	-1.91542800	-1.68468100
H	-3.88546000	1.53907500	-1.22820800
H	-6.12613400	0.66882200	-0.61796000
H	-6.34735300	-1.54747300	0.50585400
H	-4.30846900	-2.88885500	1.01830100
H	-2.04650000	-2.01047100	0.41240400
H	-0.84234300	3.60250800	-0.05557100
H	0.07825800	3.77737200	1.45583200
H	0.86033500	3.03467600	0.03638100
H	0.62315600	-0.78752600	3.09134000
H	-0.39740300	0.54710400	3.69509400
H	-1.02835900	-0.58939900	2.47918300

Zero-point energy: 0.383209 Hartree
Electronic and zero point energy: -1009.251896 Hartree
One imaginary frequency at: -175.67 cm^{-1}

Structure 2F

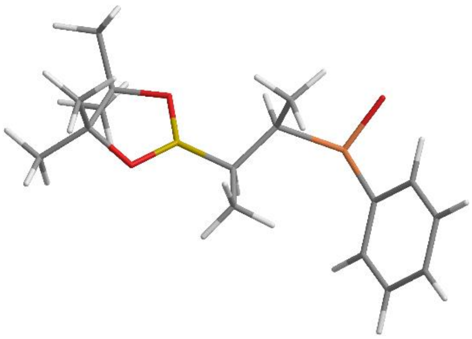

C	-0.32996000	-0.69847000	0.42065600
C	0.37779200	0.49617000	-0.25258600
Se	-1.98021900	-1.35964900	-0.59594200
B	1.94865400	0.36288800	-0.12116100
O	2.80198800	1.43145000	-0.18752900
C	4.14979800	0.95509000	0.12405900
C	4.03051700	-0.59604900	-0.15986100
O	2.59890900	-0.83382800	0.02549300
C	4.79840800	-1.49436300	0.80548000
C	4.35215000	-0.97910400	-1.60880800
C	5.14322000	1.70225000	-0.76127900
C	4.40061700	1.28952000	1.59878300
C	-3.29762800	0.07164100	-0.25918000
C	-3.42924400	1.15381800	-1.13373900
C	-4.42877800	2.10465300	-0.90372400
C	-5.29222000	1.96034600	0.18783800
C	-5.16580100	0.86090200	1.04370400
C	-4.16925500	-0.09350400	0.81713500
O	-2.52051500	-2.57499100	0.39733000
C	-0.64372500	-0.58185500	1.90273900
C	-0.10912200	1.89238800	0.18349600
H	0.28168300	-1.60254300	0.26210700
H	0.20703900	0.40689700	-1.34837300
H	4.63140300	-2.54933900	0.54106300
H	5.88048200	-1.29625100	0.74590100
H	4.47275500	-1.35168000	1.84401900
H	5.42791100	-0.88885400	-1.82243700
H	4.05377000	-2.02465000	-1.77596800
H	3.80130400	-0.35071100	-2.32449700
H	5.13455800	2.77231600	-0.50479700
H	6.16613700	1.32395800	-0.60563700
H	4.89341400	1.60858500	-1.82612100
H	5.42207800	1.02027500	1.90735200
H	4.26913200	2.37169600	1.74631000
H	3.68938300	0.76915400	2.25730800
H	-2.76530300	1.25786000	-1.99690900
H	-4.53835000	2.95388700	-1.58229000
H	-6.07519500	2.70184500	0.36319800
H	-5.85085700	0.74247100	1.88674400
H	-4.06424900	-0.98254900	1.44483100
H	0.29201500	-0.50699300	2.48402900
H	-1.25996800	0.30076700	2.13298200
H	-1.18173300	-1.48252900	2.23751100
H	0.38778500	2.67027300	-0.41541400
H	-1.19516400	2.01393400	0.06932500
H	0.14047900	2.09415300	1.23696400

Zero-point energy: 0.385135 Hartree
Electronic and zero point energy: -1009.240388 Hartree

Structure 2F_TS_SE2

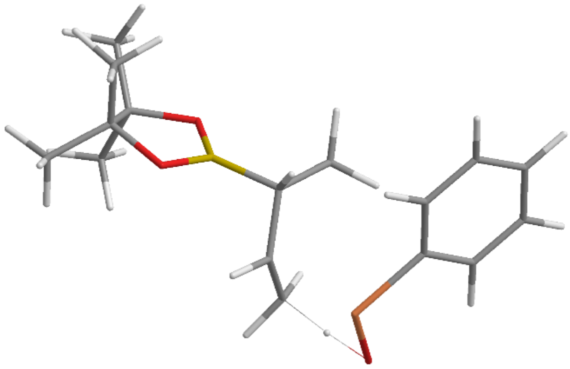

C	-0.05763000	-1.20808300	0.27273100
C	0.33427700	0.25264300	0.21015300
Se	-2.49209800	-1.47795100	-0.71454000
B	1.91721800	0.27130100	0.07607900
O	2.75764700	0.80851200	1.01019500
C	4.12773400	0.44106000	0.64685900
C	3.98690000	0.10498700	-0.89222000
O	2.57531000	-0.26407400	-1.00007400
C	4.83238700	-1.06811700	-1.37980200
C	4.19137100	1.31982900	-1.80447000
C	5.04486700	1.61598000	0.97369900
C	4.49455000	-0.77312500	1.50721100
C	-3.26491300	0.26637700	-0.33464800
C	-3.08658400	1.31477700	-1.24484900
C	-3.66578200	2.56144800	-0.98701700
C	-4.42180800	2.75742100	0.17268100
C	-4.60307200	1.70093500	1.07299600
C	-4.02915400	0.45226400	0.82144800
O	-2.81119400	-2.33541300	0.72478900
C	-0.35591400	-1.91678700	1.48119500
C	-0.16927200	1.12806600	1.36697700
H	0.31130900	-1.79705300	-0.57266100
H	-0.03787900	0.66330900	-0.74735900
H	4.64633500	-1.23789200	-2.45102200
H	5.90576400	-0.85596600	-1.25105600
H	4.59208700	-1.99574200	-0.84432700
H	5.24618800	1.63231700	-1.83310800
H	3.88227200	1.05439400	-2.82636300
H	3.58301300	2.17645800	-1.47812000
H	5.05475900	1.78550900	2.06087300
H	6.07770700	1.40558900	0.65320500
H	4.71022700	2.54329500	0.49081400
H	5.53695000	-1.08547200	1.34310400
H	4.37663900	-0.50709000	2.56805900
H	3.83626600	-1.62944700	1.29846700
H	-2.50101800	1.16706600	-2.15737600
H	-3.52441400	3.38122400	-1.69552800
H	-4.87375400	3.73158200	0.37250200
H	-5.20006200	1.84932200	1.97630000
H	-4.16084200	-0.38607700	1.50928800
H	0.07022200	-2.92797500	1.54724200
H	-0.26416400	-1.34699500	2.41355200
H	-1.57141700	-2.18847400	1.37097400
H	0.09433400	2.18185300	1.18855800
H	-1.26097700	1.06720100	1.47743100
H	0.29384700	0.84265800	2.32357100

Zero-point energy: 0.380574 Hartree
Electronic and zero point energy: -1009.224448 Hartree
One imaginary frequency at: -663.38 cm^{-1}

Structure 2D_TS_SE3

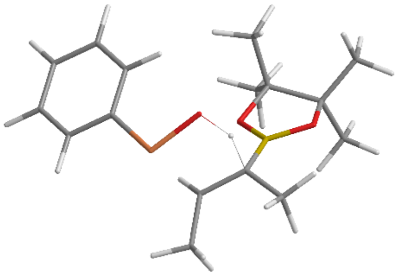

C	0.53137200	-1.60708400	1.06866100
C	-0.54174500	-1.80478800	0.11968000
Se	2.37653600	-1.27140900	-0.55190300
B	-1.65207200	-0.71738100	0.12263200
O	-1.49718300	0.54340800	0.65088600
C	-2.62860100	1.35289900	0.21130500
C	-3.71425100	0.25337700	-0.12234300
O	-2.88973700	-0.90635900	-0.44361300
C	-4.60279500	0.56333600	-1.32430600
C	-4.57624400	-0.13804100	1.08413800
C	-3.00267500	2.31372600	1.33681300
C	-2.15999000	2.13811000	-1.01975500
C	2.67993200	0.61136200	-0.15673300
C	3.59079300	0.96469600	0.84494600
C	3.83273000	2.31532400	1.11286400
C	3.17167600	3.30510500	0.37856000
C	2.26802600	2.94153200	-0.62551300
C	2.01919400	1.59321400	-0.89779700
O	1.16167900	-1.20750800	-1.74609800
C	1.03594200	-2.69479700	1.98136100
C	-0.97325700	-3.24438700	-0.18373000
H	0.60891100	-0.59635700	1.48463900
H	0.05185200	-1.42918700	-0.94855600
H	-5.30688100	-0.26664100	-1.48742700
H	-5.19081000	1.47888900	-1.15079100
H	-4.01549400	0.69183200	-2.24276500
H	-5.28268700	0.66101500	1.35628400
H	-5.15472200	-1.03888000	0.83056700
H	-3.95634600	-0.37004500	1.96298500
H	-2.17682700	3.02058900	1.50868400
H	-3.89960500	2.89647800	1.07227900
H	-3.19442100	1.78391800	2.27908300
H	-2.93788600	2.82666500	-1.38363500
H	-1.27376700	2.72965600	-0.74583700
H	-1.87616800	1.46433600	-1.84184300
H	4.11654800	0.19530200	1.41860100
H	4.54152700	2.59346200	1.89641200
H	3.36453000	4.35990600	0.58675100
H	1.75569000	3.71317000	-1.20547600
H	1.32181700	1.28858300	-1.68080000
H	1.96557400	-2.40139100	2.49343700
H	0.27935200	-2.89381200	2.76514700
H	1.21106100	-3.64632600	1.45786200
H	-1.73067700	-3.25692900	-0.98037800
H	-0.12741200	-3.86217200	-0.52805900
H	-1.41372400	-3.74507700	0.69604900

Zero-point energy: 0.379975 Hartree
Electronic and zero point energy: -1009.234468 Hartree
One imaginary frequency at: -694.01 cm^{-1}

3.1 References

3.1.1 References for Supporting Information

[1] A. B. Pangborn, M. A. Gairdello, R. H. Grubbs, R. K. Rosen, F. J. Timmers, *Organometallics*, **1996**, *15*, 1518.

[2] A. F. Burchat, J. M. Chong, N. Nielsen, *J. Organomet. Chem.* **1997**, *542*, 281.

[3] *Purification of Laboratory Chemicals*, 3rd edition. D.D. Perrin, W. L. F. Armarego, *Pergamon Press*, Oxford, **1988**.

[4] Y. R. Bhorge, S.-H. Chang, C.-T. Chang, T.-H. Yan, *Tetrahedron* **2012**, *68*, 4846.

[5] Z. Huang, E. Negishi, *Org. Lett.* **2006**, *8*, 3675.

[6] L. M. Kreis, E. M. Carreira, *Angew. Chem. Int. Ed.* **2012**, *51*, 3436.

[7] M. Sidera, A. M. Costa, J. Vilarrasa, *Org. Lett.* **2011**, *13*, 4934.

[8] H. Le, A. Batten, J. P. Morken, *Org. Lett.* **2014**, *16*, 2096.

[9] W. B. Reid, J. J. Spillane, S. B. Krause, D. A. Watson, *J. Am. Chem. Soc.* **2016**, *138*, 5539.

[10] R. Larouche-Gauthier, T. G. Elford, V. K. Aggarwal, *J. Am. Chem. Soc.* **2011**, *133*, 16794.

[11] V. K. Aggarwal, L. T. Ball, S. Carobene, R. L. Connelly, M. J. Hesse, B. M. Partridge, P. Roth, S. P. Thomas, M. P. Webster, *Chem. Commun.* **2012**, *48*, 9230.

[12] B. G. Lawhorn, S. B. Boga, S. E. Wolkenberg, D. A. Colby, C.-M. Gauss, M. R. Swingle, L. Amable, R. E. Honkanen, D. L. Boger, *J. Am. Chem. Soc.* **2006**, *128*, 16720.

[13] C. Sandford, R. Rasappan, V. K. Aggarwal, *J. Am. Chem. Soc.* **2015**, *137*, 10100.

[14] P. J. Unsworth, D. Leonori, V. K. Aggarwal, *Angew. Chem. Int. Ed.* **2014**, *53*, 9846.

[15] Y. Kiyotsuka, Y. Katayama, H. P. Acharya, T. Hyodo, Y. Kobayashi, *J. Org. Chem.* **2009**, *74*, 1939.

[16] C. Sandford, R. Rasappan, V. K. Aggarwal, *J. Am. Chem. Soc.* **2015**, *137*, 10100.

[17] H. J. Reich, S. K. Shah, F. Chow, *J. Am. Chem. Soc.* **1979**, *101*, 6648.

[18] H. Ito, K. Kubota, *Org. Lett.* **2012**, *14*, 890.

[19] T. Nakagiri, M. Murai, K. Takai, *Org. Lett.* **2015**, *17*, 3346.

[20] M.-B. Li, Y. Wang, S.-K. Tian, *Angew. Chem. Int. Ed.* **2012**, *51*, 2968.

[21] A. Schmidt, A. R. Nödling, G. Hilt, *Angew. Chem. Int. Ed.,* **2015**, *54*, 801

[22] M. Sidera, A. M. Costa, J. Vilarrasa, *Org. Lett.*, **2011**, *13*, 4934.

[23] S. Farhat, I. Zouev, I. Marek, *Tetrahedron*, **2004**, *60*, 1329

[24] A. Schmidt, A. R. Nödling, G. Hilt, *Angew. Chem. Int. Ed.,* **2015**, *54*, 801

[25] T. Tokuyasu, S. Kunikawa, K. J. McCullough, A. Masuyama, M. Nojima, *J. Org. Chem.* **2005**, *70*, 251.

[26] E. Vedejs, D. A. Perry, R. G. Wilde, *J. Am. Chem. Soc.* **1986**, *108*, 2985.

[27] Y. Li, Y.-Y. Hu, S.-L. Zhang, *Chem. Commun.* **2013**, *49*, 10635.

[28] A. Larivée, J. B. Unger, M. Thomas, C. Wirtz, C. Dubost, S. Handa, A. Fürstner, *Angew. Chem. Int. Ed.* **2011**, *50*, 304.

[29] J. Gallon, J. Esteban, S. Bouzbouz, M. Campbell, S. Reymond, J. Cossy, *Chem. Eur. J.* **2012**, *18*, 11788.

[30] Gaussian 09, Revision D.01, M. J. Frisch, G. W. Trucks, H. B. Schlegel, G. E. Scuseria, M. A. Robb, J. R. Cheeseman, G. Scalmani, V. Barone, B. Mennucci, G. A. Petersson, H. Nakatsuji, M. Caricato, X. Li, H. P. Hratchian, A. F. Izmaylov, J. Bloino, G. Zheng, J. L. Sonnenberg, M. Hada, M. Ehara, K. Toyota, R. Fukuda, J. Hasegawa, M. Ishida, T. Nakajima, Y. Honda, O. Kitao, H. Nakai, T. Vreven, J. A. Montgomery, Jr., J. E. Peralta, F. Ogliaro, M. Bearpark, J. J. Heyd, E. Brothers, K. N. Kudin, V. N. Staroverov, T. Keith, R. Kobayashi, J. Normand, K. Raghavachari, A. Rendell, J. C. Burant, S. S. Iyengar, J. Tomasi, M. Cossi, N. Rega, J. M. Millam, M. Klene, J. E. Knox, J. B. Cross, V. Bakken, C. Adamo, J. Jaramillo, R. Gomperts, R. E. Stratmann, O. Yazyev, A. J. Austin, R. Cammi, C. Pomelli, J. W. Ochterski, R. L. Martin, K. Morokuma, V. G. Zakrzewski, G. A. Voth, P. Salvador, J. J. Dannenberg, S. Dapprich, A. D. Daniels, O. Farkas, J. B. Foresman, J. V. Ortiz, J. Cioslowski, and D. J. Fox, Gaussian, Inc., Wallingford CT, 2013.

[31] (a) A. D. Becke, *J. Chem. Phys*. **1993**, *98*, 5648. (b) C. Lee, W. Yang, R. G. Parr, *Phys. Rev. B* **1988**, *37*, 785. (c) R. Colle, O. Salvetti, *Theor*. *Chim*. *Acta* **1975**, *37*, 329.

[32] (a) H. P. Hratchian, H. B. Schlegel, *J. Chem. Phys*. **2004**, *120*, 9918. (b) H. P. Hratchian, H. B. Schlegel, in *Theory and Applications of Computational Chemistry: The First 40 Years*, Ed. C. E. Dykstra, G. Frenking, K. S. Kim, and G. Scuseria, Elsevier, Amsterdam, 2005, 195. (c) H. P. Hratchian, H. B. Schlegel*, J. Chem. Theory and Comput*. **2005**, *1*, 61.

[33] (a) C. A. Bayse, B. D. Allison, *J. Mol. Model*. **2007**, *13*, 47. (b) J. A. Ritchey, B. M. Davis, P. A. Pleban, C. A. Bayse, *Org. Biomol. Chem*. **2005**, *3*, 4337.

[34] (a) D. Feller, *J. Comp. Chem*. **1996**, *17*, 1571. (b) K. L. Schuchardt, B. T. Didier, T. Elsethagen, L. Sun, V. Gurumoorthi, J. Chase, J. Li, T. L. Windus, *J. Chem. Inf. Model*. **2007**, *47*, 1045.

[35] M. M. Hurley, L. F. Pacios, P. A. Christiansen, R. B. Ross, W. C. Ermler, *J. Chem. Phys*. **1986**, *84*, 6840.

[36] T. H. Dunning, *J. Chem. Phys*. **1971**, *55*, 716.

[37] T. H. Dunning, *J. Chem. Phys*. **1970**, *53*, 2823.

3.1.2 Ref. [20] from Manuscript With Extended Author List

S. J. Mickel, G. H. Sedelmeier, D. Niederer, F. Schuerch, M. Seger, K. Schreiner, R. Daeffler, A. Osmani, D. Bixel, O. Loiseleur, J. Cercus , H. Stettler , K. Schaer, R. Gamboni, *Org. Process. Res. Dev.* **2004**, *8*, 113–121.

4. NMR Spectra

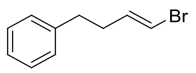

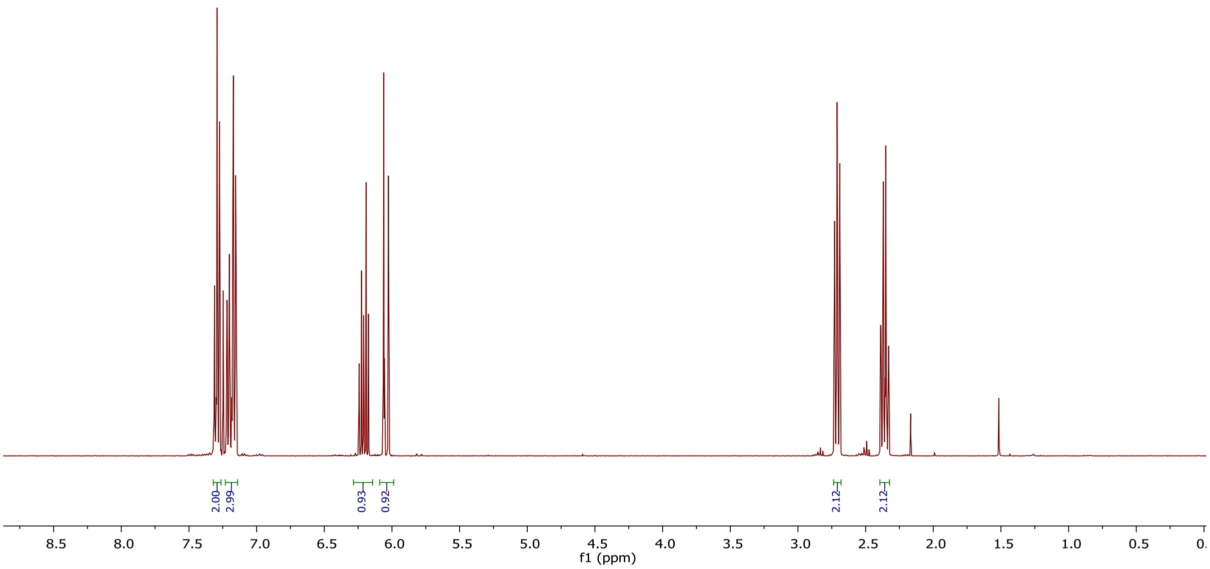

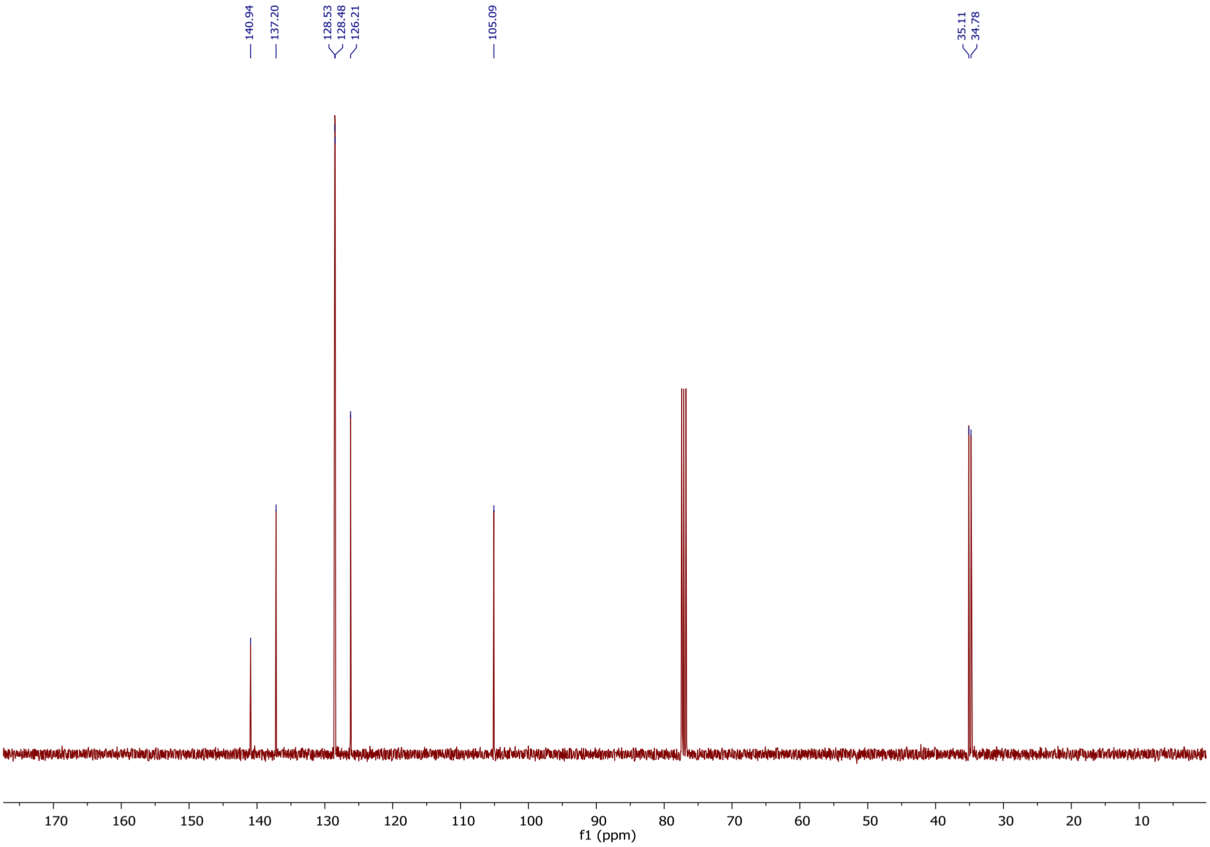

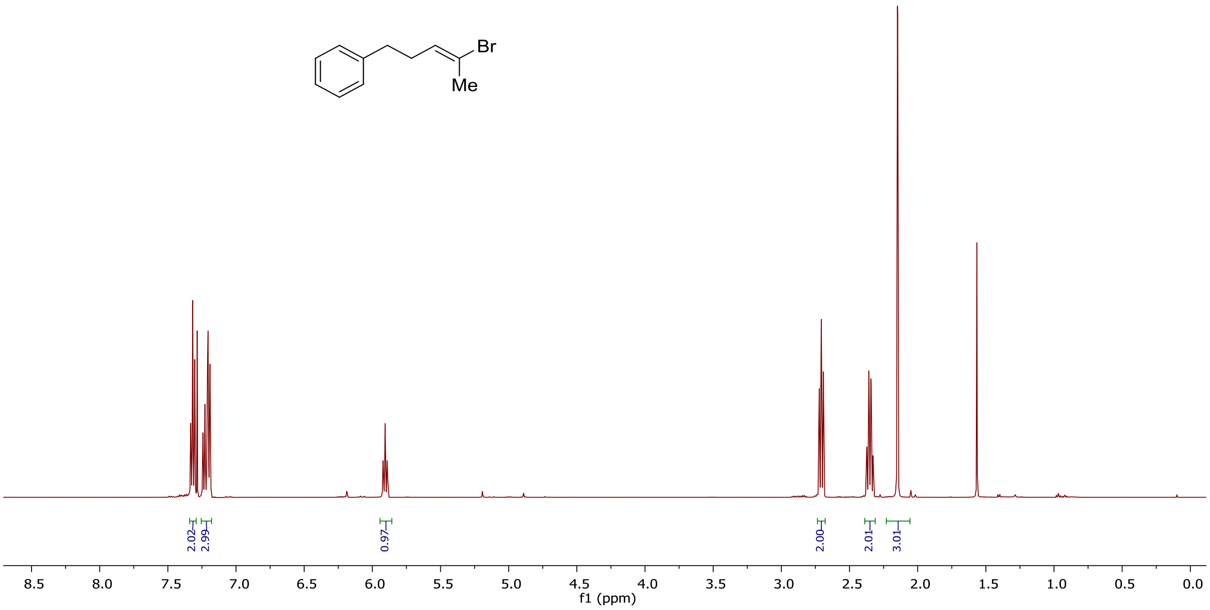
Br
Me
2.02
2.99
0.97
2.00
2.01
3.01
8.5 8.0 7.5 7.0 6.5 6.0 5.5 5.0 4.5 4.0 3.5 3.0 2.5 2.0 1.5 1.0 0.5 0.0
f1 (ppm)

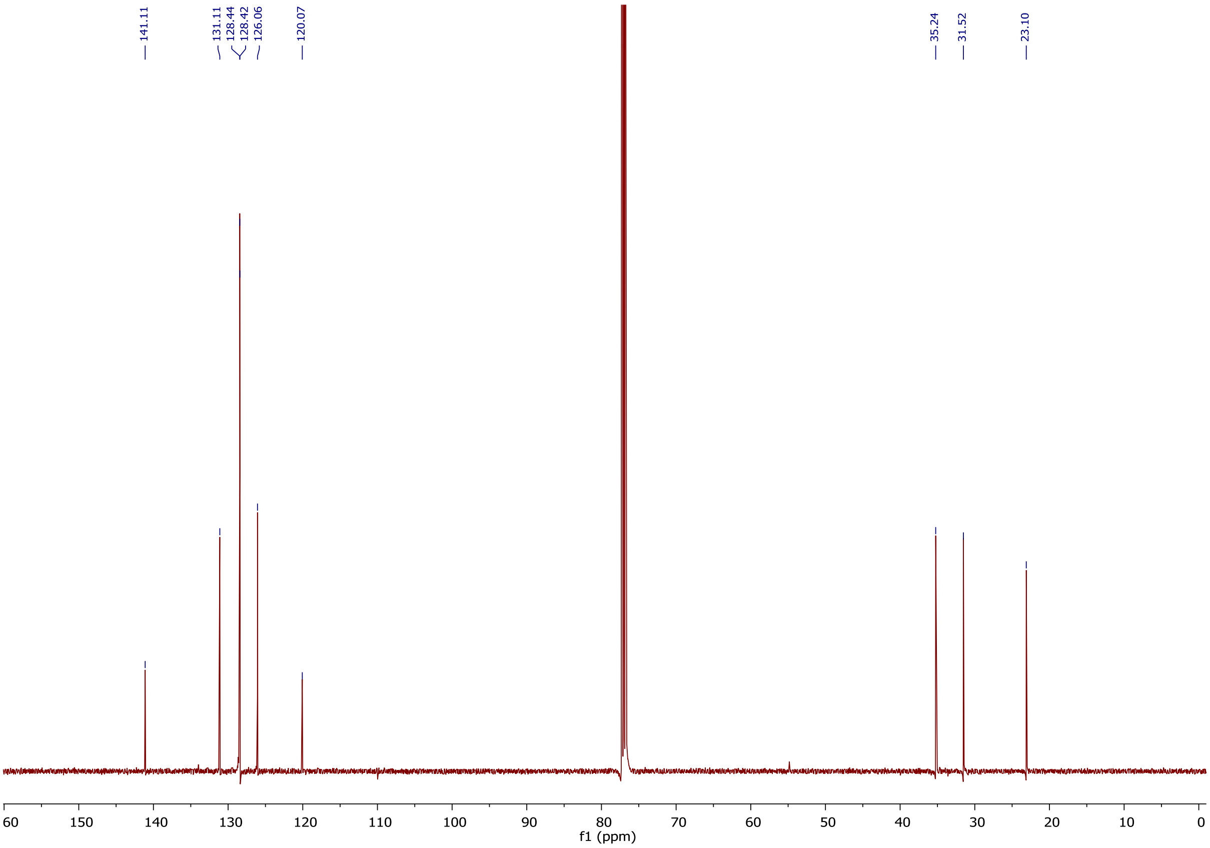
141.11
131.11
128.44
128.42
126.06
120.07
35.24
31.52
23.10
60 150 140 130 120 110 100 90 80 70 60 50 40 30 20 10 0
f1 (ppm)

1D nOe data obtained by irradiation of H_2 and H_1:

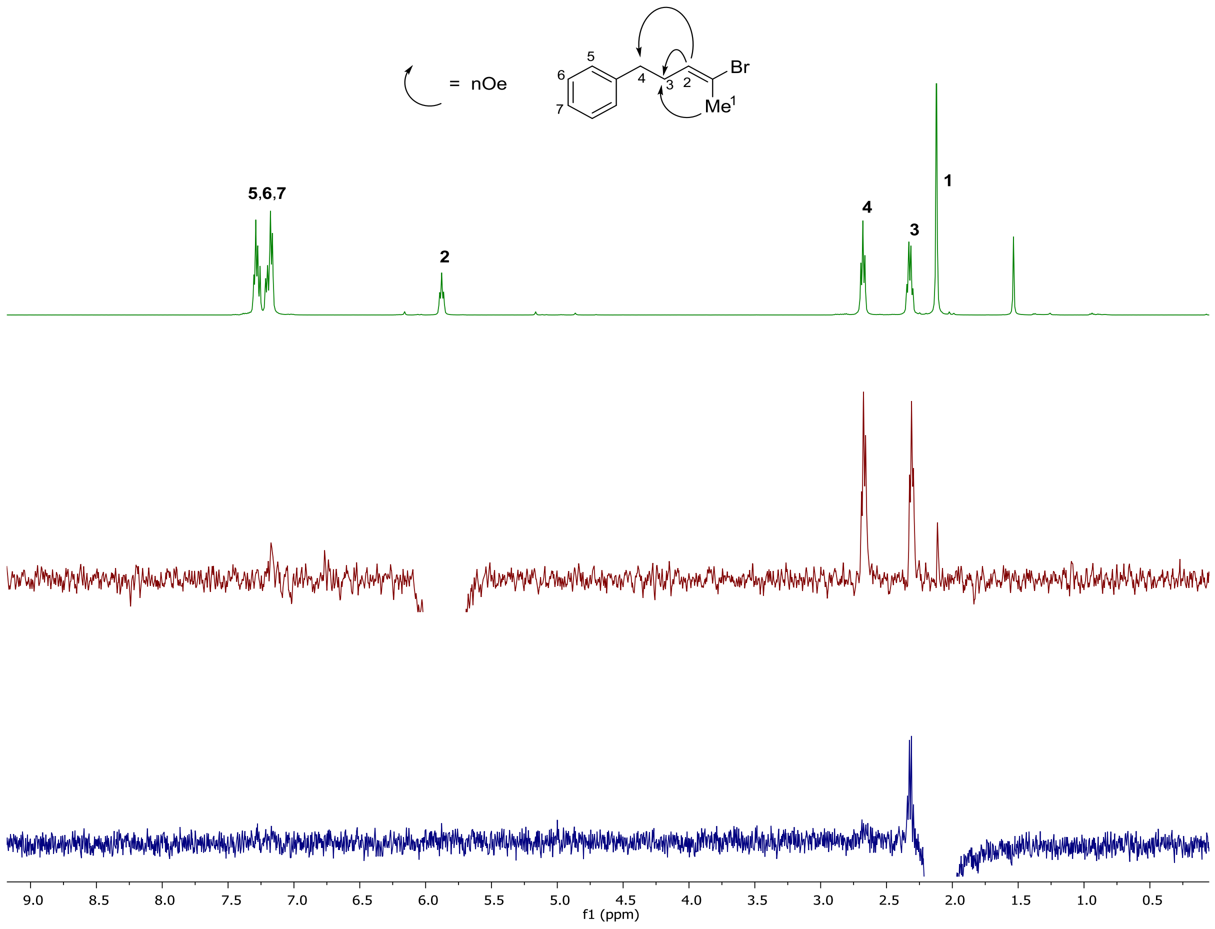

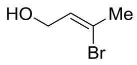
HO
Me
Br

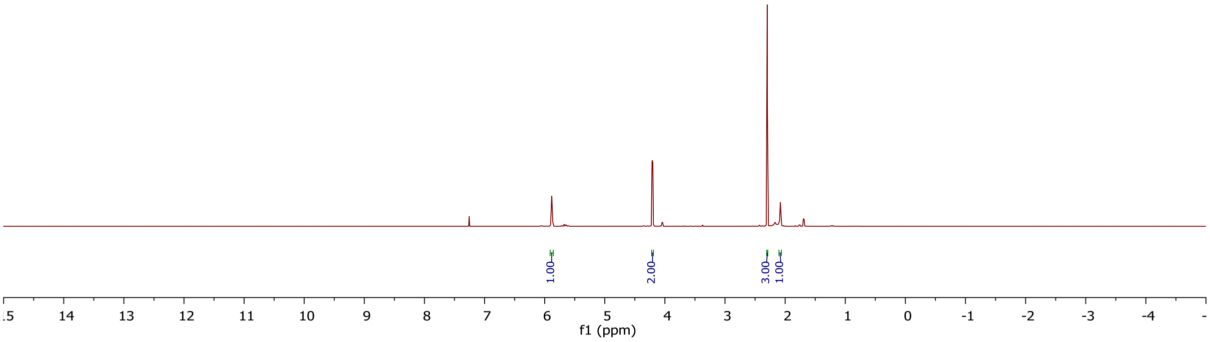
1.00
2.00
3.00
1.00
f1 (ppm)

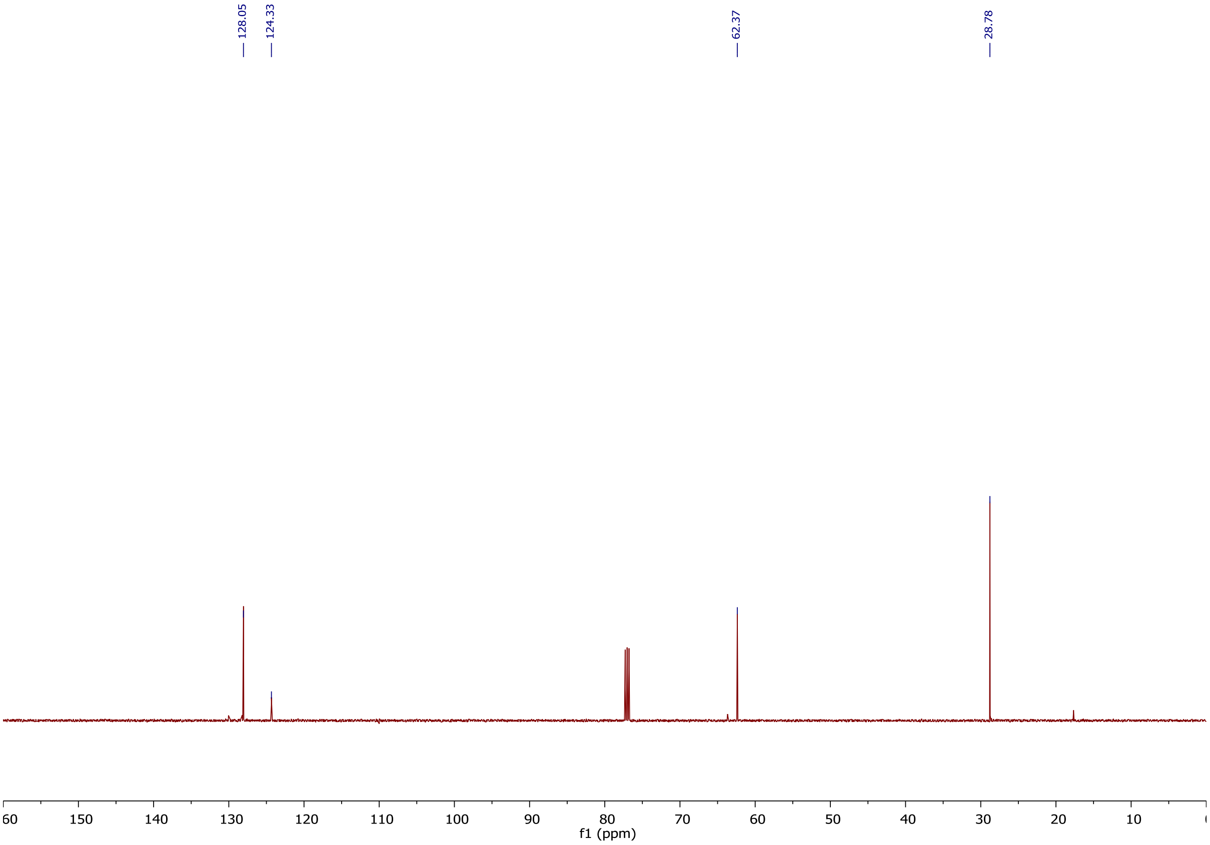
128.05
124.33
62.37
28.78
f1 (ppm)

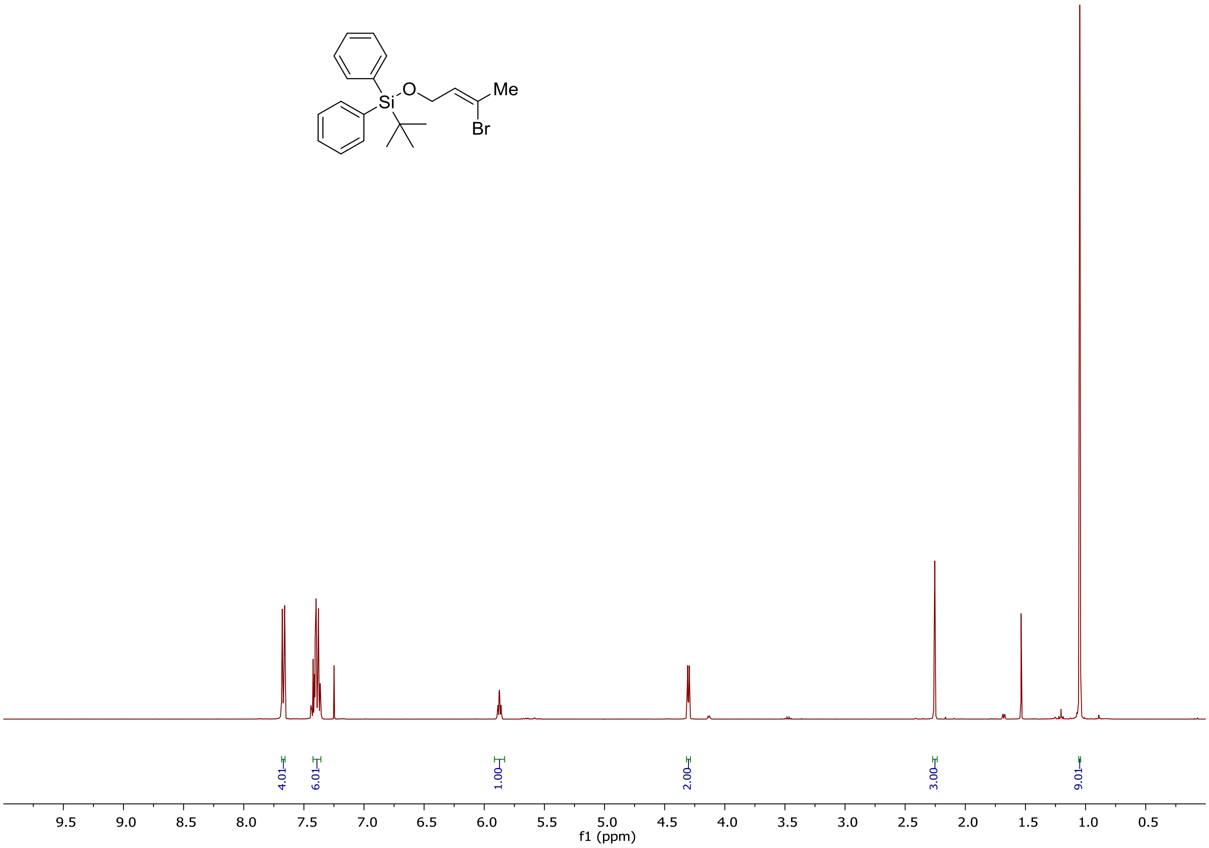
Si
O
Me
Br
4.01
6.01
1.00
2.00
3.00
9.01
f1 (ppm)

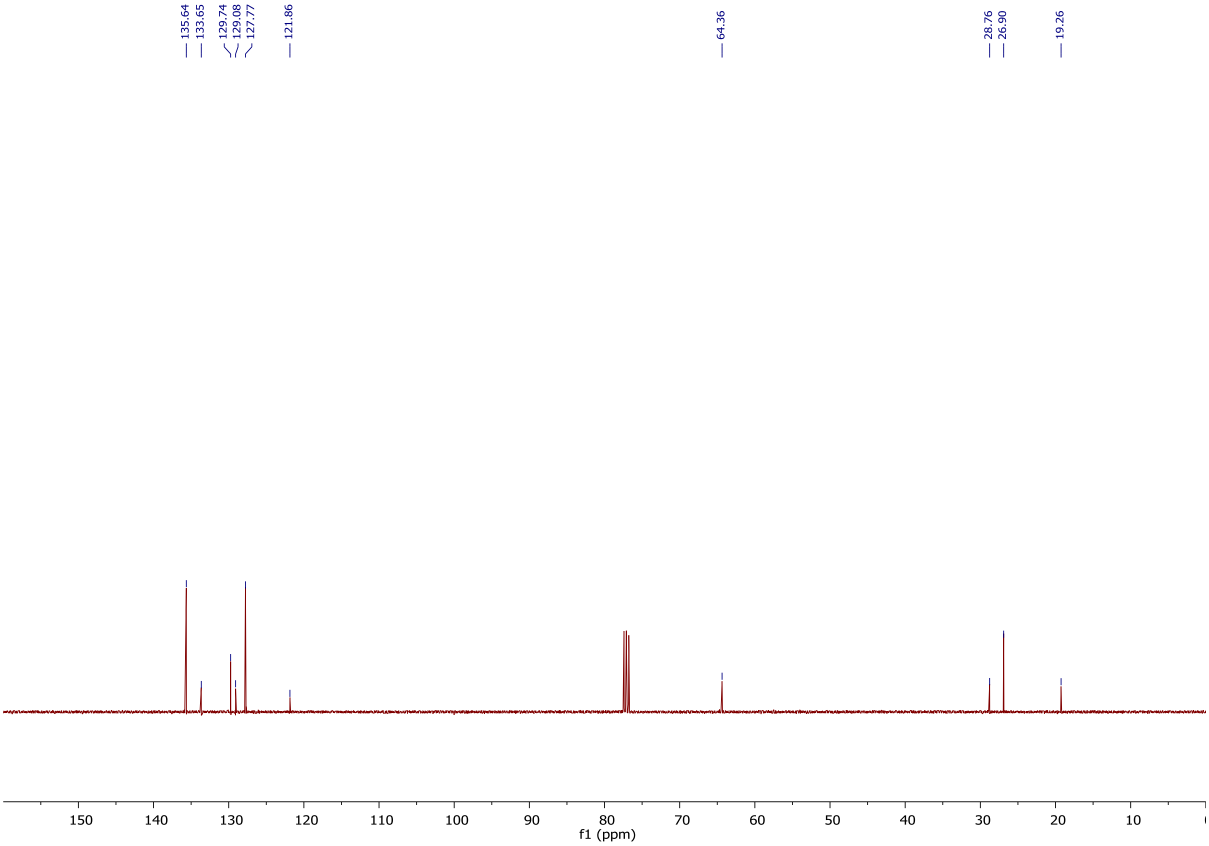
135.64
133.65
129.74
129.08
127.77
121.86
64.36
28.76
26.90
19.26
f1 (ppm)

1D nOe data obtained by irradiation of H_2:

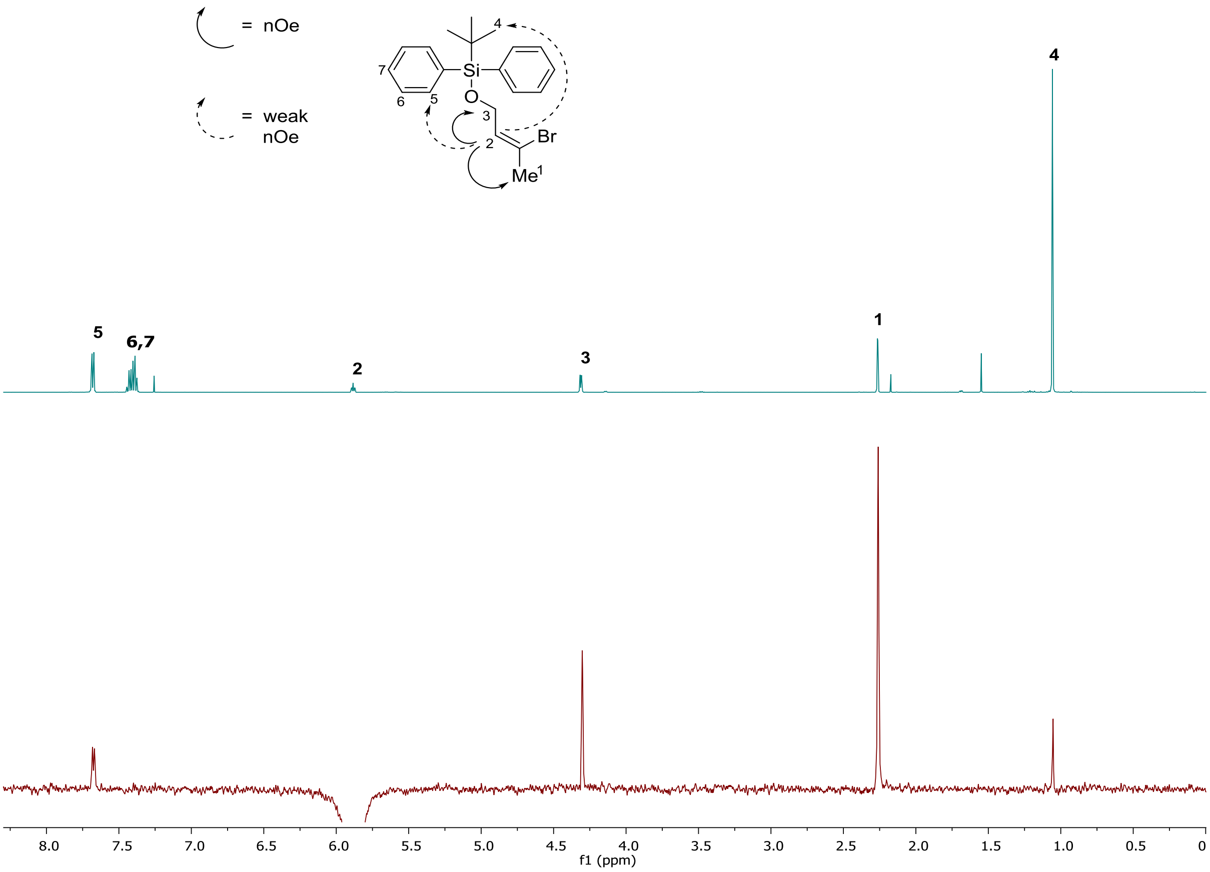

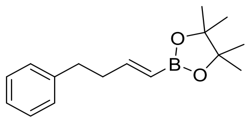
O
B
O

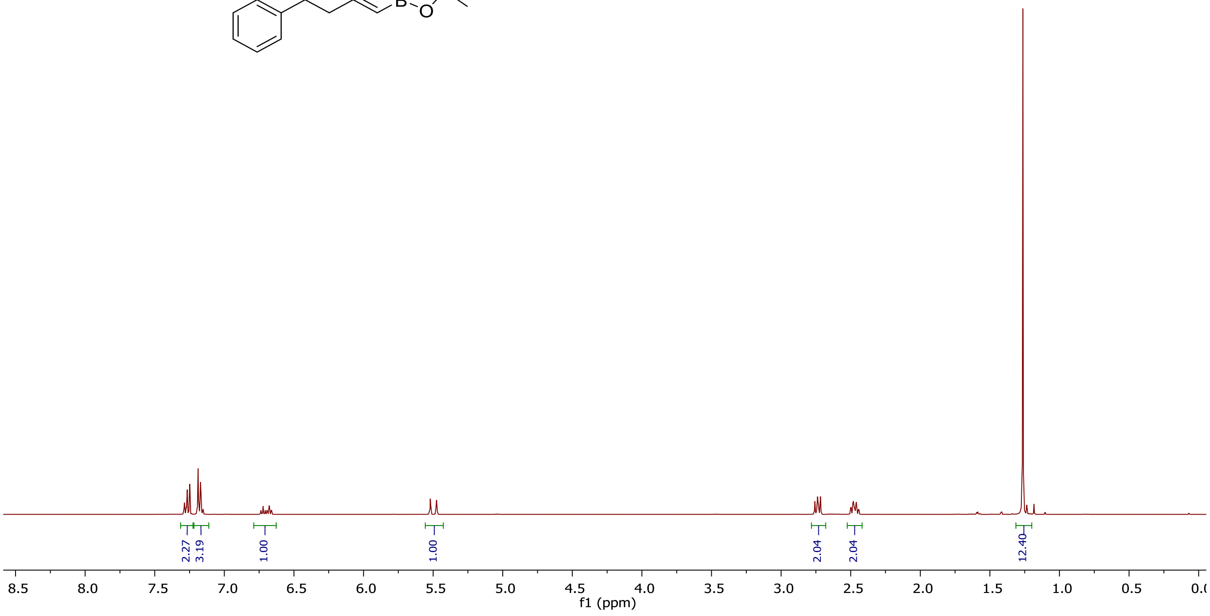
2.27
3.19
1.00
1.00
2.04
2.04
12.40
8.5 8.0 7.5 7.0 6.5 6.0 5.5 5.0 4.5 4.0 3.5 3.0 2.5 2.0 1.5 1.0 0.5
f1 (ppm)

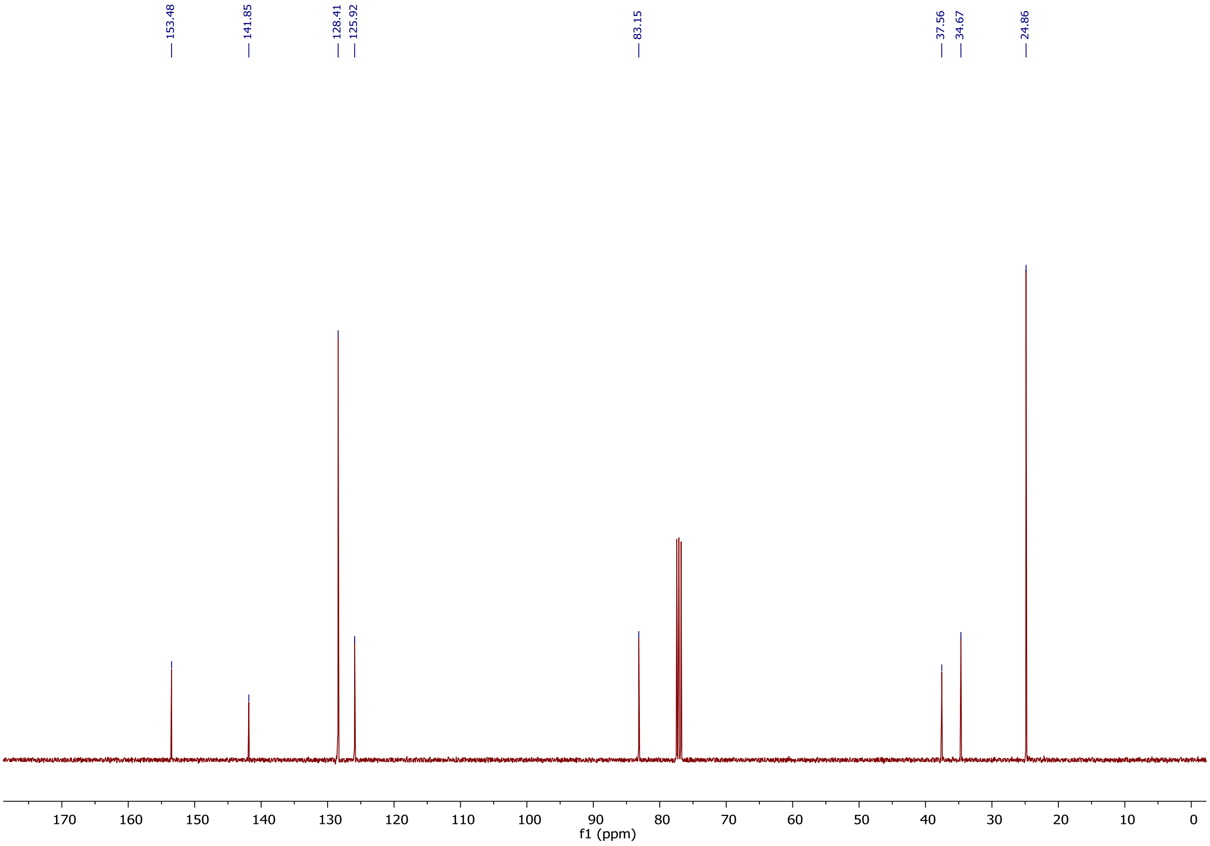
153.48
141.85
128.41
125.92
83.15
37.56
34.67
24.86
170 160 150 140 130 120 110 100 90 80 70 60 50 40 30 20 10 0
f1 (ppm)

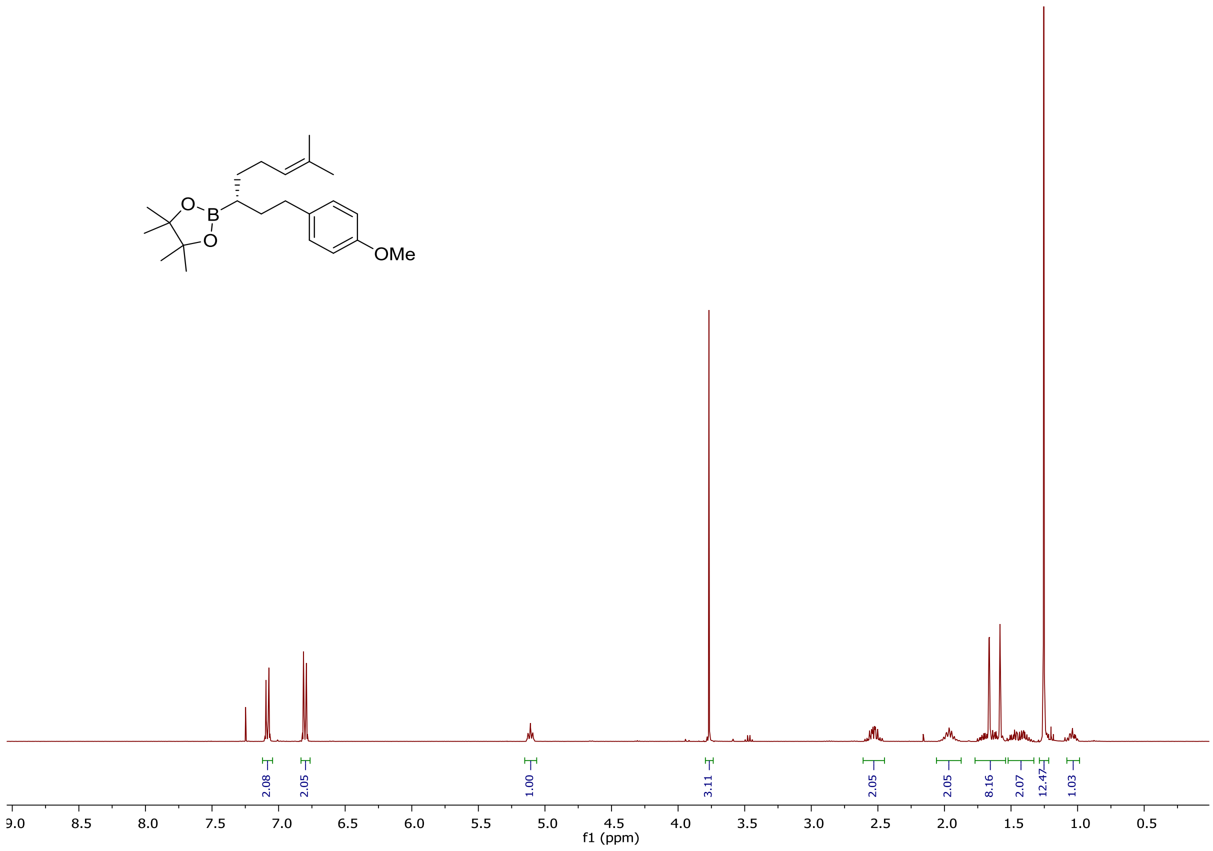
O
B
O
OMe
2.08
2.05
1.00
3.11
2.05
2.05
8.16
2.07
12.47
1.03
9.0 8.5 8.0 7.5 7.0 6.5 6.0 5.5 5.0 4.5 4.0 3.5 3.0 2.5 2.0 1.5 1.0 0.5
f1 (ppm)

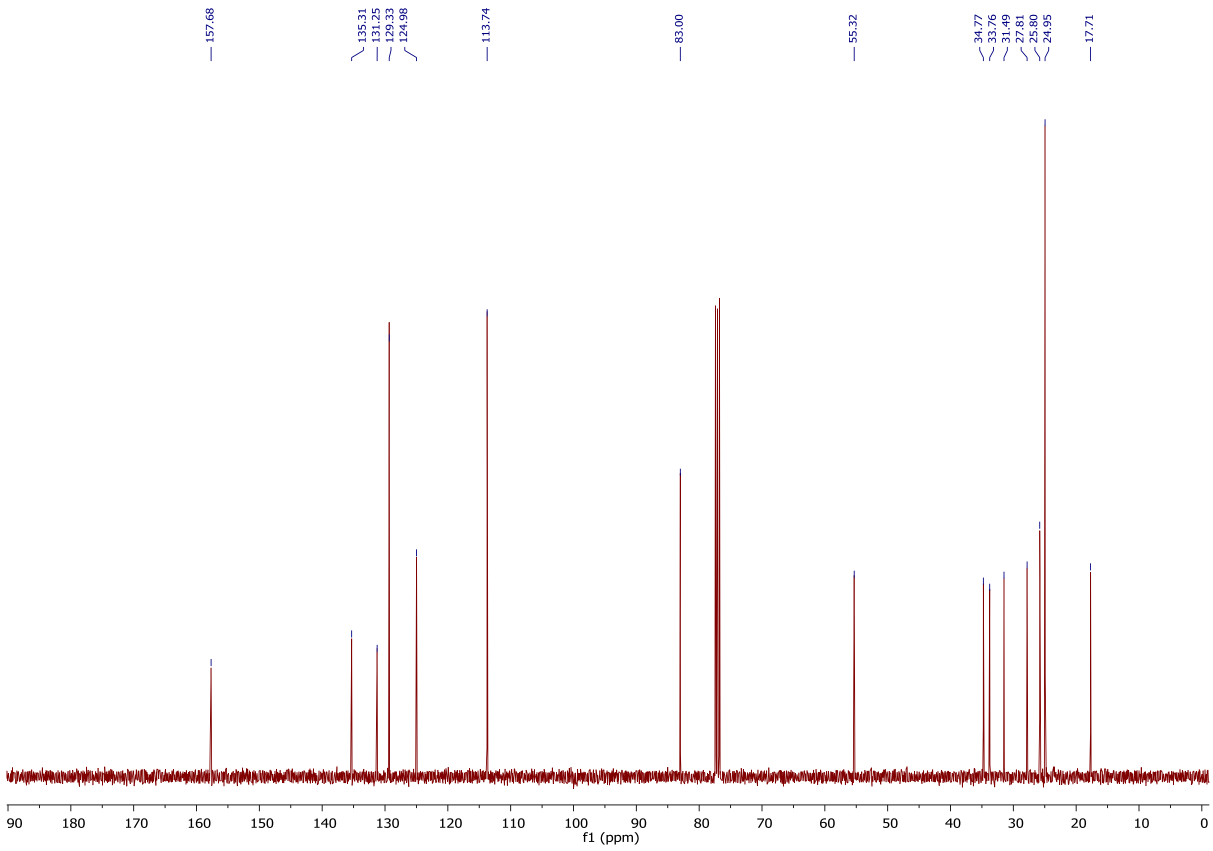
157.68
135.31
131.25
129.33
124.98
113.74
83.00
55.32
34.77
33.76
31.49
27.81
25.80
24.95
17.71
90 180 170 160 150 140 130 120 110 100 90 80 70 60 50 40 30 20 10 0
f1 (ppm)

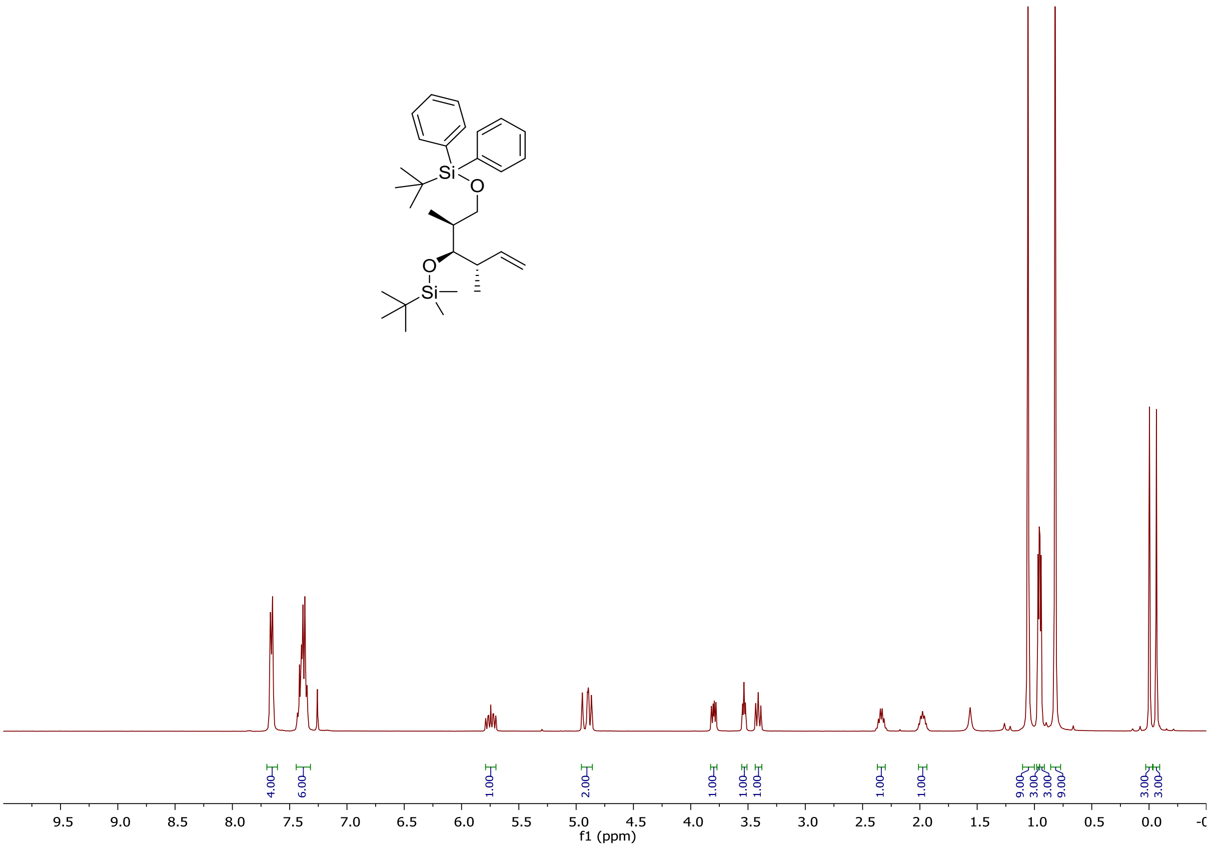
Si
O
O
Si
4.00
6.00
1.00
2.00
1.00
1.00
1.00
1.00
1.00
9.00
3.00
3.00
9.00
3.00
3.00
9.5 9.0 8.5 8.0 7.5 7.0 6.5 6.0 5.5 5.0 4.5 4.0 3.5 3.0 2.5 2.0 1.5 1.0 0.5 0.0
f1 (ppm)

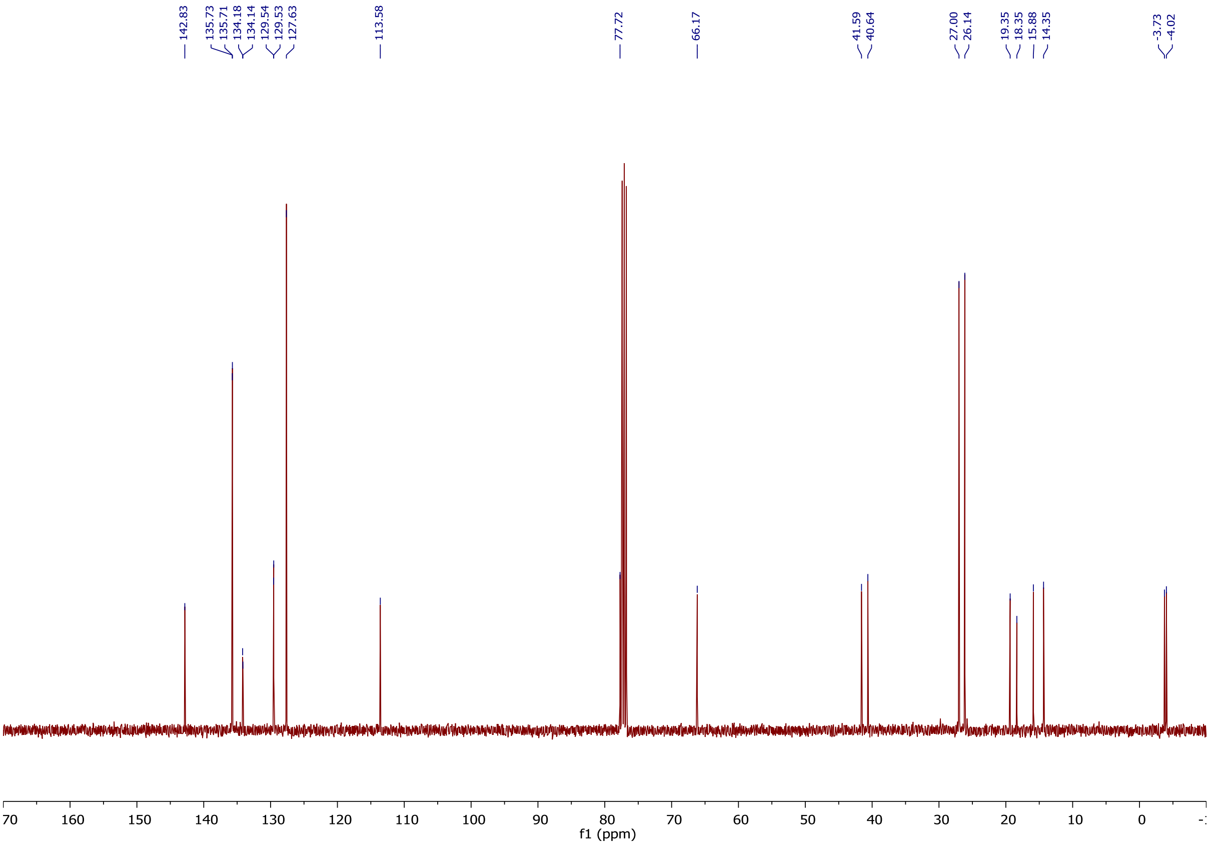
142.83
135.73
135.71
134.18
134.14
129.54
129.53
127.63
113.58
77.72
66.17
41.59
40.64
27.00
26.14
19.35
18.35
15.88
14.35
-3.73
-4.02
160 150 140 130 120 110 100 90 80 70 60 50 40 30 20 10 0
f1 (ppm)

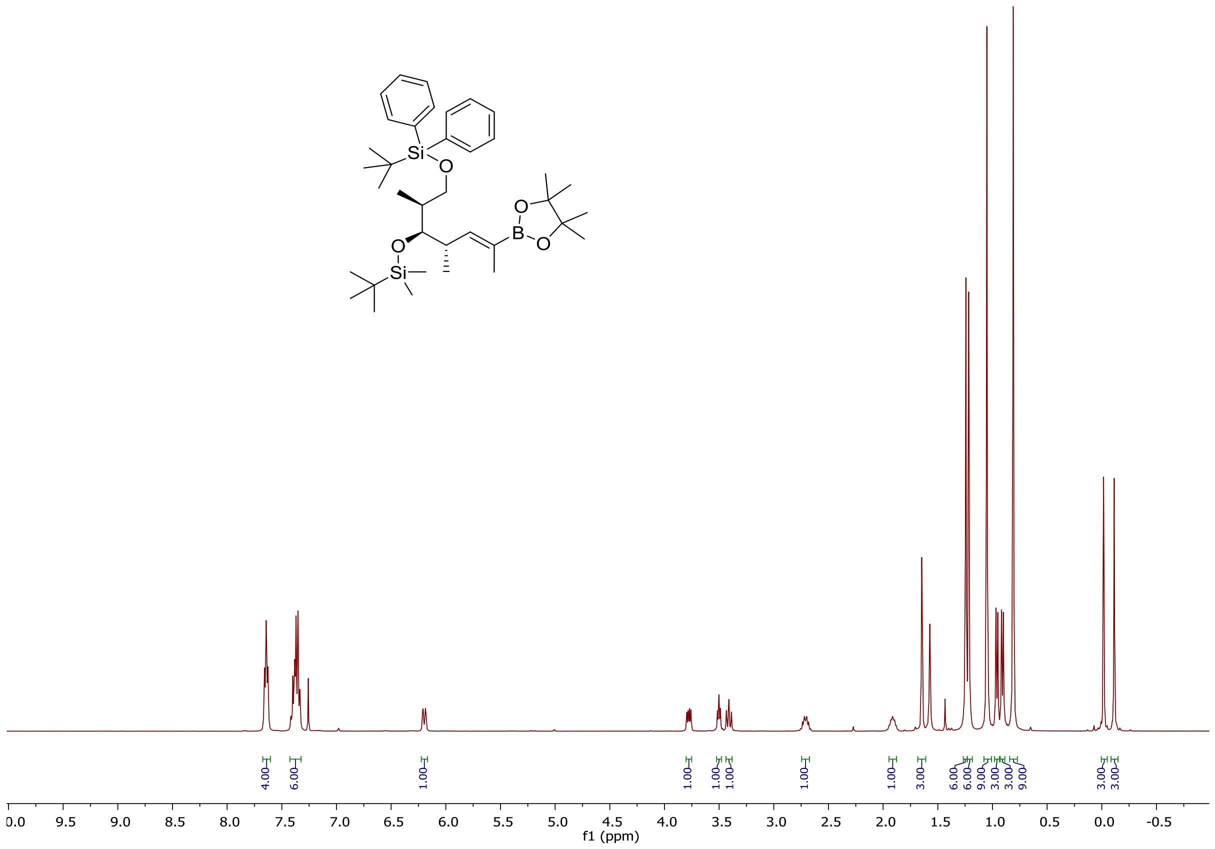

Si
O
O
Si
B
O
O
4.00
6.00
1.00
1.00
1.00
1.00
1.00
1.00
3.00
6.00
6.00
9.00
3.00
3.00
9.00
3.00
3.00
f1 (ppm)

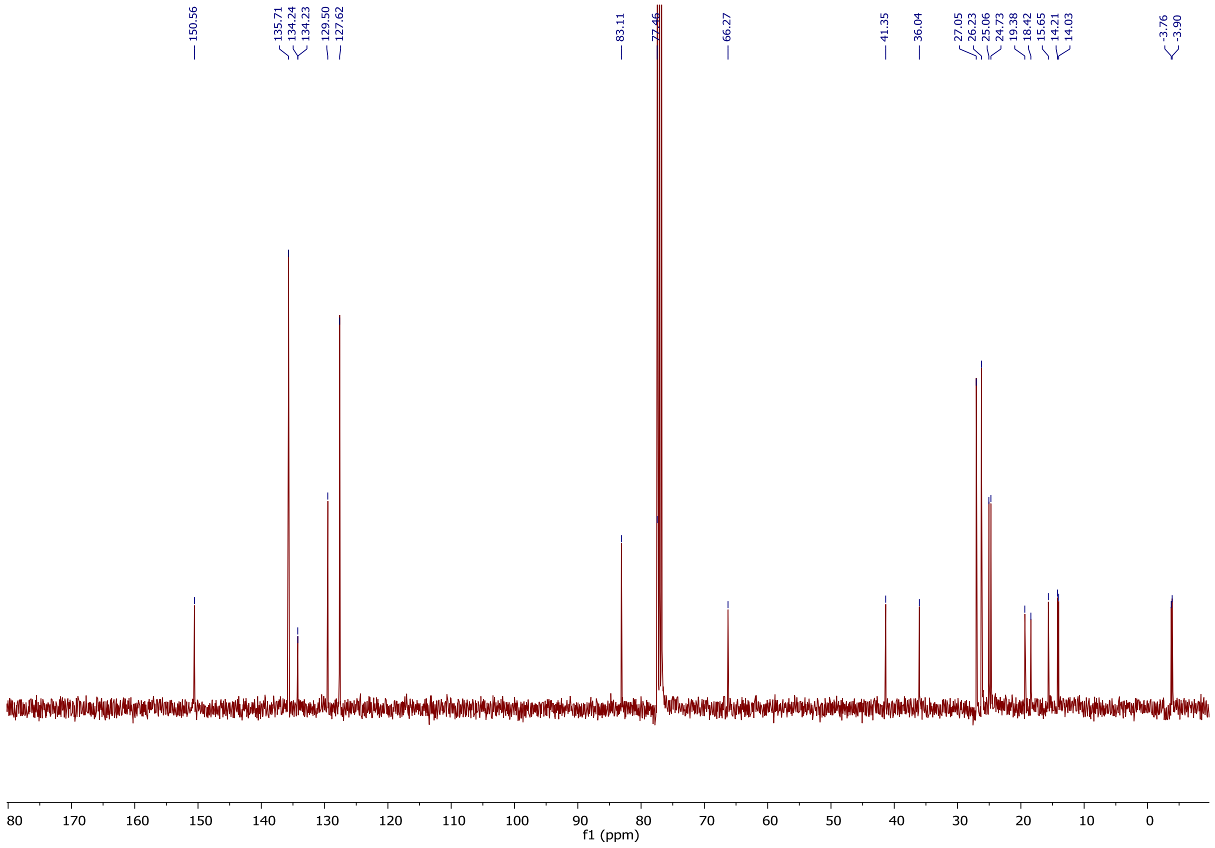

150.56
135.71
134.24
134.23
129.50
127.62
83.11
77.46
66.27
41.35
36.04
27.05
26.23
25.06
24.73
19.38
18.42
15.65
14.21
14.03
-3.76
-3.90
f1 (ppm)

1D nOe data obtained by irradiation of H_2 and H_3:

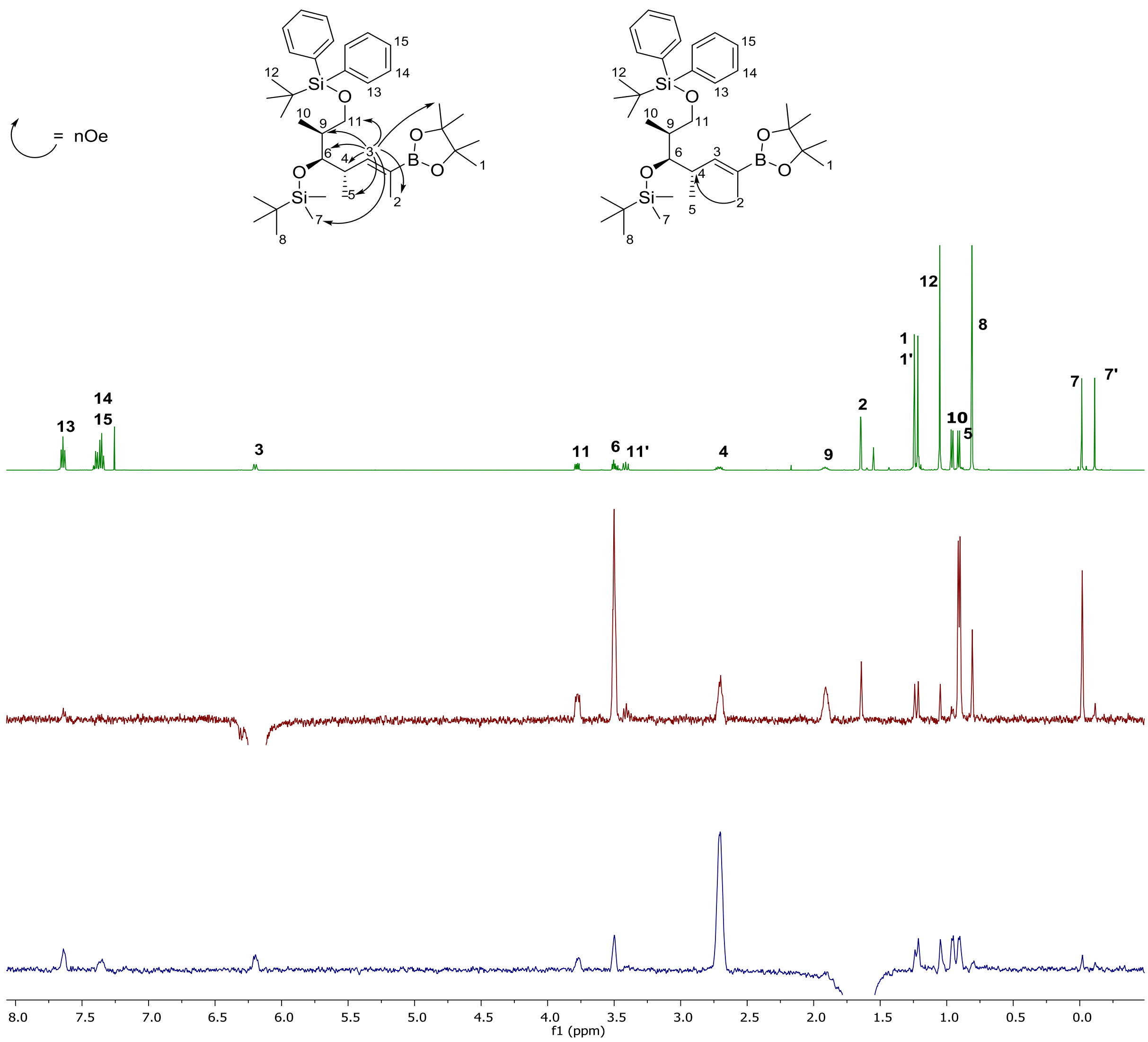

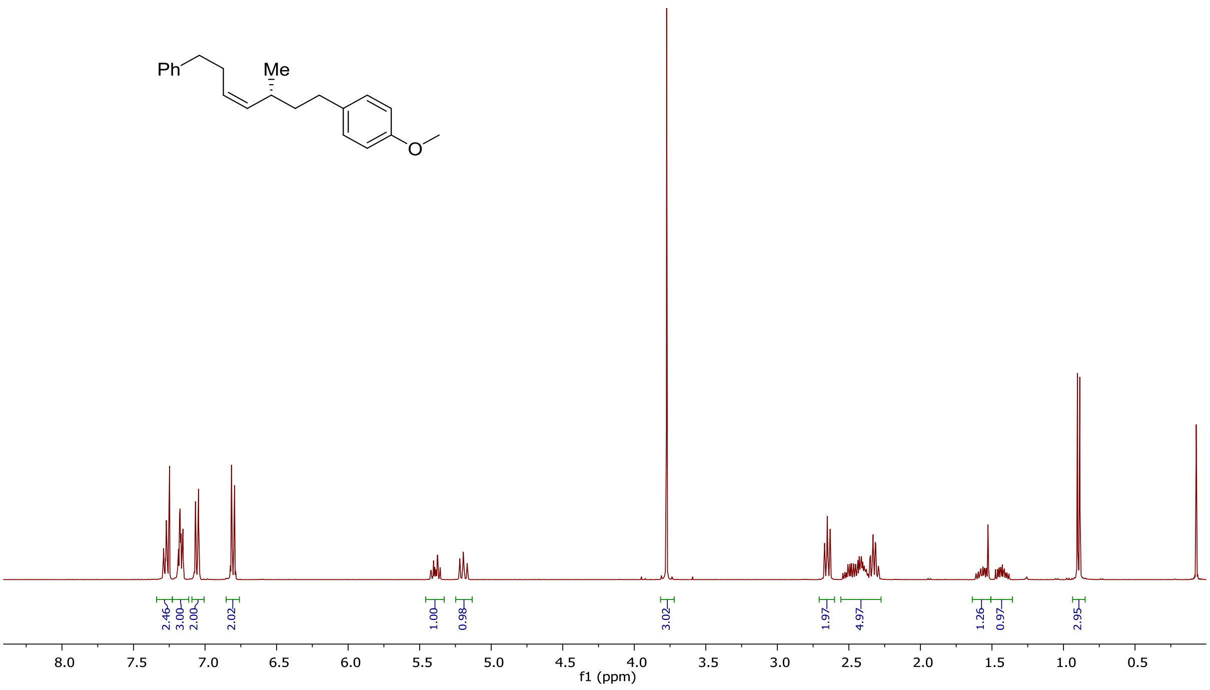
Ph
Me
O
2.46
3.00
2.00
2.02
1.00
0.98
3.02
1.97
4.97
1.26
0.97
2.95
8.0 7.5 7.0 6.5 6.0 5.5 5.0 4.5 4.0 3.5 3.0 2.5 2.0 1.5 1.0 0.5
f1 (ppm)

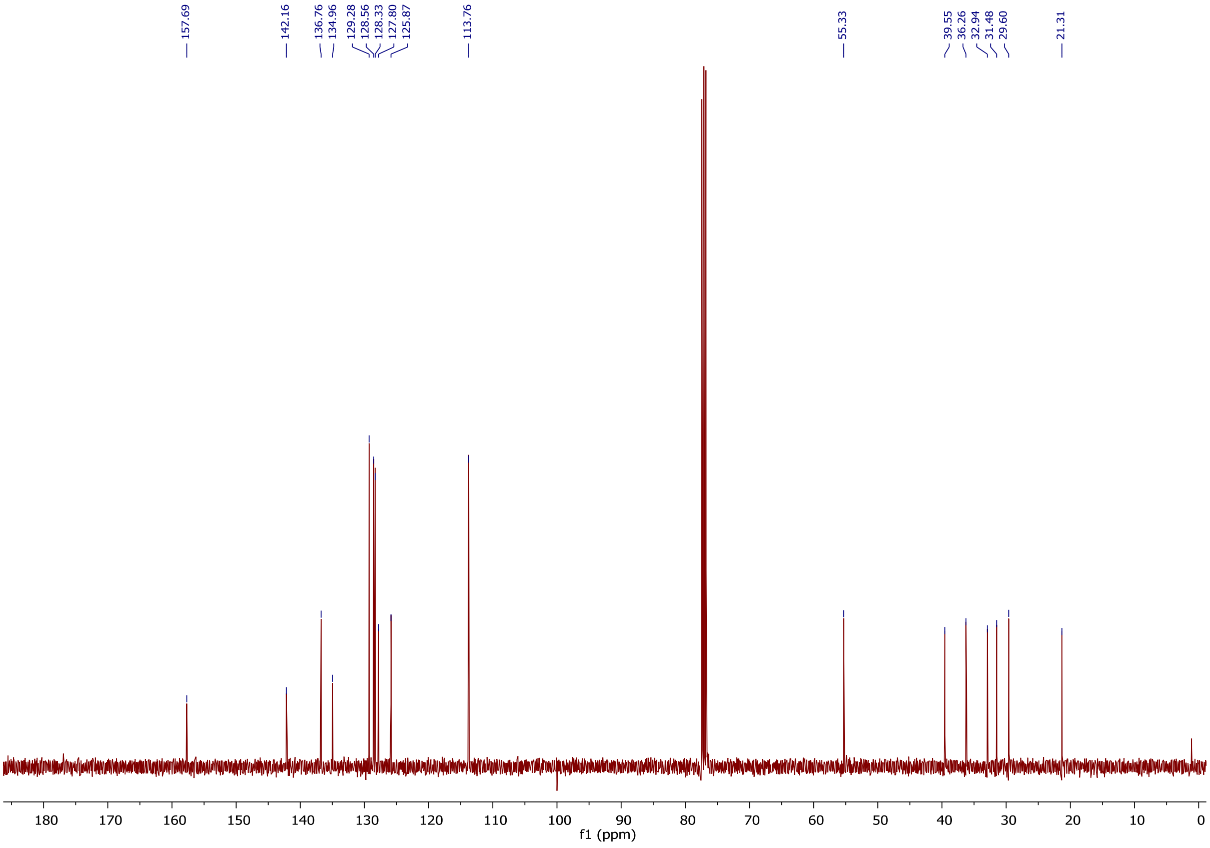
157.69
142.16
136.76
134.96
129.28
128.56
128.33
127.80
125.87
113.76
55.33
39.55
36.26
32.94
31.48
29.60
21.31
180 170 160 150 140 130 120 110 100 90 80 70 60 50 40 30 20 10 0
f1 (ppm)

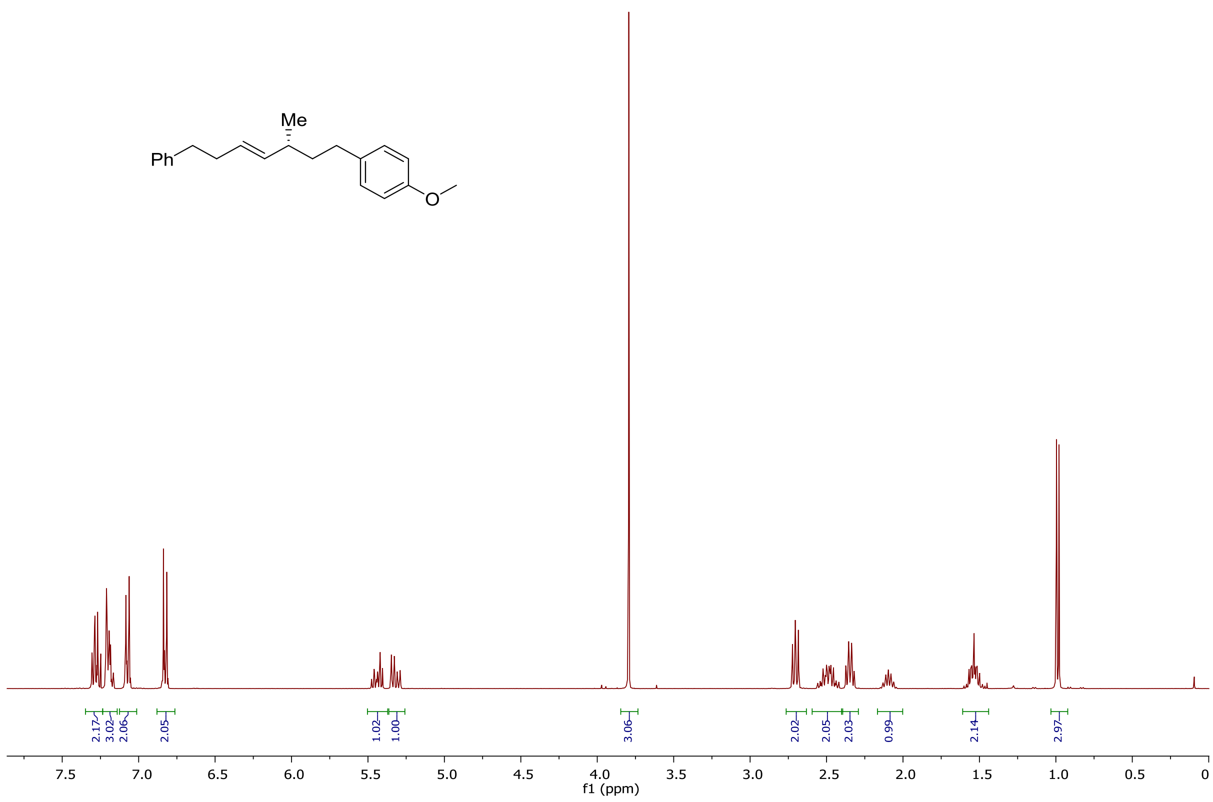
Me
Ph
O
2.17
3.02
2.06
2.05
1.02
1.00
3.06
2.02
2.05
2.03
0.99
2.14
2.97
7.5
7.0
6.5
6.0
5.5
5.0
4.5
4.0
3.5
3.0
2.5
2.0
1.5
1.0
0.5
0
f1 (ppm)

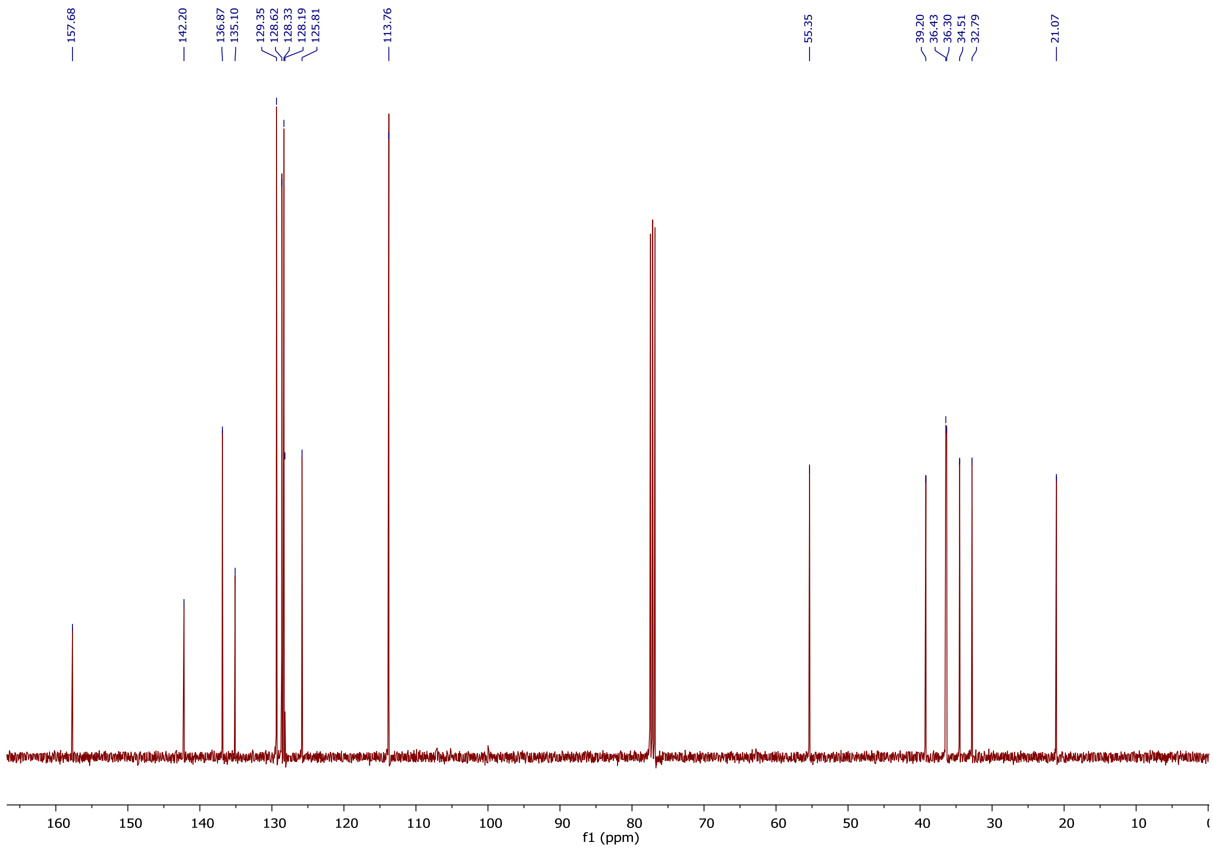
157.68
142.20
136.87
135.10
129.35
128.62
128.33
128.19
125.81
113.76
55.35
39.20
36.43
36.30
34.51
32.79
21.07
160
150
140
130
120
110
100
90
80
70
60
50
40
30
20
10
f1 (ppm)

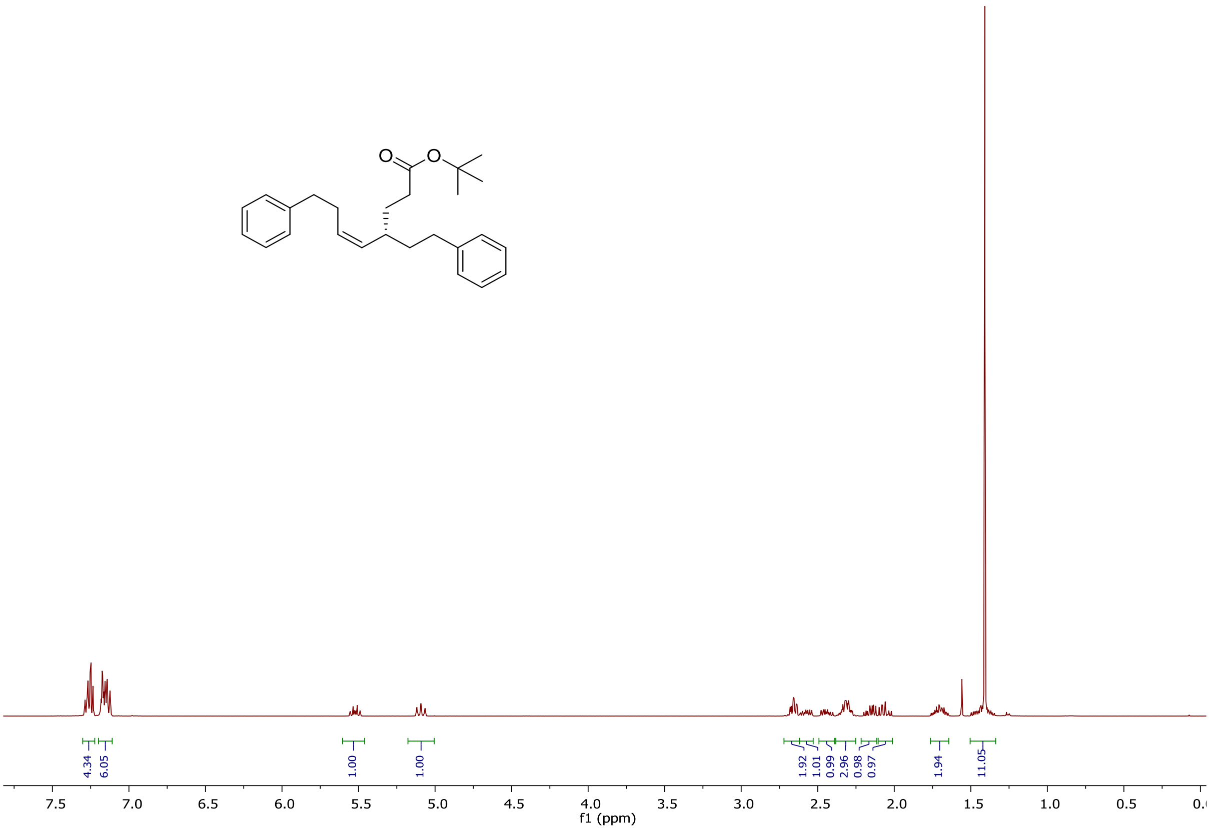

4.34
6.05
1.00
1.00
1.92
1.01
0.99
2.96
0.98
0.97
1.94
11.05
7.5 7.0 6.5 6.0 5.5 5.0 4.5 4.0 3.5 3.0 2.5 2.0 1.5 1.0 0.5
f1 (ppm)

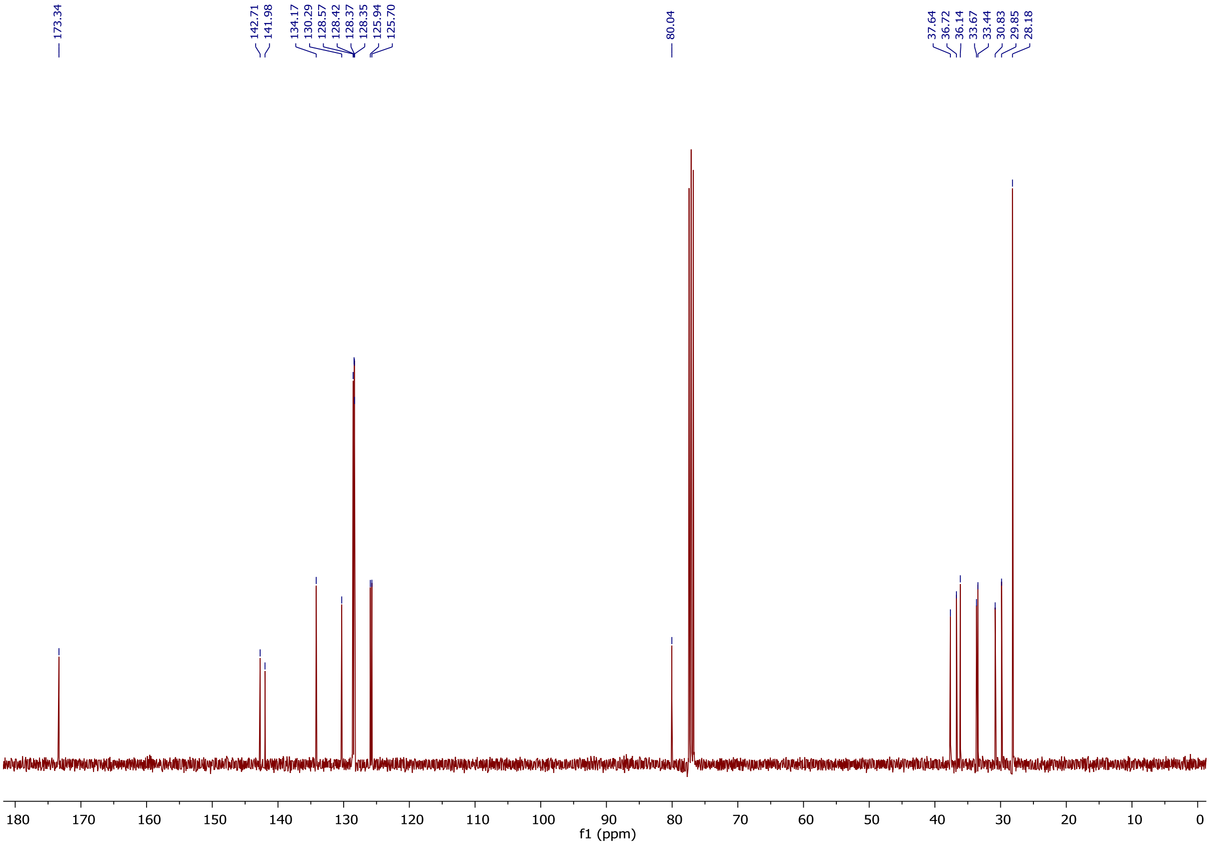

173.34
142.71
141.98
134.17
130.29
128.57
128.42
128.37
128.35
125.94
125.70
80.04
37.64
36.72
36.14
33.67
33.44
30.83
29.85
28.18
180 170 160 150 140 130 120 110 100 90 80 70 60 50 40 30 20 10 0
f1 (ppm)

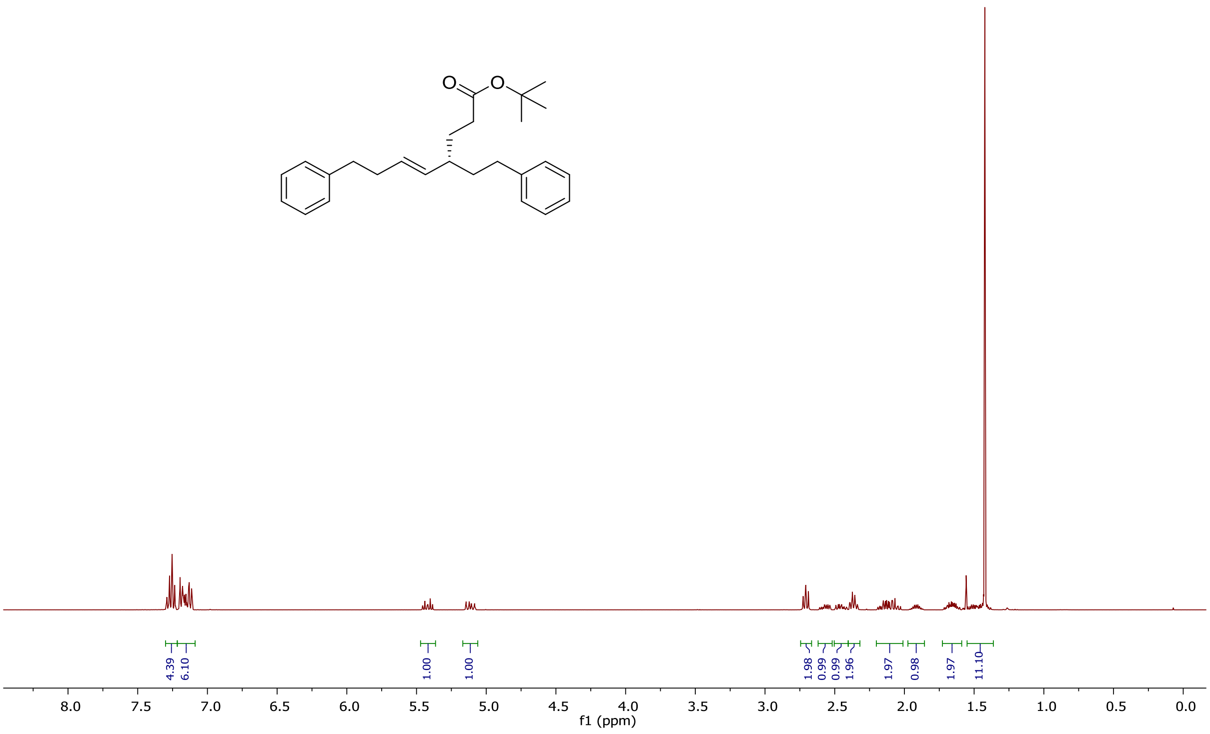
4.39
6.10
1.00
1.00
1.98
0.99
0.99
1.96
1.97
0.98
1.97
11.10
8.0 7.5 7.0 6.5 6.0 5.5 5.0 4.5 4.0 3.5 3.0 2.5 2.0 1.5 1.0 0.5 0.0
f1 (ppm)

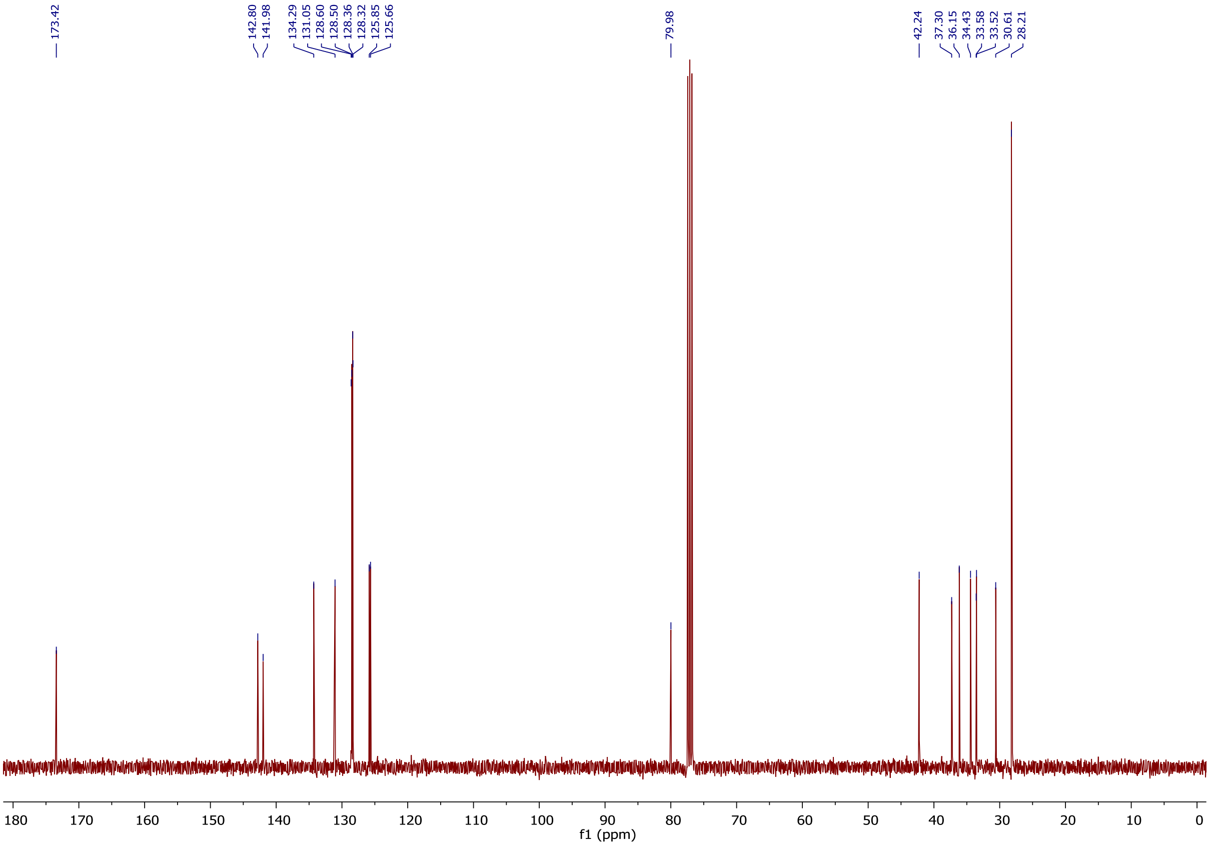
173.42
142.80
141.98
134.29
131.05
128.60
128.50
128.36
128.32
125.85
125.66
79.98
42.24
37.30
36.15
34.43
33.58
33.52
30.61
28.21
180 170 160 150 140 130 120 110 100 90 80 70 60 50 40 30 20 10 0
f1 (ppm)

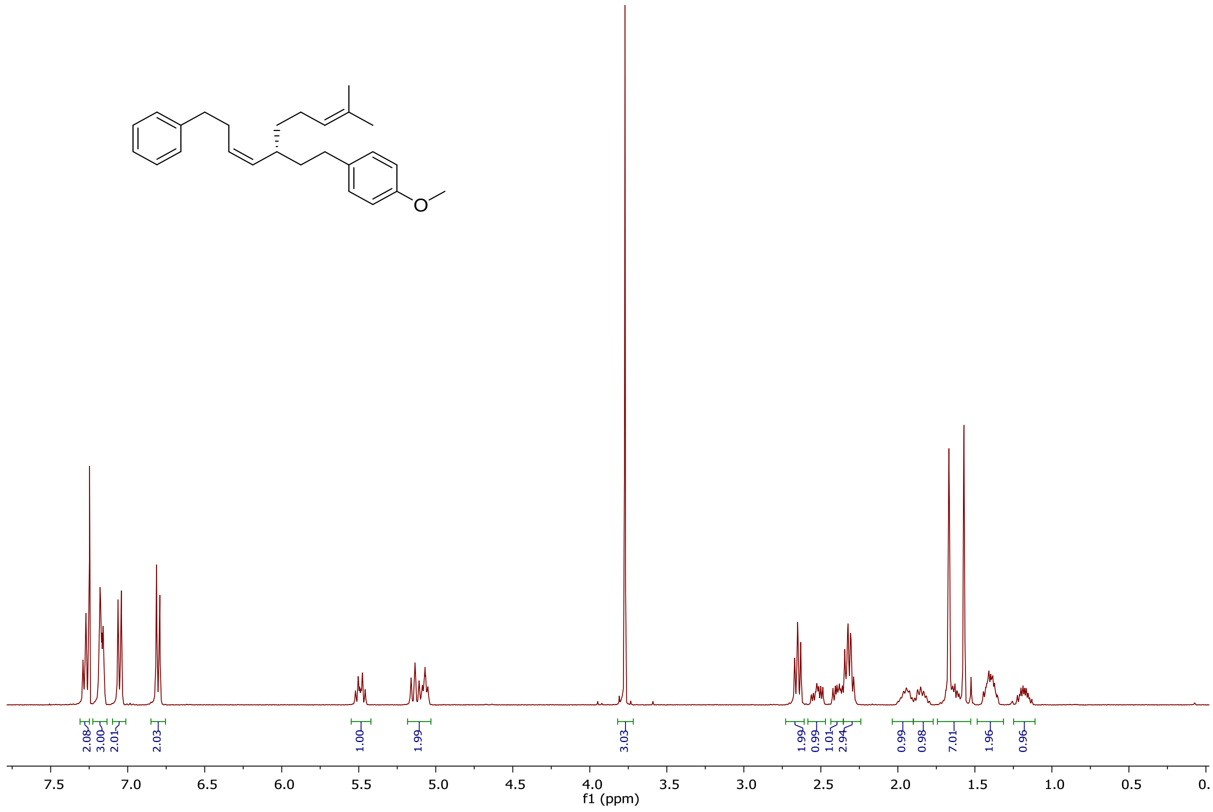
O
2.08
3.00
2.01
2.03
1.00
1.99
3.03
1.99
0.99
1.01
2.94
0.99
0.98
7.01
1.96
0.96
f1 (ppm)

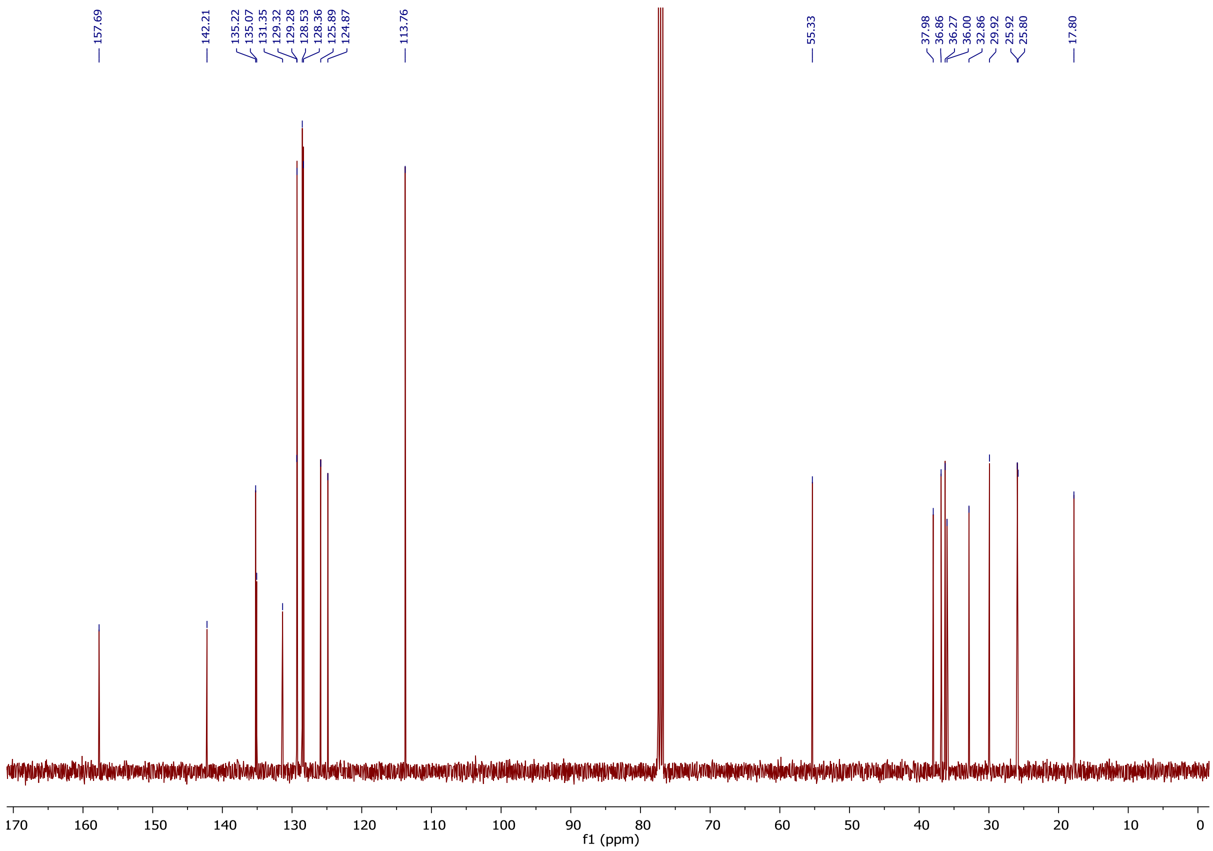
157.69
142.21
135.22
135.07
131.35
129.32
129.28
128.53
128.36
125.89
124.87
113.76
55.33
37.98
36.86
36.27
36.00
32.86
29.92
25.92
25.80
17.80
f1 (ppm)

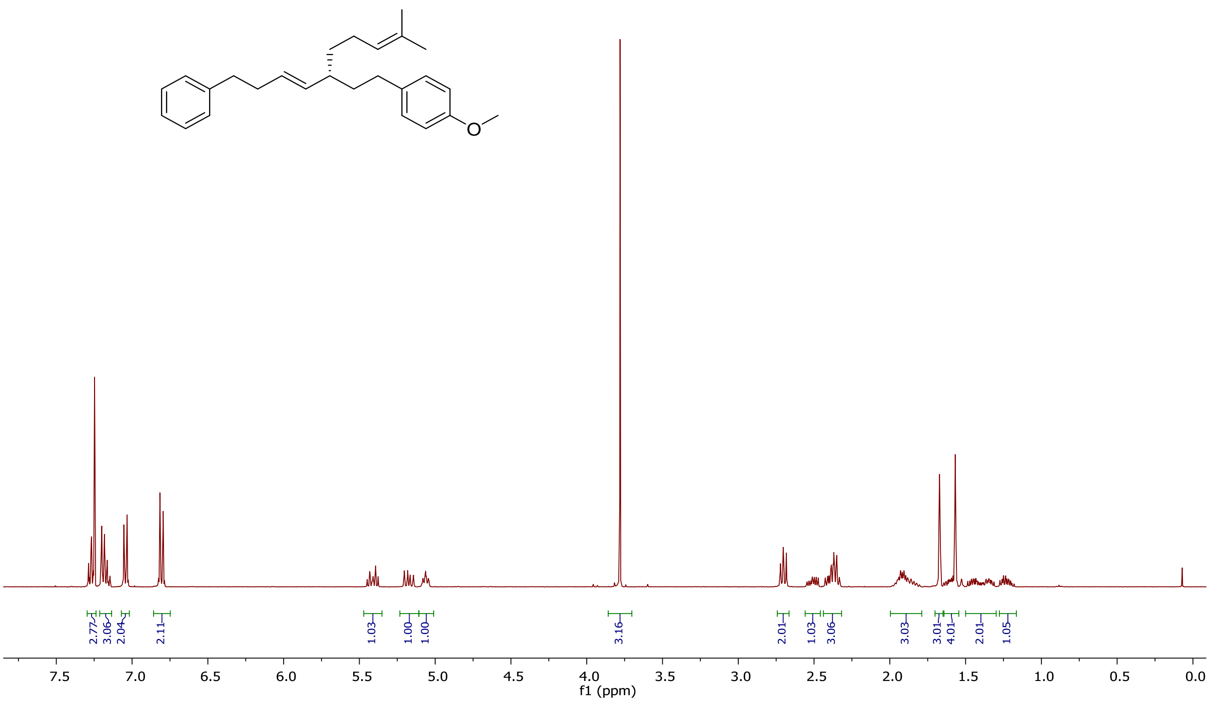
2.77 3.06 2.04 2.11 1.03 1.00 1.00 3.16 2.01 1.03 3.06 3.03 3.01 4.01 2.01 1.05
7.5 7.0 6.5 6.0 5.5 5.0 4.5 4.0 3.5 3.0 2.5 2.0 1.5 1.0 0.5 0.0
f1 (ppm)

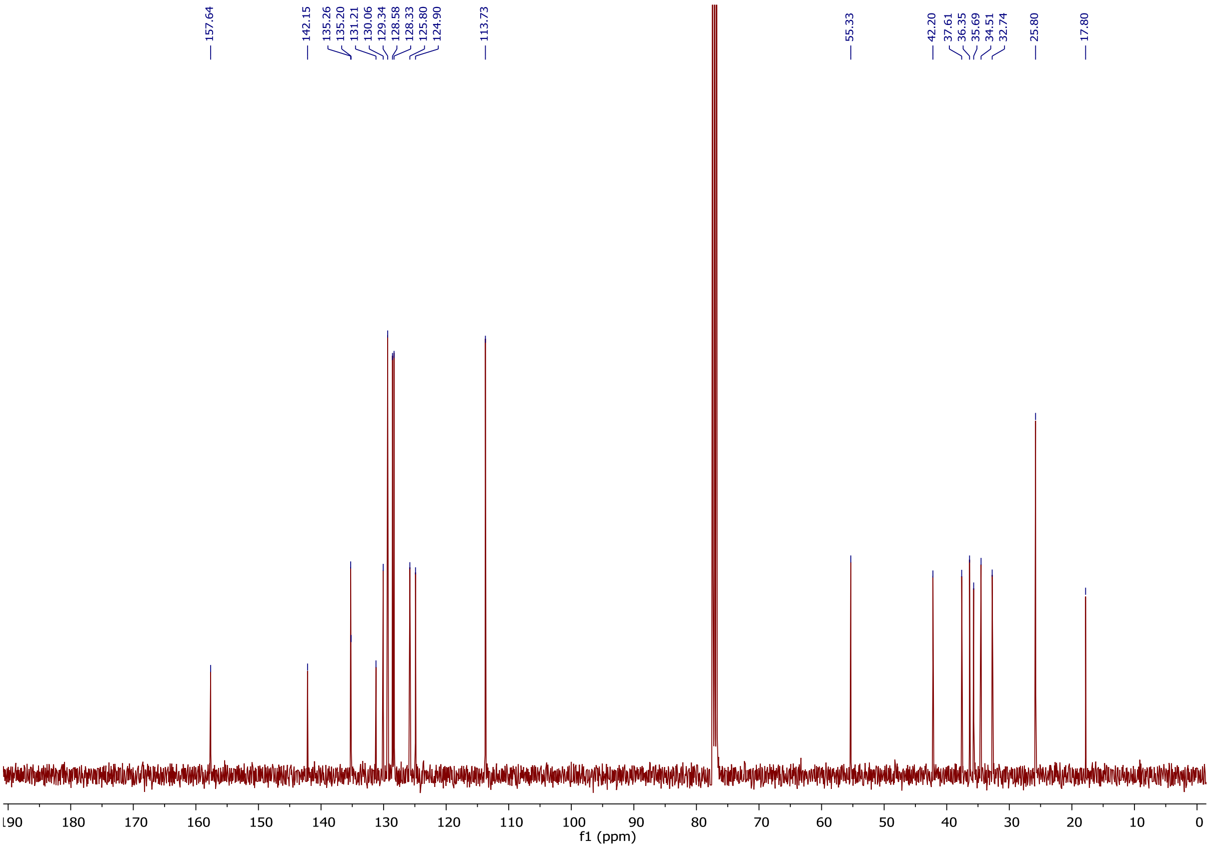
157.64
142.15 135.26 135.20 131.21 130.06 129.34 128.58 128.33 125.80 124.90
113.73
55.33
42.20 37.61 36.35 35.69 34.51 32.74
25.80
17.80
190 180 170 160 150 140 130 120 110 100 90 80 70 60 50 40 30 20 10 0
f1 (ppm)

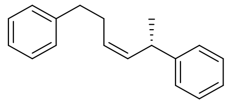

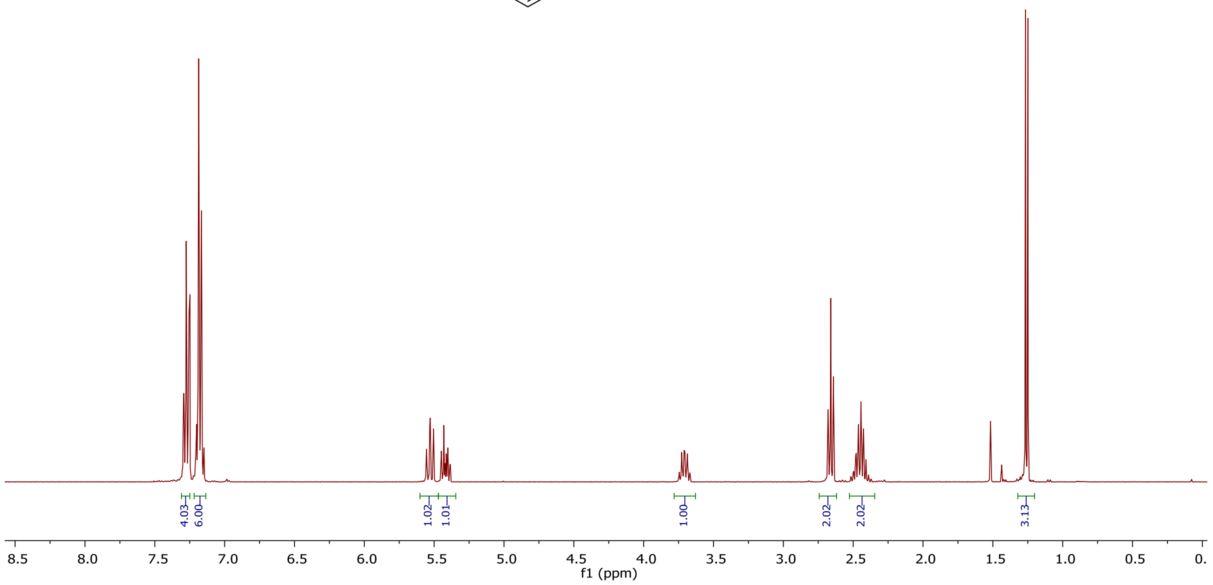
4.03
6.00
1.02
1.01
1.00
2.02
2.02
3.13
8.5 8.0 7.5 7.0 6.5 6.0 5.5 5.0 4.5 4.0 3.5 3.0 2.5 2.0 1.5 1.0 0.5 0.
f1 (ppm)

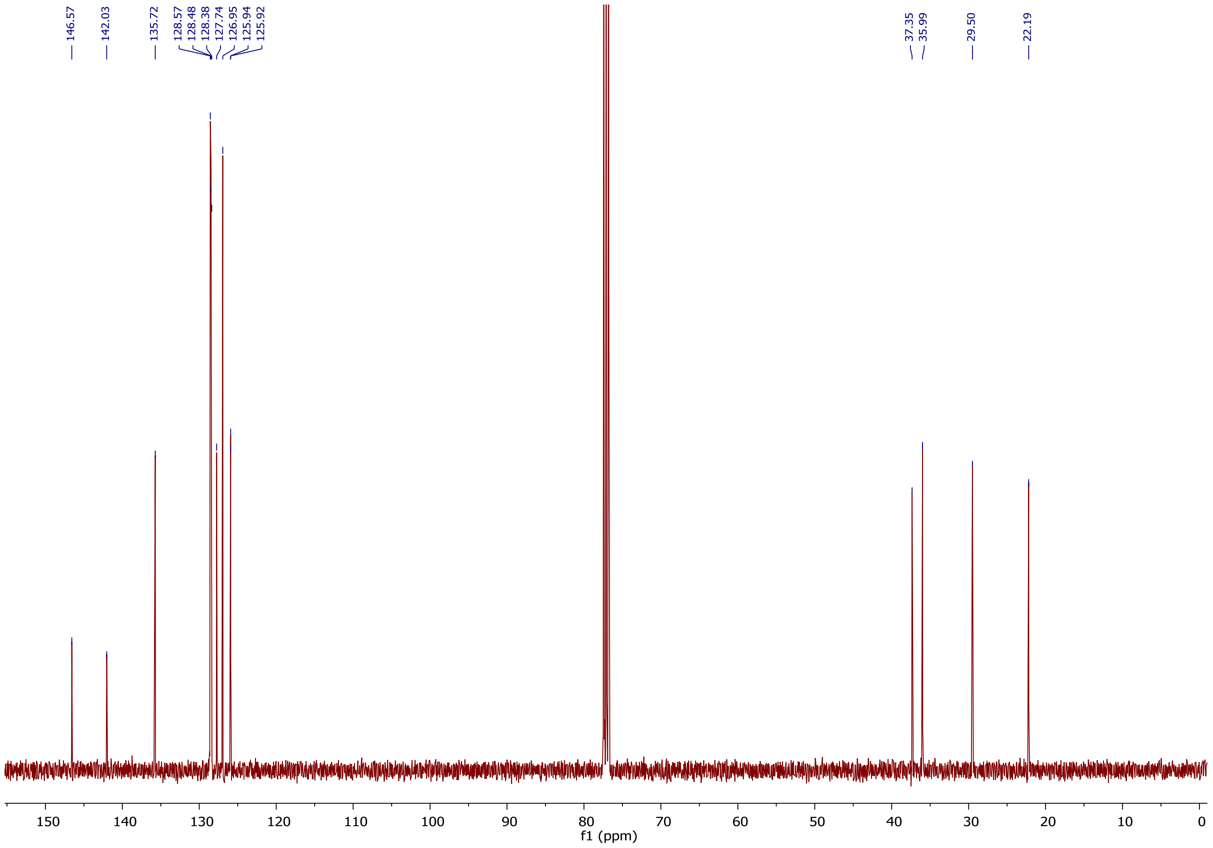
146.57
142.03
135.72
128.57
128.48
128.38
127.74
126.95
125.94
125.92
37.35
35.99
29.50
22.19
150 140 130 120 110 100 90 80 70 60 50 40 30 20 10 0
f1 (ppm)

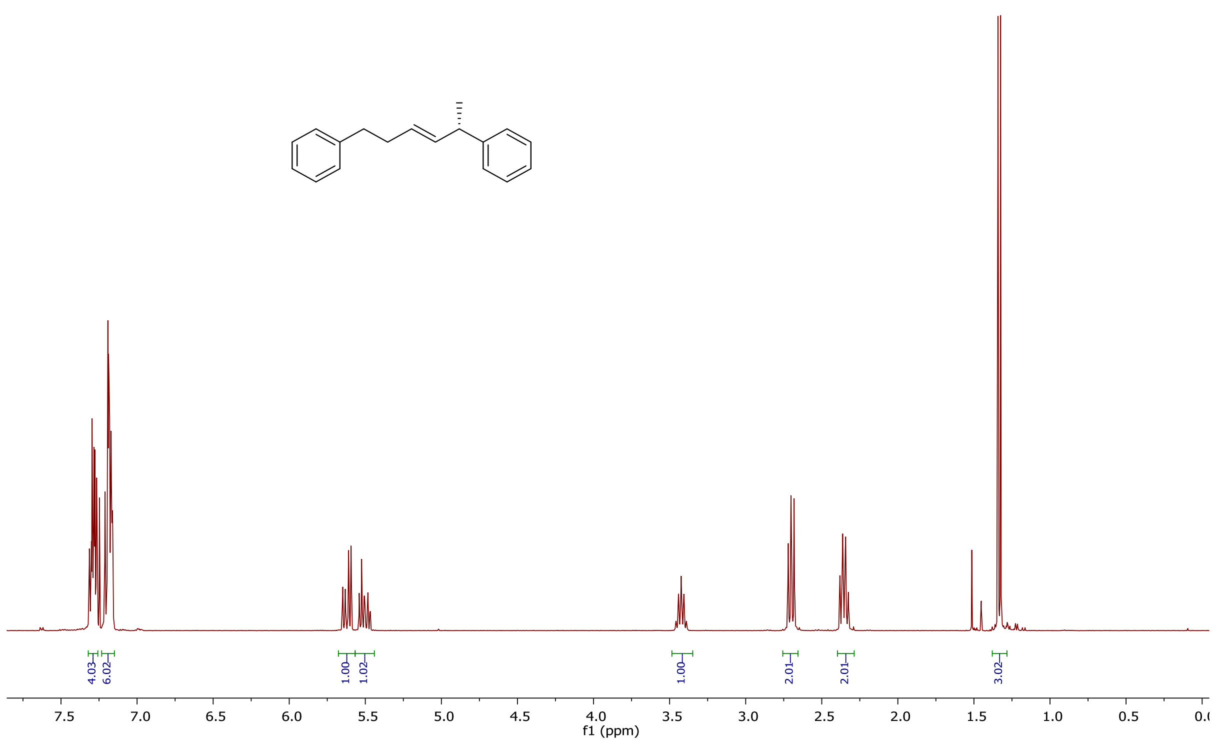
4.03
6.02
1.00
1.02
1.00
2.01
2.01
3.02
7.5
7.0
6.5
6.0
5.5
5.0
4.5
4.0
3.5
3.0
2.5
2.0
1.5
1.0
0.5
f1 (ppm)

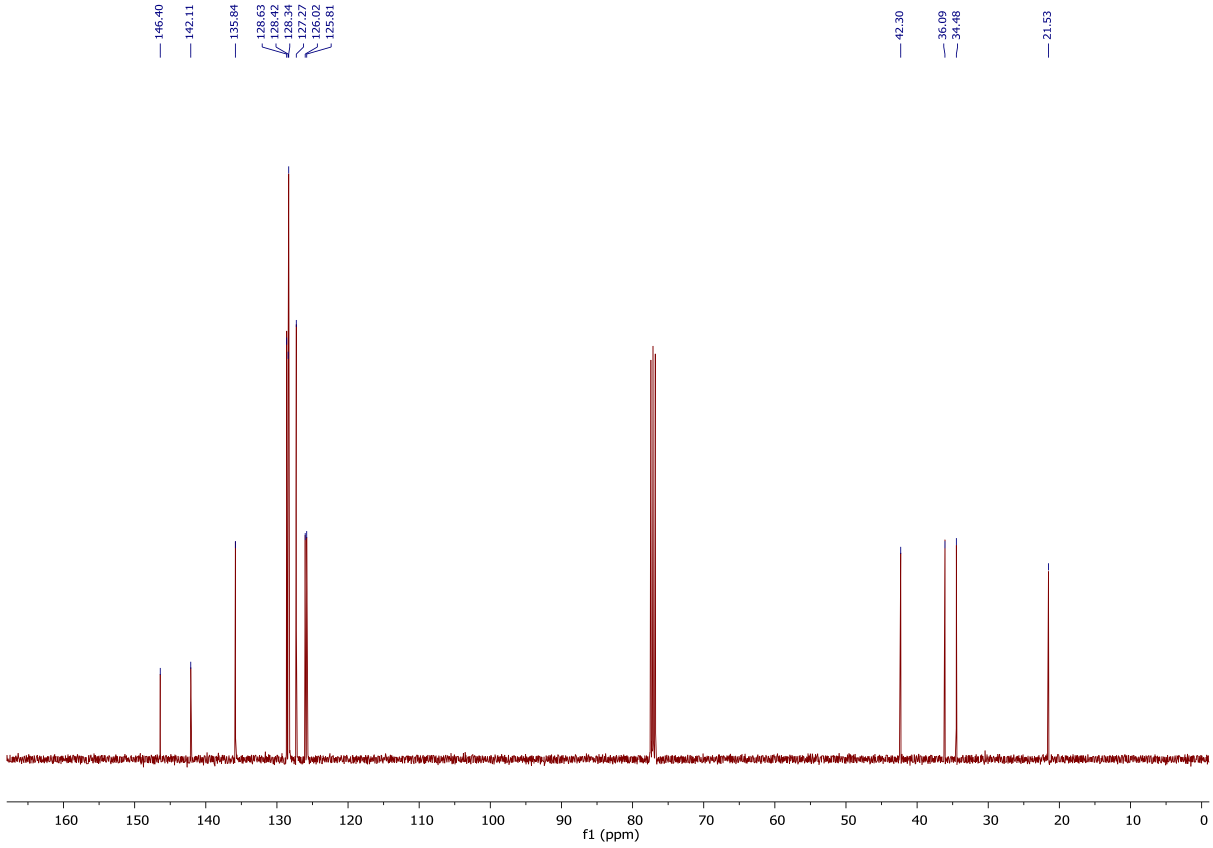
146.40
142.11
135.84
128.63
128.42
128.34
127.27
126.02
125.81
42.30
36.09
34.48
21.53
160
150
140
130
120
110
100
90
80
70
60
50
40
30
20
10
0
f1 (ppm)

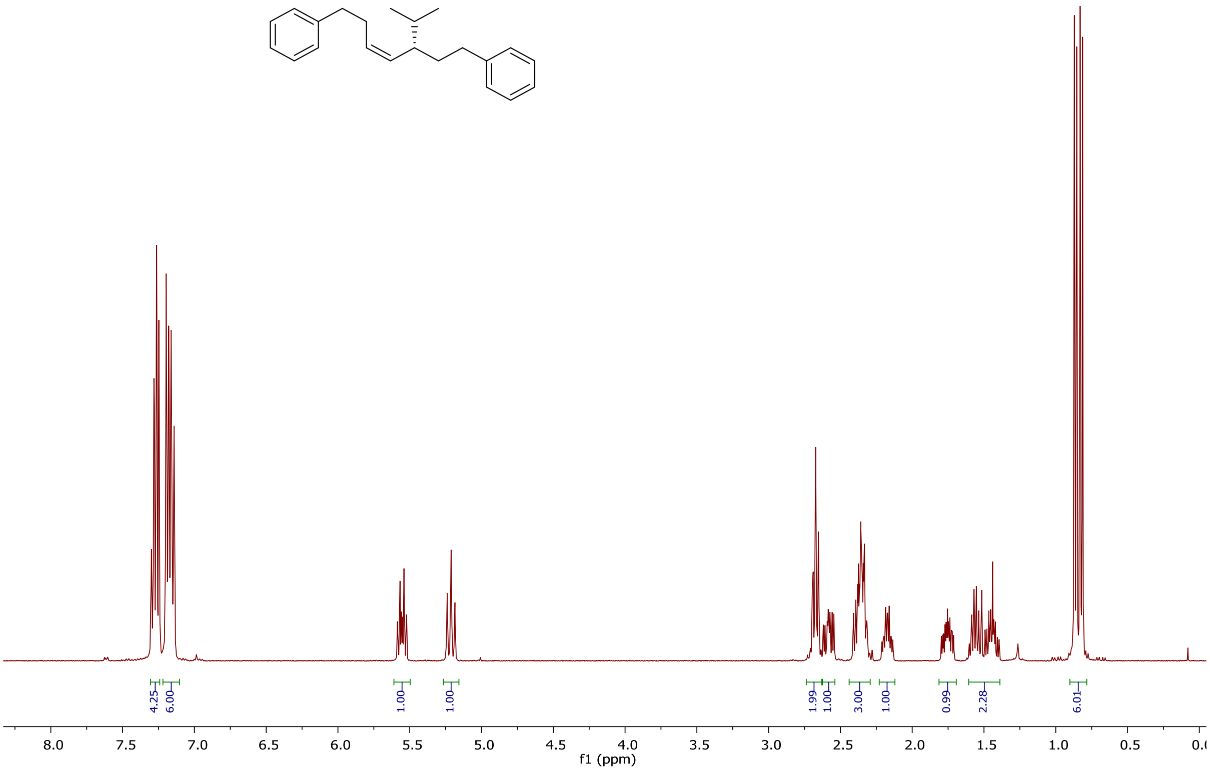
4.25
6.00
1.00
1.00
1.99
1.00
3.00
1.00
0.99
2.28
6.01
f1 (ppm)

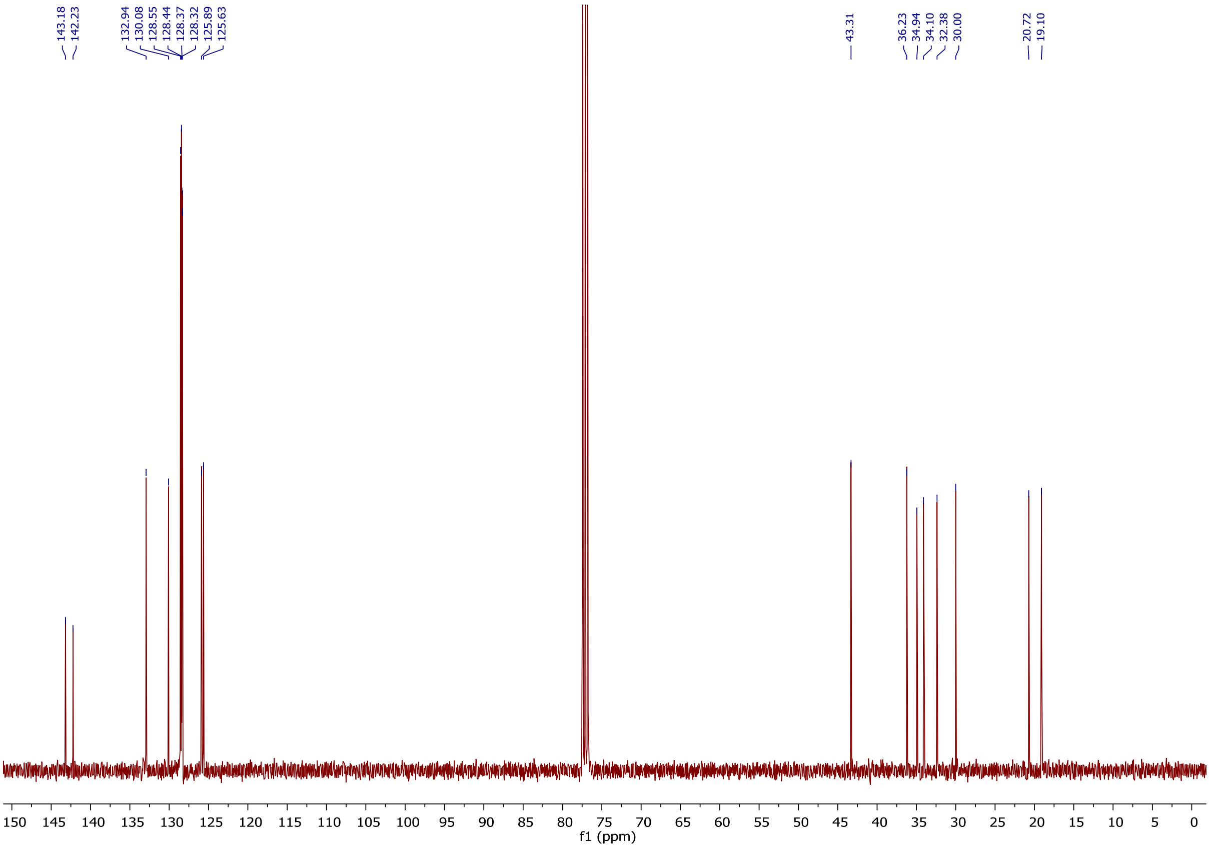
143.18
142.23
132.94
130.08
128.55
128.44
128.37
128.32
125.89
125.63
43.31
36.23
34.94
34.10
32.38
30.00
20.72
19.10
f1 (ppm)

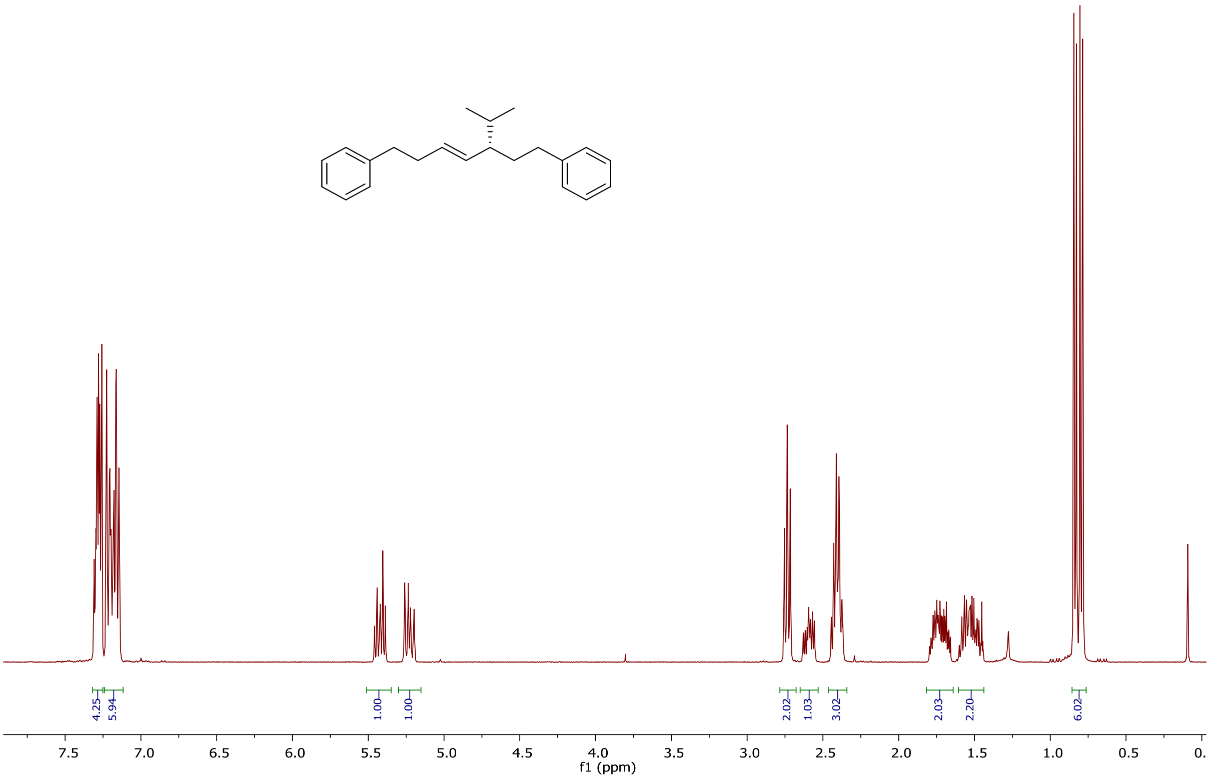
4.25
5.94
1.00
1.00
2.02
1.03
3.02
2.03
2.20
6.02
7.5 7.0 6.5 6.0 5.5 5.0 4.5 4.0 3.5 3.0 2.5 2.0 1.5 1.0 0.5 0.
f1 (ppm)

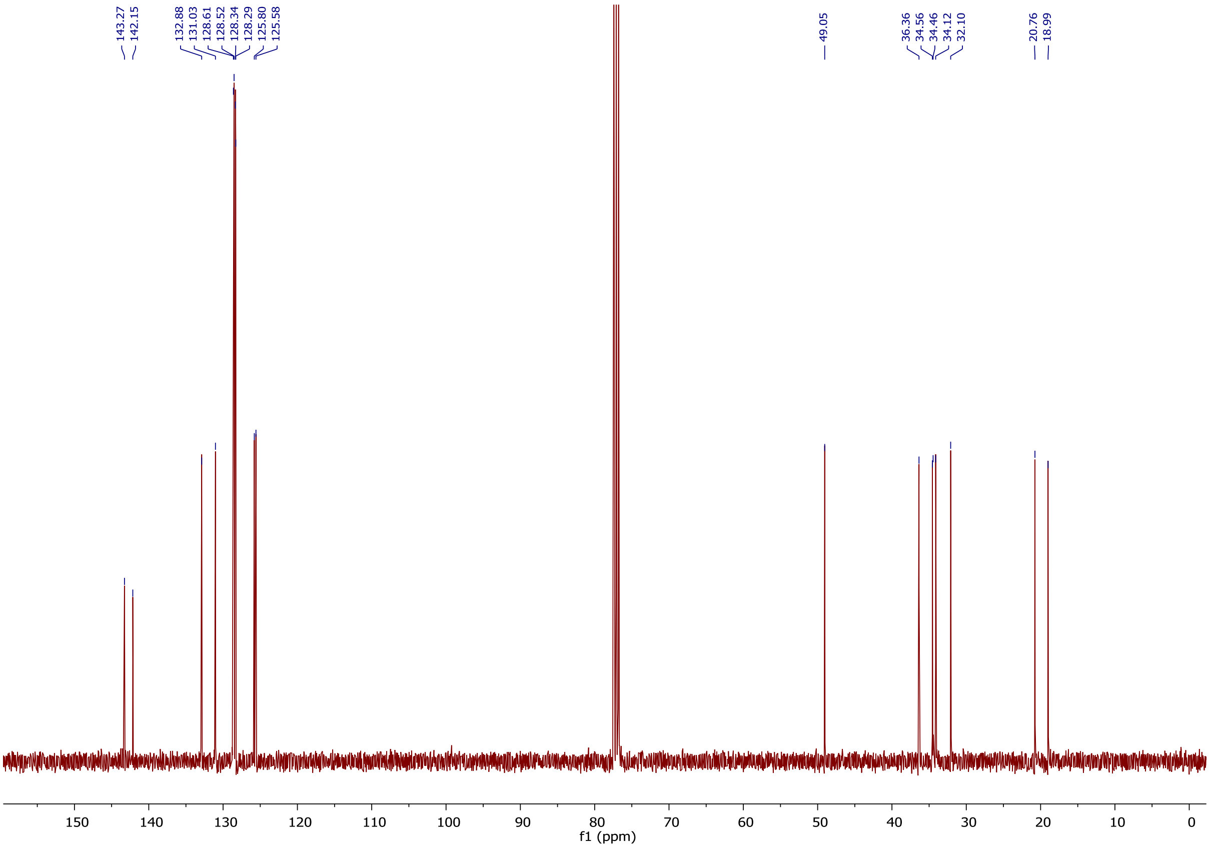
143.27
142.15
132.88
131.03
128.61
128.52
128.34
128.29
125.80
125.58
49.05
36.36
34.56
34.46
34.12
32.10
20.76
18.99
150 140 130 120 110 100 90 80 70 60 50 40 30 20 10 0
f1 (ppm)

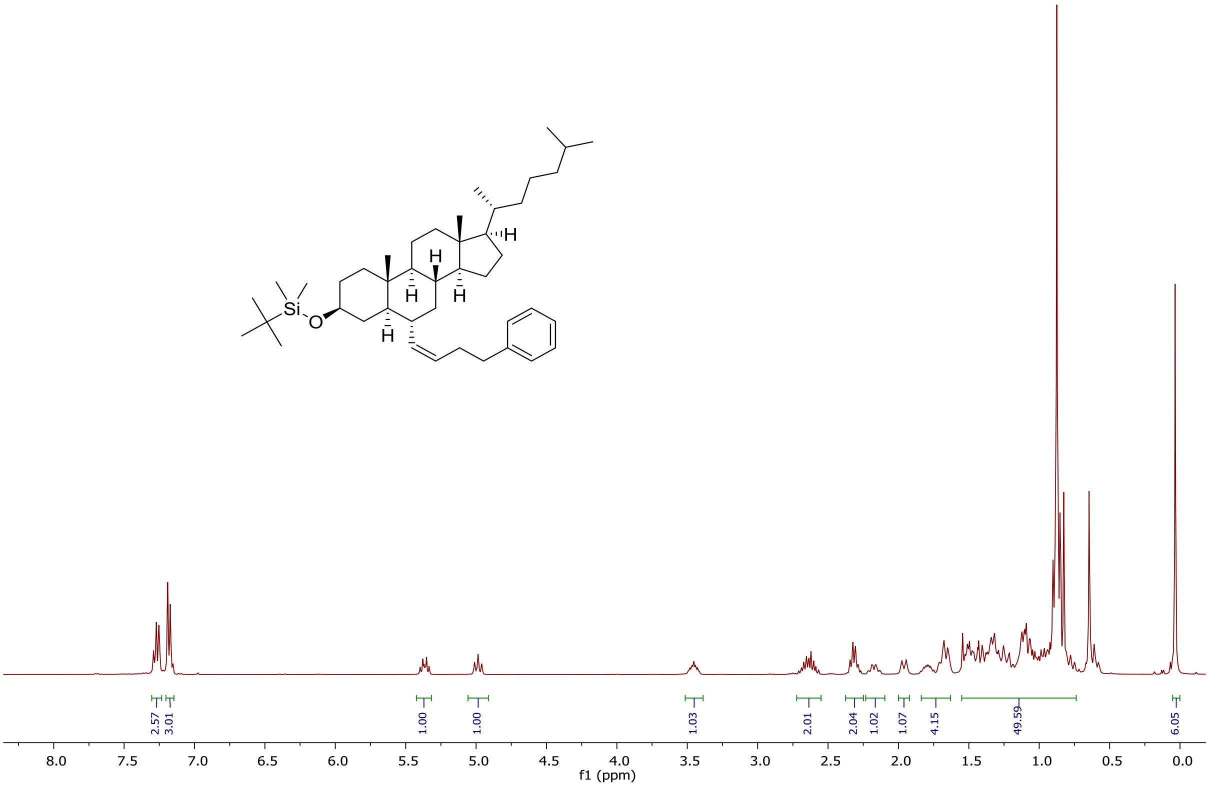

Si
O
H
2.57
3.01
1.00
1.00
1.03
2.01
2.04
1.02
1.07
4.15
49.59
6.05
8.0 7.5 7.0 6.5 6.0 5.5 5.0 4.5 4.0 3.5 3.0 2.5 2.0 1.5 1.0 0.5 0.0
f1 (ppm)

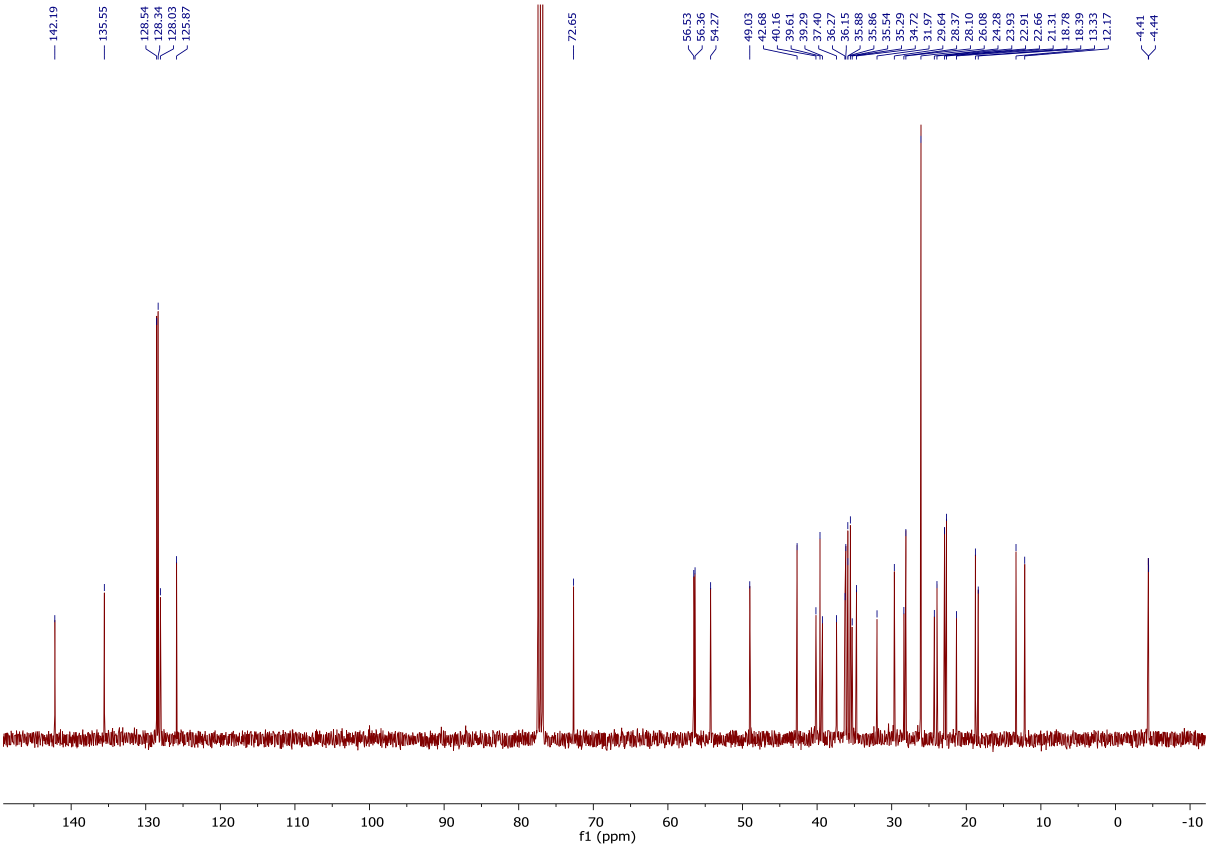

142.19
135.55
128.54
128.34
128.03
125.87
72.65
56.53
56.36
54.27
49.03
42.68
40.16
39.61
39.29
37.40
36.27
36.15
35.88
35.86
35.54
35.29
34.72
31.97
29.64
28.37
28.10
26.08
24.28
23.93
22.91
22.66
21.31
18.78
18.39
13.33
12.17
-4.41
-4.44
140 130 120 110 100 90 80 70 60 50 40 30 20 10 0 -10
f1 (ppm)

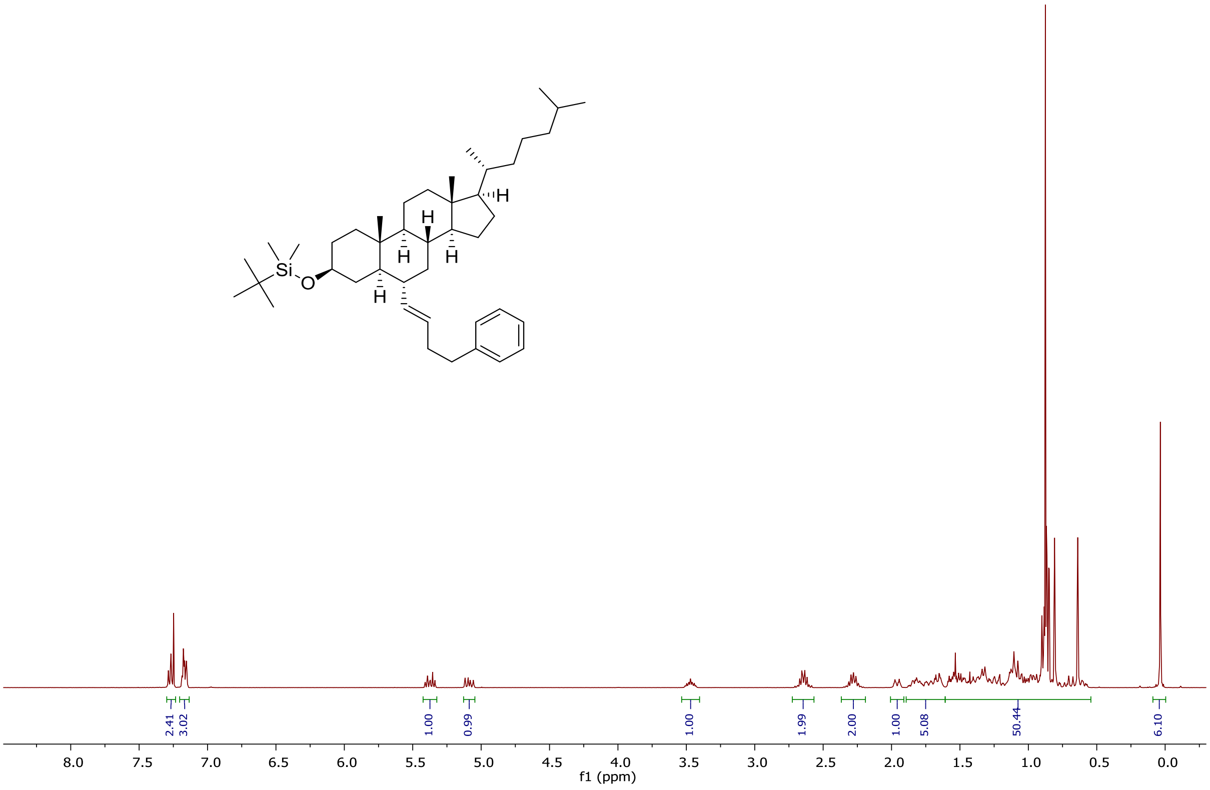

Si
O
H
H
H
H
H
2.41
3.02
1.00
0.99
1.00
1.99
2.00
1.00
5.08
50.44
6.10
8.0 7.5 7.0 6.5 6.0 5.5 5.0 4.5 4.0 3.5 3.0 2.5 2.0 1.5 1.0 0.5 0.0
f1 (ppm)

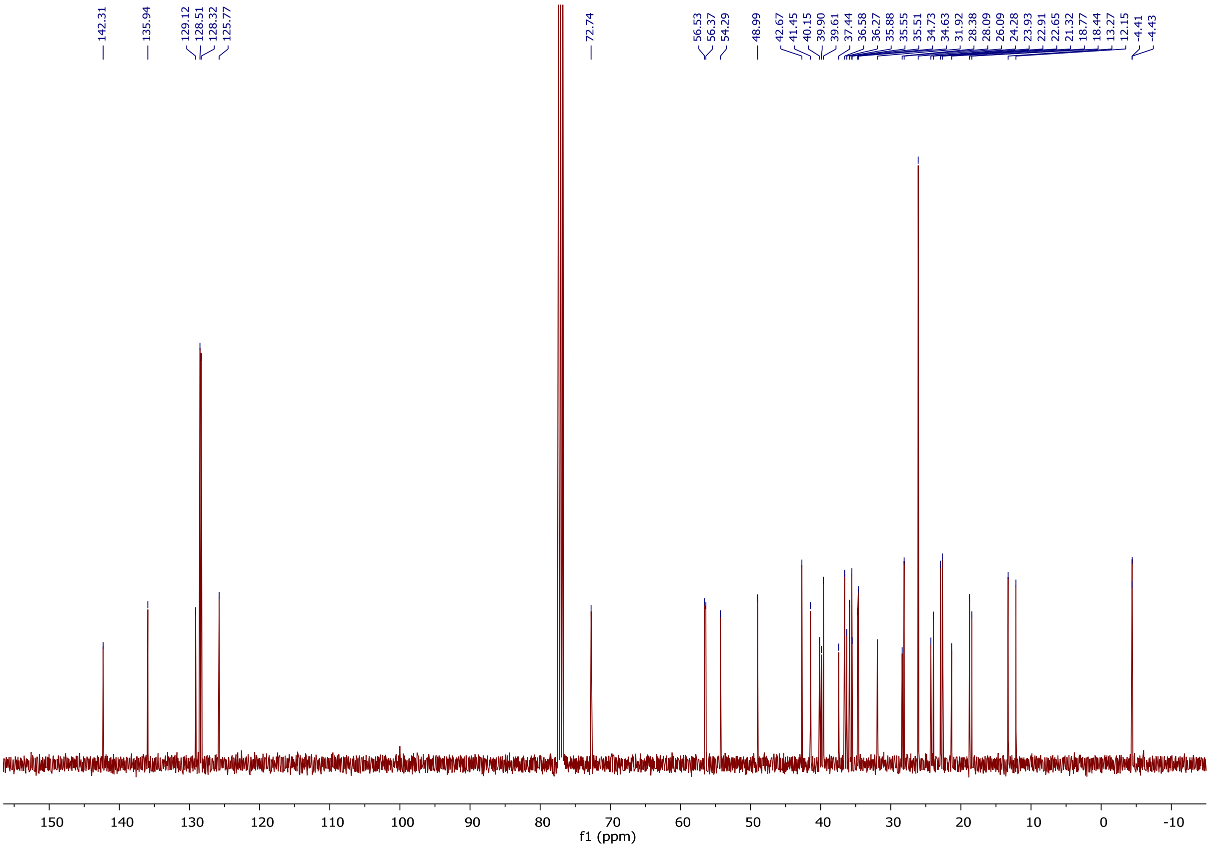

142.31
135.94
129.12
128.51
128.32
125.77
72.74
56.53
56.37
54.29
48.99
42.67
41.45
40.15
39.90
39.61
37.44
36.58
36.27
35.88
35.55
35.51
34.73
34.63
31.92
28.38
28.09
26.09
24.28
23.93
22.91
22.65
21.32
18.77
18.44
13.27
12.15
-4.41
-4.43
150 140 130 120 110 100 90 80 70 60 50 40 30 20 10 0 -10
f1 (ppm)

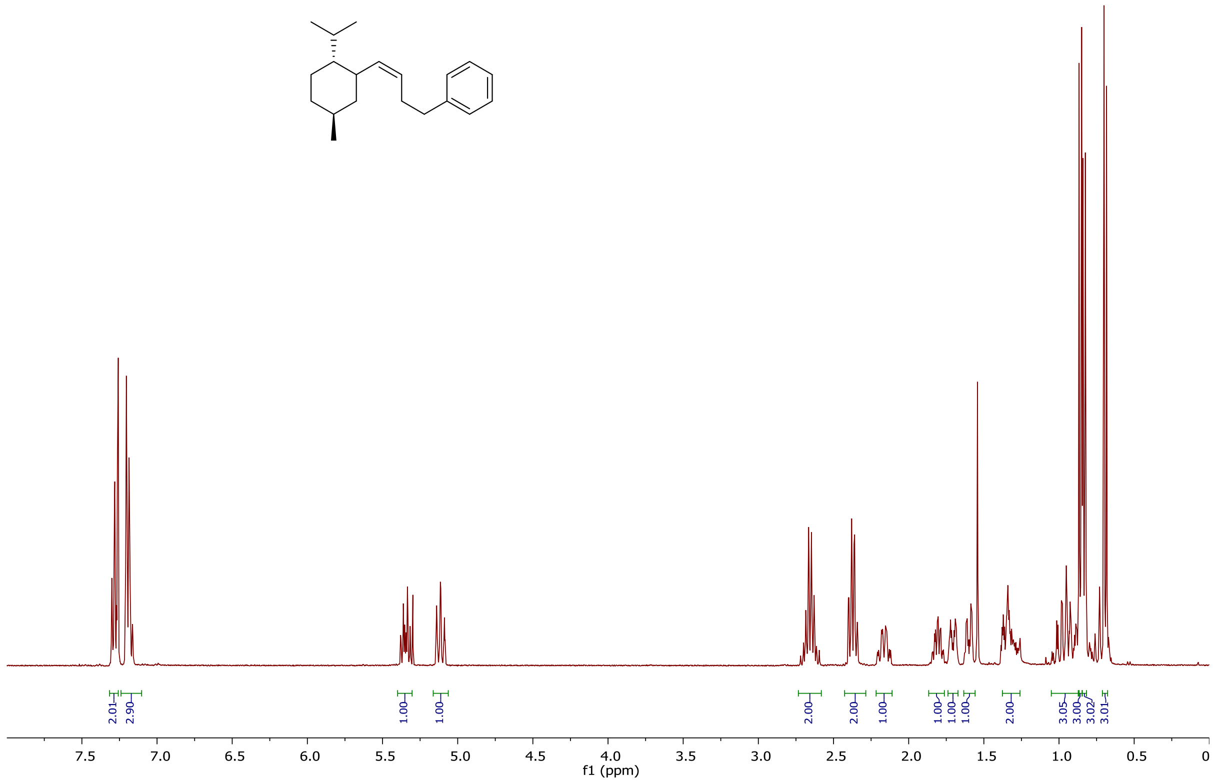

2.01
2.90
1.00
1.00
2.00
2.00
1.00
1.00
1.00
1.00
2.00
3.05
3.00
3.02
3.01
7.5
7.0
6.5
6.0
5.5
5.0
4.5
4.0
3.5
3.0
2.5
2.0
1.5
1.0
0.5
0
f1 (ppm)

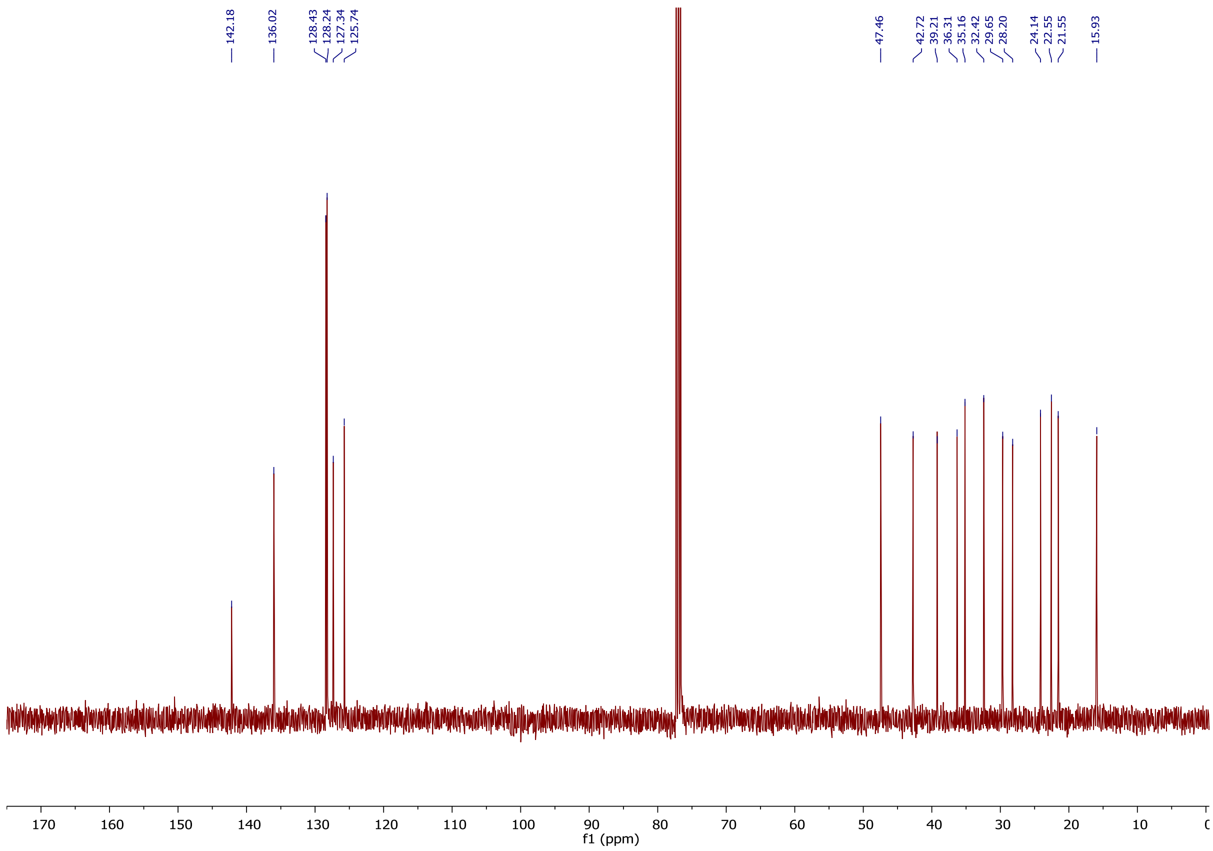

142.18
136.02
128.43
128.24
127.34
125.74
47.46
42.72
39.21
36.31
35.16
32.42
29.65
28.20
24.14
22.55
21.55
15.93
170
160
150
140
130
120
110
100
90
80
70
60
50
40
30
20
10
f1 (ppm)

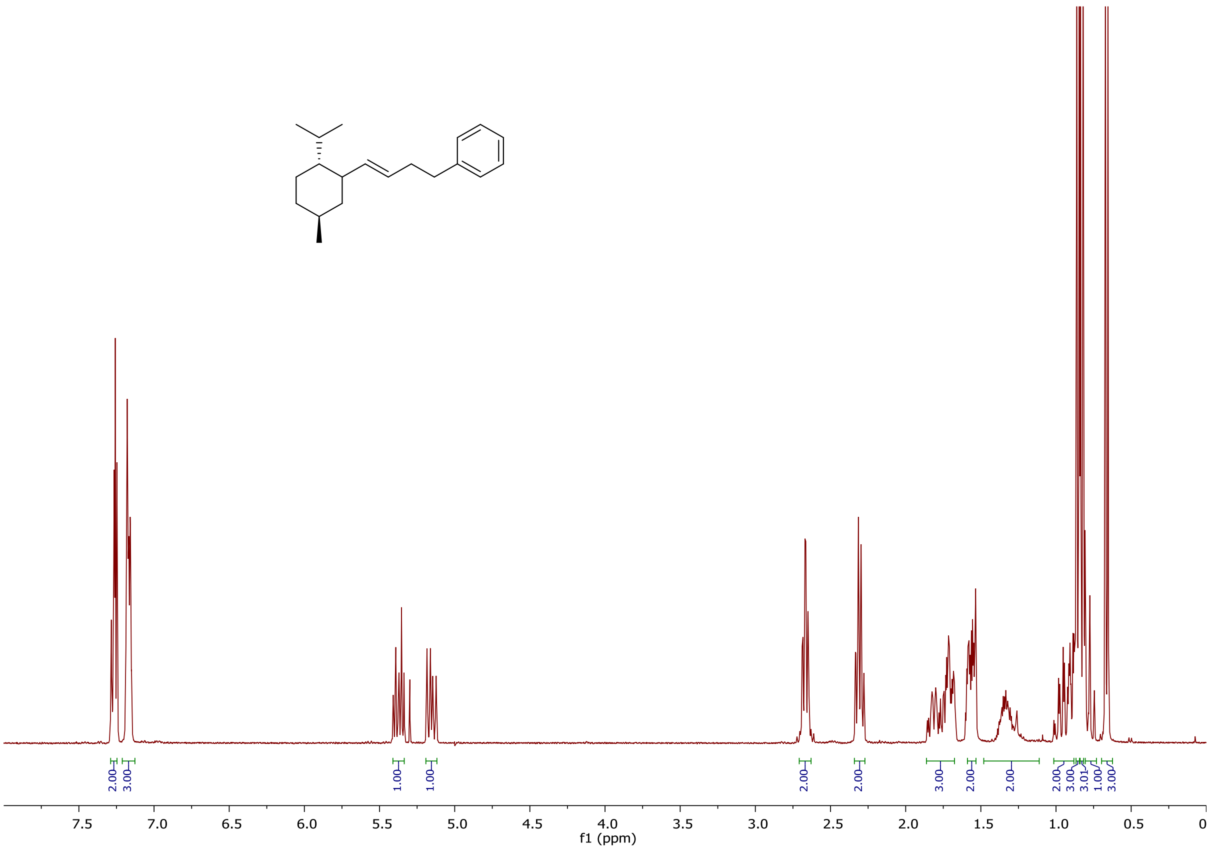
2.00
3.00
1.00
1.00
2.00
2.00
3.00
2.00
2.00
2.00
3.00
3.01
1.00
3.00
7.5
7.0
6.5
6.0
5.5
5.0
4.5
4.0
3.5
3.0
2.5
2.0
1.5
1.0
0.5
0
f1 (ppm)

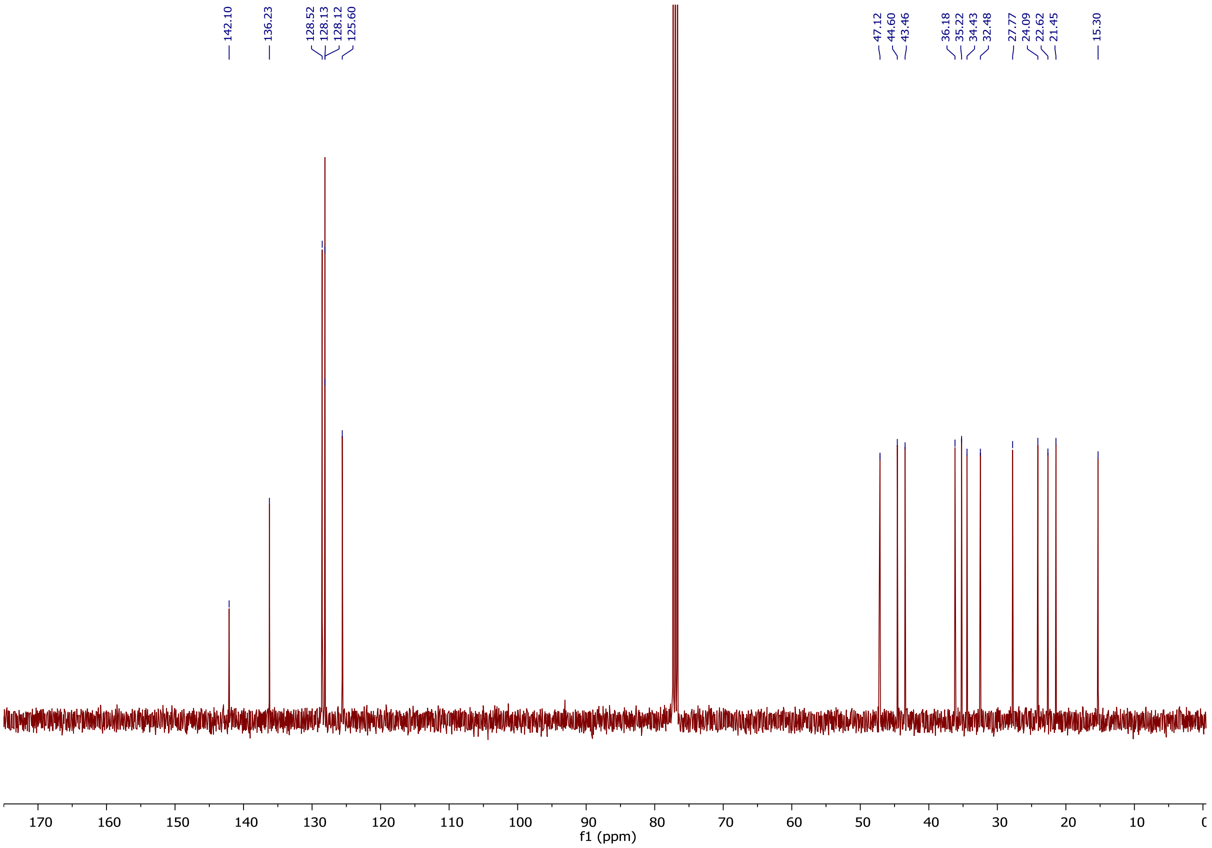
142.10
136.23
128.52
128.13
128.12
125.60
47.12
44.60
43.46
36.18
35.22
34.43
32.48
27.77
24.09
22.62
21.45
15.30
170
160
150
140
130
120
110
100
90
80
70
60
50
40
30
20
10
f1 (ppm)

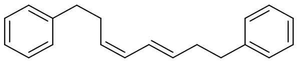

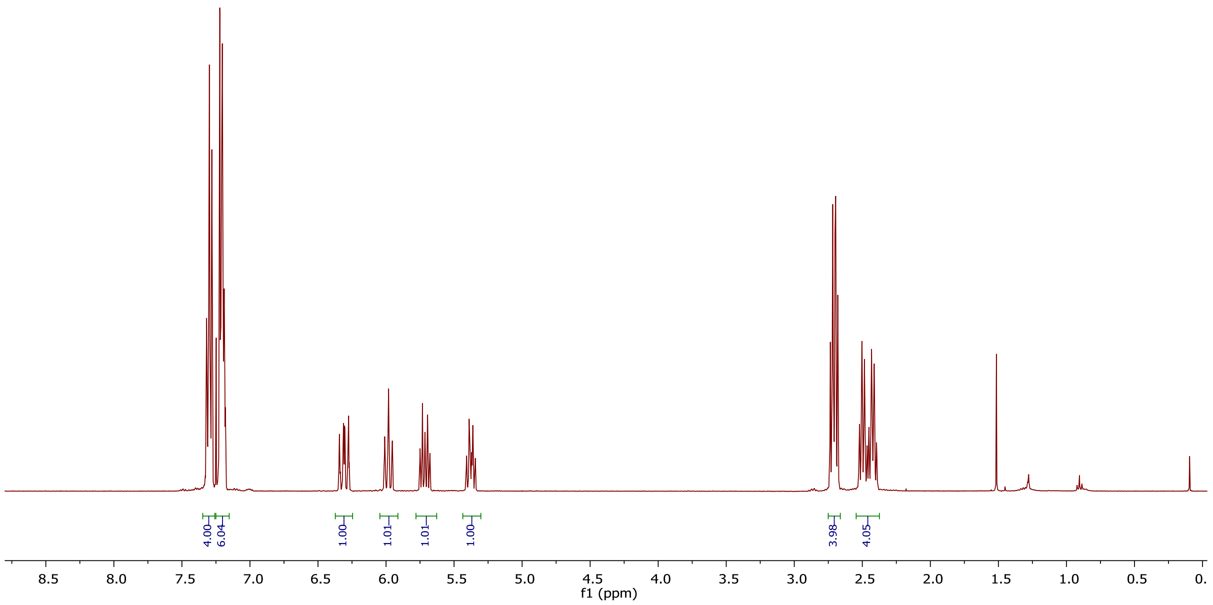
4.00
6.04
1.00
1.01
1.01
1.00
3.98
4.05
8.5 8.0 7.5 7.0 6.5 6.0 5.5 5.0 4.5 4.0 3.5 3.0 2.5 2.0 1.5 1.0 0.5 0.
f1 (ppm)

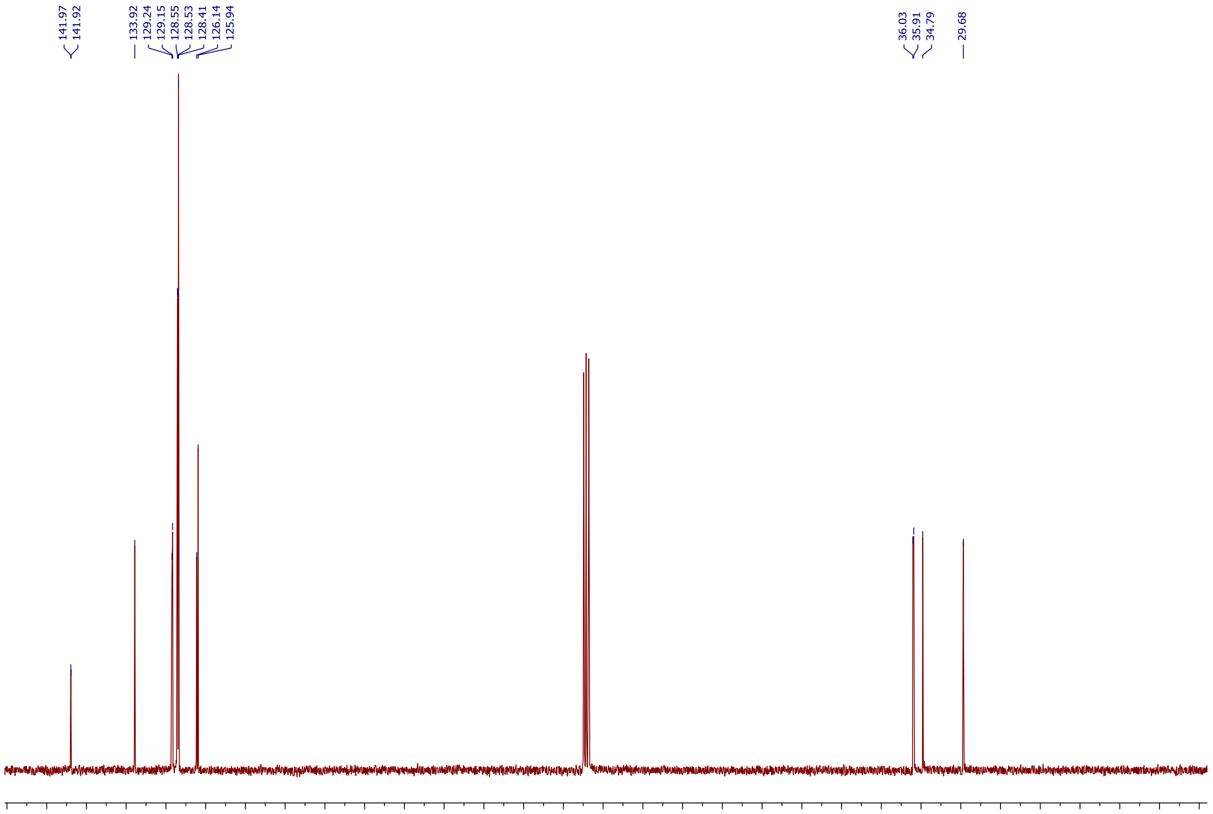
141.97
141.92
133.92
129.24
129.15
128.55
128.53
128.41
126.14
125.94
36.03
35.91
34.79
29.68
50 145 140 135 130 125 120 115 110 105 100 95 90 85 80 75 70 65 60 55 50 45 40 35 30 25 20 15 10 5 0
f1 (ppm)

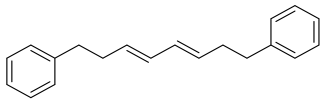

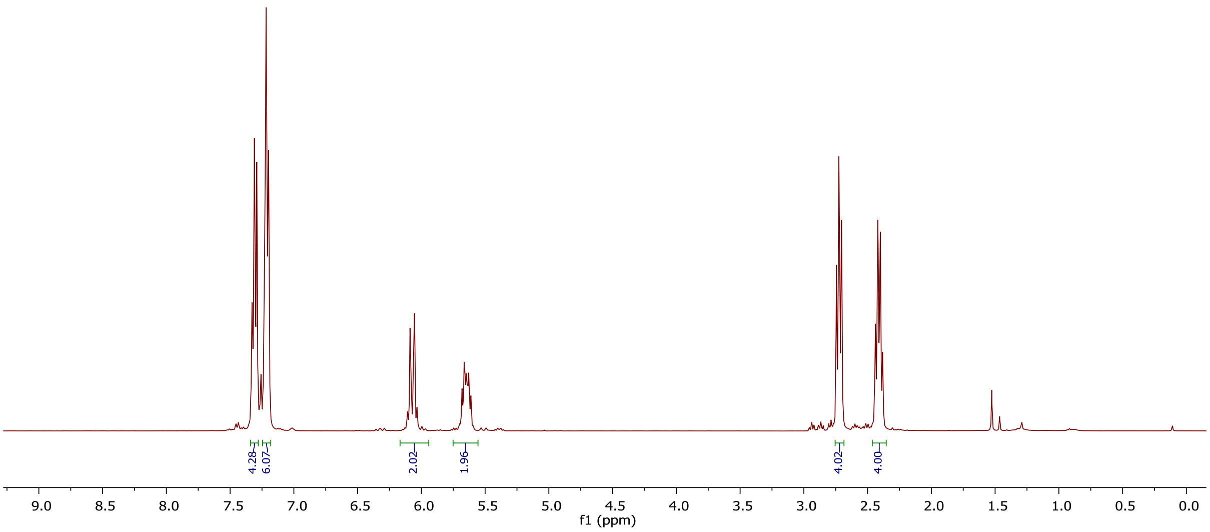
4.28
6.07
2.02
1.96
4.02
4.00
9.0 8.5 8.0 7.5 7.0 6.5 6.0 5.5 5.0 4.5 4.0 3.5 3.0 2.5 2.0 1.5 1.0 0.5 0.0
f1 (ppm)

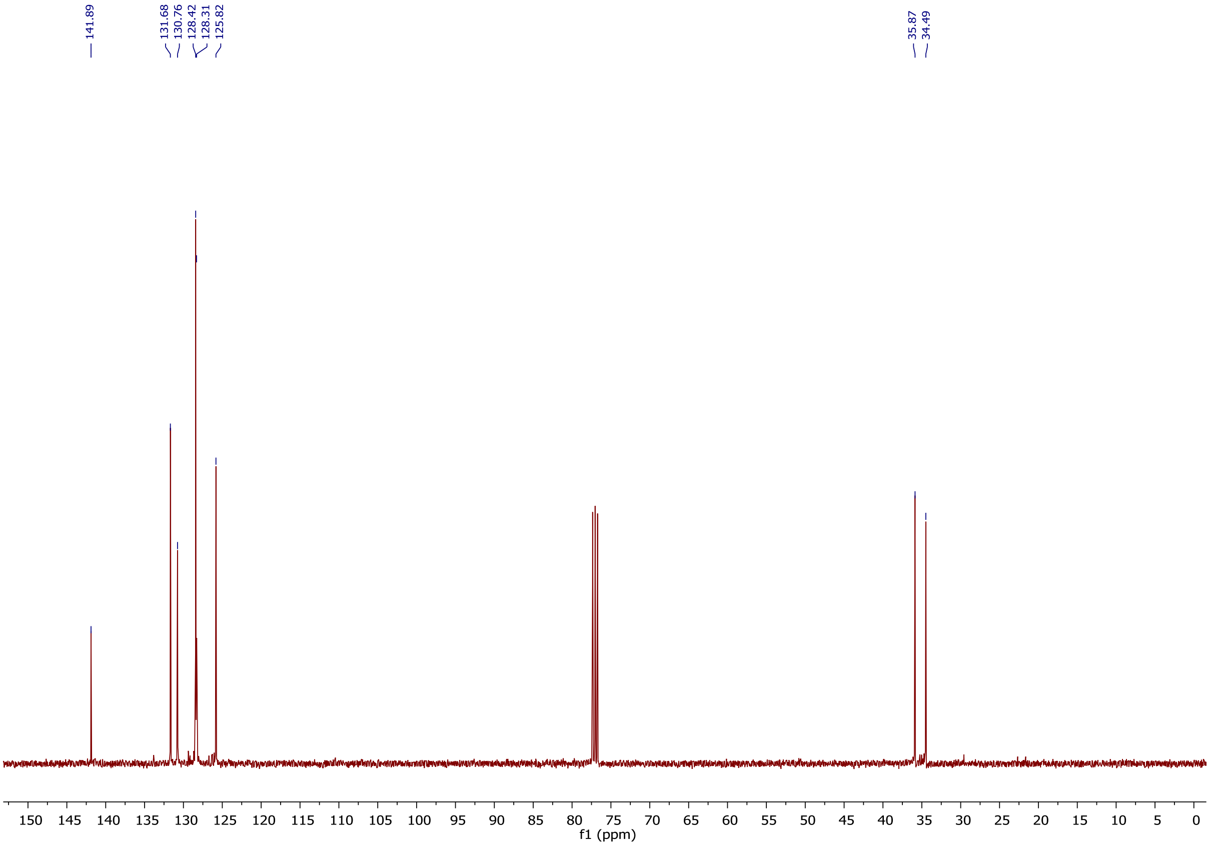
141.89
131.68
130.76
128.42
128.31
125.82
35.87
34.49
150 145 140 135 130 125 120 115 110 105 100 95 90 85 80 75 70 65 60 55 50 45 40 35 30 25 20 15 10 5 0
f1 (ppm)

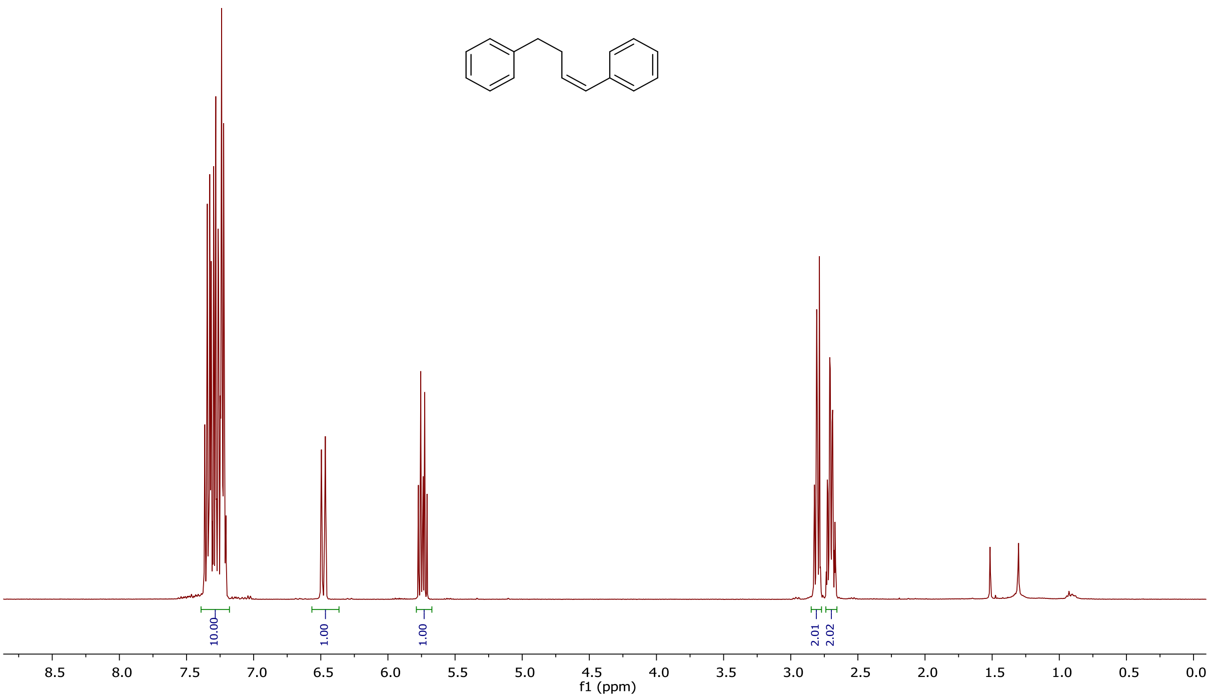
10.00
1.00
1.00
2.01
2.02
8.5 8.0 7.5 7.0 6.5 6.0 5.5 5.0 4.5 4.0 3.5 3.0 2.5 2.0 1.5 1.0 0.5 0.0
f1 (ppm)

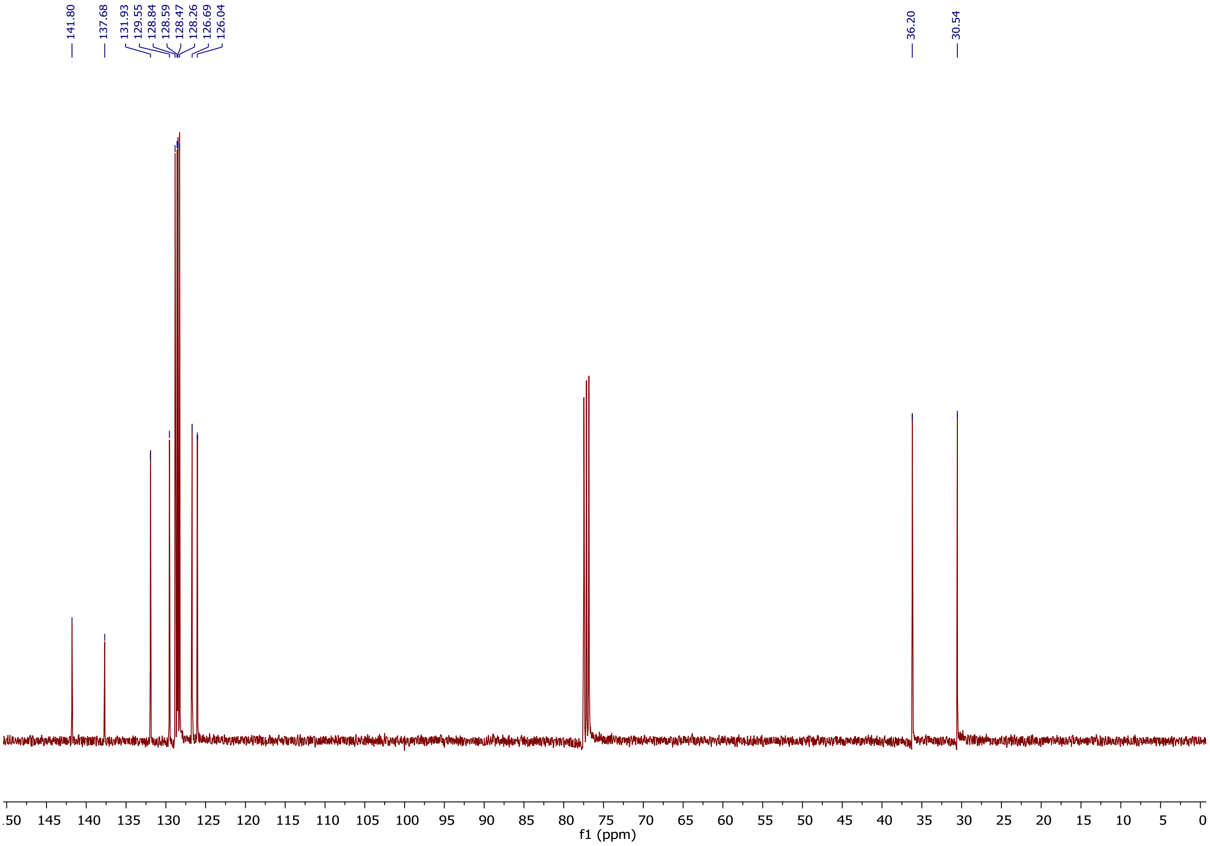
141.80
137.68
131.93
129.55
128.84
128.59
128.47
128.26
126.69
126.04
36.20
30.54
150 145 140 135 130 125 120 115 110 105 100 95 90 85 80 75 70 65 60 55 50 45 40 35 30 25 20 15 10 5 0
f1 (ppm)

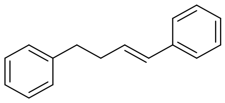

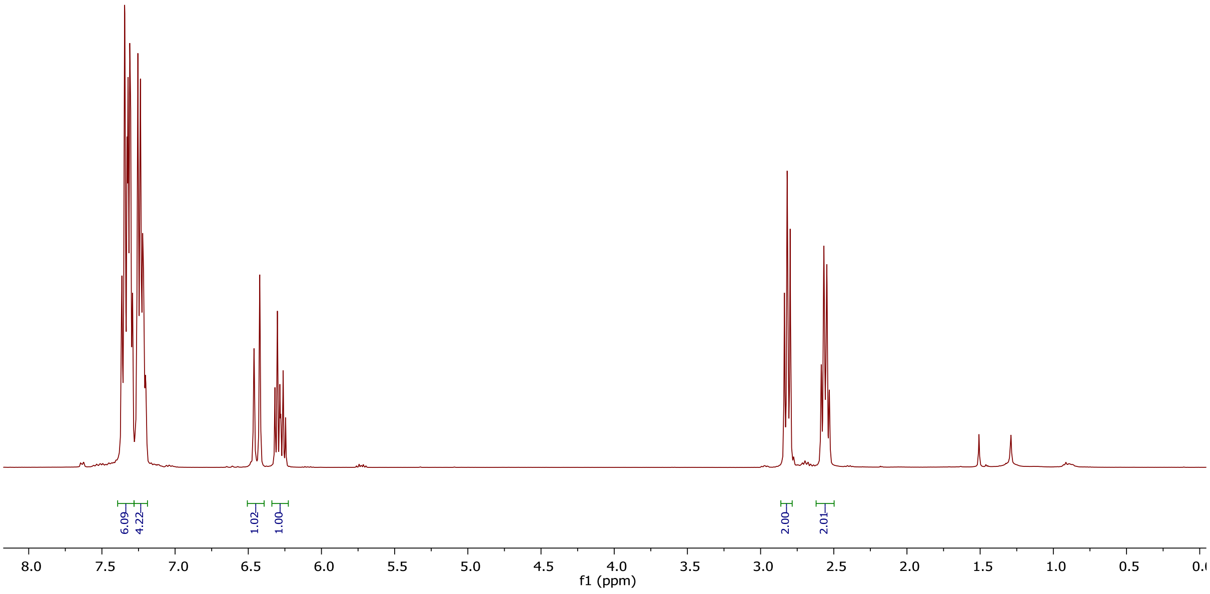
6.09
4.22
1.02
1.00
2.00
2.01
8.0 7.5 7.0 6.5 6.0 5.5 5.0 4.5 4.0 3.5 3.0 2.5 2.0 1.5 1.0 0.5
f1 (ppm)

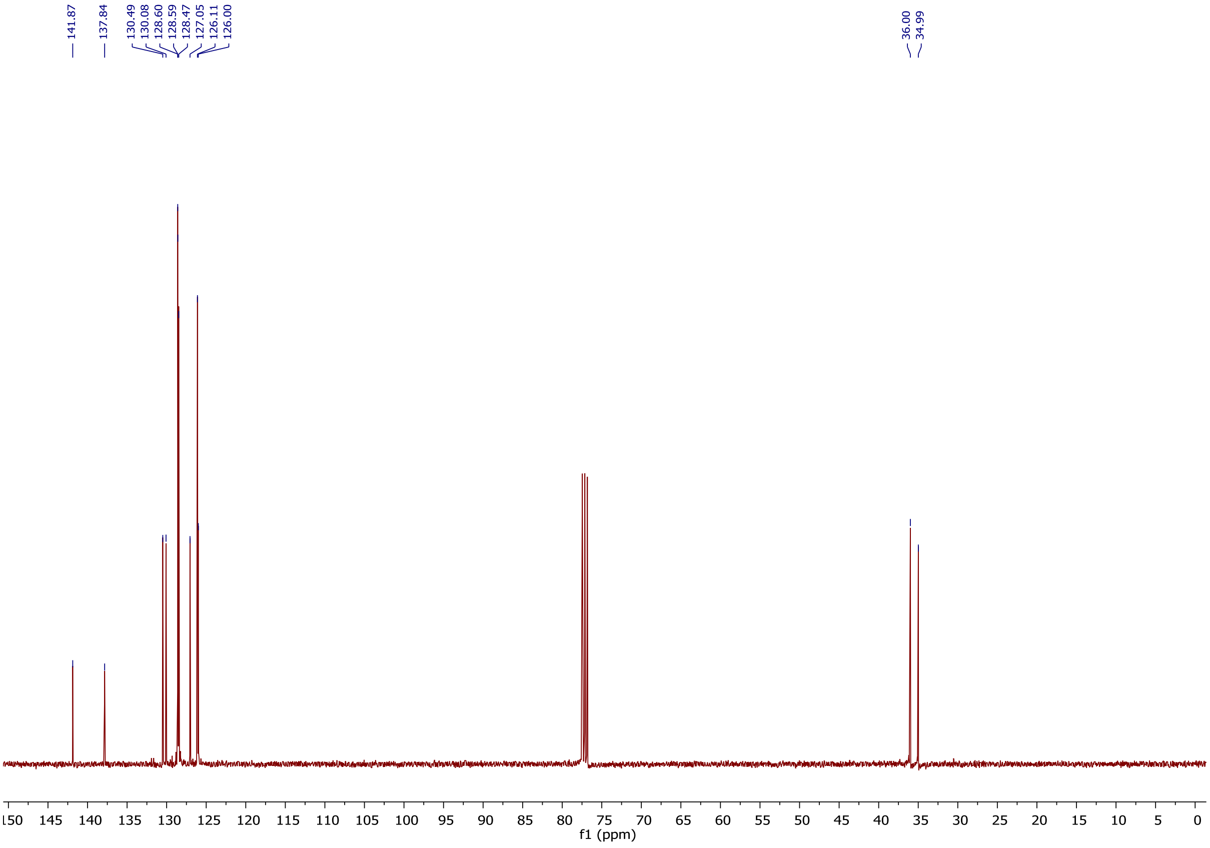
141.87
137.84
130.49
130.08
128.60
128.59
128.47
127.05
126.11
126.00
36.00
34.99
145 140 135 130 125 120 115 110 105 100 95 90 85 80 75 70 65 60 55 50 45 40 35 30 25 20 15 10 5 0
f1 (ppm)

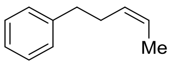
Me

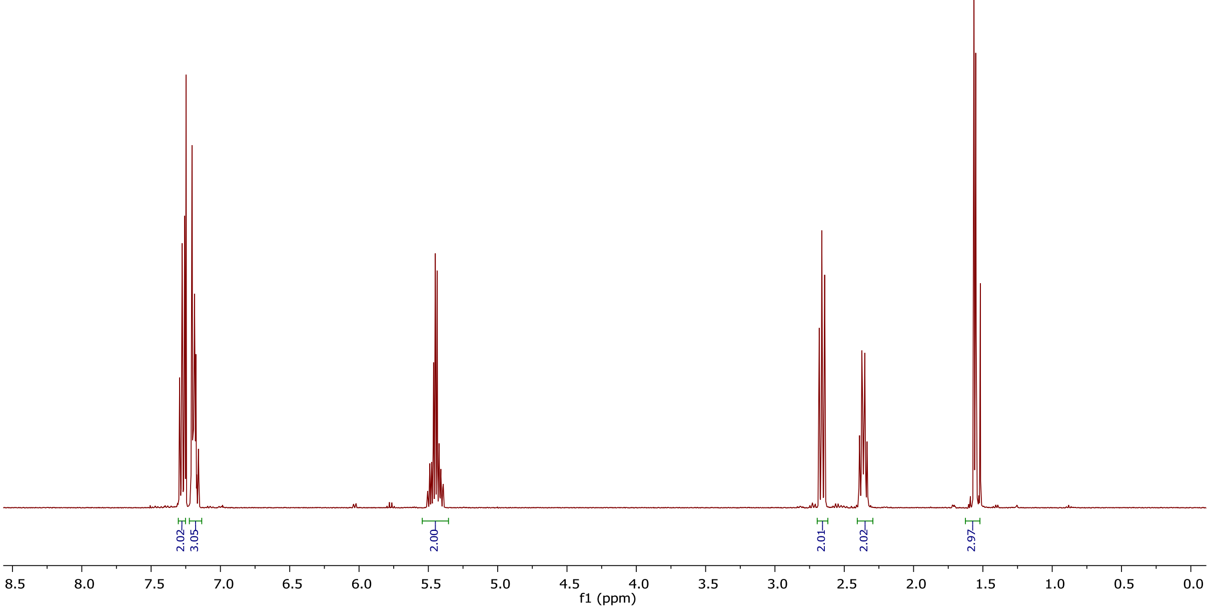
2.02
3.05
2.00
2.01
2.02
2.97
8.5
8.0
7.5
7.0
6.5
6.0
5.5
5.0
4.5
4.0
3.5
3.0
2.5
2.0
1.5
1.0
0.5
0.0
f1 (ppm)

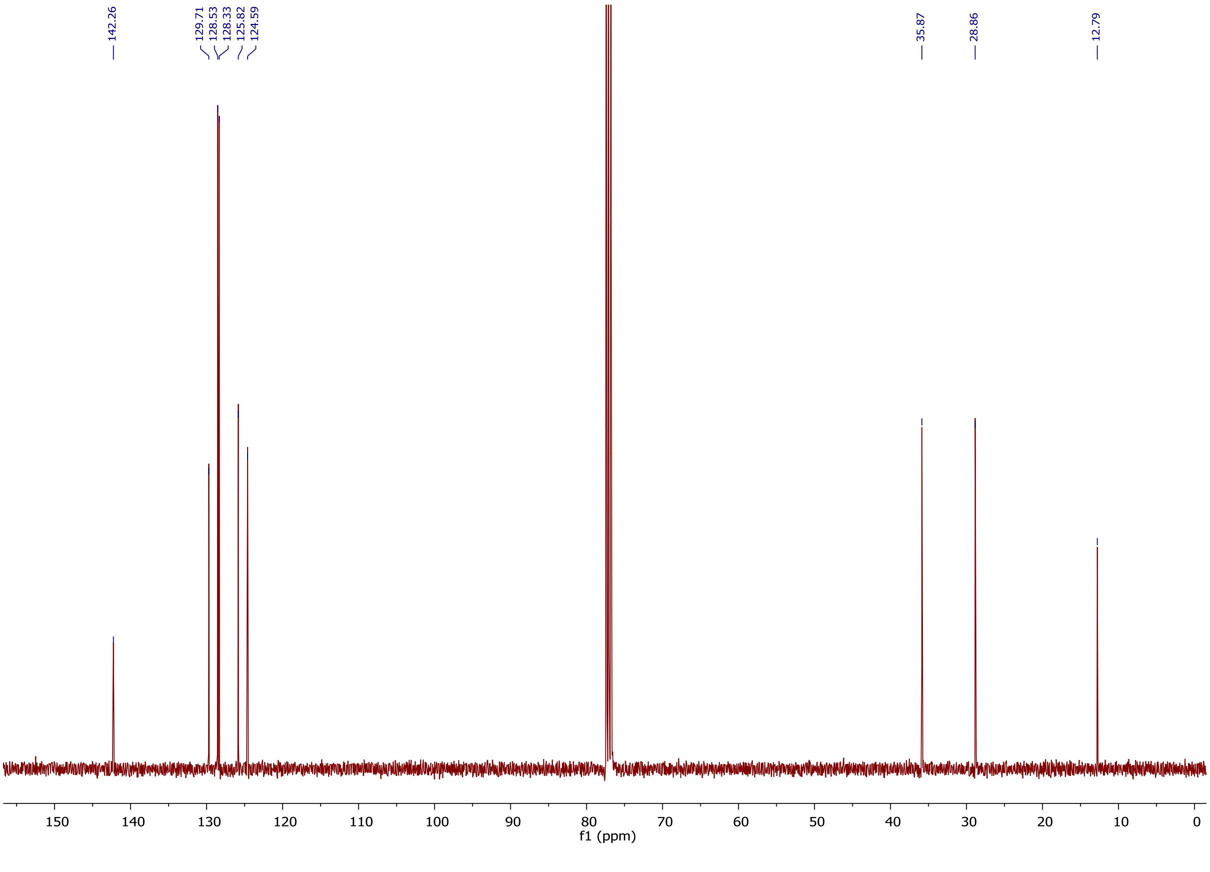
142.26
129.71
128.53
128.33
125.82
124.59
35.87
28.86
12.79
150
140
130
120
110
100
90
80
70
60
50
40
30
20
10
0
f1 (ppm)

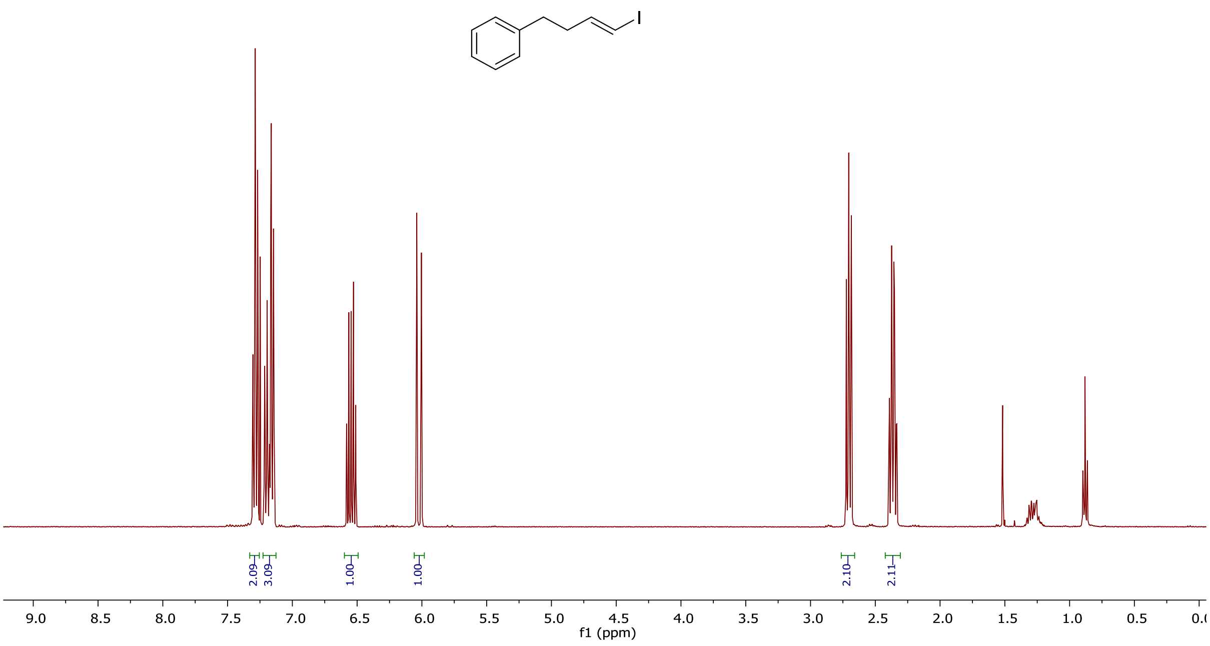
I
2.09
3.09
1.00
1.00
2.10
2.11
9.0 8.5 8.0 7.5 7.0 6.5 6.0 5.5 5.0 4.5 4.0 3.5 3.0 2.5 2.0 1.5 1.0 0.5
f1 (ppm)

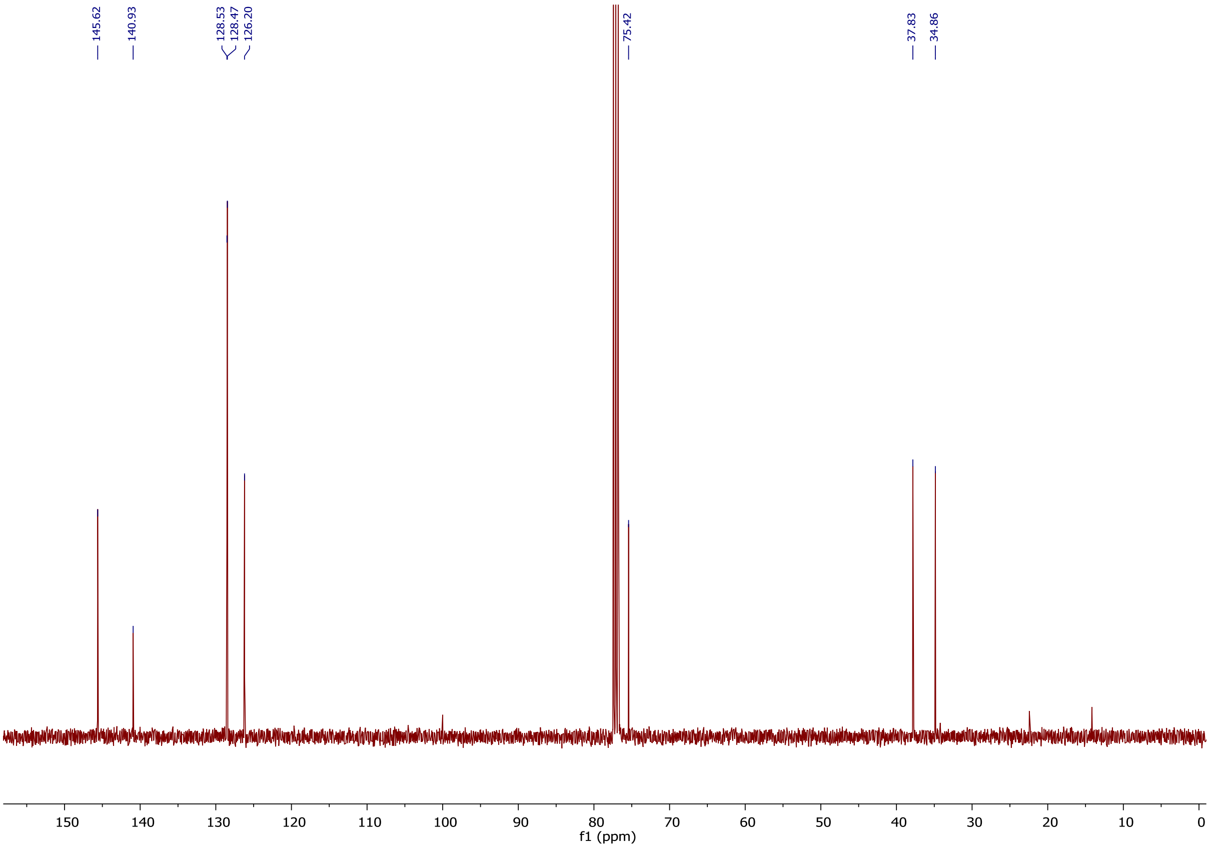
145.62
140.93
128.53
128.47
126.20
75.42
37.83
34.86
150 140 130 120 110 100 90 80 70 60 50 40 30 20 10 0
f1 (ppm)

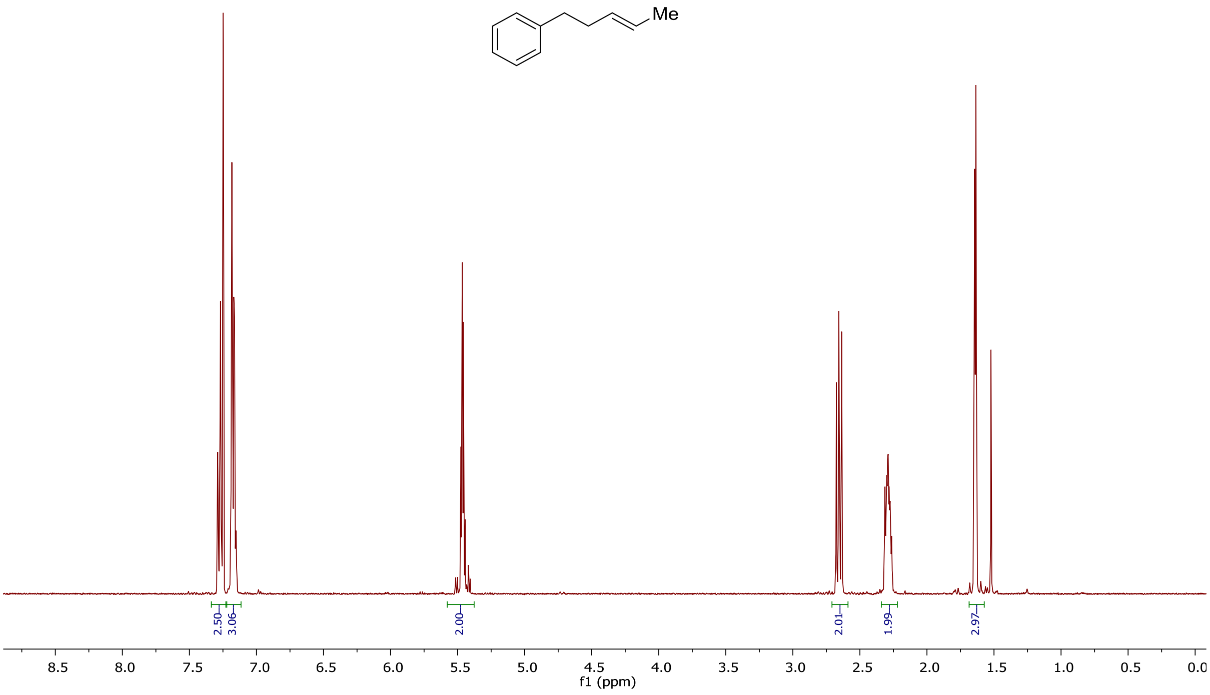
Me
2.50
3.06
2.00
2.01
1.99
2.97
8.5 8.0 7.5 7.0 6.5 6.0 5.5 5.0 4.5 4.0 3.5 3.0 2.5 2.0 1.5 1.0 0.5 0.0
f1 (ppm)

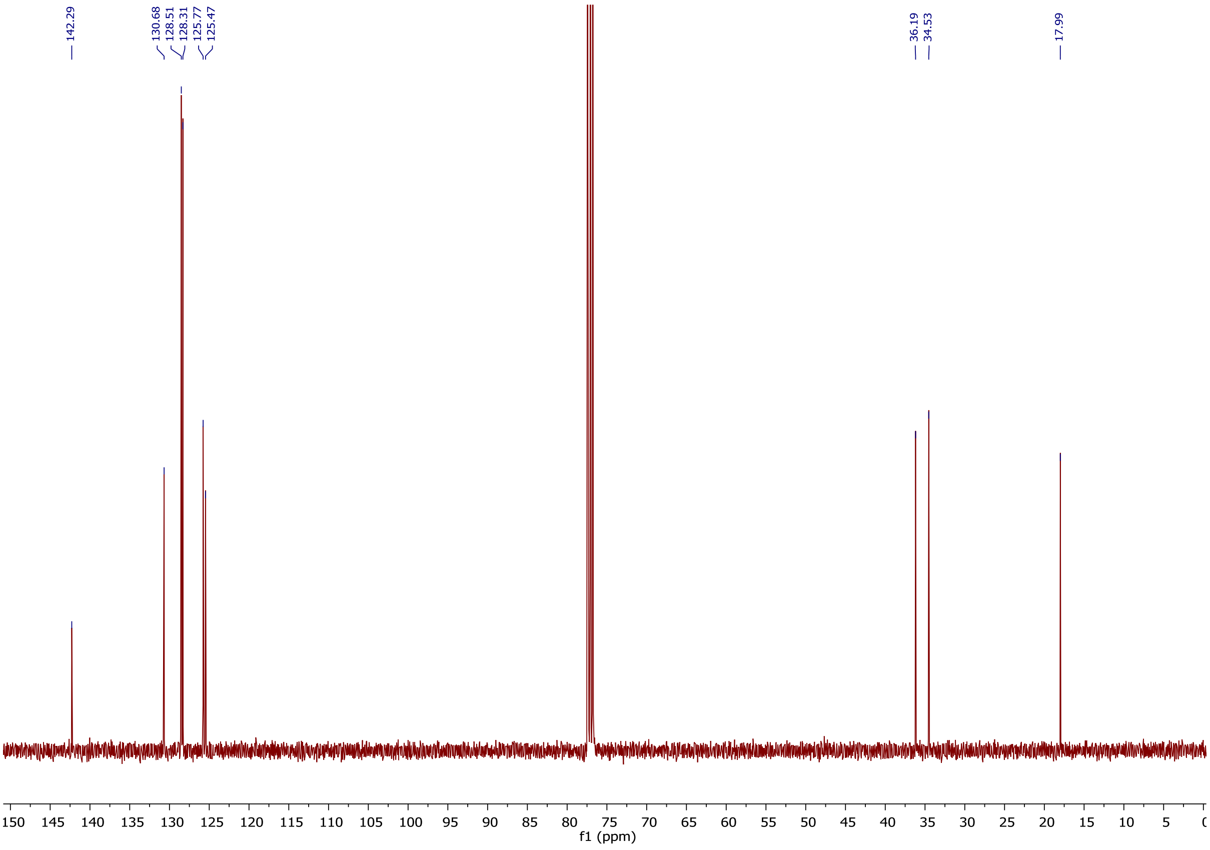
142.29
130.68
128.51
128.31
125.77
125.47
36.19
34.53
17.99
150 145 140 135 130 125 120 115 110 105 100 95 90 85 80 75 70 65 60 55 50 45 40 35 30 25 20 15 10 5
f1 (ppm)

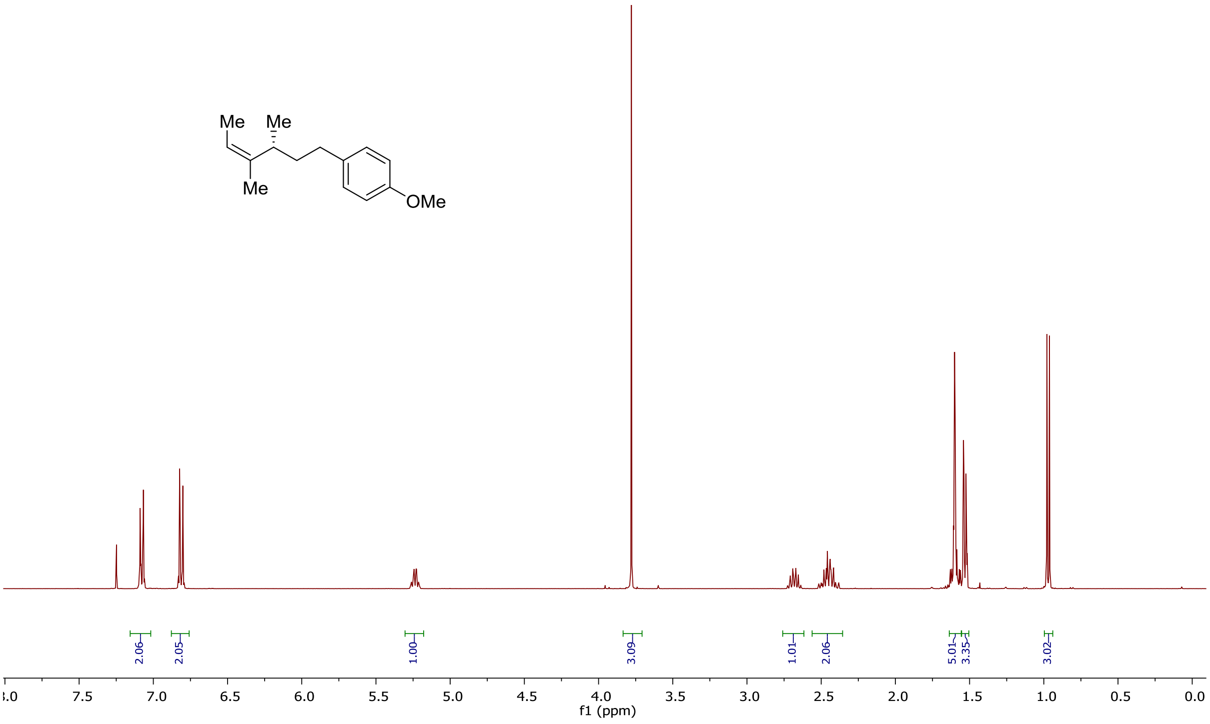
Me
Me
Me
OMe
2.06
2.05
1.00
3.09
1.01
2.06
5.01
3.35
3.02
f1 (ppm)

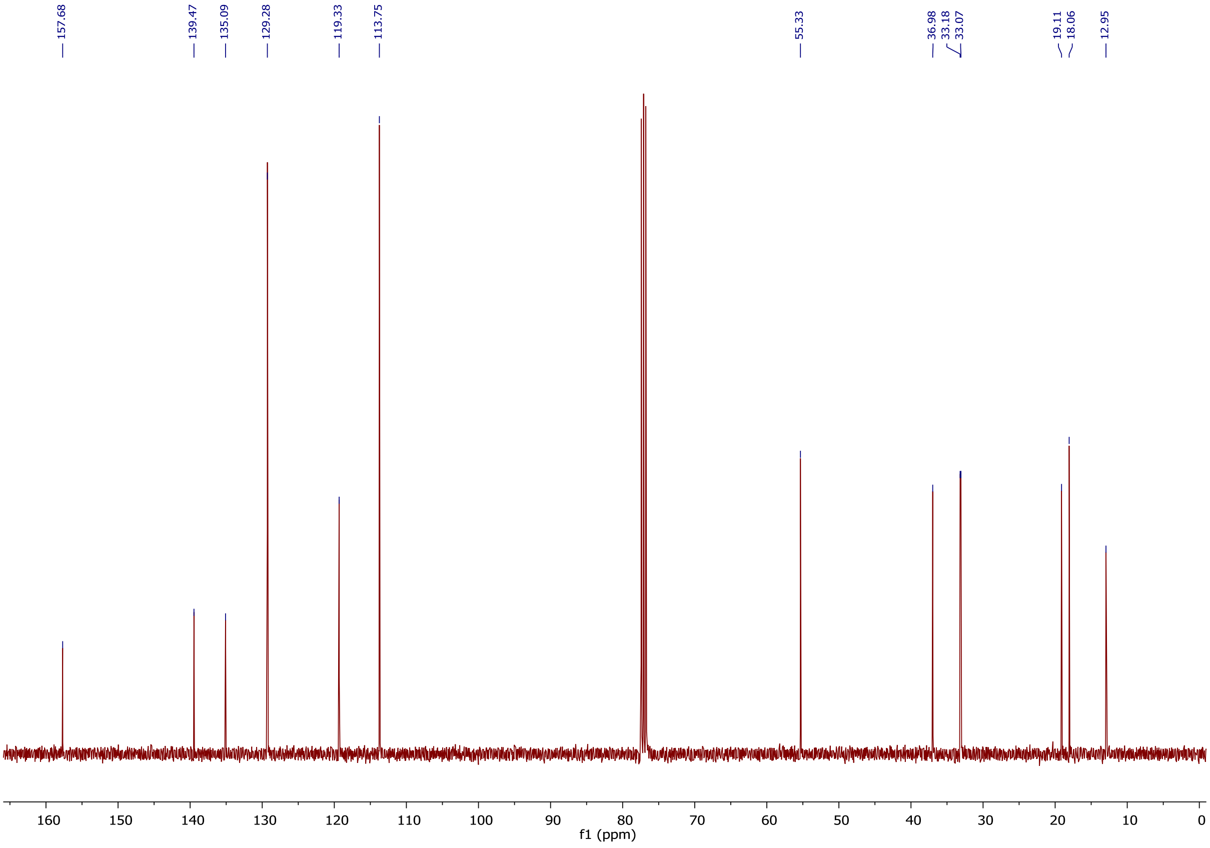
157.68
139.47
135.09
129.28
119.33
113.75
55.33
36.98
33.18
33.07
19.11
18.06
12.95
f1 (ppm)

1D nOe data obtained by irradiation of H_2: n.b. multiplicities of peaks for H_1 and H_3 lost as splitting results from coupling to the irradiated proton (H_2)

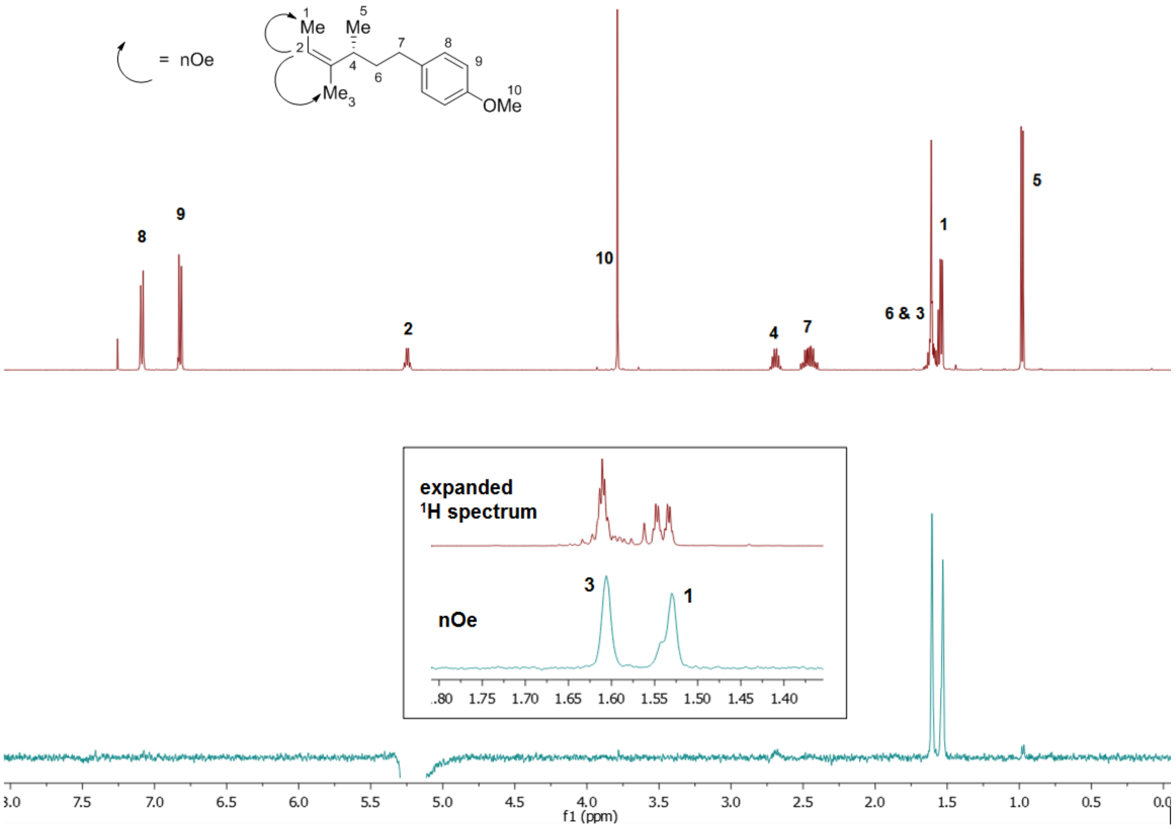

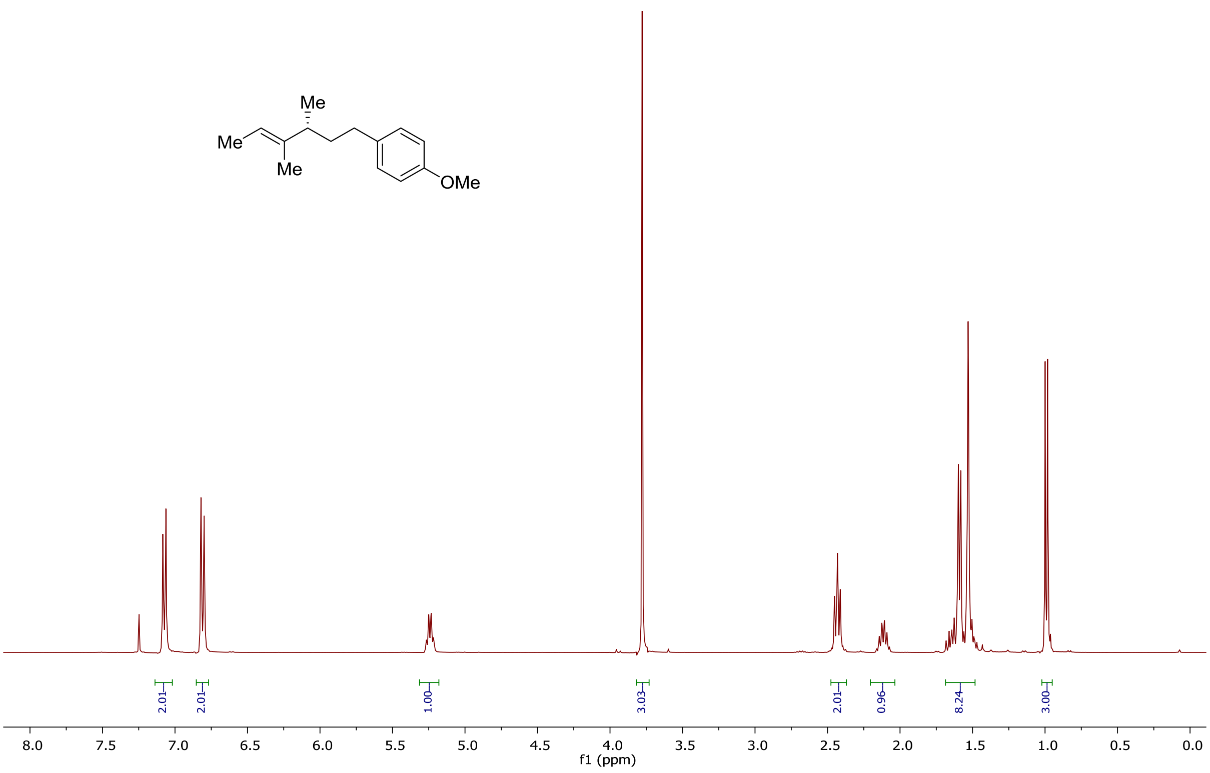
Me
Me
Me
OMe
2.01
2.01
1.00
3.03
2.01
0.96
8.24
3.00
8.0 7.5 7.0 6.5 6.0 5.5 5.0 4.5 4.0 3.5 3.0 2.5 2.0 1.5 1.0 0.5 0.0
f1 (ppm)

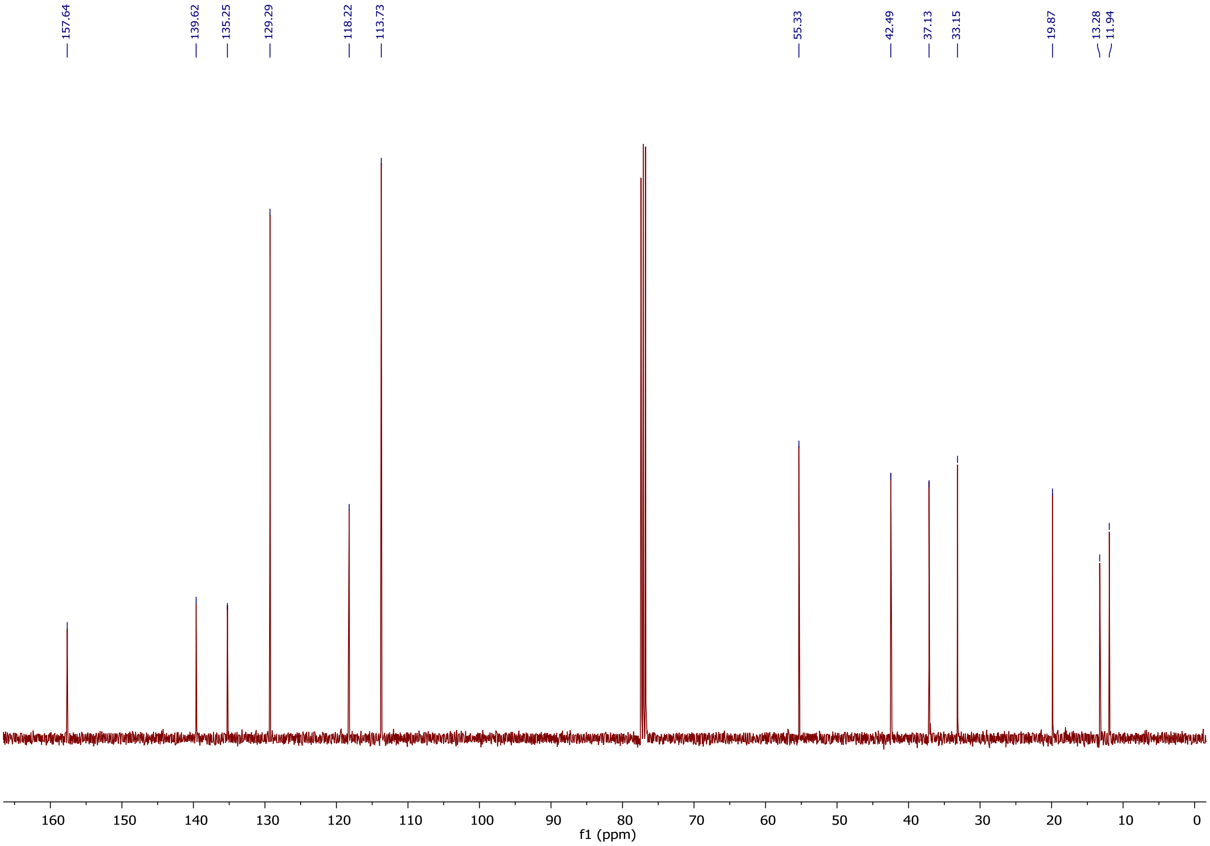
157.64
139.62
135.25
129.29
118.22
113.73
55.33
42.49
37.13
33.15
19.87
13.28
11.94
160 150 140 130 120 110 100 90 80 70 60 50 40 30 20 10 0
f1 (ppm)

1D nOe data obtained by irradiation of H_2: n.b. multiplicities of peaks for H_1 lost as splitting results from coupling to the irradiated proton (H_2)

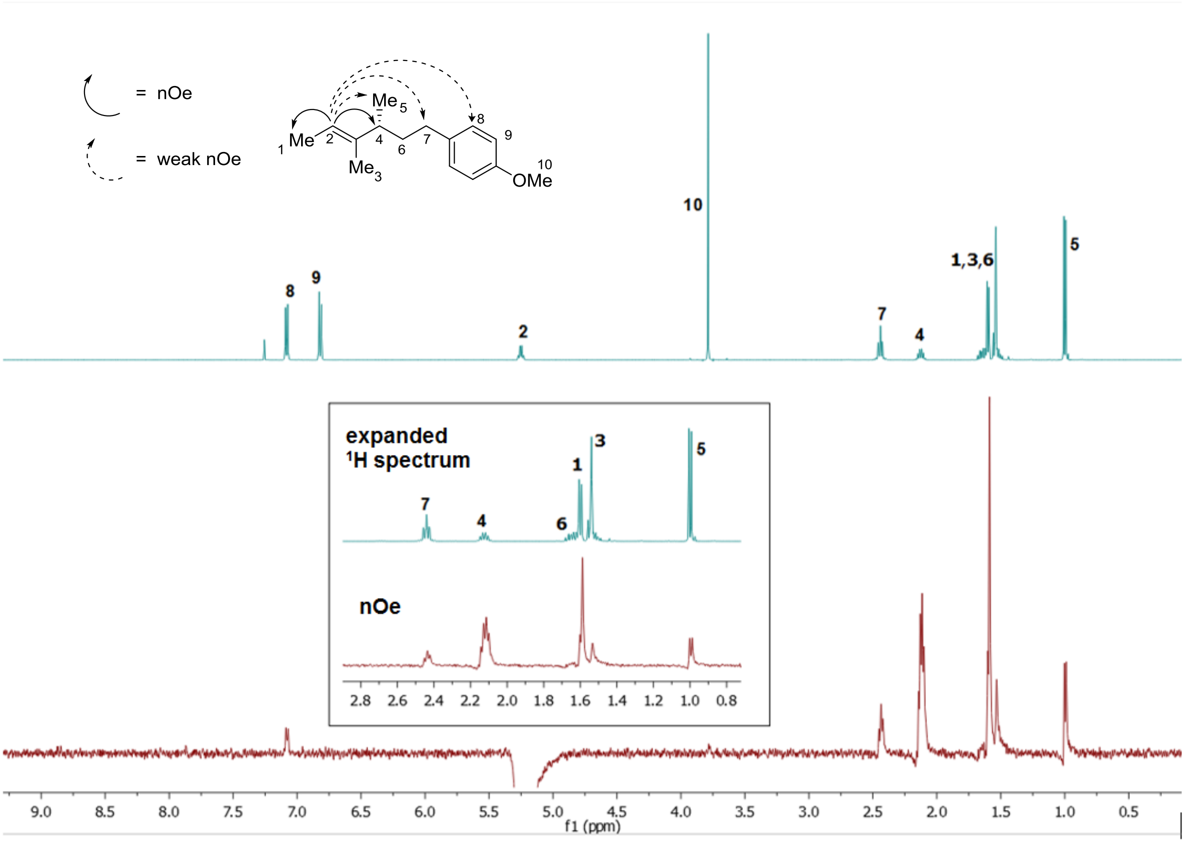

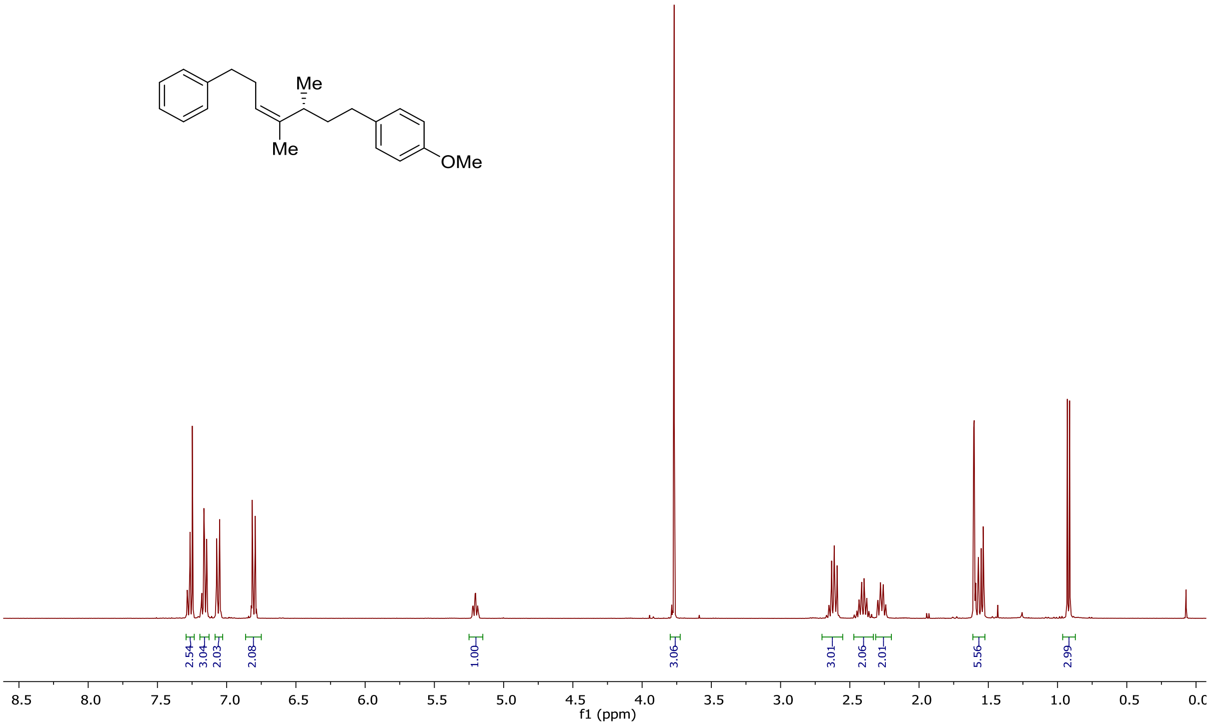
Me
Me
OMe
2.54
3.04
2.03
2.08
1.00
3.06
3.01
2.06
2.01
5.56
2.99
8.5 8.0 7.5 7.0 6.5 6.0 5.5 5.0 4.5 4.0 3.5 3.0 2.5 2.0 1.5 1.0 0.5 0.0
f1 (ppm)

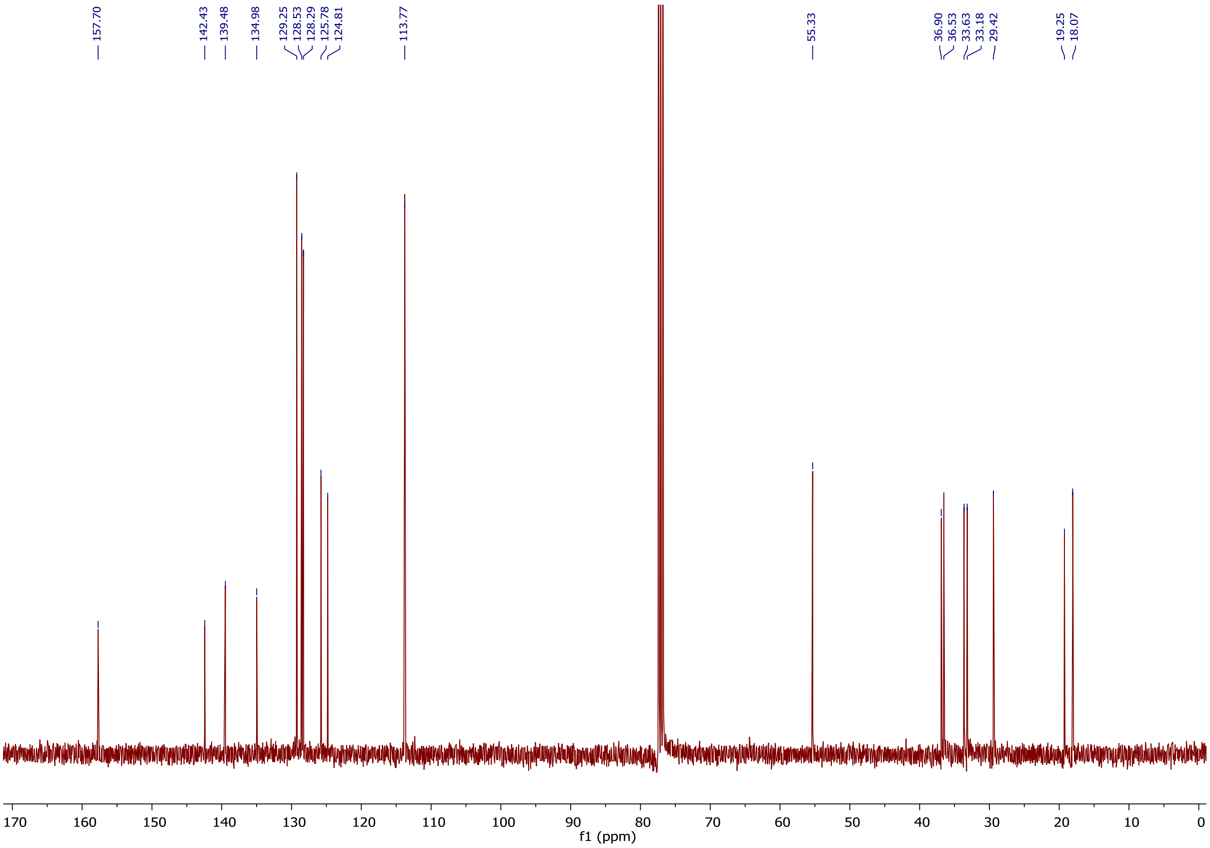
157.70
142.43
139.48
134.98
129.25
128.53
128.29
125.78
124.81
113.77
55.33
36.90
36.53
33.63
33.18
29.42
19.25
18.07
170 160 150 140 130 120 110 100 90 80 70 60 50 40 30 20 10 0
f1 (ppm)

1D nOe data obtained by irradiation of H_6:

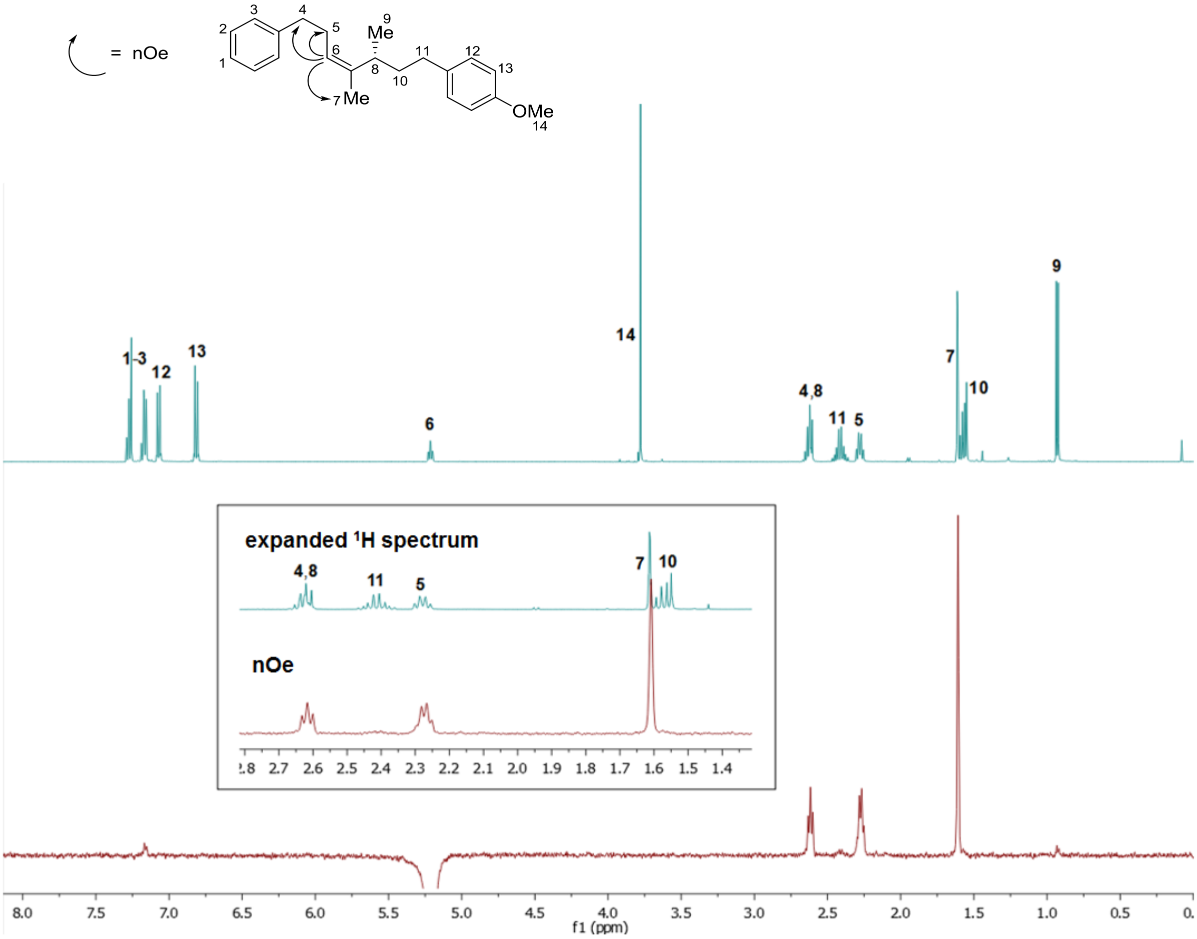

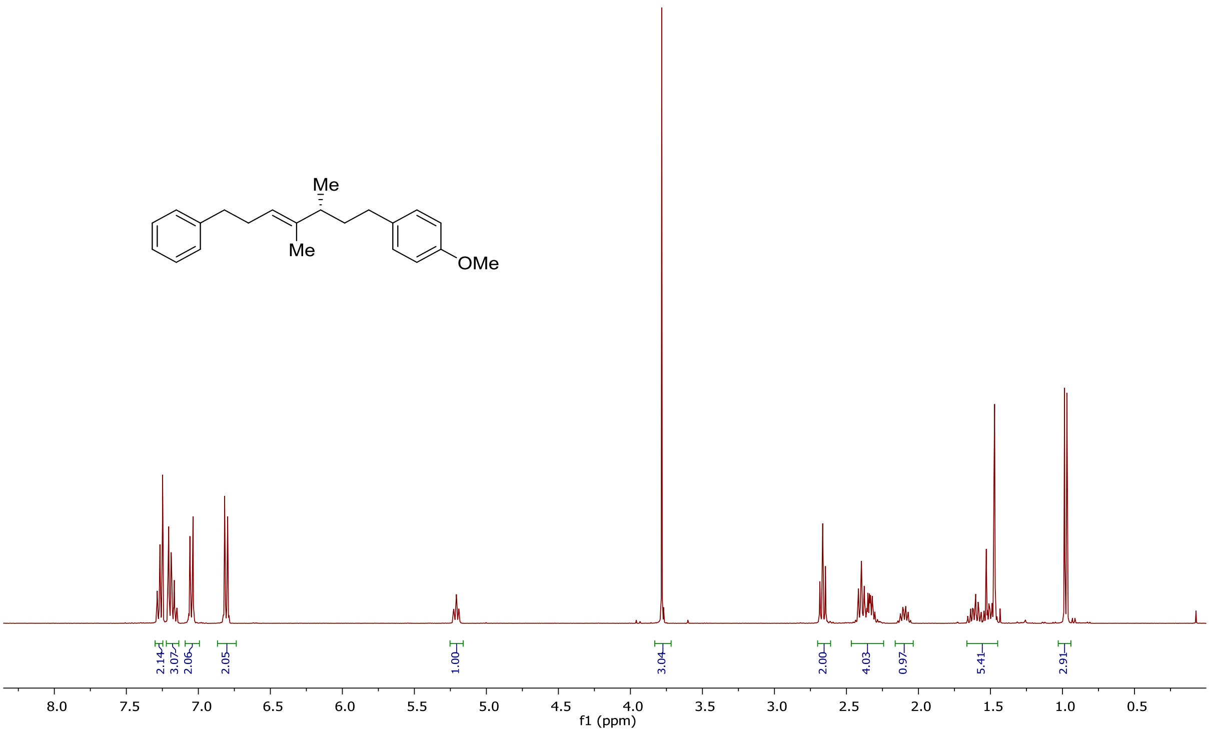
Me
Me
OMe
2.14
3.07
2.06
2.05
1.00
3.04
2.00
4.03
0.97
5.41
2.91
8.0 7.5 7.0 6.5 6.0 5.5 5.0 4.5 4.0 3.5 3.0 2.5 2.0 1.5 1.0 0.5
f1 (ppm)

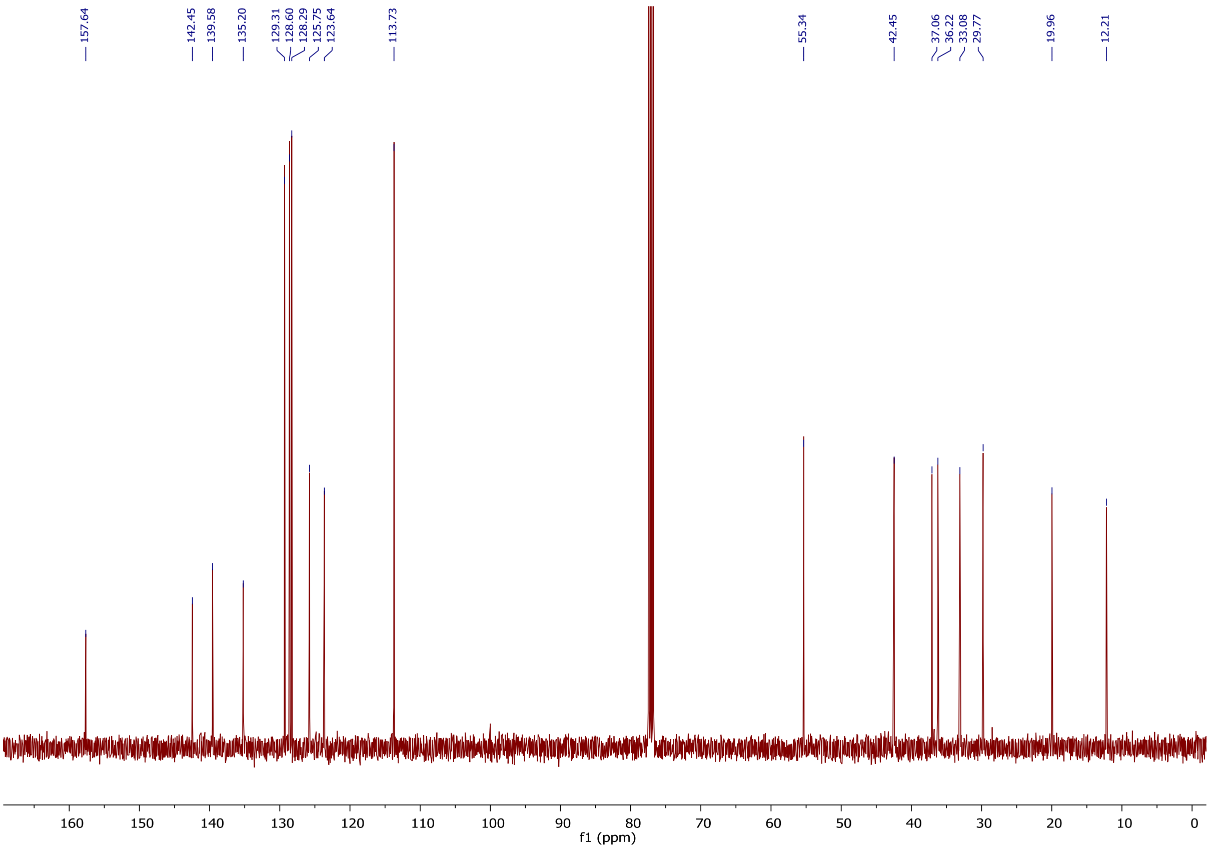
157.64
142.45
139.58
135.20
129.31
128.60
128.29
125.75
123.64
113.73
55.34
42.45
37.06
36.22
33.08
29.77
19.96
12.21
160 150 140 130 120 110 100 90 80 70 60 50 40 30 20 10 0
f1 (ppm)

1D nOe data obtained by irradiation of H_6:

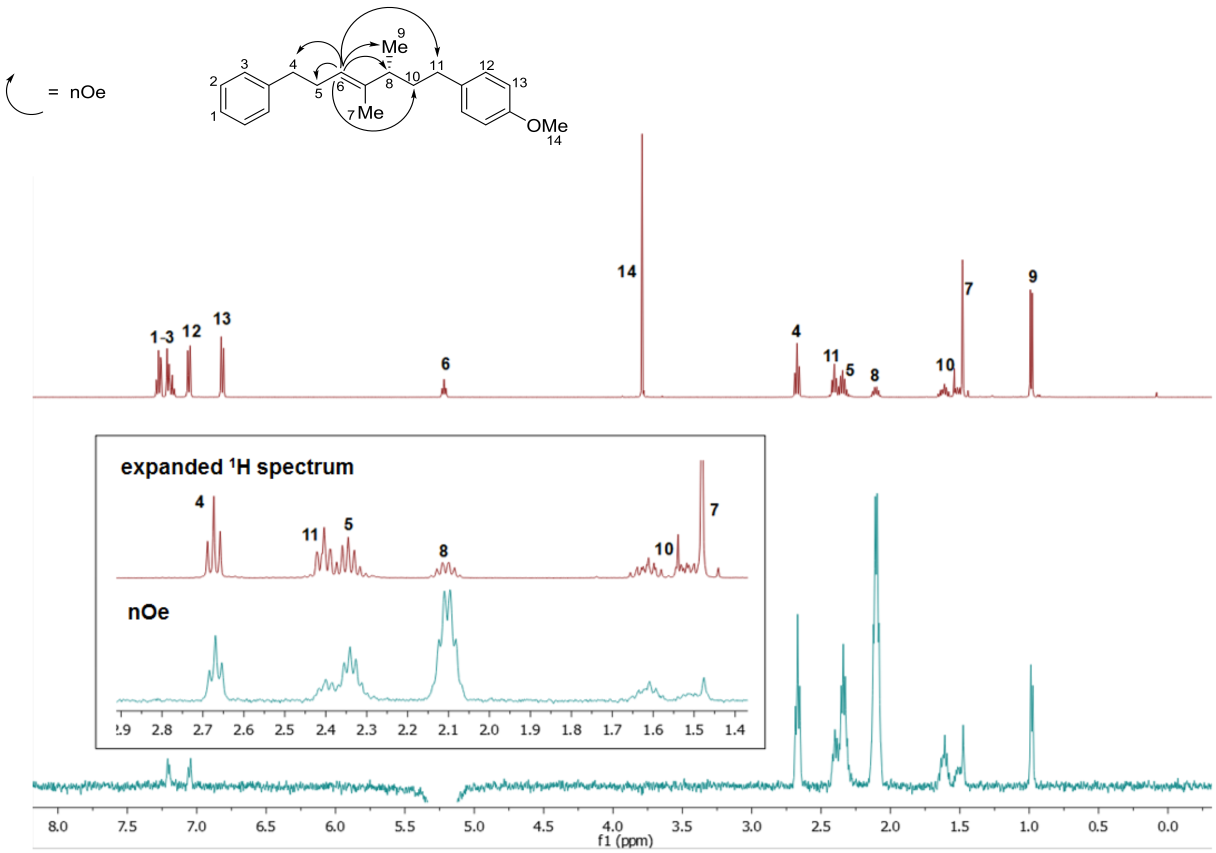

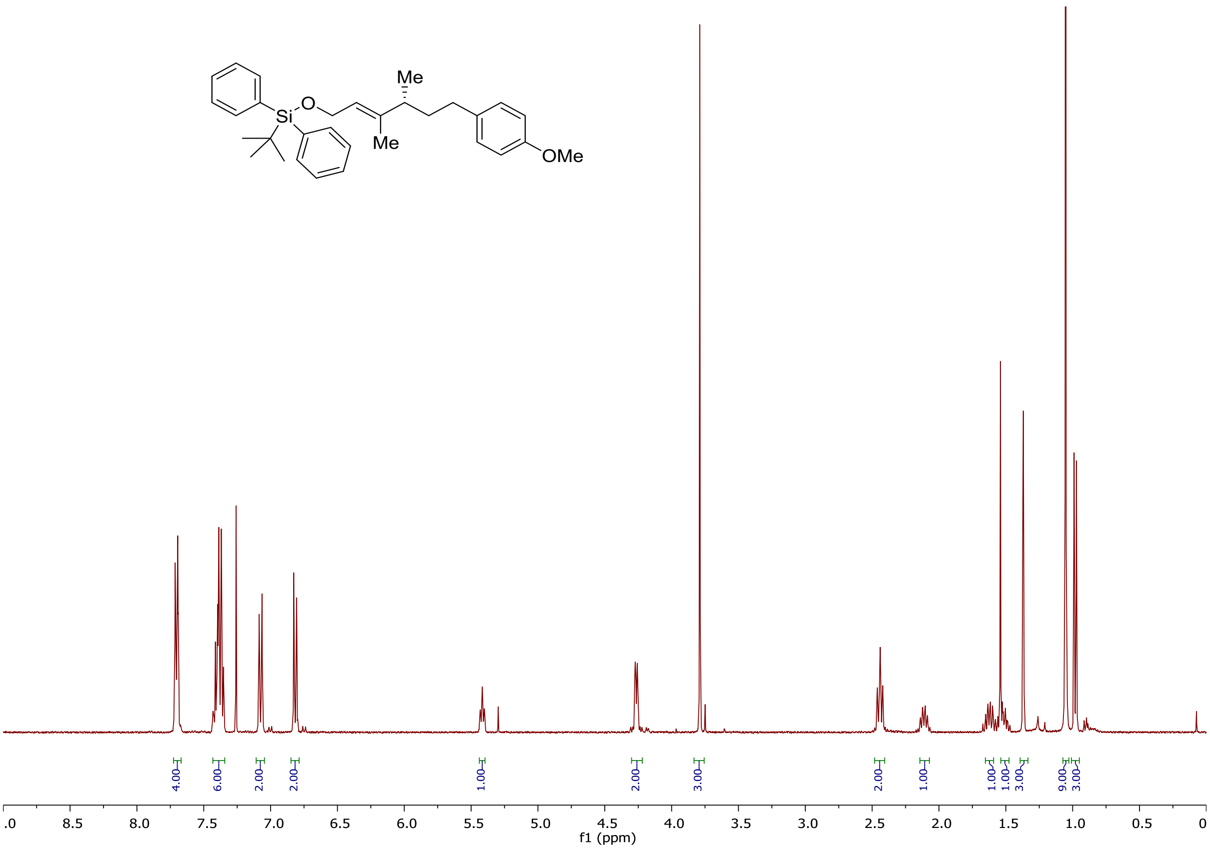
Si
O
Me
Me
OMe
4.00
6.00
2.00
2.00
1.00
2.00
3.00
2.00
1.00
1.00
1.00
3.00
9.00
3.00
f1 (ppm)

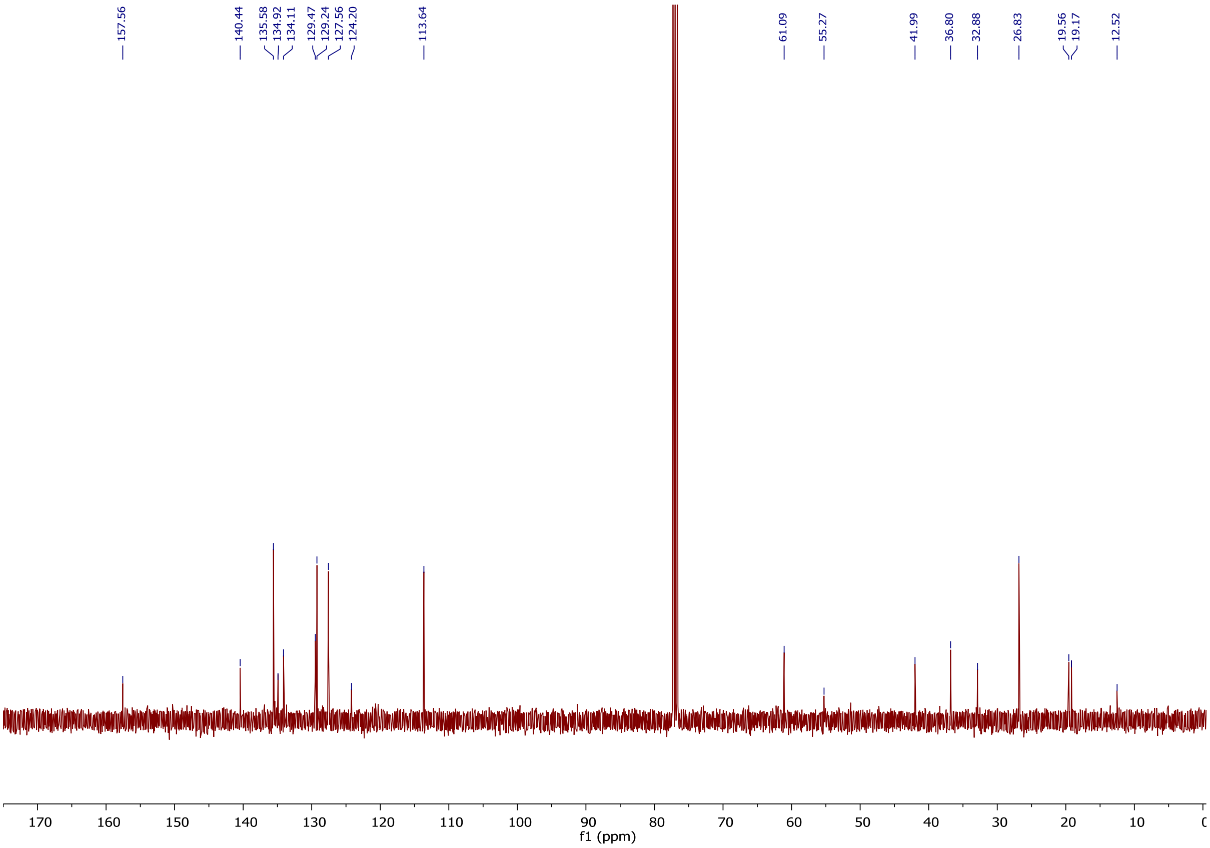
157.56
140.44
135.58
134.92
134.11
129.47
129.24
127.56
124.20
113.64
61.09
55.27
41.99
36.80
32.88
26.83
19.56
19.17
12.52
f1 (ppm)

1D nOe data obtained by irradiation of H_6:

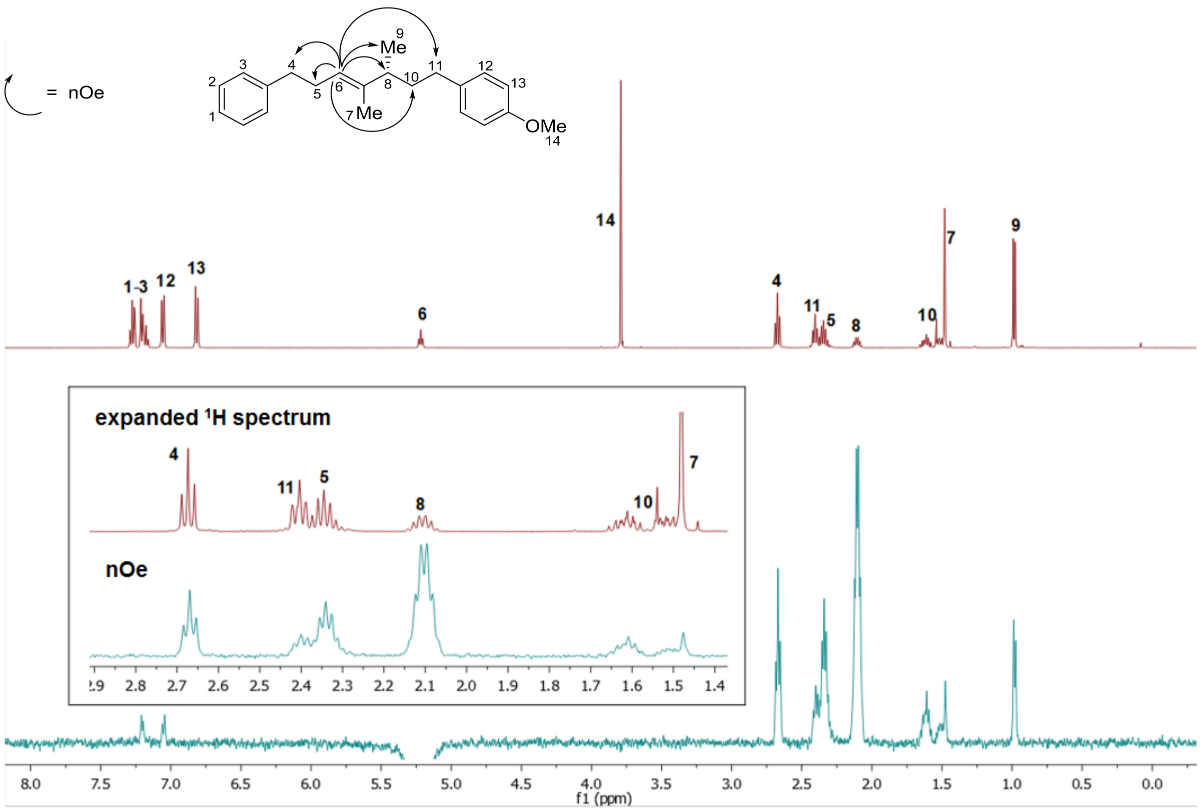

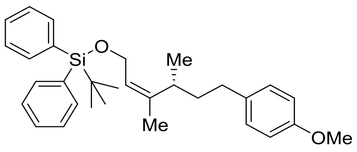

Si
O
Me
Me
OMe

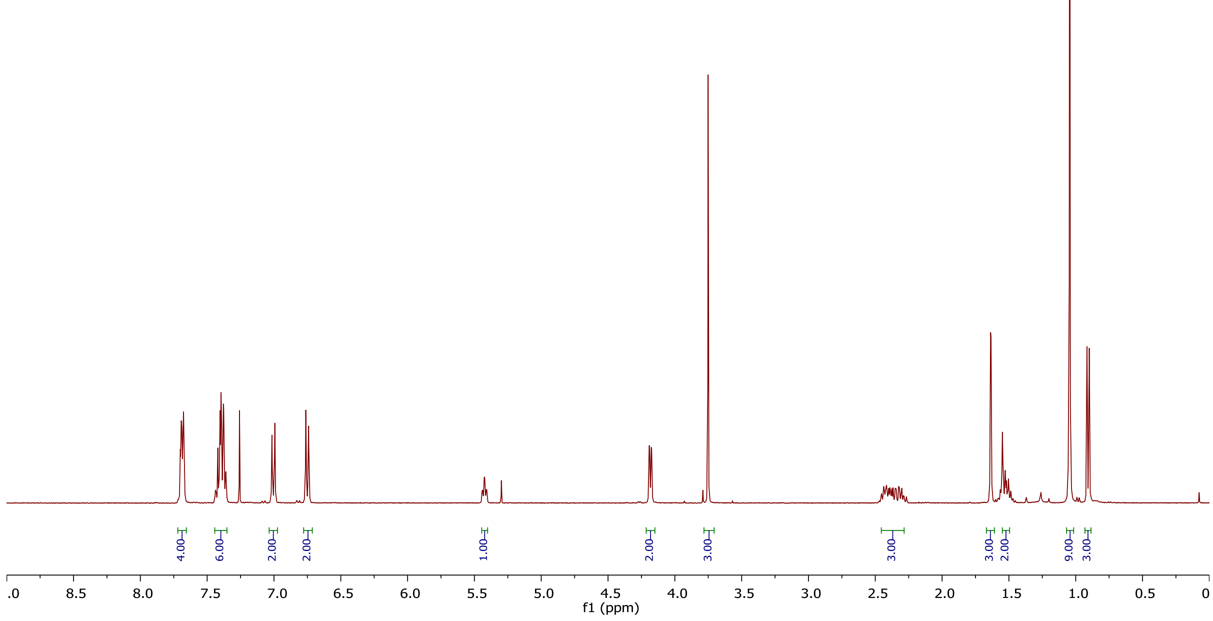

4.00
6.00
2.00
2.00
1.00
2.00
3.00
3.00
3.00
2.00
9.00
3.00
f1 (ppm)

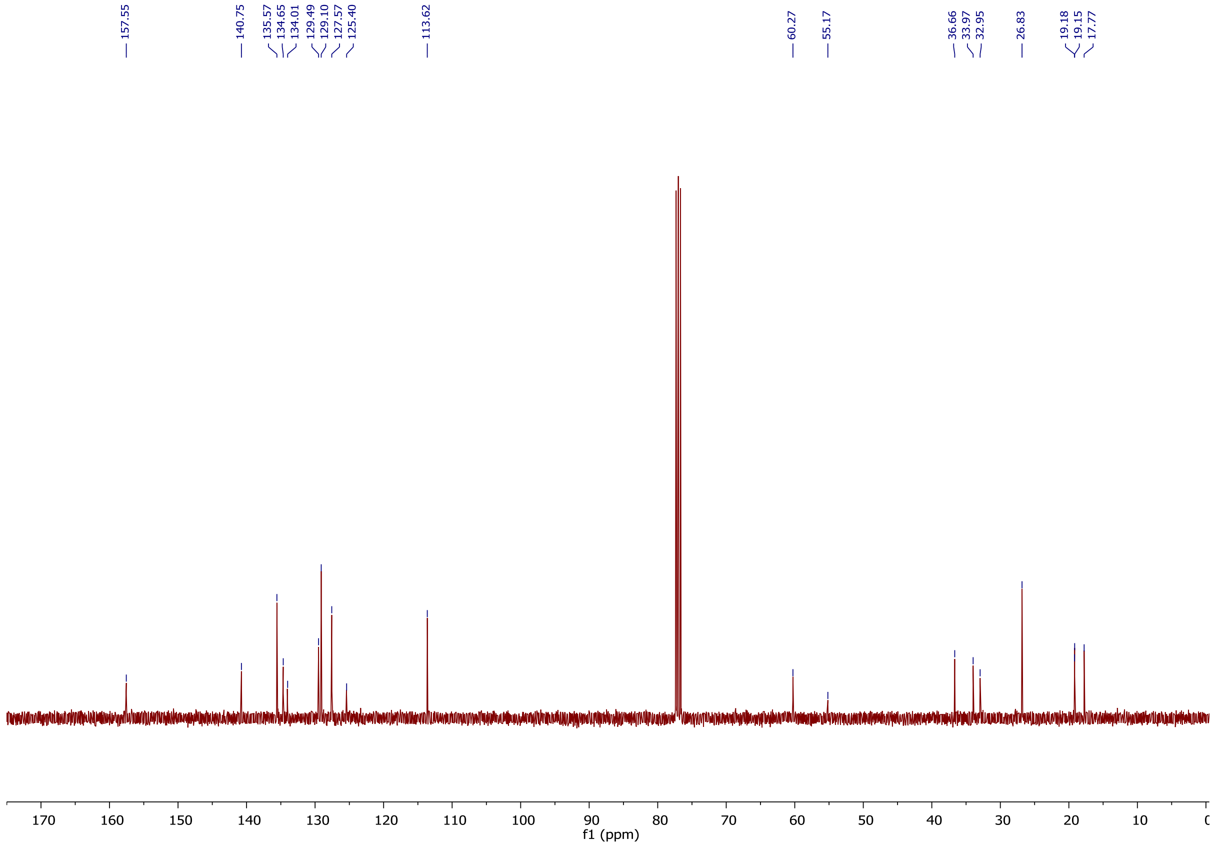

157.55
140.75
135.57
134.65
134.01
129.49
129.10
127.57
125.40
113.62
60.27
55.17
36.66
33.97
32.95
26.83
19.18
19.15
17.77
f1 (ppm)

1D nOe data obtained by irradiation of H_6:

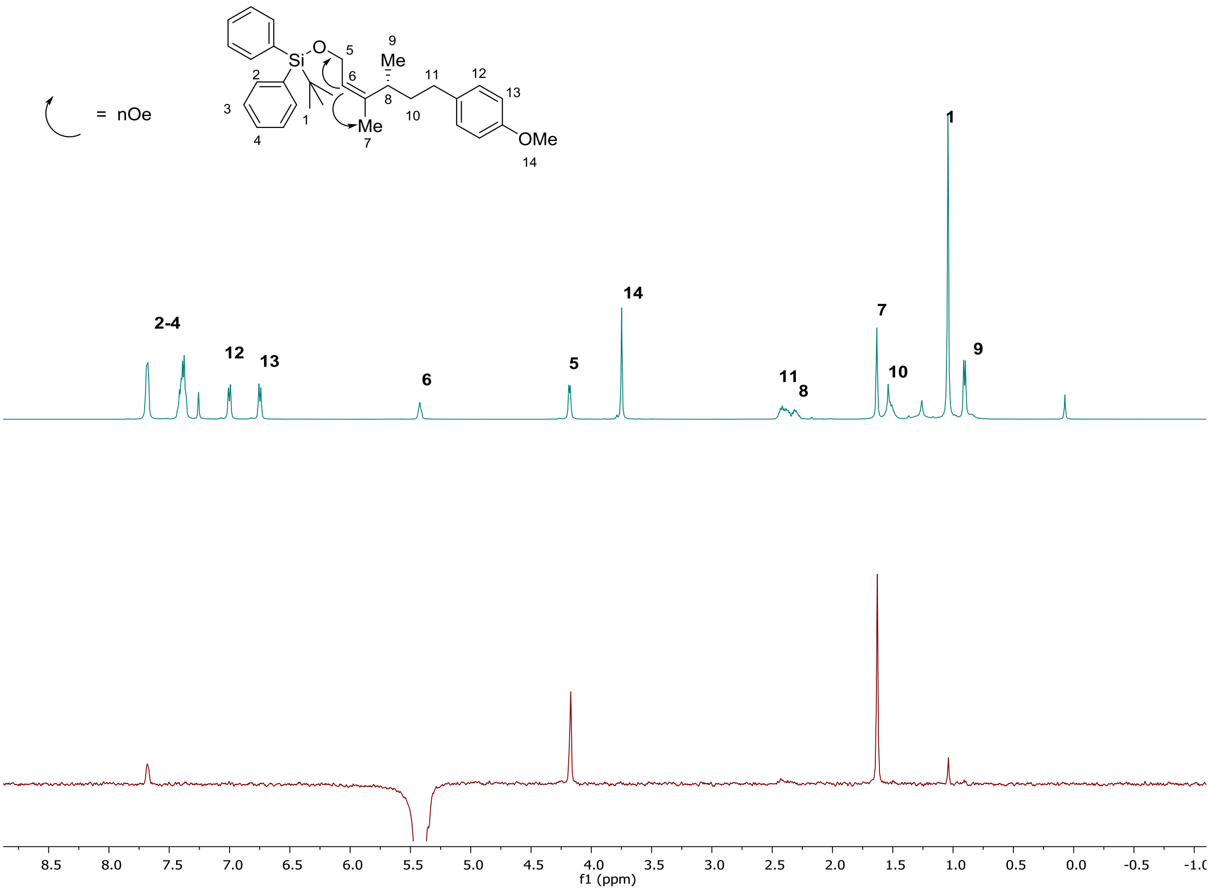

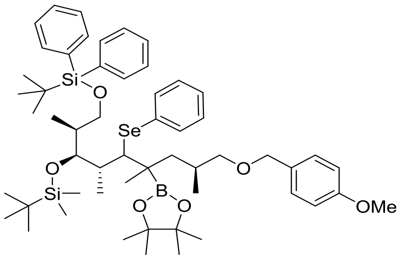
Si
O
Se
O
Si
B
O
O
O
OMe

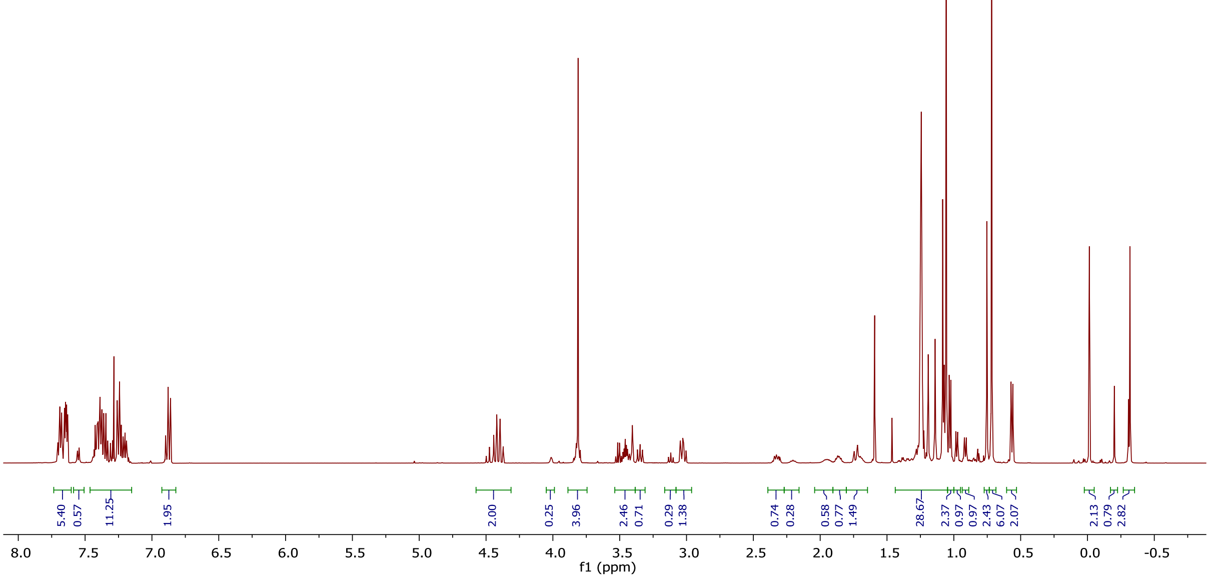
5.40
0.57
11.25
1.95
2.00
0.25
3.96
2.46
0.71
0.29
1.38
0.74
0.28
0.58
0.77
1.49
28.67
2.37
0.97
0.97
2.43
6.07
2.07
2.13
0.79
2.82
8.0 7.5 7.0 6.5 6.0 5.5 5.0 4.5 4.0 3.5 3.0 2.5 2.0 1.5 1.0 0.5 0.0 -0.5
f1 (ppm)

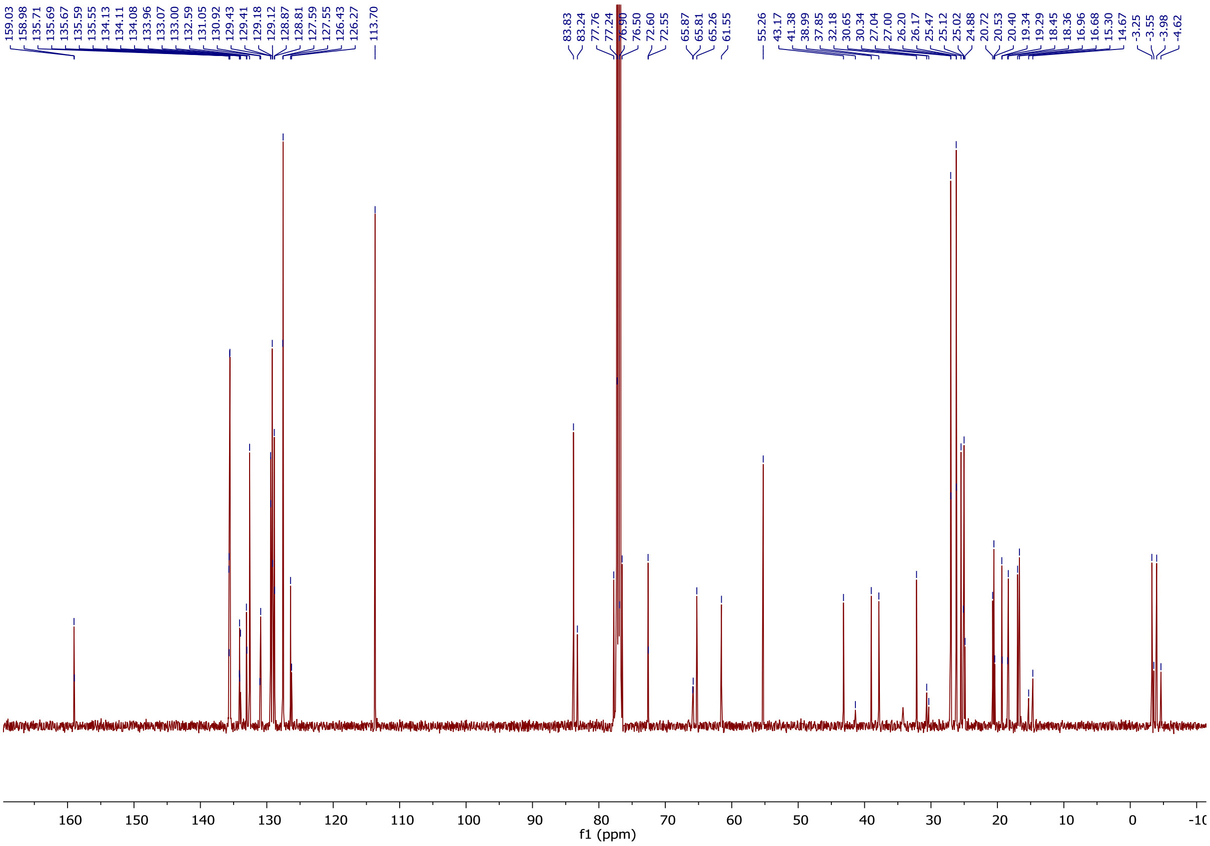
159.03
158.98
135.71
135.69
135.67
135.59
135.55
134.13
134.11
134.08
133.96
133.07
133.00
132.59
131.05
130.92
129.43
129.41
129.18
129.12
128.87
128.81
127.59
127.55
126.43
126.27
113.70
83.83
83.24
77.76
77.24
76.90
76.50
72.60
72.55
65.87
65.81
65.26
61.55
55.26
43.17
41.38
38.99
37.85
32.18
30.65
30.34
27.04
27.00
26.20
26.17
25.47
25.12
25.02
24.88
20.72
20.53
20.40
19.34
19.29
18.45
18.36
16.96
16.68
15.30
14.67
-3.25
-3.55
-3.98
-4.62
160 150 140 130 120 110 100 90 80 70 60 50 40 30 20 10 0 -10
f1 (ppm)

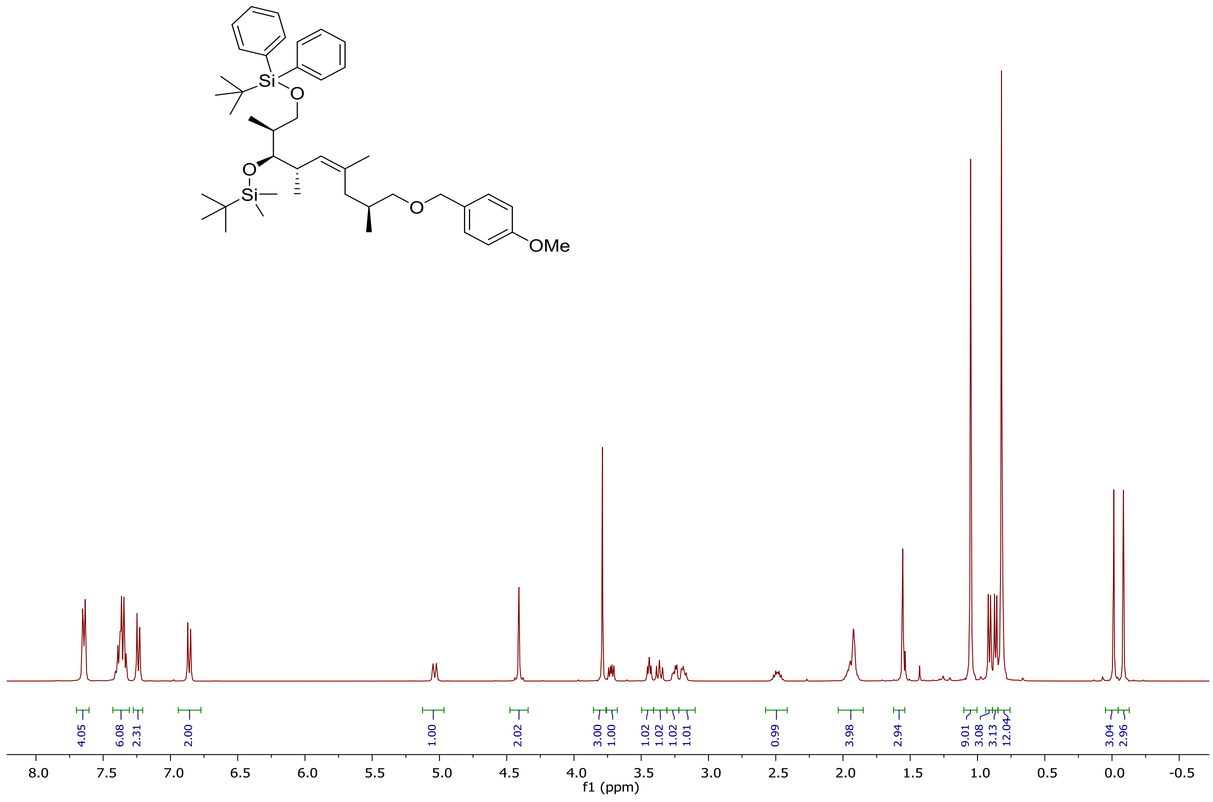

4.05
6.08
2.31
2.00
1.00
2.02
3.00
1.00
1.02
1.02
1.02
1.01
0.99
3.98
2.94
9.01
3.08
3.13
12.04
3.04
2.96
f1 (ppm)

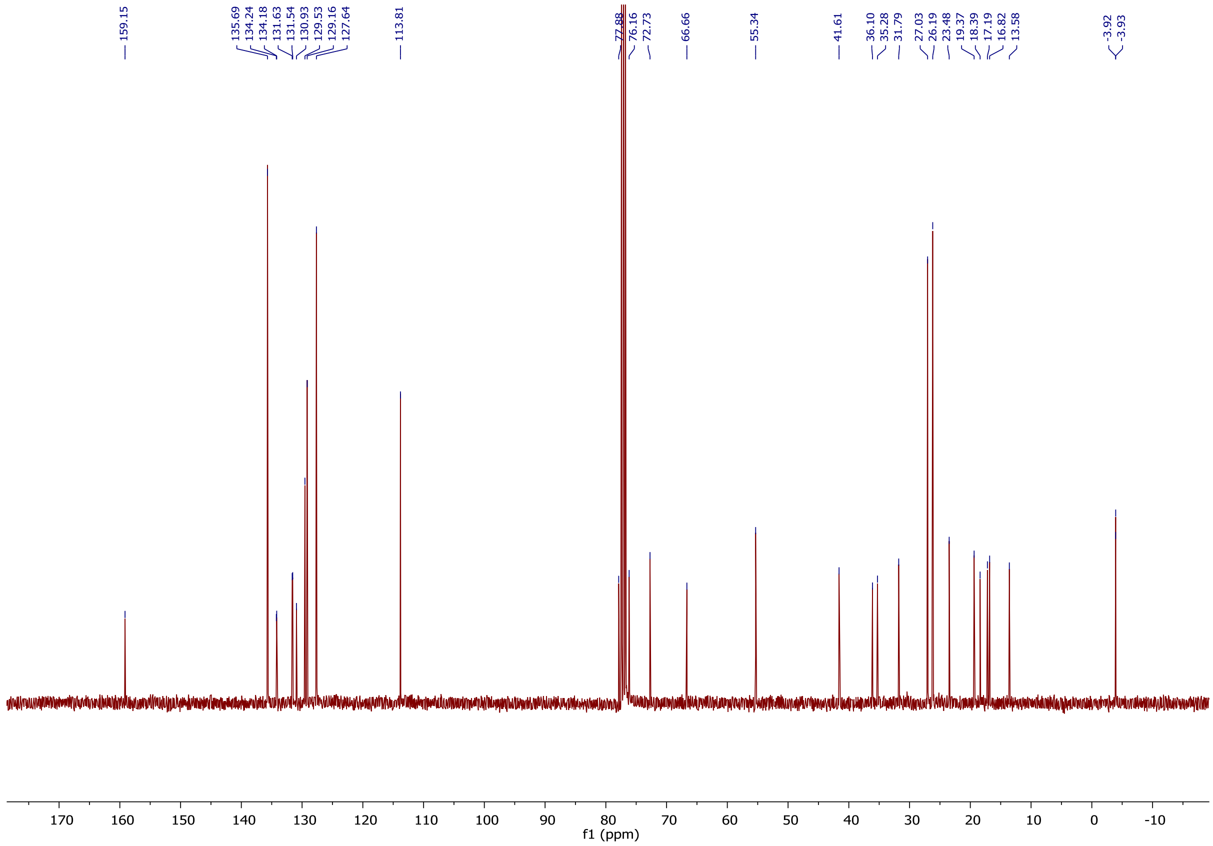

159.15
135.69
134.24
134.18
131.63
131.54
130.93
129.53
129.16
127.64
113.81
77.88
76.16
72.73
66.66
55.34
41.61
36.10
35.28
31.79
27.03
26.19
23.48
19.37
18.39
17.19
16.82
13.58
-3.92
-3.93
f1 (ppm)

1D nOe data obtained by irradiation of H_{10}:

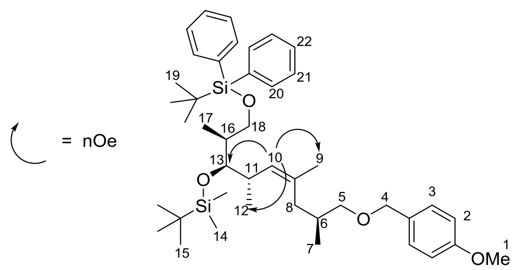

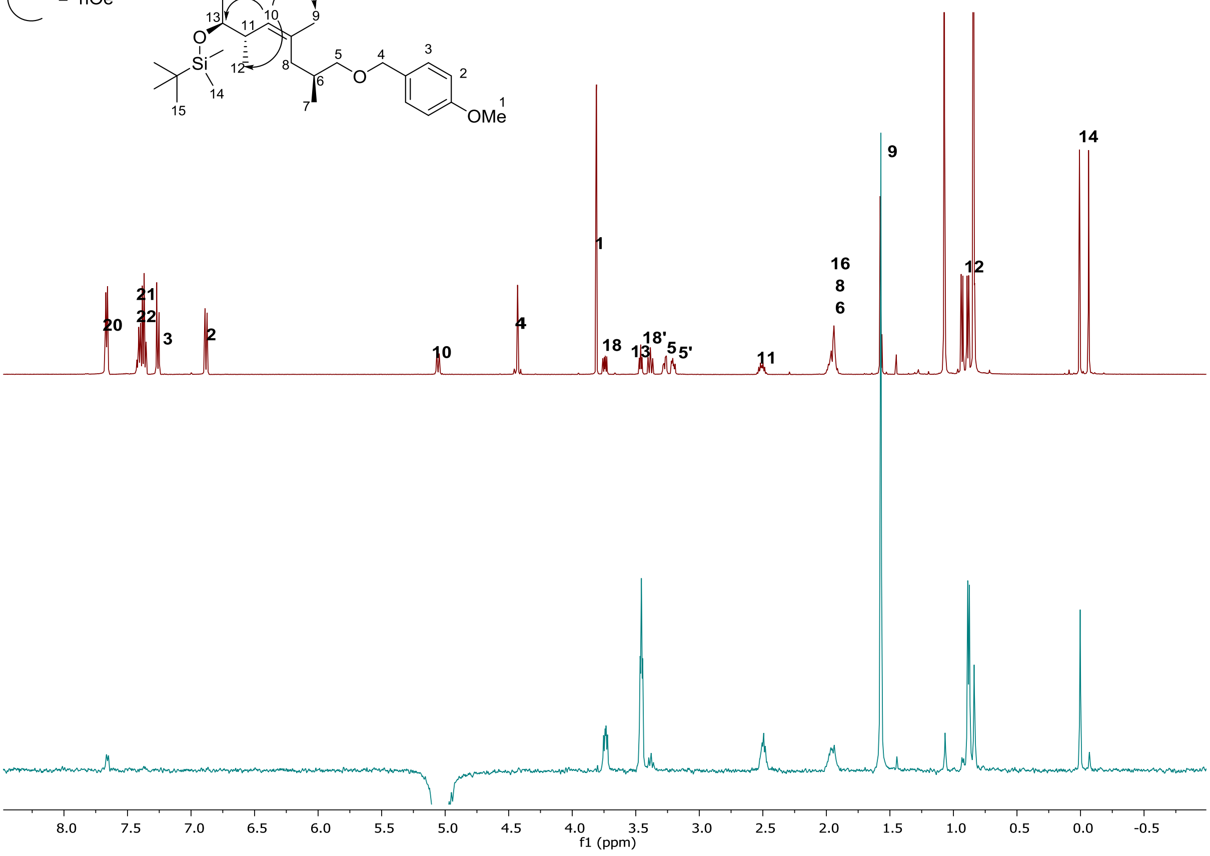

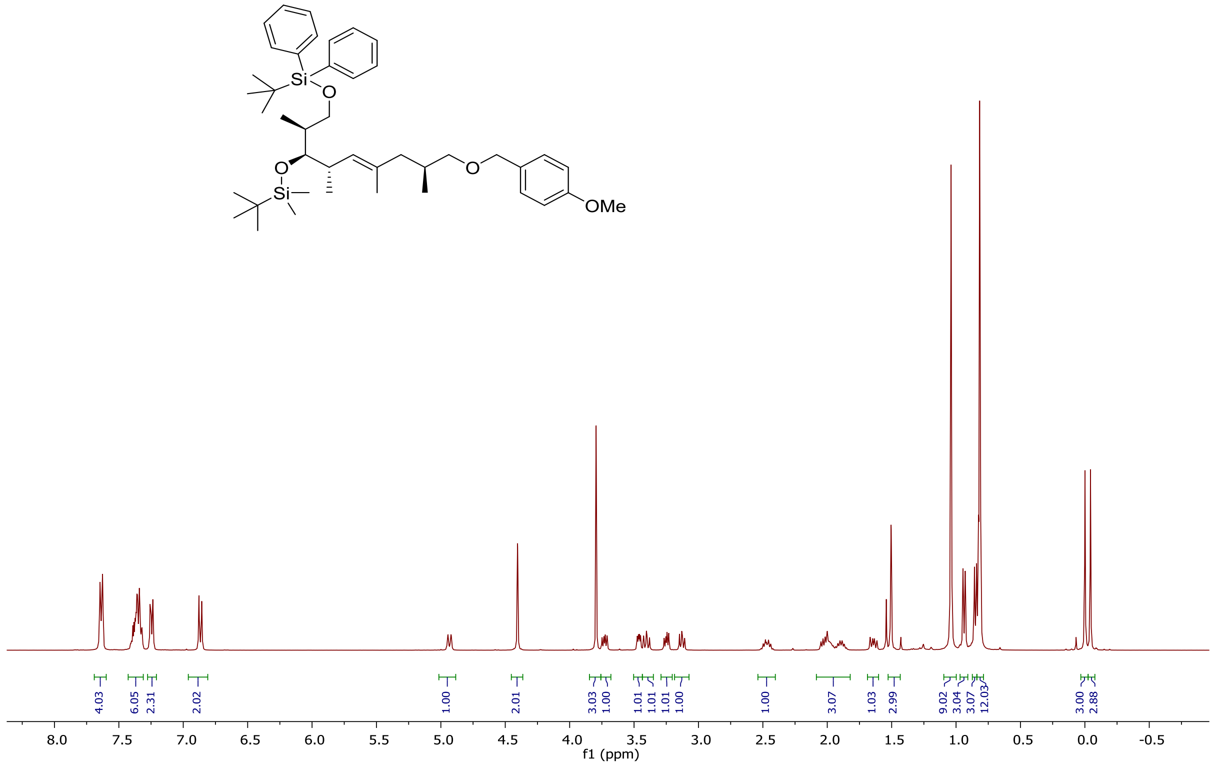

Si
O
O
Si
O
OMe
4.03
6.05
2.31
2.02
1.00
2.01
3.03
1.00
1.01
1.01
1.01
1.00
1.00
3.07
1.03
2.99
9.02
3.04
3.07
12.03
3.00
2.88
8.0 7.5 7.0 6.5 6.0 5.5 5.0 4.5 4.0 3.5 3.0 2.5 2.0 1.5 1.0 0.5 0.0 -0.5
f1 (ppm)

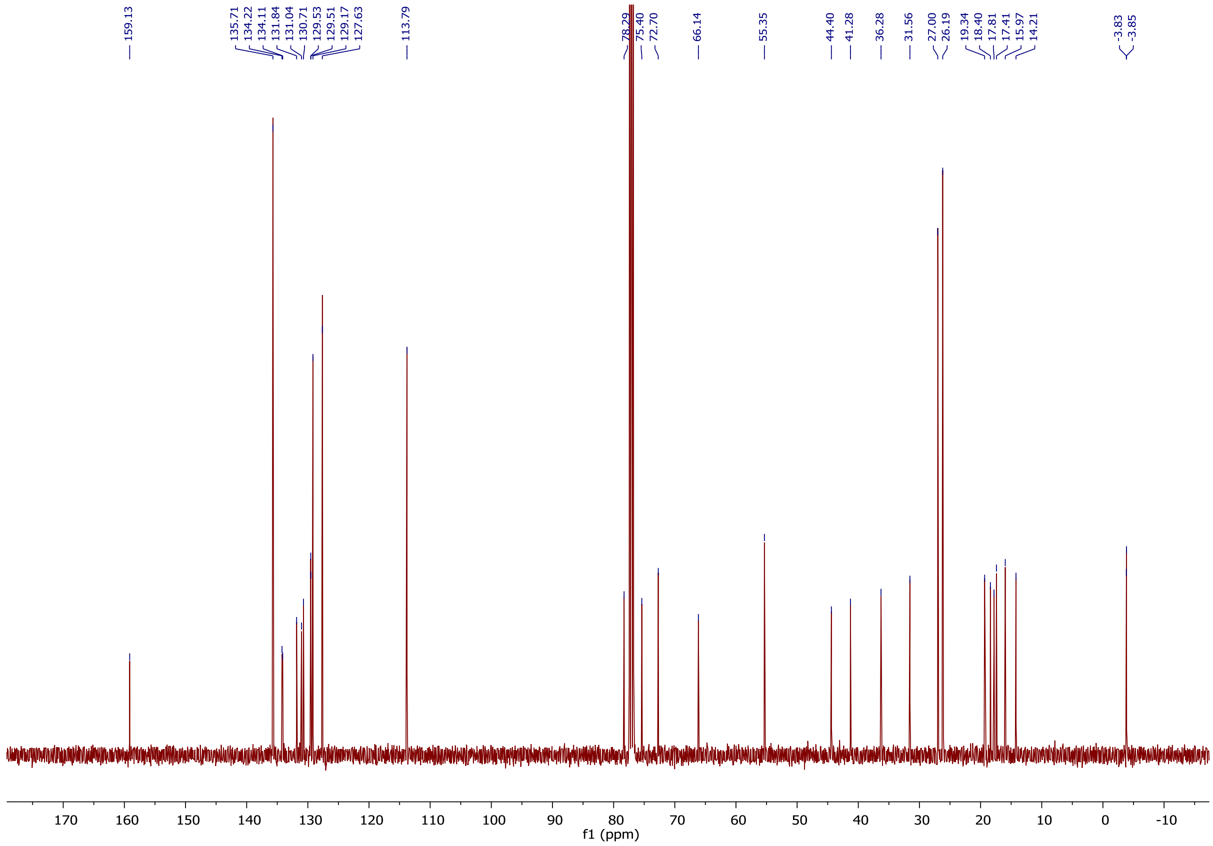

159.13
135.71
134.22
134.11
131.84
131.04
130.71
129.53
129.51
129.17
127.63
113.79
78.29
75.40
72.70
66.14
55.35
44.40
41.28
36.28
31.56
27.00
26.19
19.34
18.40
17.81
17.41
15.97
14.21
-3.83
-3.85
170 160 150 140 130 120 110 100 90 80 70 60 50 40 30 20 10 0 -10
f1 (ppm)

1D nOe data obtained by irradiation of H_{10}:

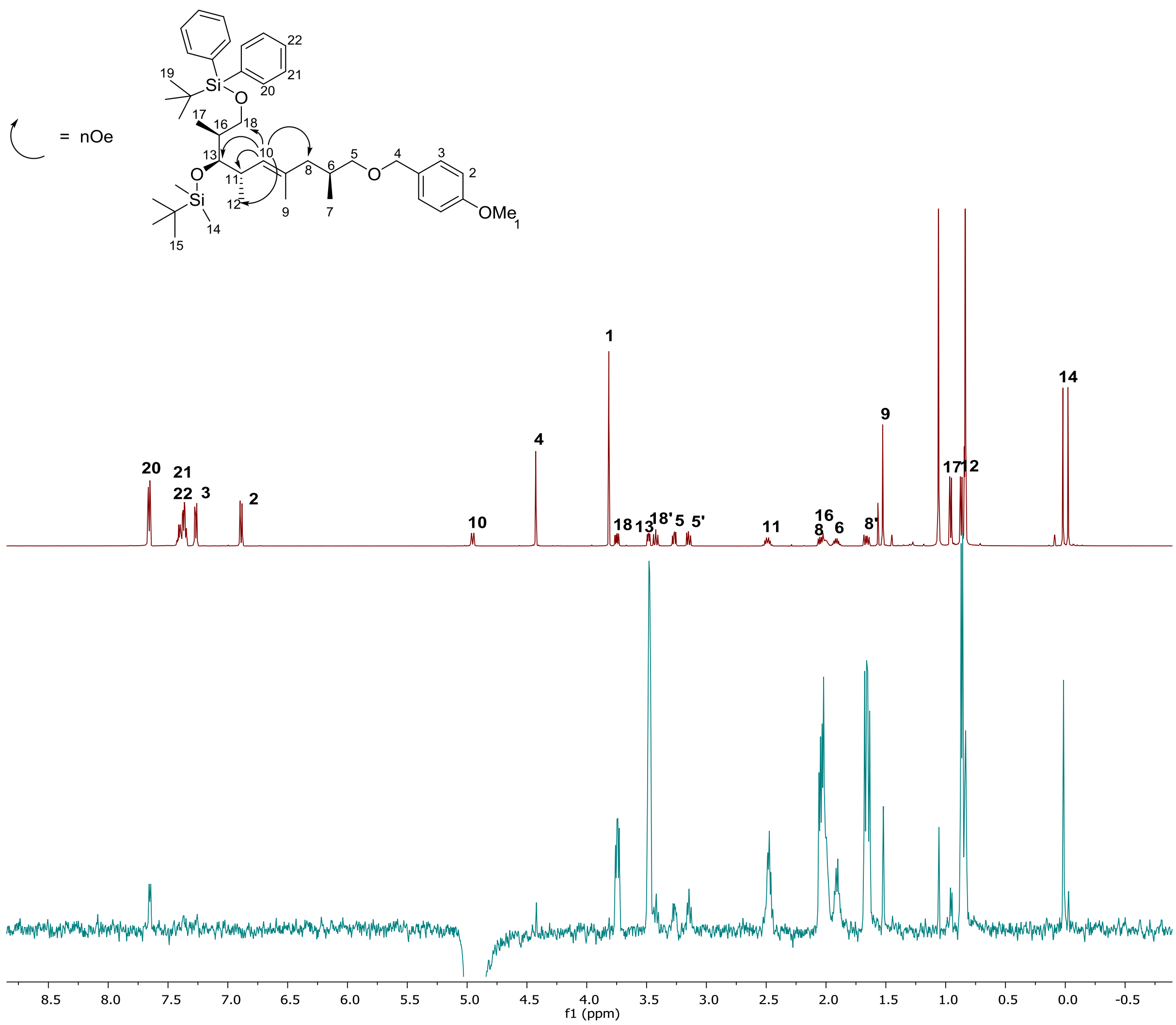

Supplement: Supplementary file 1 — Supplementary [file ANIE-56-786-s001.pdf]
